# Mantis-ml: Disease-Agnostic Gene Prioritization from High-Throughput Genomic Screens by Stochastic Semi-supervised Learning

**DOI:** 10.1016/j.ajhg.2020.03.012

**Published:** 2020-05-07

**Authors:** Dimitrios Vitsios, Slavé Petrovski

**Affiliations:** 1 Centre for Genomics Research, Discovery Sciences, BioPharmaceuticals R&D, AstraZeneca, 1 Francis Crick Avenue, CB2 0RE Cambridge, UK

**Keywords:** mantis-ml, machine-learning, genomics, gene-prioritization, positive-unlabeled learning, auto-ML, tensorflow, keras

## Abstract

Access to large-scale genomics datasets has increased the utility of hypothesis-free genome-wide analyses. However, gene signals are often insufficiently powered to reach experiment-wide significance, triggering a process of laborious triaging of genomic-association-study results. We introduce mantis-ml, a multi-dimensional, multi-step machine-learning framework that allows objective assessment of the biological relevance of genes to disease studies. Mantis-ml is an automated machine-learning framework that follows a multi-model approach of stochastic semi-supervised learning to rank disease-associated genes through iterative learning sessions on random balanced datasets across the protein-coding exome. When applied to a range of human diseases, including chronic kidney disease (CKD), epilepsy, and amyotrophic lateral sclerosis (ALS), mantis-ml achieved an average area under curve (AUC) prediction performance of 0.81–0.89. Critically, to prove its value as a tool that can be used to interpret exome-wide association studies, we overlapped mantis-ml predictions with data from published cohort-level association studies. We found a statistically significant enrichment of high mantis-ml predictions among the highest-ranked genes from hypothesis-free cohort-level statistics, indicating a substantial improvement over the performance of current state-of-the-art methods and pointing to the capture of true prioritization signals for disease-associated genes. Finally, we introduce a generic mantis-ml score (GMS) trained with over 1,200 features as a generic-disease-likelihood estimator, outperforming published gene-level scores. In addition to our tool, we provide a gene prioritization atlas that includes mantis-ml’s predictions across ten disease areas and empowers researchers to interactively navigate through the gene-triaging framework. Mantis-ml is an intuitive tool that supports the objective triaging of large-scale genomic discovery studies and enhances our understanding of complex genotype-phenotype associations.

## Introduction

As a result of the vast interrogation of the protein-coding genome, the global research community has generated an extended amount of resources related to tissue-specific gene expression, intolerance to genetic variation, model organism function, and various other diverse annotation types. Additionally, it is evident that complex phenotypes, such as disease phenotypes, cannot be explained by the variability of a single data type (e.g., expression in tissue or animal models) but rather require the combination of a multitude of data types and resources that describe multiple aspects of the phenotype at different dimensions.^1^, ^2^, ^3^

The underlying biology of human disease is complex, and current knowledge provides a limited view of the full collection of disease-associated genes. We sought to explore this issue by leveraging the rich collection of well-curated gene-level annotations to identify patterns that are shared among genes associated with a disease and leverage those patterns to predict putatively novel genes of interest that have the most similar profiles and, thus, might also be associated with disease.

To achieve this goal, for each gene we harvested diverse types of information, including gene expression;^4^ human disease literature;^5^ mouse phenotypes;^6^ proteomic;^7^ interactome;^8^ and genic metrics of human-lineage purifying selection^9^, ^10^, ^11^ (see Supplemental Methods). Next, we developed mantis-ml, a machine-learning framework that can be applied to any disorder, given a starting set of genes that are associated with disease according to the Human Phenotype Ontology (HPO).^5^ Mantis-ml’s gene predictions without HPO-based annotations for the disease of interest are characterized as ‘novel’ throughout this work. The mantis-ml framework is based on a stochastic semi-supervised learning approach that solves inherent challenges presented by the problem of the high class imbalance in a finite space of data points (see Supplemental Methods).

Unlike other published gene-prioritization methods, we provide validation of our method’s predictions against results from real cohort genetic studies. The cohort statistics from these analyses refer to gene-level statistics emerging from rare-variant genetic-association studies.^12^ Specifically, we apply the predictions from our tool, mantis-ml, to the results of published exome-wide-association statistics and show a striking preferential enrichment of mantis-ml-predicted genes among the genes achieving the lowest p values in the respective case-control studies of those diseases. The three diverse disorders that we highlight as applications of mantis-ml are amyotrophic lateral sclerosis (ALS),^13^ chronic kidney disease (CKD),^14^ and epilepsy.^15^

## Methods

### Feature Pre-processing

**Figure 1:**
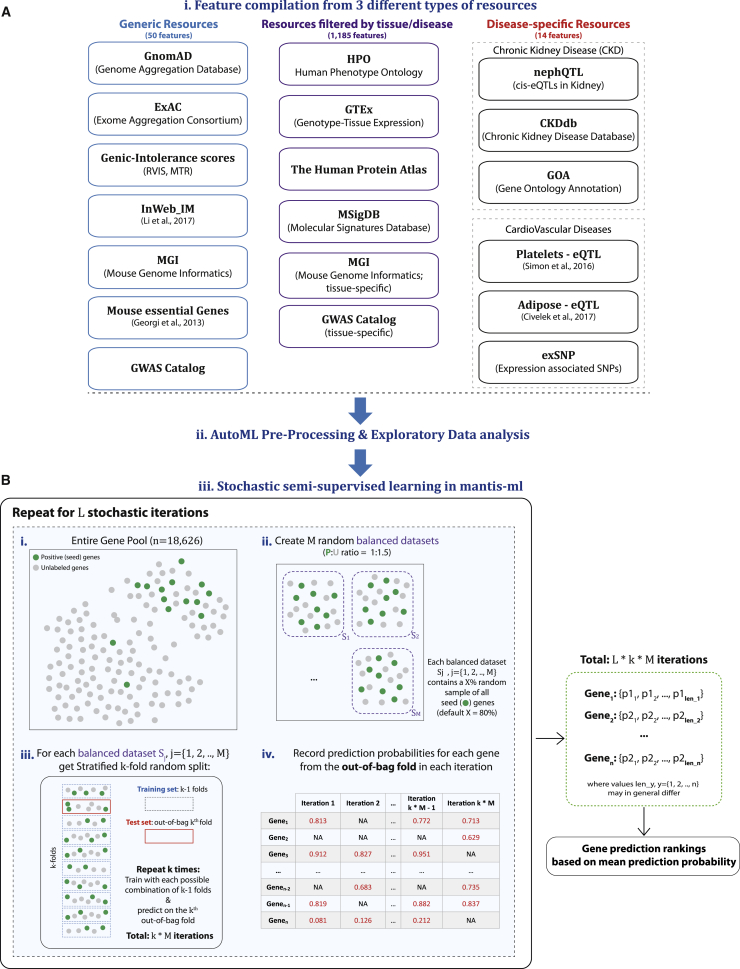
Generic Overview of Mantis-ml Workflow (A) Data resources used by mantis-ml for feature extraction. Three data-type resources are integrated: generic (i.e., non-tissue- and non-disease-specific), filtered by disease or tissue, and filtered by disease-specific features (currently including disease-specific features for CKD and cardiovascular disease). All features are compiled automatically on the basis of user-provided disease-associated query terms and pre-processed so they are ready to be provided as input to the supervised/un-supervised learning tasks. of mantis-ml. (B) Illustration of the stochastic semi-supervised approach followed by mantis-ml over *L* iterations: (1) positive (seed) genes are annotated using the HPO (static for each stochastic iteration), (2) the entire gene pool is split into random balanced sets, each of which includes a random sample (default: 80%) of seed genes, (3) each balanced dataset is split into a stratified *k* number of folds, training is performed for each combination of *k − 1* folds, and prediction is subsequently based on the *k*^*th*^ out-of-bag fold each time, and (4) prediction probabilities are aggregated for each gene across all *L* × *k* × *M* iterations.

Mantis-ml integrates gene-associated features from a diverse pool of gene-annotation sources, classified into three categories: generic resources (disease and/or tissue agnostic), resources filtered by tissue, and disease-specific features (Figure 1A and Table S1). mantis-ml performs automatic feature pre-processing, which includes filtering of highly correlated features on the basis of a Pearson’s r correlation threshold (parameter “eda_parameters -> high_corr_thres” in mantis_ml/conf/.config; default value: 0.8). Additionally, features with more than a certain amount of missing data are discarded (parameter “eda_parameters -> missing_data_thres” in mantis_ml/conf/.config; default value: 0.25). The remaining features with a missing-data ratio below the cut-off threshold are imputed with either a zero value or the median of the respective feature. Imputation with zero is performed either because most of the genes represent a binary flag (“non-existent” or 0 for missing data, e.g., “MGI_mouse_knockout_feature,” “GOA_Kidney_Research_Priority,” etc.) or because these features were extracted from computational or experimental studies that retrieve a biologically relevant signal only from a specific set of genes that are associated with the hypothesis under examination (e.g., “platelets_eQTL,” “adipose_GWAS_locus” features, etc.). The features that are imputed with a median value are all genome-wide association study (GWAS) metrics, ExAC CNV-associated features, the residual variation intolerance score (RVIS), the missense tolerance ratio (MTR), and gene length. The selection of the median value for imputation in that case is based on the use of different global reference sets of genes for different studies or resources. This difference requires extrapolation of these features to genes with missing values because penalizing them with a zero value would most likely not be representative of the actual gene behavior with respect to these features. Finally, features are standardized to have mean zero value and unit variance.

### Exploratory Data Analysis

Mantis-ml generates an extended set of visualizations, including heatmaps for pairwise feature correlations (prior and post feature filtering), missing-data ratios across features, and the distribution of numerical and/or categorical variables across the known and unlabeled genes from the entire gene pool (Figures S2 and S3). Additionally, mantis-ml automatically performs dimensionality reduction on the original feature set via principal component analysis (PCA), t-distributed stochastic neighboring embedding^16^ (t-SNE), and uniform manifold approximation and projection^17^ (UMAP) to allow for visualization of the original high-dimensional space in two dimensions (Figure S4).

Unlike PCA, t-SNE and UMAP are both non-linear projections of a high-dimensional space into a lower-dimensional space. They both compute probability distributions regarding the relationships between the points in the high-dimensional space but use different similarity kernels: Gaussian for t-SNE and non-Gaussian for UMAP. The computed similarities are then recreated in the lower-dimensional space (embedding space) with a Student’s t distribution for t-SNE and a kernel proximal to t distribution for UMAP. The main practical difference between these algorithms is that t-SNE can capture local relationships well in the embedding space but does not preserve the global structure, whereas UMAP can preserve both the local and global structure in the lower dimensional space.

PCA is accompanied by a Scree plot summarizing the cumulative variance explained by the first 20 principal components. Because PCA can only capture linear relationships between the original vectors, the variance explained by the top principal components provides an insight into the prevalence of linear versus non-linear relationships between features in the original space. t-SNE is calculated with a default perplexity value of 30, whereas UMAP is run with the following default parameter values: “n_neighbors” = 5, “min_dist” = 0.3, “metric” = “correlation”. Finally, all two-dimensional projected feature visualizations are provided both as static files (PDF format) and interactive visualizations (HTML format).

These visualizations aim to highlight any evident and/or trivial segregation of the known disease-associated genes from the unlabeled genes on the basis of either pairwise relationships between the features or any linear and/or non-linear relationships captured by the top projected vectors of the respective transformation spaces. However, the complexity underlying each of the studied diseases imposes the exploration of high-dimensional interactions between all features to elucidate the complex mechanisms that primarily drive pathogenicity in each case. We thus resort to machine-learning techniques to tackle this problem.

### Stochastic Positive-Unlabeled Prediction with Standard Classifier and Benchmarking

Mantis-ml seeks to uncover any feature patterns among a collection of known positive-labeled disease-associated genes to then prioritize novel genes that share a highly similar feature profile with the known disease-associated genes. This problem falls into the broader machine-learning area of positive-unlabeled learning, a semi-supervised learning technique where the only labeled data points available are positive.

This is important in this context because we often have insufficient information about which genes among the remainder of the genome are definitively not associated with that disease (i.e., true negatives). There are several approaches aiming to solve positive-unlabeled problems, the most popular of which are (a) to treat unlabeled data as negative and perform learning with a standard classifier,^18^ (b) to use bootstrap and bagging to iteratively train on random samples of positive and unlabeled data and make predictions on the basis of out-of-bag unlabeled data,^19^ and (c) to use two-step approaches in which the first step tries to identify a confident set of negative points among the unlabeled set and then continues learning with a standard classifier.^20^

Here, we developed a gene-prioritization framework that is based on a variation of two of the positive-unlabeled approaches suggested above (a and b): a stochastic semi-supervised learning technique that is performed across multiple random balanced datasets from the entire gene set (*L* iterations over random partitionings of the entire gene space) and makes iterative predictions on out-of-bag data.

The input data for mantis-ml are all coding genes, labeled on the basis of known or unknown annotation for a disease and accompanied by a large set of gene-level features extracted from public databases. We tested seven different classifiers to be used during positive-unlabeled learning for each balanced dataset of positive and unlabeled data points. These seven classifiers were random forest, extra trees (extremely randomized trees—a variation of random forest), gradient boosting, extreme gradient boosting (XGBoost), support vector classifier (SVC), deep neural networks (DNN), and a stacking (ensemble) classifier with four base classifiers (random forest, extra trees, gradient boosting, and SVC) followed by a DNN in the second layer.

**Figure 2:**
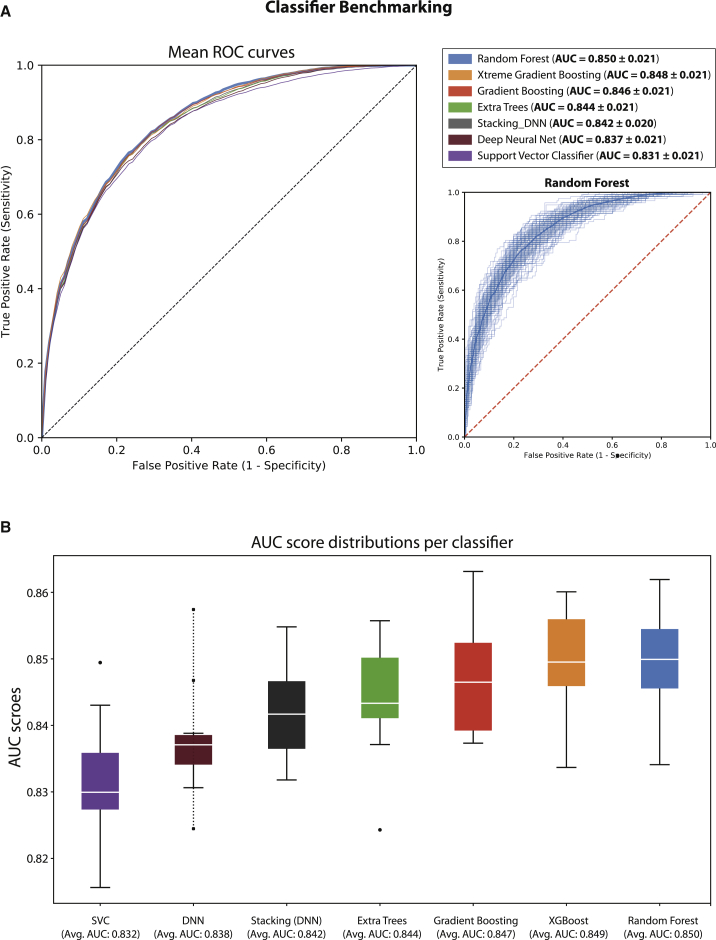
Mantis-ml Classification Performance Benchmarking Using Different Supervised Models Benchmarking of Seven Different Classifiers during the Positive-Unlabeled Learning Step of mantis-ml: Random Forest, Extra Trees, Gradient Boosting, Extreme Gradient Boosting (XGBoost), Support Vector Classifier (SVC), Deep Neural Network (DNN), and a Stacking (Ensemble) Classifier with Four Base Classifiers (Random Forest, Extra Trees, Gradient Boosting, and SVC) followed by a DNN Mantis-ml was run on ten random balanced datasets with 10-fold cross-validation based on the CKD example. (A) Mean receiver operating characteristic (ROC) curves from stochastic positive-unlabeled learning with one of the seven classifiers. ROC curves from all runs are also shown for the best performing classifier during benchmarking (random forest). (B) Distribution of AUC scores across the seven classifiers tested. All classifiers showed comparable performance (AUC: 0.83–0.85), and tree-based methods ranked on the top.

We first fine-tuned each classifier separately by using two random balanced datasets from the CKD disease example with 10-fold cross-validation and performing grid search over a finite parameter space. We then benchmarked all classifiers by assessing their “area under curve” (AUC) performance on the same set of ten random balanced datasets with 10-fold cross-validation. All classifiers performed comparably (average AUC: 0.831–0.850), and random forest and extreme gradient boosting ranked as the top two classifiers, with mean AUCs equal to 0.850 ± 0.021 and 0.848 ± 0.021, respectively (Figures 2 and S5). Given the comparable performance across classifiers, we do not pick a single classifier that outperforms the rest in the problem of gene prioritization with positive-unlabeled learning. Thus, we apply all classifiers to each disease example examined in this work and then select the best performing classifier in each disease example on the basis of the average AUC scores achieved.

Eventually, we have scaled up the positive-unlabeled learning task to the entire gene space, which is covered by a random partitioning of the unlabeled genes in combination with a random subset of the positive (seed) genes each time. We extract the final ranking by averaging the prediction probabilities assigned to each gene from all the generated out-of-bag sets. This approach allows genes to compete with each other in a stochastic semi-supervised manner and self-sort because their respective features can capture enough of the variance of truly disease-informative characteristics.

### Selection of Optimal Classifier Parameters via Grid-Search Cross-Validation

Fine-tuning for all “scikit-learn”-based classifiers (random forest, extra trees, gradient boosting, and SVC) and for XGBoost was performed over a pre-defined finite parameter grid space with GridSearchCV from “scikit-learn’s” “model_selection” module and tested on two random balanced datasets from the CKD disease example (Table 1). The available kernel options that were tested with SVC were “linear,” “poly,” “rbf,” and “sigmoid”.

**Table 1:** Optimal Parameters for Each Classifier Calculated with Grid Search and 10-fold Cross-Validation

**Parameters Selected with Grid Search**
**Deep neural network (DNN)**

hidden layers: 2
nodes per layer: [32, 32]
dropout ratio: 0.3
L2 regularization parameter: 0.01
optimizer: ‘Adagrad’
epochs: 50
batch_size: 128
activation function: “ReLU”

**Extreme gradient boosting (XGBoost)**

learning_rate: 0.01
n_estimators: 300
max_depth: 5
min_child_weight: 3
gamma: 0
subsample: 0.8
colsample_bytree: 0.8
objective: “binary:logistic”
scale_pos_weight: 1

**Extra trees**

n_estimators: 100
max_features: “auto”
max_depth: 15
min_samples_leaf: 2
min_samples_split: 5

**Gradient boosting**

n_estimators: 500
max_features: “sqrt”
max_depth: 20
min_samples_leaf: 4
min_samples_split: 5

**Random forest**

n_estimators: 100
max_features: “auto”
max_depth: 15
min_samples_leaf: 2
min_samples_split: 4
warm_start: false

**Support vector classifier (SVC)**

C: 0.01
kernel: “linear”
gamma: “auto”
probability: true
shrinking: true

With regards to *keras*-based DNNs (comprised of feed-forward fully connected layers), we developed a module that performs grid search with cross-validation (*dnn_grid_search_cv.py)*, including tuning of parameters such as size and number of hidden layers. This module currently supports simultaneous fine-tuning of up to two features but can otherwise fine-tune any DNN-related parameters in a single run with sequential steps of optimization that progressively select near-optimal features in a heuristic manner. “ReLU” has been used as the activation function across all hidden nodes, while “softmax” was used in the output layer. Additionally, seven different optimizers where tested as part of the grid search: “SGD,” “RMSprop,” “Adagrad,” “Adadelta,” “Adam,” “Adamax,” and “Nadam.” The optimal parameters returned by grid search with cross-validation for each classifier are available in Table 1.

### Selection of the Optimal Number of Stochastic Iterations in Positive-Unlabeled Learning

We examined the number of known and novel genes predicted for different numbers of stochastic iterations of positive-unlabeled learning by using an “extra trees” classifier on a disease-specific example (Figure S19). We observed that the number of predicted known genes is practically insensitive to the number of stochastic iterations. However, the number of novel genes requires a certain number of iterations until it reaches a stable state, which is around 1,000 genes. Specifically, the novel-gene count enters an oscillation zone around the stable state after *L* = 10 iterations, which is then further stabilized after *L* = 100 iterations.

In more rigorous testing, we assessed the correlation of mantis-ml average prediction probabilities when run for different number of stochastic iterations. Ideally, a robust algorithm should capture the same average profile for each gene irrespective of the number of iterations mantis-ml has been trained on. Specifically, we ran mantis-ml for the following numbers of stochastic iterations: 1, 10, 30, 50, 70, 100, 150, and 200. Pearson’s r correlation of the average mantis-ml prediction probabilities extracted from just one iteration compared to all other numbers of iterations is always >0.984 (p < 2.2 × 10^−308^). For any other pair of stochastic iterations with *L* > 1, Pearson’s correlations are in the range of 0.9976–0.999 (p < 2.2 × 10^−308^). These predictions demonstrate the robustness of mantis-ml predictions irrespective of the number of stochastic iterations used. Thus, we suggest using *L* = 10 iterations by default for a run on a disease-specific case. The user can further adjust this through the “*-i*” parameter when running the mantis-ml tool.

### Application of Mantis-ml on Disease Examples

We applied all seven classifiers used during benchmarking across *L* = 10 stochastic iterations for each disease-specific positive-unlabeled learning task. No selection of positive-labeled genes, tissue, and disease-relevant features requires human curation beyond provision of the user-defined disease-associated inclusion and exclusion terms in the input config file. The total number of training-test tasks performed across an equivalent number of random balanced gene samples with cross-validation was 25,000, 17,000, and 79,500 for CKD, epilepsy, and ALS, respectively. These sizes are inversely proportional to the number of seed genes in each case, and this number directly affects the size of constructed balanced datasets across the entire gene pool.

All classifiers except for stacking showed comparable performance when applied to the entire gene set for each disease case. The stacking classifier consistently demonstrated slightly lower performance than the rest of the classifiers (on average 0.05 lower AUC score). This is somewhat expected because one of stacking’s most notable properties is to smooth out predictions from its base classifiers. This means that predictions supported by most of its base classifiers are more likely to survive in the end, thus potentially lowering the total number of correctly identified genes, which might however be more robust than the predictions from each individual classifier as a result of its conservative nature.

### Dictionary of Inclusion and Exclusion Query Terms for the Studied Disease Examples

Mantis-ml requires as input a YAML ain’t markup language (YAML) config file containing information about the diseases/phenotypes of interest. One field in the config file is required (“disease/phenotype terms”), whereas another two fields are optional (“additional associated terms” and “diseases/phenotypes to exclude”). The descriptions for these fields are as follows:

• “disease/phenotype terms”- terms that characterize a phenotype or disease of interest and that are used for known disease-associated gene selection and filtering of relevant features (free text), required
• “additional associated terms”- terms used along with “disease/phenotype” terms to extract additional disease- or phenotype-associated features (free text), optional
• “diseases/phenotypes to exclude”- terms to exclude from disease or phenotype characterization and feature selection (free text), optional

The terms provided in these fields for each of the diseases under study (CKD, epilepsy, and ALS) are available in Table 2.

**Table 2:** Query Terms per Disease Category Provided in the Configuration File for Mantis-ml (*config.yaml*)

	**Disease or Phenotype terms**	**Additional associated terms**	**Diseases or Phenotypes to exclude**
Chronic kidney disease (CKD)	renal, kidney, nephro, glomerul, distal tubule	-	adrenal
Epilepsy	epilep, seizure	brain, nerve, nervous, neuronal, cerebellum, cerebral, hippocampus, hypothalamus	-
Amyotrophic lateral sclerosis (ALS)	amyotrophic lateral sclerosis, degeneration of the lateral corticospinal tracts, dysfunction of lateral corticospinal tracts, atrophy of the spinal cord, progressive distal muscular atrophy, spinal muscular atrophy, first dorsal interossei muscle atrophy, cervical spinal cord atrophy, corticospinal tract atrophy, corticospinal tract hypoplasia, atrophy/degeneration involving the spinal cord	brain, muscle - skeletal, nerve, nervous, neuronal, spine, spinal, cerebellum, cerebral, hippocampus, hypothalamus, muscular dystrophy, muscular fitness, muscle function, muscle, neuromuscular	heart muscle, heart_muscle, cardiac_muscle, smooth_muscle, cardiac muscle, smooth muscle, striated_muscle, striated muscle

All query terms are case insensitive and follow regular rules of wild card pattern matching. Cells with a dash should be left empty in the config.yaml file.

### Estimating Feature Importance with the Boruta Algorithm

The Boruta algorithm was run on top of a “random forest” classifier trained on 100 random balanced datasets with 10-fold cross-validation and was run internally for 100 iterations. Boruta assesses the importance of each feature by comparing its contribution with the ones from random permuted features and eventually provides Z scores that quantify the distance from these comparisons. Upon each Boruta training cycle on a random balanced dataset, features are characterized as “confirmed,” “tentative,” or “rejected,” and the full distribution of Z scores is provided for each of them. Because of the stochastic nature of the positive-unlabeled learning implemented by mantis-ml, features can be characterized by different labels in different runs of the Boruta algorithm. We have thus defined a decision threshold to classify each feature as “confirmed,” “tentative,” or “rejected” on the basis of its extracted labels across all Boruta runs. Specifically, for CKD and epilepsy, features are eventually classified as “confirmed” when they receive this label across at least 90% of all Boruta runs. The decision threshold for the “confirmed” feature classification has been set to 60% for ALS to compensate for the higher variance of extracted feature importance labels across all iterations; this higher variance is most likely due to the smaller set of seed genes in that case. In all three cases, features labeled as “rejected” are eventually classified as such in at least 90% of the cases, whereas the remaining features are characterized as “tentative” on the consensus labeling.

We also added an option for mantis-ml to be trained with only the set of “confirmed” features extracted by the Boruta algorithm (parameter “supervised_filters -> feature_selection: boruta” in mantis_ml/conf/.*config*). We then tested mantis-ml’s performance with each of the standard classifiers when we used different configurations of features: all features that survived after the feature pre-processing step or only Boruta-confirmed features (Figure S18). Training and prediction were performed with a set of 15 random balanced datasets from the CKD disease example. Mantis-ml performed slightly but non-significantly better when using the entire feature set (average AUC, 0.817 versus 0.815; two sample t test, p = 0.515). However, since Boruta does not need to be run by default as part of the mantis-ml workflow, the default configuration retains the entire processed feature space and allows the user to further explore by explicitly specifying “boruta” as the feature selection algorithm in the “supervised_filters” field in mantis_ml/conf/.*config.* We also tested the performance of the “stacking” classifier when fed with only the base classifier predictions versus by employing the original feature space on top of the extracted base classifier predictions. The stacking classifier’s performance was considerably better when retaining the original feature space at the second layer of the ensemble training and prediction, and this is the default configuration used by mantis-ml*.*

### Enrichment of Top Mantis-ml Predictions among Different Types of Qualifying Variants

We performed a stepwise hypergeometric test to assess the enrichment of high mantis-ml predictions (top 5% per disease) among different types of qualifying variants from the collapsing analyses (focusing on the collapsing analyses gene subsets with p value <0.05).

We observed that the enrichment of high mantis-ml predictions among top-ranked putative loss-of-function (pLoF)-associated genes is always statistically significantly different both from a shuffled (randomized pLoF-associated gene list) enrichment signal (Mann-Whitney U test p value = 2.91 × 10^−300^, 5.53 × 10^−147^, and 4.17 × 10^−194^ for CKD, epilepsy, and ALS, respectively) and the enrichment signal of genes associated with synonymous variants (Mann-Whitney U test p value = 1.34 × 10^−125^, 1.60 × 10^−33^, and 2.36 × 10^−84^ for CKD, epilepsy, and ALS, respectively). For the enrichment analysis of synonymous variants, we have considered “Dom_coding” (dominant coding) as the comparator class in ALS because of the lack of a real synonymous-based collapsing-analysis gene list in the published analysis.

We sought to explore how each of the seven classifiers performed when overlapped with top-ranked whole-exome sequencing (WES)-based gene lists. We observed that with regard to AUC, the best-performing classifiers per disease category in mantis-ml also ranked among the top three classifiers in terms of area-under-curve ratios and/or total pLoF area (Figures S10–S12). Other classifiers also performed comparably or slightly better, which is in concordance with the similar AUC performance achieved from differing classifiers retrieved from the original mantis-ml training, again reinforcing the consistency and robustness of the framework irrespective of chosen classifier.

### Visualization of Cross-Validated Mantis-ml Predictions and Downstream Analysis

After the cross-validation of mantis-ml predictions with rare-variant collapsing-analysis studies, we apply dimensionality reduction on the original feature space to highlight the novel predicted genes-of-highest interest in the entire exome space. PCA performed for each of the three disease examples used in this study achieves a slight segregation of positive and unlabeled genes; the consensus novel and known gene predictions tend to differentiate the most from the rest of genes (Figure S14). However, PCA fails to capture a high ratio of the total variance to be explained by its first two or three components (the variance explained by the first three components in each disease case is on average about 21%). Because PCA is representing the original features as linear combinations of the projected principal components, its inability to identify patterns of high variability in the entire gene set implies that the associations between the various collections of features driving gene predisposition to disease are probably non-linear.

Thus, we then apply two popular dimensionality-reduction techniques that can identify non-linear patterns in the original high-dimensional feature space for each disease example: t-SNE and UMAP. We observe that both methods map most of the consensus novel genes-of-highest-interest in the neighborhood of distinct clusters of known genes (Figure S15). Both techniques capture patterns in more localized regions of genes, although UMAP might also retain elements from the global structure more efficiently than t-SNE. By contrasting the two projections, we can identify clusters of genes that are more likely to be close (i.e., similar) to each other in absolute terms of distance (similarity). Finally, the mantis-ml tool provides interactive visualizations of all three projections (PCA, t-SNE, and UMAP) for further inspection of gene clusters and offers all extracted two-dimensional representations of the original data space that allow for further extraction of clusters of genes (e.g., through the use of HDBSCAN for further downstream analysis).

### Benchmarking of Gene-Prioritization Tools

Benchmarking of gene-prioritization tools is usually impeded by the high variability of input data, target-prediction goals, and output results provided by each tool. For this test, we selected the current state-of-the-art tools that allow for as much direct comparison against mantis-ml as possible.

Phenolyzer runs similarly to mantis-ml, in that the user only needs to provide disease-associated terms in free text. We ran Phenolyzer with the following terms by disease:

• CKD- “chronic kidney disease, nephropathy, glomerulopathy, kidney, renal” (in accordance with the terms employed by mantis-ml- “renal, kidney, nephro, glomerul, distal tubule”)
• Epilepsy- epilepsy, seizure (in accordance with the terms employed by mantis-ml- “epilep, seizure”)
• ALS: “amyotrophic lateral sclerosis, degeneration of the lateral corticospinal tracts, dysfunction of lateral corticospinal tracts, atrophy of the spinal cord, progressive distal muscular atrophy, spinal muscular atrophy, first dorsal interossei muscle atrophy, cervical spinal cord atrophy, corticospinal tract atrophy, corticospinal tract hypoplasia, atrophy/degeneration involving the spinal cord” (exactly the same as with mantis-ml).

For all Phenolyzer runs, we selected the “disease only” option in the “phenotype interpret” parameter. Selecting the “phenotype interpretation” option (i.e., looking for both diseases and phenotypes) for this parameter did not give any enrichment against collapsing analysis predictions (data not shown). On the other hand, we trained mantis-ml by selecting any phenotype or disease term associated with the provided string input (i.e., not restricting it exclusively to disease-associated terms), yet it managed to capture probable pathogenic gene signals substantially better than Phenolyzer. All other parameters for Phenolyzer were used with their default value in the webserver application:

• *“*Gene selection/Region selection/Weight Adjust/Word Cloud*”*- no
• “Addon Seed Gene”- “DisGenet Disease Gene Mapping,” “Genetic Association Database”
• “Addon Gene Relations”- nothing selected
• “Addon Gene Scores”: “Gene Haploinsufficiency Score,” “Gene Intolerance Score”

When we tested Phenolyzer’s predictions against the collapsing analyses, we ensured that we were testing only for genes that are represented both in Phenolyzer’s output and in the collapsing results to avoid any unfair conclusions in the results.

With regard to ToppGene, for benchmarking on ALS we provided the same set of seed genes as with mantis-ml (77 genes) and provided the rest of the exome as the test set. Attempting to run ToppGene for CKD and epilepsy by providing the respective mantis-ml-generated sets of seed genes (587 and 864 genes, respectively) and providing the rest of the exome as the test set was not possible because the webserver was crashing while trying to read and pre-process the original training and test-set input. We thus employed as our test set a smaller set of genes that was amenable to processing by the ToppGene webserver. In order to compensate for the inability of ToppGene to look into the entire exome, we provided the top 7,000 genes predicted by the collapsing analyses on CKD and epilepsy as our test set, thus informing it with approximately the top one-thirdof the exome, which is more likely to be associated with the respective disease.

The training and test sets employed by ToppGene for each disease were also used for ToppNet. In addition to that, we selected the default graph-prioritization parameters for each disease example:

• Prioritization method: k-step Markov
• Step size: 6

All data and scripts used for benchmarking are available at the GitHub repository under “mantis-ml-release/misc/overlap-collapsing-analyses” and are separated into folders for each benchmarked tool.

### Concordance of Classifier Predictions for the Generic Mantis-ml Score

The generic mantis-ml score (GMS) was trained on the basis of all OMIM disease-associated genes via six different classifiers: random forest, extra trees, gradient boosting, XGBoost, SVC, and DNN. Here, we have excluded the stacking classifier because it has a much longer training time than the other classifiers and, as a result, has a slightly lower AUC performance. Results retrieved by all classifiers were highly concordant. Specifically, of the 4,041 known genes, around 1,300 (32.2%) were consistently identified by all classifiers (with probability >0.5). Another 380 known genes (9.4%) were further identified by at least five of the six classifiers. With regard to novel disease-associated genes, again the largest group of predicted genes (n = 600) was predicted by all classifiers, and ~320 novels genes were predicted by at least five classifiers. In both cases, DNN had the highest number of known and novel predictions identified solely by a single classifier (280 and 680 predicted genes, respectively).

### Mantis-ml Package Structure

We built our mantis-ml framework by using Python on top of the *sckit-learn* and *keras* libraries. We have also employed the “Boruta*”* R package for feature selection based on the Boruta algorithm. The main components of mantis-ml are the “pre_processing,” “unsupervised_learn,” “supervised_learn,” “post_processing,” and “validation” modules. The “pre_processing” module implements the functionality for compilation of the input feature table, which contains three classes of features: generic features (tissue and/or disease-agnostic), features filtered by tissue and disease-specific features. Compilation of tissue and/or disease-specific features is performed with a curated dictionary of relevant query terms. After data compilation, the “pre_processing” module implements the rest of its main functionality around feature pre-processing, exploratory data analysis, and visualization of features distribution. The “unsupervised_learn” module performs dimensionality reduction on the processed feature set for visualization purposes and extraction of two-dimensional representations of the data for downstream analysis, such as clustering and pathway-enrichment analysis. Processed feature tables are then passed on to the “supervised_learn” module for feature selection with Boruta and the stochastic positive-unlabeled learning task, which is the core of the mantis-ml workflow. Prediction probabilities extracted from this step are fed to the “post_processing” module for aggregation of results and optional overlap with third-party studies (e.g., rare-variant cohort studies or any independently generated ranked gene list) through use of the “validation” module.

### External Libraries Used for Implementations of Supervised-Learning Models

For random forest, extra trees, gradient boosting, and SVC, we used their implementations from scikit-learn (v0.20.3), whereas for DNNs we used Keras (v2.2.4) with Tensorflow (v1.10.0) in the backend. XGBoost’s implementation was provided by the xgboost Python package (v0.80).

### Computational Requirements and Time Complexity

All three benchmarked disease examples have been run on a simple linux utility for resource management (SLURM) cluster with 10 CPU cores (Intel(R) Xeon(R) CPU E5-2683 v. 4 at 2.10 GHz) for ten stochastic iterations. The total time required for each study was inversely proportional to the respective number of seed genes because this directly influences the total number of random balanced datasets that need to be trained with k-fold cross-validation. Specifically, total execution time across all classifiers was 1 h 55 min, 2 h 33 min, and 11 h 23 min for epilepsy, CKD, and ALS, respectively (for 864, 587, and 77 seed genes, respectively).

## Results

### Mantis-ml Overview

We developed mantis-ml as an automated machine-learning (AutoML) framework to enable learning from an arbitrary set of gene-associated features (Figure S1). We collated data from a diverse set of gene-annotation sources (Figure 1A and Table S1) classified into three categories: generic resources (disease and/or tissue agnostic), resources filtered by tissue, and finally, disease-specific features. Given a set of user-specified query terms relating to a tissue and/or disease of interest, the data compilation and cleaning is performed automatically (see Methods). Currently, over 1,200 gene-annotation features are integrated in our framework. Additionally, mantis-ml automatically generates a rich set of visualizations for exploratory analysis on the original feature space (see Methods).

### Stochastic Semi-supervised Learning for Gene Prioritization

Positively labeled genes for the gene prioritization are retrieved by mantis-ml from the Human Phenotype Ontology^5^ (HPO) on the basis of user-provided inclusion and exclusion query terms that are relevant to a particular disease (see Methods). The HPO component then identifies seed genes on the basis of the documentation of gene-disease association in OMIM and additional clinical terminology curated by clinicians participating in regular workshops hosted by the HPO team. An important peculiarity for this problem derives from the fact that in most diseases, the overall set of known disease-associated genes (positive labels) is typically in the range of approximately tens to hundreds of genes. This makes the entire protein-coding gene set (n = 18,626) highly imbalanced in terms of the overall population of positive and unlabeled data points. Additionally, the entire gene space is finite and practically already known, so we are not bound by the usual machine-learning requirement to train a model that can generalize well on unseen data. Our end goal is to rank the entire gene set with respect to a diverse pool of disease groups and ensure that our predictions are not biased by a single training dataset of a subset of genes assumed to be representative of the global distribution of all genes. As a result, and in combination with the lack of a well-defined negative set, training a sufficiently generalizable model to then make predictions on the basis of a test set would not be ideal in this context.

To address this issue, we constructed a gene-prioritization framework that is based on a positive-unlabeled approach: a stochastic semi-supervised learning technique (*L* iterations) across multiple random balanced datasets from the entire gene set (Figures 1B and S1B) with iterative predictions on out-of-bag data. In each stochastic iteration, we create a random partitioning of the unlabeled gene space to form *M* balanced datasets, with a positive-to-unlabeled points ratio equal to 1:1.5. Each balanced dataset contains a random *X* percentage sample of the positive (seed) genes (default *X* = 80%) to reduce bias induced by use of the entire positive gene pool in each training task. The unlabeled data are treated as negative because training on positive and unlabeled data in general gives scores proportional to the ones retrieved by training on positive and negative data.^18^ We then perform a stratified *k*-fold split on each balanced dataset (default *k* = 10) and train with a standard classifier for each possible combination of *k* − 1 folds (training set) followed by prediction each time on the out-of-bag *k*^*th*^ fold (test set). This process is performed *k* times over each balanced dataset, and upon each training cycle, prediction probabilities are retrieved only for the genes belonging to the respective test set (out-of-bag *k*^*th*^ fold). We tested seven different classifiers to be used during positive-unlabeled learning for each balanced dataset (see Methods). All classifiers performed comparably (average AUC, 0.831–0.850), implying that mantis-ml is not sensitive to the underlying machine-learning method but is rather enabled by the informativeness of the integrated data itself.

This process creates, from the entire gene space, multiple smaller gene poolsthat have comparable numbers of known and unlabeled genes and allow the classifier to capture strong patterns among the known genes to then rank all genes (both known and novel) with respect to a disease profile. Notably, this process can also identify mislabeled known genes and automatically readjust their rank given a sufficiently curated starting set of known disease-associated genes. The entire procedure is repeated for *L* iterations, each one leading to a random set of balanced sets to allow inclusion of each gene in out-of-bag sets multiple times and subsequently lead to less biased and more robust results. mantis-ml does not define a static underlying model but prioritizes all genes on the basis of the probability prediction they have achieved over multiple iterations that have grouped the genes into random balanced groups. Eventually, we aggregate the prediction probabilities assigned to each gene member from out-of-bag sets (either positive or unlabeled) across all *L* × *k* × *M* iterations. For each gene, this forms a probability distribution regarding the association of a gene with the disease under examination. The final gene predictions are ranked on the basis of the mean of their probability distributions (results equivalent with median, Pearson’s r > 0.9996, p < 2.2 × 10^−308^).

### Application on Three Disease Examples: ALS, CKD, and Epilepsy

We applied mantis-ml on three complex diseases: ALS, CKD and epilepsy (genetic generalized epilepsy). We selected these because studies involving hypothesis-free exome-wide association statistics have been previously published for these disease examples. The positively labeled gene set for each disease was selected on the basis of a user-defined curated dictionary of inclusion and exclusion terms used for automated querying and extraction from HPO (Table 2). Tissue-specific and disease-relevant features were automatically extracted on the basis of the same query terms applied to the mantis-ml integrated knowledgebase (see Methods). The total number of known (seed) positively labeled genes found for each disease on the basis of HPO was 587, 864, and 77 for CKD, epilepsy, and ALS, respectively.

We first ran a benchmarking test on ten random balanced datasets from the CKD example and obtained an average best AUC performance of 0.85 (Figure 2; see Methods); scores were comparable across all classifiers. We then applied all seven classifiers on each disease example Each yielded comparable performance (see Methods): average AUC scores were 0.846, 0.821, and 0.814 for CKD, epilepsy, and ALS, respectively. Specifically, XGBoost and random forest had the best performance in CKD (average AUC: 0.846); this was followed by gradient boosting and extra trees (average AUC: 0.843 and 0.839, respectively), and some of the most well-established CKD genes (*PKD1*, *PKD2*, *COL4A1*, *COL4A3*, *COL4A4,* and *COL4A5*) ranked in the top 0.2%–0.7% of all genes (Figure S6). An aggressive prediction-probability threshold of 0.5 was used for classifying genes as either predicted known or novel and assessing the concordance of results across classifiers. We observe high concordance between the predictions from all classifiers with regard to known and novel disease-associated genes (see Supplemental Methods). Having no further knowledge to validate mantis-ml predictions at this stage, we choose to consider the gene rankings of the classifier with the highest average and individual AUC scores as the default mantis-ml prioritization scheme for the respective disease.

**Figure 3:**
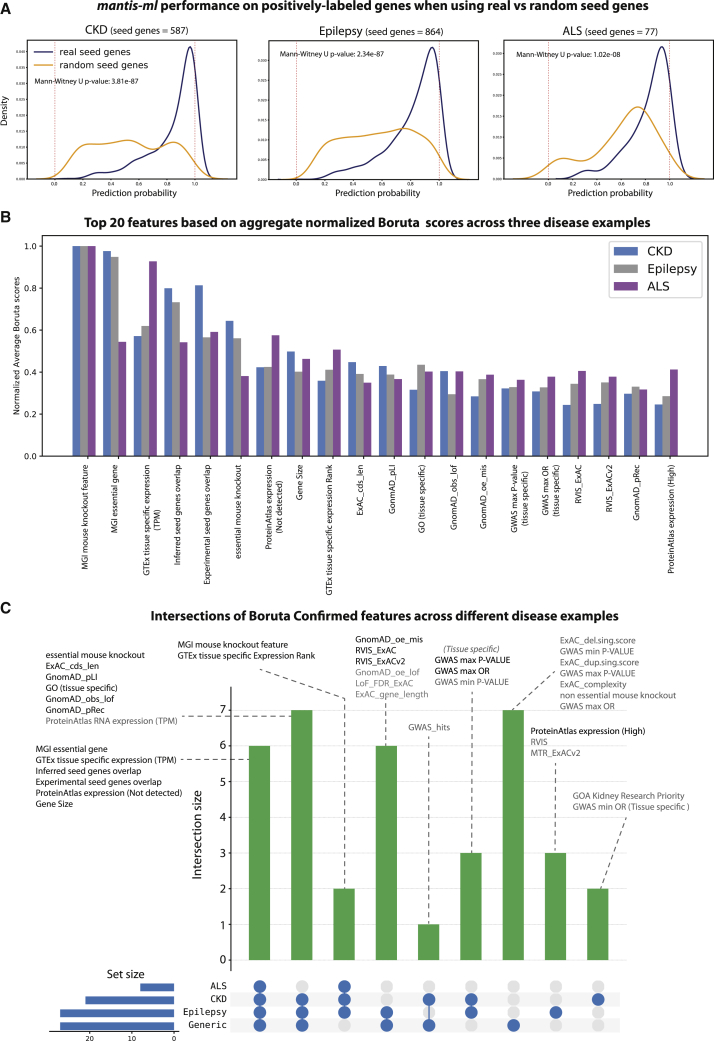
Mantis-ml Performance Sensitivity on Seed Genes and Consensus of Top Feature Contributors across Different Disease Examples as Determined with the Boruta Algorithm (A) Prediction probability distributions from positively labeled (seed) genes across the three disease examples when selected from HPO versus randomly assigned. Random seed genes are predicted with an almost uniform probability distribution, whereas real seed genes successfully get ranked to the top of the spectrum (probability values close to 1). (B) Top feature contributors per disease example are based on the sum of normalized average Z scores returned by Boruta. Features are ranked merely on the basis of their Z scores and are considered confirmed features according to this rank but without reference to whether they reach significance level. (C) Intersection of confirmed Boruta features across the three disease examples (CKD, epilepsy, and ALS) and the generic disease classifier. Features in black font correspond to the top 20 features as determined by the aggregate normalized Boruta scores.

We then sought to examine the importance of the seed-gene set for the prediction performance of mantis-ml. We thus tested the prediction probabilities across all seed (positively labeled) genes in the three disease examples (CKD, epilepsy, and ALS) when the seed-gene set is selected either randomly or on the basis of the HPO annotation (Figure 3A). The size of seed-gene sets varies in these disease examples (CKD: 587, epilepsy: 864, and ALS: 77), allowing exploration of the importance of seed-gene lists of varying length. We observed that in all three diseases, the prediction probabilities for seed genes when a real seed-gene set was used are skewed toward a probability of 1.0, whereas the respective distribution acquired when random seed genes of the same length were used is almost uniformly distributed across the entire probability spectrum (0.0–1.0). In all cases, the mantis-ml probability-score distributions obtained when real seed genes were used were significantly different from those obtained with random seed genes of matched gene-set size (Mann-Whitney U test p value = 3.81 × 10^−87^, 2.34 × 10^−87^, and 1.02 × 10^−08^ for CKD, epilepsy, and ALS, respectively).

### Mouse Model Phenotypes, Tissue Expression, Protein-Protein Interactions, and Intolerance Metrics Are Recurrently among the Top Features

Although mantis-ml focuses on identifying features highly predictive of known seed genes, we expect that some features are generally strong predictors of disease-associated genes. In total, mantis-ml integrates more than 1,200 features (Figure 1) that are automatically subset according to the disease under study. We sought to explore the contribution of each of the features during learning across all three examined disease examples. We adopted the Boruta^21^ algorithm based on a random forest classifier across 100 random balanced gene subsets with 10-fold cross-validation (see Methods). The Boruta algorithm provides an unbiased assessment of feature contribution because it constructs artifactual features (shadow features) from random permutations of each of the actual features of a dataset and then iteratively confirms or rejects the original features on the basis of their Z score distances from the importance levels achieved by the random (shadow) features. We ran Boruta for CKD, epilepsy, and ALS and extracted the consensus profile of feature importance in each disease case across ten stochastic iterations (Figures S9A–S9C; see Methods). We then normalized the Z scores among the three disease cases (min-max normalization) to compare the relative importance of each feature in the different diseases and provide a disease-agnostic consensus of the feature-importance profile (Figures 3B and 3C).

The consensus of feature selection across CKD, epilepsy, and ALS reveals mouse-model phenotypes and tissue-specific expression (in kidney, brain, and either brain or skeletal muscle, respectively, on the basis of ProteinAtlas and GTEx) as consistently highly important contributors. Specifically, the “MGI mouse knockout feature” is the top contributor in all three cases. This feature captures human genes with mouse orthologs that (according to Mouse Genome Informatics [MGI]) are associated with a “high-level mammalian phenotype” relevant to the disease under study (see Methods). Moreover, human orthologs of mouse genes that have been found to be essential for basic developmental functions and/or survival in mice (MGI essential gene) are the next top contributors in CKD and epilepsy and are among the top contributors for ALS. Tissue-specific expression [“GTEx tissue specific expression (TPM)”, “ProteinAtlas expression (not detected),” and “GTEx tissue specific expression rank”] follow in the order of consistent feature importance and makes a particularly high contribution to ALS compared to the other two disease cases. An interesting outcome of the Boruta algorithm is the emergence of protein-protein interactions-related features’ (“inferred seed genes overlap” and “experimental seed genes overlap”) being ranked in the top five recurrently important features. These represent bespoke constructed features to capture the ratio of known (seed) genes interacting directly with the index gene on the basis of either an “experimental” or “inferred” prediction from the “InWeb_IM” resource^8^ (see Supplemental Methods). Finally, tissue-specific gene ontology (GO) terms, intolerance scores based on ExAC and GnomAD (“GnomAD_pLI”, “GnomAD_obs_lof,” “GnomAD_oe_mis,” “GnomAD_pRec,” and “RVIS”), tissue-specific GWAS metrics (“GWAS max p value” and “GWAS max OR”), and gene size complete the picture of the most contributing features for classification of disease-associated genes.

### Application of Mantis-ml Predictions to Support Triaging of Exome-wide Cohort Association Studies

The rapid development of next-generation sequencing (NGS) technologies in recent years has led to the ubiquitous application of large-scale genomic studies by large genomic and/or healthcare institutions for research and diagnostic purposes. Of special interest are large association studies that assess the enrichment of rare predicted deleterious variants in a collection of disease-ascertained cases in comparison to an available control population. Depending on the contribution of individual genes to disease risk, these studies can provide experiment-wide significant results,^15^^,^^22^ but more often they yield many highly ranked genes of interest that do not exceed the multiplicity-adjusted genome-wide statistical-significance threshold. Thus, biologically relevant genes are expected to be residing among stochastic signals representing the natural tail of the null distribution. Teasing apart the biological signals from stochastic signals among the top ranks is a key challenge for most high-throughput genomics screens. Often downstream bioinformatic analysis involves laborious post-hoc review through existing literature, and this process can be biased by differing decisions influenced by an individual researcher’s prior experience or familiarity. Thus, the adoption of different resources by different researchers will ultimately result in triaging different genes.

Mantis-ml eliminates subjectivity and post-hoc design from gene prioritization by using a standardized set of community knowledge and collectively assessing all interactions (both linear and non-linear) between multiple features. To demonstrate the utility of leveraging the power of mantis-ml predictions to support triaging candidate genes found among results from WES-based association studies, we selected three disease studies whereby genes have been previously ranked on the basis of the significant case-enrichment of various types of qualifying variants, e.g., pLoF, missense, synonymous, etc. For CKD, we are cross-referencing a study examining the preponderance of rare pLoF and other types of rare variants across a population of 3,150 affected individuals and 9,563 controls.^14^ Another study of 640 individuals with familial genetic generalized epilepsy and 3,877 controls has been employed for further triaging on the basis of the mantis-ml predictions for the epilepsy disease case.^15^ Finally, for ALS, we are using the results from a study looking into the collapsing analysis results of nearly 3,000 individuals with ALS versus 6,405 controls.^13^

In each example, we ask the question of whether the lowest p values from the exome-wide association statistics (highest ranked from cohort studies) are significantly enriched for genes that achieved among the highest mantis-ml predictions for that corresponding disease. To this end, we apply a hypergeometric enrichment test asking whether the top 5% of mantis-ml predictions in each disease example are preferentially overlapping with the top signals (genes with p < 0.05) from the cohort-level association studies. To strengthen our experimental design with negative control components, we assess this enrichment across several differing classes of qualifying variants, including synonymous and permutation-based gene-ranks, which should represent the null, to contrast the more biologically interesting models, such as the pLoF and ultra-rare deleterious missense collapsing analyses.

**Figure 4:**
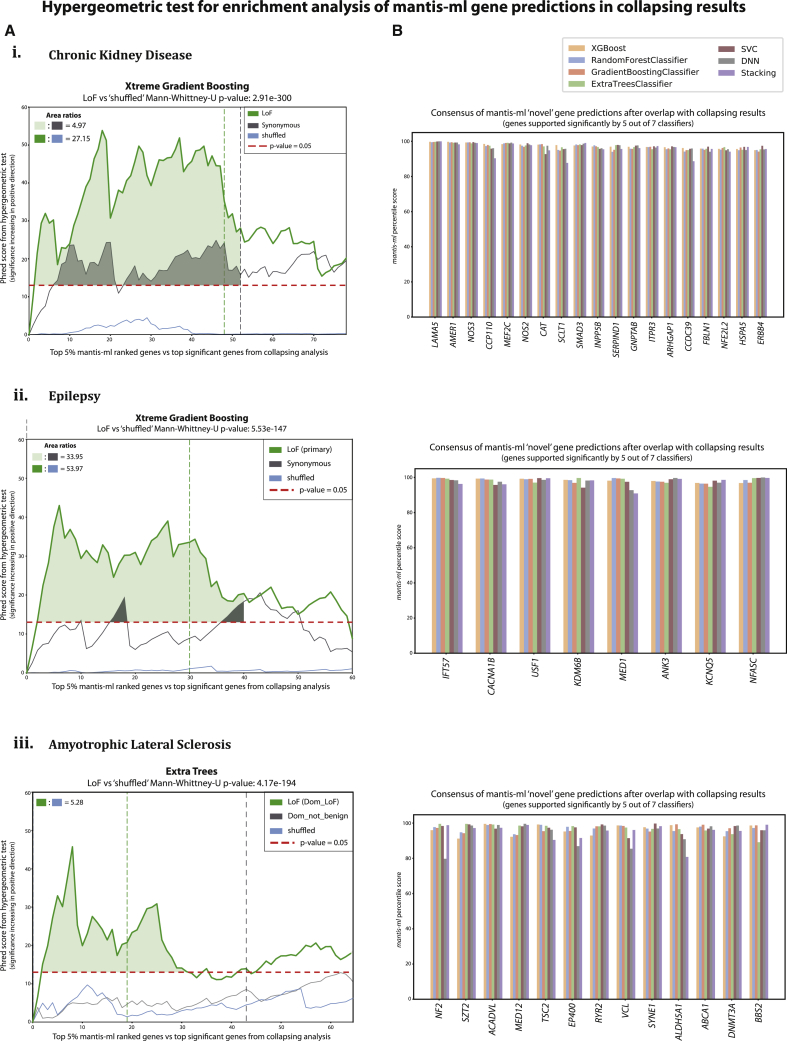
Cross-Validation of Mantis-ml Predictions (Wehn Both Known and Novel Genes Are Considered) with Cohort-Level Rare-Variant Association Studies (A) Hypergeometric test enrichment of disease-specific mantis-ml predictions with collapsing-analysis results from CKD (1), epilepsy (2), and ALS (3) cohorts. The horizontal dashed red line corresponds to the significance threshold of p = 0.05 for the hypergeometric tests. The places where the plot(s) go above this line highlight significant enrichment of mantis-ml-top-gene predictions among the population-genomic collapsing analyses. The vertical dashed lines, colored on the basis of the different classes of qualifying variants, indicate the last index of top-ranked genes from the collapsing analyses achieving a p value <0.05. The highlighted areas (light green and gray) represent the magnitude of enrichment signal for LoF and synonymous variants identified both from the collapsing analyses (p < 0.05) and the hypergeometric enrichment test against mantis-ml predictions (p < 0.05). (B) Consensus of genes of highest interest (novel) that satisfy the significance-threshold criteria in both the collapsing-analysis and the hypergeometric results and that are supported by five out of seven classifiers used by mantis-ml in the CKD (1), epilepsy (2), and ALS (3) disease examples.

We observed a strong enrichment in all three disease examples, and mantis-ml predictions overlapped significantly with the exome study ranking for pLoF variants (Figure 4A; see Methods). Notably, the significance of enrichment for pLoF signal within mantis-ml predictions is higher than for signals formed on the basis of other types of qualifying variant classes (synonymous, common, missense, and shuffled/random permutation; pLoF p value = 0.0004, 0.0005, and 0.009 for CKD, epilepsy, and ALS, respectively, on the basis of the hypergeometric test at the step where collapsing analysis reaches a p value of 0.05). Additionally, the enrichment of high mantis-ml predictions among top-ranked pLoF-associated genes is always statistically significantly different both from a shuffled enrichment signal (Mann-Whitney U test p value = 2.91 × 10^−300^, 5.53 × 10^−147^, and 4.17 × 10^−194^ for CKD, epilepsy, and ALS, respectively) and from the enrichment signal of genes associated with synonymous variants (Mann-Whitney U test p value = 1.34 × 10^−125^, 1.60 × 10^−33^, and 2.36 × 10^−84^ for CKD, epilepsy, and ALS, respectively). This evidently suggests the biological relevance of mantis-ml predictions with pathogenicity likelihood.

We further quantified the enrichment signal by calculating the ratios of areas under the curve between the pLoF and synonymous collapsing-analysis signals for CKD and epilepsy; for ALS, we used the total areas covered by the pLoF enrichment signal for ALS (because of the lack of a synonymous-associated signal in the respective published study; Figure 4A). The synonymous enrichment signal serves as a negative control (technical baseline) because we expect genes prioritized during collapsing analysis based on synonymous variants not to be associated with pathogenicity, in general.

We also performed the hypergeometric test to assess the enrichment of mantis-ml-predicted “known” or “novel” genes, separately, against the results of the collapsing analysis. We observe that mantis-ml predictions for “known” genes are significantly enriched for the top results of the collapsing analysis for all three disease examples (Figure S22A), indicating the biological relevance of the HPO-extracted seed genes (average p values in the significantly enriched region for ALS, 0.0085; CKD, 0.0034; epilepsy, 0.019). Similarly, mantis-ml predictions of “novel” genes are also significantly enriched for the top collapsing-analysis hits (Figure S22B) consistently across all three disease examples, proving that mantis-ml captures novel signals that have direct genetic evidence and, thus, are more likely to be biologically relevant for the disease of interest (average p values in the significantly enriched region for ALS, 0.013; CKD, 0.044 ; epilepsy, 0.031).

By applying the hypergeometric test for mantis-ml prediction enrichment in published case-control association studies, we can eventually extract a consensus list of predicted novel genes of highest interest that satisfy both the hypergeometric test (p < 0.05) and the collapsing-analysis statistical significance threshold (p < 0.05). Through the application of mantis-ml predictions onto published collapsing-analysis results, we are able to highlight 19 (CKD), eight (epilepsy), and 13 (ALS) novel (unlabeled) genes of highest interest. To validate this approach, we also assessed the results retrieved with regard to known (seed) genes for each disease. For example, in CKD we observe that some of the most well-established CKD-associated genes (*PKD1*, *PKD2*, *COL4A1*, *COL4A3*, *COL4A4*, and *COL4A5*) rank in the top nine genes among the 17 known CKD genes that achieved both a collapsing analysis p < 0.05 and hypergeometric test p < 0.05 (Figure S13).

### Downstream Review of Cross-Validated Mantis-ml and Collapsing-Analysis Predictions

The unlabeled genes of highest interest (novel mantis-ml predictions) represent a collection of genes that were not among the HPO-derived set of seed genes in the initial process of mantis-ml. We looked in the literature for references for the top-suggested novel genes per disease by mantis-ml and found supporting evidence for several genes. For instance, it has been reported in the last two years that LAMA5 variants are co-inherited with COL4A5 variants in familial hematuria,^23^ and such co-inheritance might affect pediatric nephrotic syndrome.^24^^,^^25^ Moreover, *NOS3* and *NOS2*—although not associated with CKD in OMIM—have been implicated in CKD in multiple studies,^26^, ^27^, ^28^, ^29^ whereas *MEF2C*, a gene typically associated with neurodevelopmental disorders, has also been associated with estimated glomerular filtration rate (eGFR) or proteinuria.^30^ *SCLT1* deficiency has been linked with cystic kidney disease,^31^ and *SMAD* genes (including SMAD3) have been reported to affect CKD progression when they are dysregulated.^32^ *INPP5B* impairment has been associated with severe renal phenotypes, such as proximal-tubule endocytosis,^33^ and targeting *NFE2L2* (NRF2) has been tested as a method of preventing kidney disease progression.^34^

With regard to the top novel epilepsy-associated predictions, *CACNA1B* is associated with the voltage-gated calcium channel that has been only recently implicated in epileptic phenotypes.^35^, ^36^, ^37^ *USF1* deficiency in mice (in combination with *USF2* knockout) has been shown to cause epileptic seizures,^38^ suggesting the important role this gene plays in normal brain function. Furthermore, *KDM6B* is associated with neuronal survival,^39^ and when haploinsufficient, it has been reported to cause severe seizures.^40^ *ANK3* has also been reported to be involved in epilepsy.^41^^,^^42^ Loss-of-function (LoF) and gain-of-function variants in *KCNQ5* have been shown to cause epileptic encephalopathy.^43^

As for the ALS consensus novel predictions, missense variants in *SYNE1* have been reported to be associated with a multisystemic neurological-phenotypic spectrum that includes ALS (see Web Resources).^44^^,^^45^ *ALDH5A1* is significantly downregulated in the spinal cord of an ALS murine model,^46^ whereas *ABCA1* is among the altered genes in the frontal cortex of ALS samples.^47^ Finally, motor neurons in human ALS show significant abnormalities in *DNMT3A*, which is also overexpressed in synapses of mice with motor-neuron degeneration.^48^

We also report sets of novel genes that have not been previously associated with the respective disease. Specifically, we have predicted the following sets of most likely novel disease-associated genes: *AMER1*, *CCP110*, *CAT*, *SERPIND1*, *GNPTAB*, *ITPR3*, *ARHGAP1*, *CCDC39*, *FBLN1*, *HSPA5,* and *ERBB4* for CKD; *IFT57*, *MED1*, and *NFASC* for epilepsy; and *NF2*, *SZT2*, *ACADVL*, *MED12*, *TSC2*, *EP400*, *RYR2*, *VCL*, and *BBS2* for ALS.

Finally, we employ three-dimensionality reduction methods (PCA, t-SNE, and UMAP) and provide static and interactive visualizations of the predicted known and novel genes over the transformed two-dimensional spaces retrieved with each technique for visual exploration and downstream analysis (see Methods).

### Benchmarking against Other Gene-Prioritization Methods

We then wanted to assess how mantis-ml predictions compare with previously published methods. Mantis-ml performs an exome-wide prioritization of genes without explicitly requiring a user-defined test set. Thus, we sought to employ methods that provide gene rankings across the whole exome or at least a large proportion of it so that they can be directly compared with mantis-ml. Eventually, we selected three methods for this benchmarking test: Phenolyzer,^49^ which is the current state-of-the-art method, and another two popular tools, ToppGene^50^ and ToppNet.^51^ With regards to selecting a ground truth set for our benchmarking, we wanted to use a completely independent dataset so that none of the benchmarked tools, including mantis-ml, had features informed by our truth set, thus inducing a circular feedback loop of prediction and, consequently, biasing the results. We therefore employ the results from rare-variant genetic association studies (collapsing analyses^12^) to assess the enrichment of highly ranked genes from each tool against the top ranked genes from each cohort study, which are highly enriched for loss-of-function variants in cases compared to controls.

**Figure 5:**
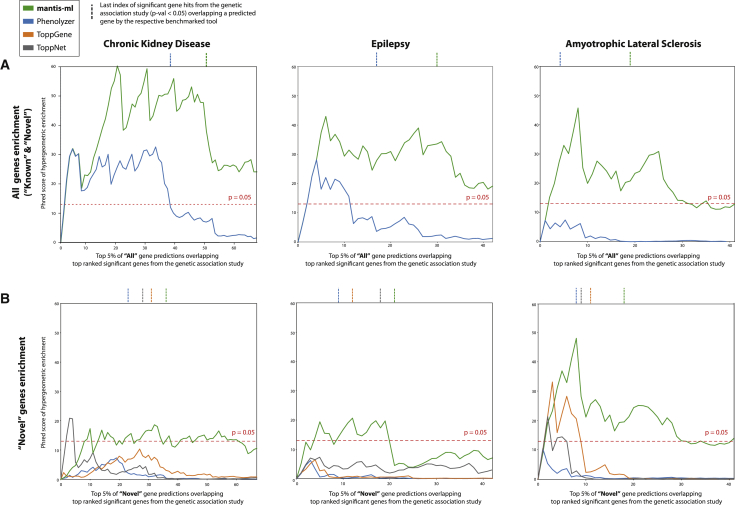
Benchmarking of Gene-Prioritization Tools Is Based on Enrichment of Their Top Predictions against Highly Ranked Genes from Rare-Variant Genetic-Association Studies across Three Disease Examples: CKD, Epilepsy, and ALS (A and B) The enrichment signal for each tool is derived from a hypergeometric test between the significant hits (p < 0.05) from the genetic-association studies (looking into LoF variant enrichment) and the top 5% of “known” and “novel” gene predictions (A) or “novel” gene predictions only (B), from each of the benchmarked tools. The horizontal dashed red line corresponds to the significance threshold of p = 0.05 for the hypergeometric tests. The vertical dashed lines above each plot, which were colored on the basis of the respective benchmarked tool, indicate the last index of top-ranked genes that achieved a p value <0.05 in the collapsing analysis and overlapped the top 5% of gene predictions from that tool.

Earlier, we demonstrated that mantis-ml’s predictions are highly enriched for the top predictions from collapsing analyses across three diseases for which data are publicly available (CKD, ALS, and epilepsy). We thus wanted to explore the degree of enrichment for the predictions from each external benchmarked tool when trained for the same diseases and phenotypes. We trained each tool by providing either the same disease-relevant terms or the same training set of genes as in mantis-ml, where applicable (see Methods). We ran the enrichment test against all gene predictions from mantis-ml and Phenolyzer because they provide prioritization scores for both “known” and “novel” genes (Figure 5A). ToppGene and ToppNet do not include prioritization results for the provided “known” (seed) genes; thus we also ran enrichment tests against only the novel “gene” predictions across all benchmarked tools (Figure 5B). We observe that mantis-ml enrichment among predictions from genetic association studies is substantially higher than enrichment for all other methods across all three disease examples (Figure 5). Phenolyzer achieves a significant enrichment for CKD and epilepsy when both known and novel genes are considered but does not reach the significance threshold for predictions of novel genes. ToppGene’s novel predictions are relatively enriched in ALS, and ToppNet’s novel predictions are borderline enriched in CKD and ALS. However, in all cases, enrichment derived from each of these tools is considerably lower than that derived from mantis-ml*,* and each of these methods overlaps with a much smaller set of genes that are significantly enriched in the respective collapsing analysis. Thus, mantis-ml provides the best-in-class prioritization for data extracted from whole-exome -sequencing studies and potentially from other high-throughput genomic studies as well (e.g., whole-genome sequencing and CRISPR screens).

### Generic Mantis-ml Score for Gene Disease Likelihood

One of the key requirements for the above three disease-specific applications of mantis-ml is a sufficient collection of known OMIM disease-associated genes based on HPO term linkage. A rich collection of genes will not always be available. Therefore, we wanted to further explore the generation of generic mantis-ml predictions. To achieve this, we trained mantis-ml by using all current OMIM disease-associated genes (4,041 in total; see Methods) as seed genes to create a GMS that can be used as a general estimate of gene-disease likelihood. Although it does not take full advantage of tissue- and disease-specific features, the GMS could be an opportunity to prioritize genes among disorders about which we currently have insufficient knowledge about disease-associated genes.

We calculated the OMIM-based GMS by using six different classifiers (see Methods). Gradient boosting was the top-performing classifier (average AUC = 0.84), followed by random forest, XGBoost, and extra trees with comparable AUC scores (Figure S16). Similar to the disease-specific cases, in the generic disease mantis-ml results, we observe a high concordance between the predictions from all classifiers with regard to known disease-associated genes among the out-of-bag test sets (see Methods). We provide the average probability scores returned by gradient boosting as the default ranking for GMS along with the respective percentile score for each gene (Table S5).

**Figure 6:**
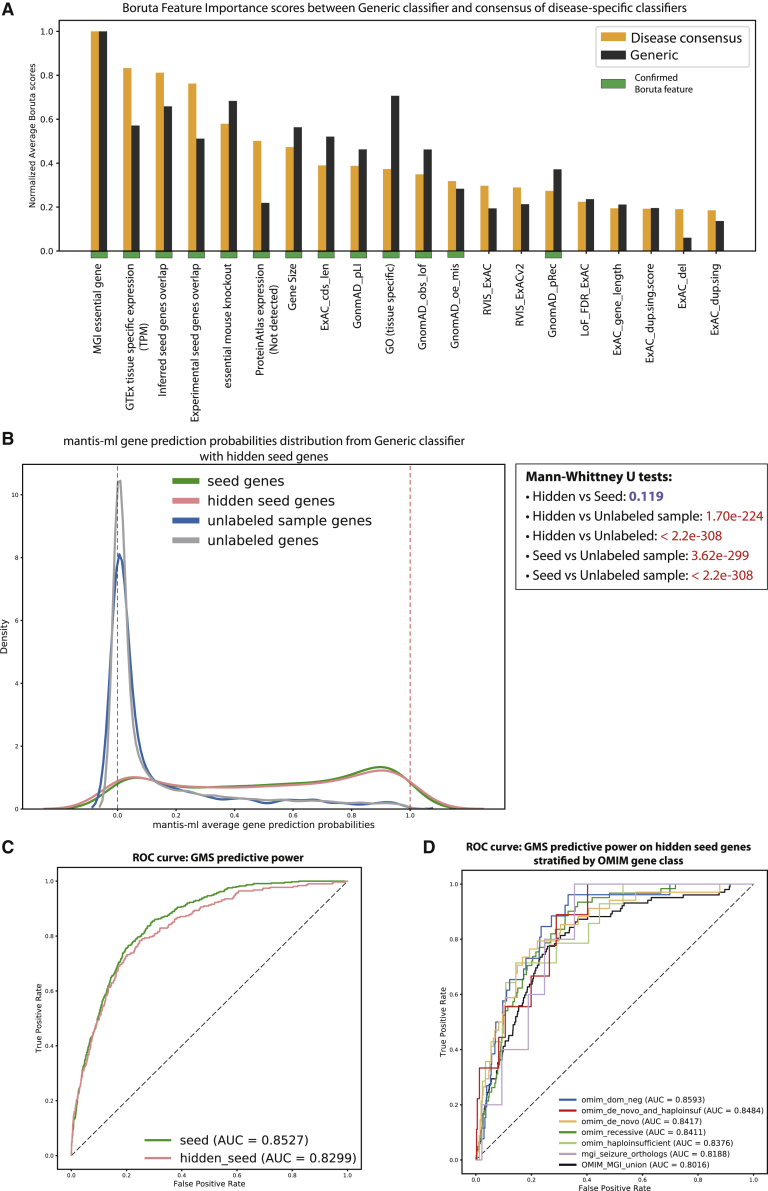
Generic Disease Mantis-ml Classifier for Estimation of Gene Disease Likelihood (A) Comparison of consensus top feature contributors from CKD, epilepsy, and ALS with GMS feature-importance scores. The consensus of the disease-specific case is calculated as the mean of the normalized average Z scores returned by Boruta for each disease case. (B) Generic mantis-ml prediction probabilities across different gene classes. The ranking was performed with 60% of the original seed genes set, and the other 40% of seed genes were treated as unlabeled. The unlabeled sample class represents a random sample from the unlabeled genes of equal size to the set of hidden genes. Mann-Whitney U tests were performed between the prediction-probability distributions of all pairs of gene classes (p values shown at the box on the right) to quantify their similarity degree. (C) Predictive power of GMS to distinguish seed genes (unmasked and hidden) from unlabeled genes via a logistic regression classifier. (D) Predictive power of GMS to distinguish different OMIM- and MGI-based hidden seed genes from unlabeled ones.

Furthermore, we re-ran the Boruta algorithm for a single (*L =* 1) stochastic iteration to identify the most important features that drive gene classification on the basis of the entire OMIM disease annotation and compared it against the respective features (where applicable) extracted from the disease-specific cases. We observe that mouse-model phenotypes (“MGI essential gene” and “essential mouse knockout”), protein expression (based on GTEx and Protein Atlas), protein-protein interaction features (“inferred seed genes overlap” and “experimental seed genes overlap”) are still the top feature contributors as in CKD, epilepsy, and ALS (Figure 6A). Additionally, gene length, gene ontology features, and intolerance scores (RVIS and the scores based on GnomAD) rank highly in the normalized average Boruta score scale.

To validate the generic disease classifier results, we explored the ability of mantis-ml to correctly identify a set of known genes that has not been provided as part of the original seed gene set. Thus, we masked a random selection of 40% of the 4,041 seed genes (considered unlabeled) and then trained the generic mantis-ml algorithm by using gradient boosting as the standard classifier for *L* = 5 stochastic iterations. Notably, there was no significant difference between predictions of masked OMIM disease-associated genes and unmasked seed genes (Mann-Whitney U test p value = 0.119; Figure 6B). We explored the predictive power of GMS in this case in terms of distinguishing seed genes from unlabeled ones and retrieved AUC scores of 0.853 and 0.83 for unmasked and masked (hidden) seed genes, respectively (Figure 6C).

To avoid any over-prediction bias from the unmasked seed genes, we assessed the ability of GMS to efficiently stratify different OMIM- and MGI-based gene classes on the basis of prediction probabilities assigned to hidden seed genes only. The gene classes that we used have been defined in a previous work,^9^ and the intersection with the hidden genes used in this case are as follows: OMIM dominant-negative genes (130), OMIM *de novo* and haploinsufficient genes (46), OMIM *de novo* genes (168), OMIM recessive genes (304), OMIM haploinsufficient genes (70), and MGI seizure orthologs (25). We also compiled the union of these gene classes as the OMIM_MGI_union set (510). We observed a high predictive power for GMS in this classification task; AUC scores ranged from 0.82–0.86 across the different OMIM or MGI gene sets and was 0.80 of the union of these genes (Figure 6D). These scores are considerably higher than those from similar assessments using other metrics of genic intolerance: gnomAD_pLI, gnomAD_mis_z, RVIS_ExACv2, ExAC_cnv.score, and LoF_FDR_ExAC. The AUC performance of these scores in the task of OMIM/MGI versus non-OMIM/MGI classification (Figure S21) were in the range of 0.58–0.85 (gnomAD_pLI), 0.47–0.79 (gnomAD_mis_z), 0.47–0.70 (ExAC_cnv.score), 0.50–0.68 (RVIS_ExACv2), and 0.50–0.72 (LoF_FDR_ExAC). The respective AUC scores for the classification of the union of OMIM/MGI gene sets versus non-OMIM/MGI genes were in the range of 0.48–0.60 across all these metrics. It is, however, important to note that these component metrics are based on more specific datatypes, whereas mantis-ml leverages the information from a wider and more diverse collection of features. The ability of mantis-ml to correctly classify hidden seed genes underlines its power to subsequently correctly identify other unlabeled genes and provide a biologically meaningful ranking.

Finally, we explored how GMS would perform when overlapped with disease-specific rare-variant collapsing-analyses results. This exploration was similar to our above assessment where we used the disease-specific mantis-ml predictions (Figure S17). We noticed that there is still significant enrichment of the LoF signal in all three disease examples, but it is at a lower level than in the disease-specific cases (GMS ratios of LoF to synonymous area, 1.18, 0.81, 2.21; respective scores with disease-specific classifiers, 4.97, 33.95, not defined, as a result of the lack of a synonymous class in the ALS disease example). This highlights the added value of leveraging the disease-specific features to efficiently identify genes associated with a disease when such information (seed genes) is available.

## Discussion

Presently, the genomics community is generating and analyzing large volumes of genomic data to better understand the genetic architecture of rare and common complex disorders. Here, we introduce a multi-dimensional machine-learning framework, mantis-ml, to support the triaging of the large-scale genome-wide readouts to further aid the prioritization of novel disease-associated genes. Mantis-ml takes its name from the Greek word *μάντης* which means “fortune teller.” Here, we have shown demonstrable predictive utility when combining mantis-ml with gene lists generated by large-scale association studies to enable a standardized and objective prioritization of genes for further functional validation in *in vitro* and *in vivo* models.

As with most machine-learning frameworks, one limitation of mantis-ml is its dependency on existing patterns. As such, mantis-ml is most powerful in identifying new disease-associated genes that might cause disease through an existing understood mechanism. Disease-associated genes representing an entirely unexpected disease mechanism might not be as highly prioritized. However, there might be opportunities to explore unsupervised approaches by clustering the results from t-SNE and UMAP (which are part of the mantis-ml processing workflow) to detect gene clusters that might not be well recognized. Moreover, the current mantis-ml package supports bespoke disease-specific features for CKD and cardiovascular disease (e.g., CKDdb, GOA, exSNP, etc.). These sets of features could be expanded for additional diseases via relevant data resources that might enable even more refined stratification of genes in other disease categories.

It will be interesting to explore how mantis-ml results validate against additional independent datasets that reflect gene rankings (e.g., CRISPR screens, GWAS etc.). For micro-array variant-level GWASs, one way to explore this would be to extract gene rankings on the basis of the lowest p value found for each gene. However, the mapping process is a challenge in this context because many of the variant signals from GWASs do not map to a single gene, especially in gene-dense regions of the genome, and thus, the confidence that a variant is mapped to the underlying biologically relevant gene can be low when the closest protein-coding gene is selected. Although this potential application on GWAS data is currently out of scope, we do provide the “mantisml-overlap” command line tool as part of the mantis-ml framework to enable users to validate the mantis-ml predictions against any other gene rankings (extracted e.g., from GWAS, CRISPR, or other high-throughput genetic screens).

Opportunities for future technical expansion of this approach include exploring integrating autoencoders for fully feature-agnostic dimensionality reduction as well as applying graph convolutional networks into a multiple kernel learning approach to better leverage information from multiple protein-protein interaction networks at the same time. Our framework could also extend to variant prioritization; however, this would require a revised design and a different collection of variant-level features.

We propose use of mantis-ml as an objective, standardized, fully quantitative and automated gene-prioritization tool for disease-specific or disease-agnostic studies. Additionally, we provide it as a complementary tool for the assessment of putative disease-associated genes extracted from completely orthogonal large-scale studies of human genetics thus reducing the required time for triaging top gene candidates from what is often a weeks to months-long process down to just a couple of hours.

## Declaration of Interests

The authors declare no competing interests. D.V. and S.P. report personal fees from AstraZeneca, during the conduct of the study. D.V.’s work was funded by the AstraZeneca post-doctorate program.

## Web Resources

The Mantis-ml Tool, https://github.com/astrazeneca-cgr-publications/mantis-ml-release
The Mantis-ml Gene Prioritization Atlas*,* https://dvitsios.github.io/mantis-ml-predictions

## Supplemental Information

Tables S1–S5

Document S2. Article plus Supplemental Information
