# Supplementary material for: Mantis-ml: Disease-Agnostic Gene Prioritization from High-Throughput Genomic Screens by Stochastic Semi-supervised Learning
Source: Am J Hum Genet. 2020 May 7;106(5):659–78. doi: 10.1016/j.ajhg.2020.03.012 (PMC7212270; doi:10.1016/j.ajhg.2020.03.012)
Supplement: Document S1 — Figures S1–S25 and Supplemental Methods [file mmc1.pdf]

The American Journal of Human Genetics, Volume *106*

Supplemental Data

Mantis-ml: Disease-Agnostic Gene Prioritization from High-Throughput Genomic Screens by Stochastic Semi-supervised Learning

Dimitrios Vitsios and Slavé Petrovski

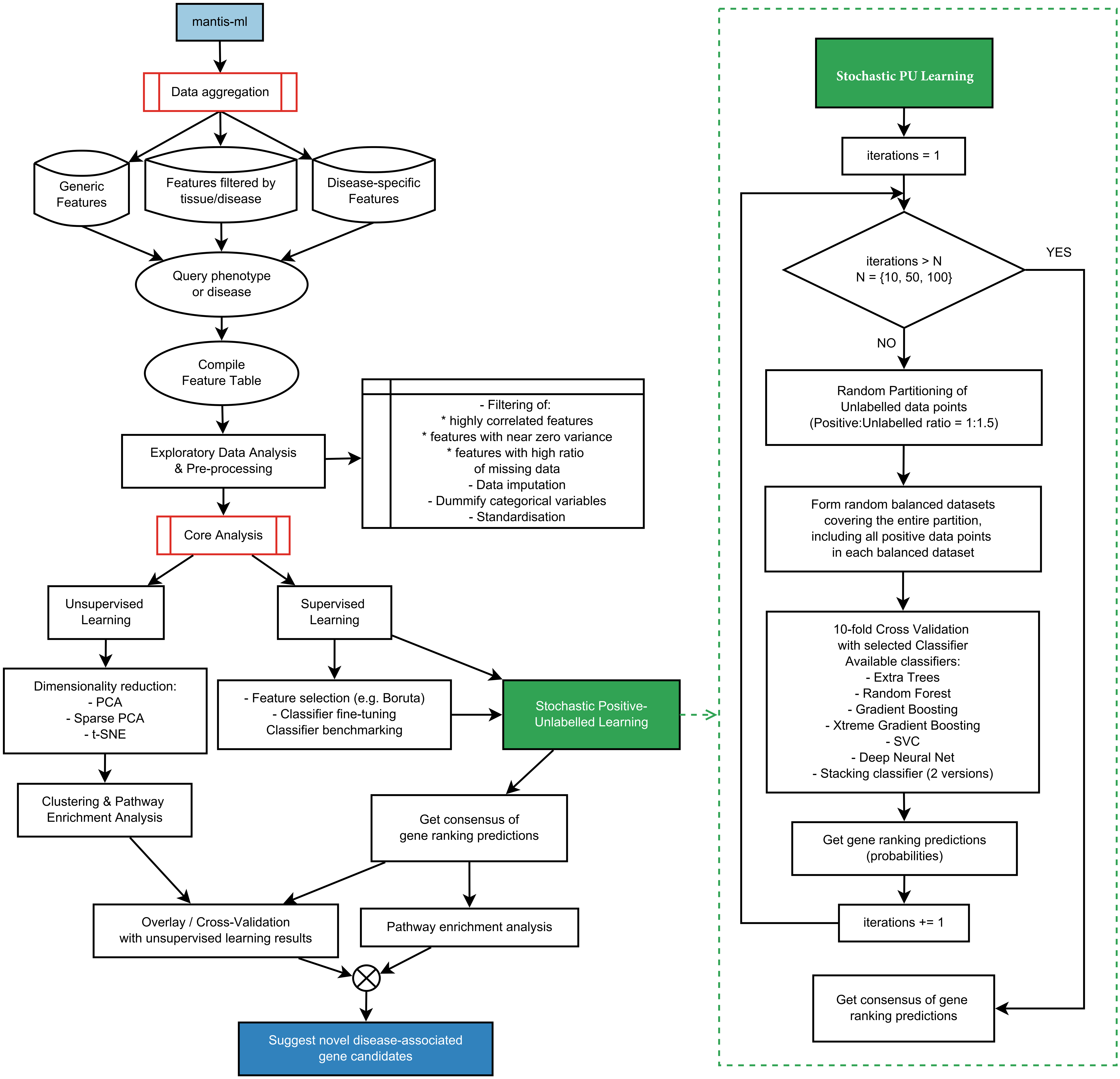

Figure S1. mantis-ml flowchart diagram visualizing the steps for data aggregation, pre-processing and the core analysis, including a detailed overview of the stochastic positive-unlabeled learning implementation.

mantis-ml pre-processing and exploratory analysis (CKD example)

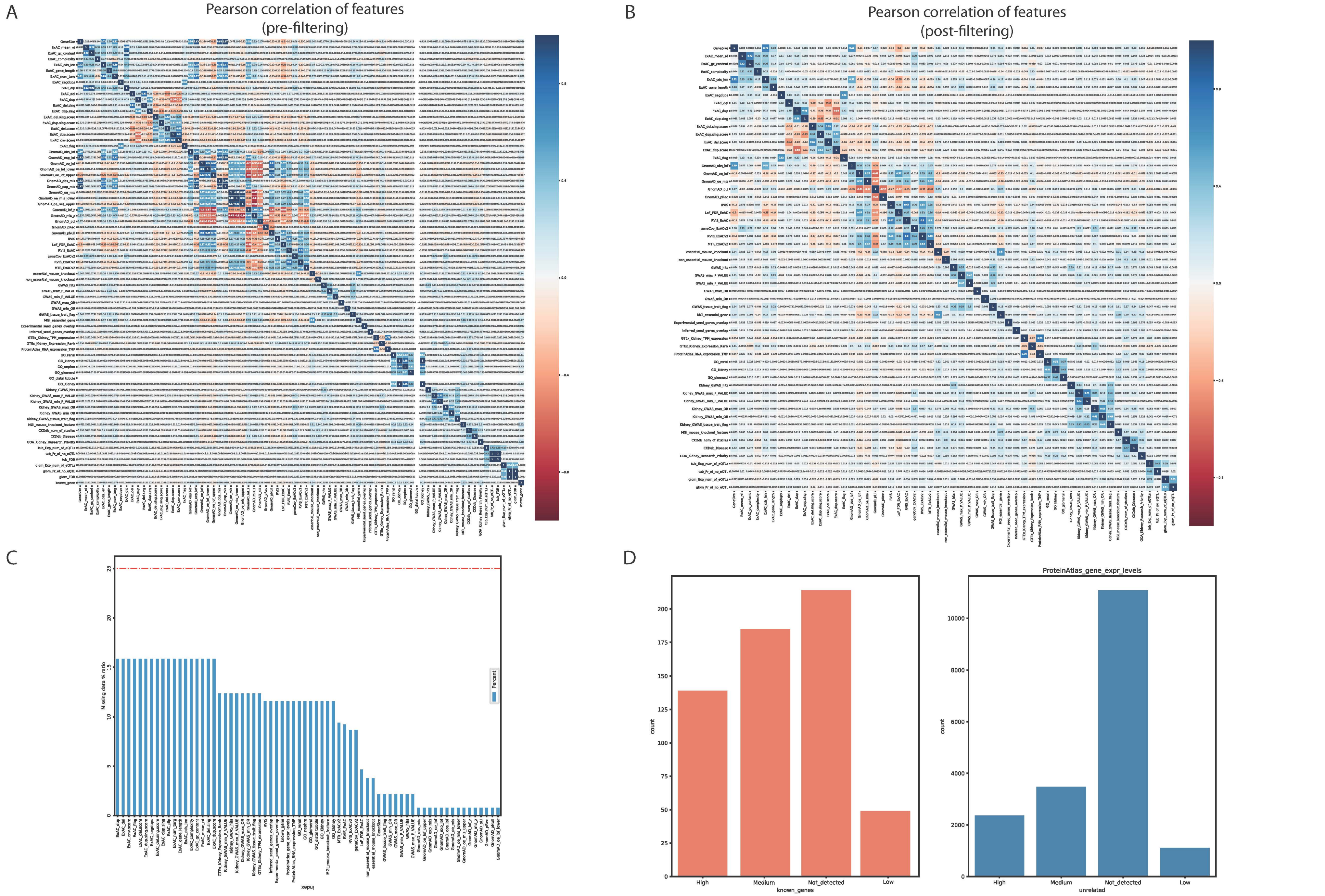

Figure S2. Data pre-processing and exploratory data analysis in Chronic Kidney Disease case: A) Pairwise feature correlation **before** filtering of highly-correlated features, B) Pairwise feature correlation **after** filtering of highly-correlated features, C) Missing data ratios and D) distribution of categorical feature profiles in positive and unlabelled genes.

Exploratory analysis - numerical features distribution

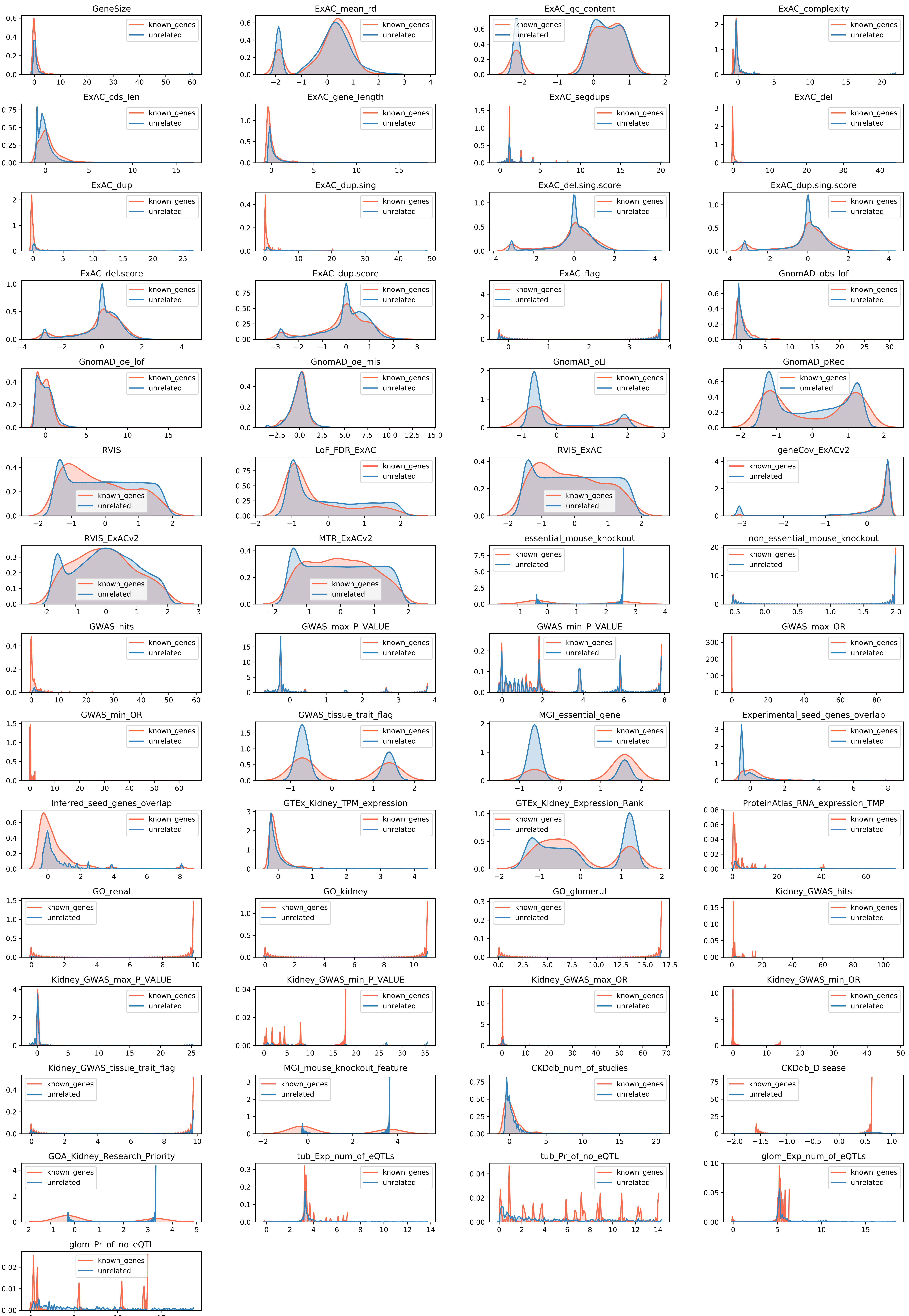

Figure S3. Exploratory data analysis in Chronic Kidney Disease case: distribution of numerical feature profiles in positive (known) and unlabelled genes.

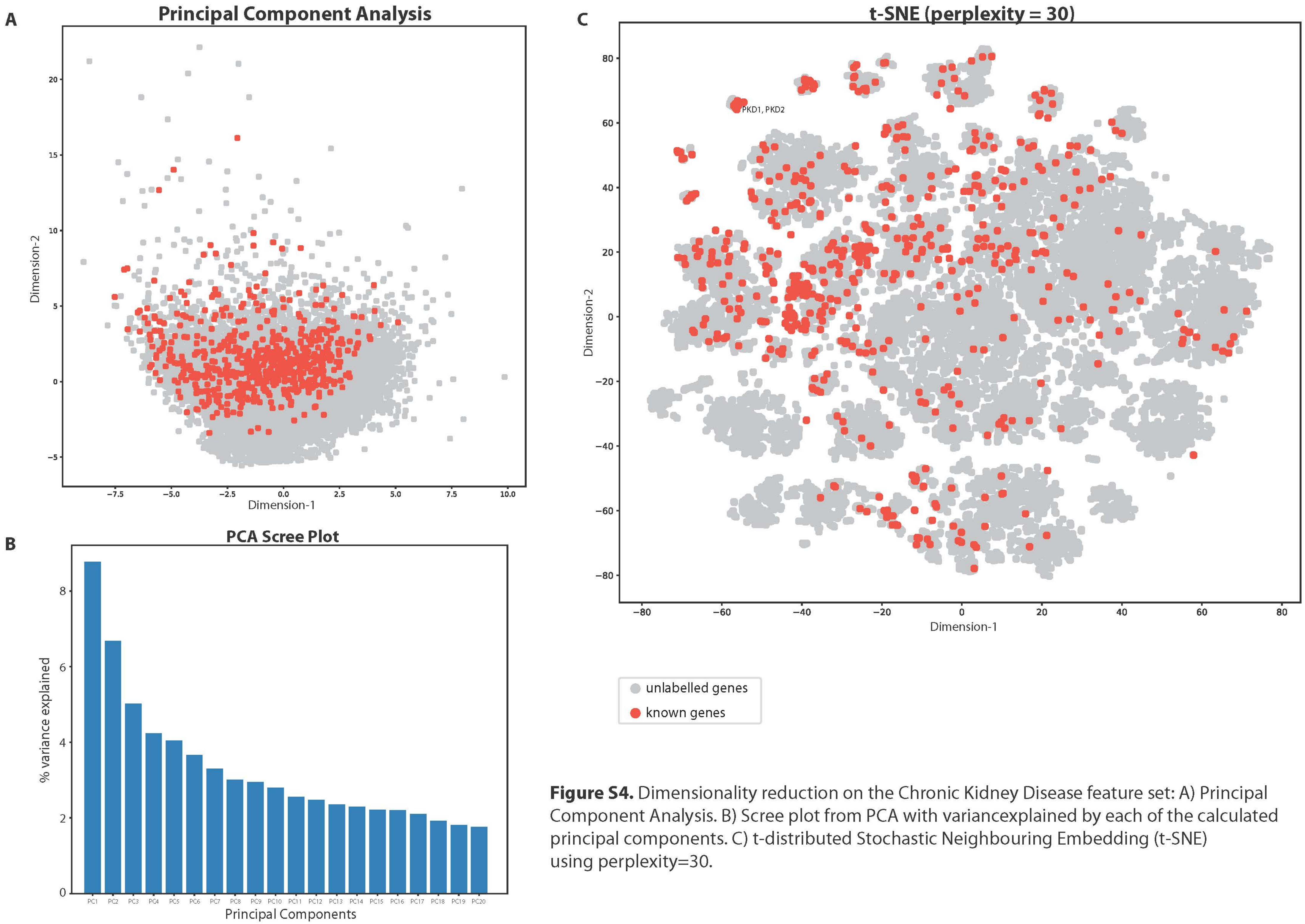

Figure S4. Dimensionality reduction on the Chronic Kidney Disease feature set: A) Principal Component Analysis. B) Scree plot from PCA with variancexplained by each of the calculated principal components. C) t-distributed Stochastic Neighbouring Embedding (t-SNE) using perplexity=30.

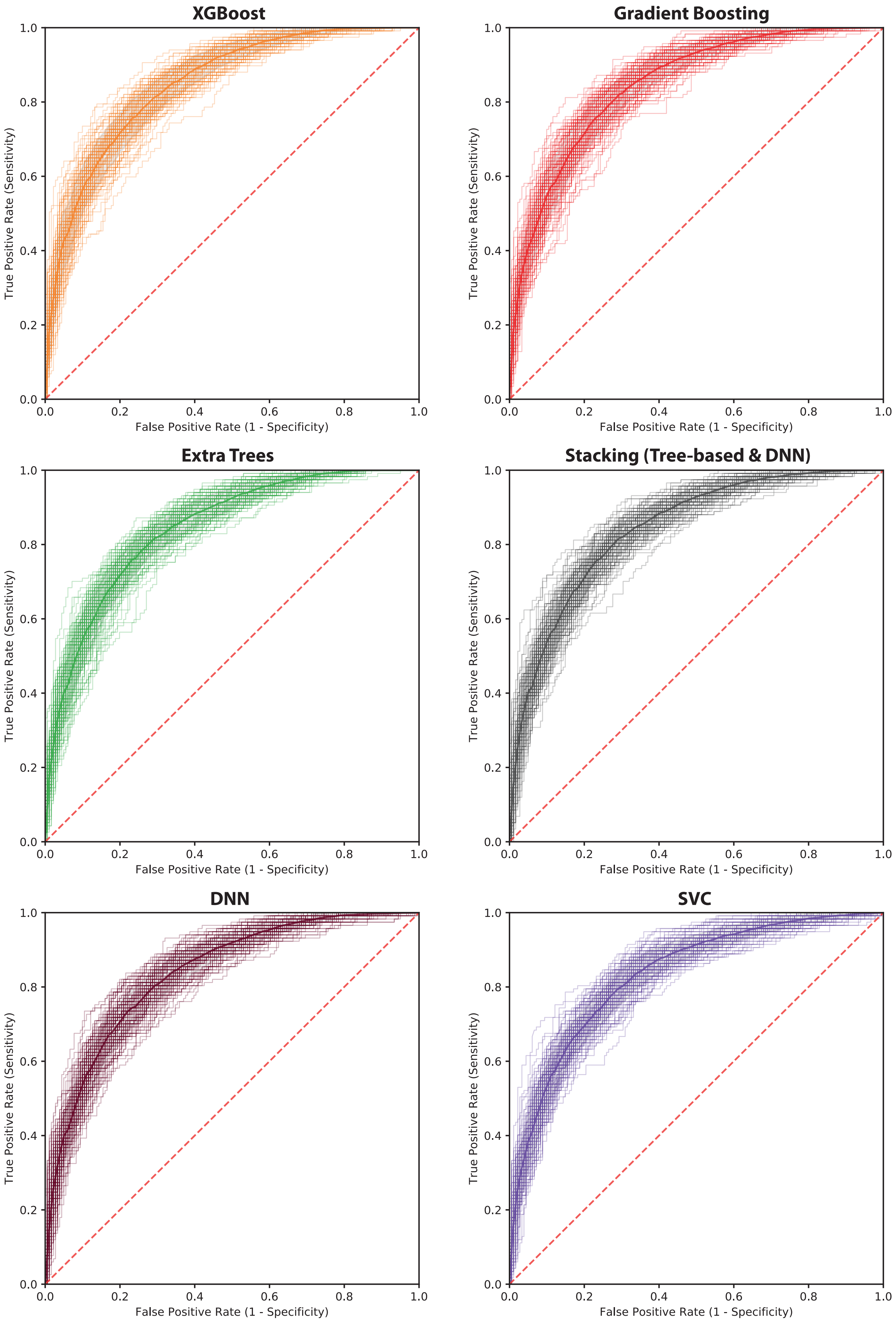

Figure S5. ROC curves from 10 batches of 10-fold Cross Validation with 6 different classiffers, in decreasing order of mean AUC: a) Random Forest, b) Xtreme Gradient Boosting, c) Gradient Boosting, d) Extra Trees, e) Stacking Classifier (1st layer: Extra Trees + Random Forest + Gradient Boosting + SVC; 2nd layer: DNN), f) Deep Neural Net (2-hidden layers) and g) Support Vector Classiffer.

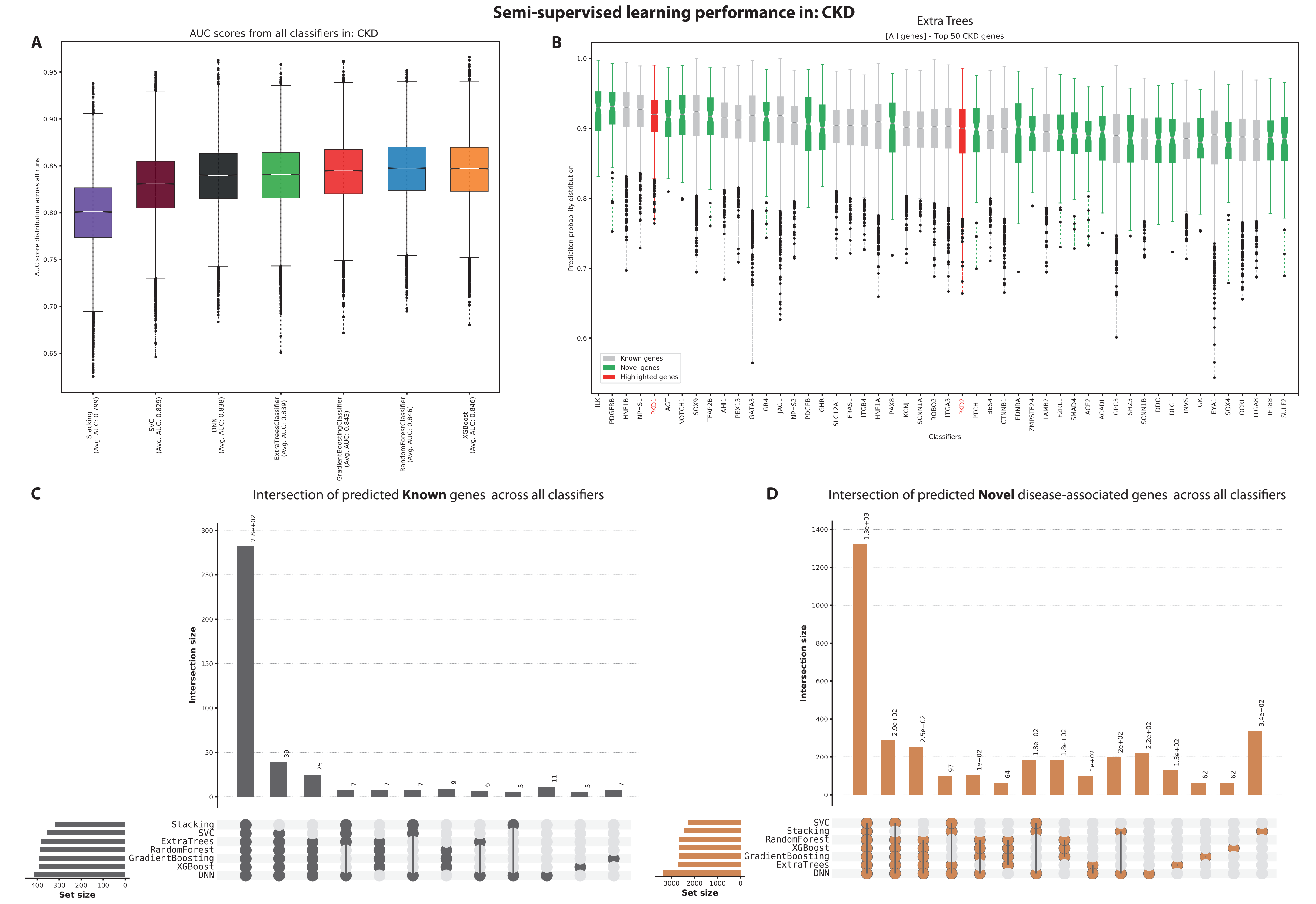

Figure S6. Mantis-ml performance on the Chronic Kidney Disease case. A) AUC score distribution per standard classifier used during mantis-ml training. B) Prediction probabilities from the top 50 (known and novel) genes predicted with Extra Trees as the standard classifier. C/D) Intersection sets of predicted known/novel genes across all classifiers.

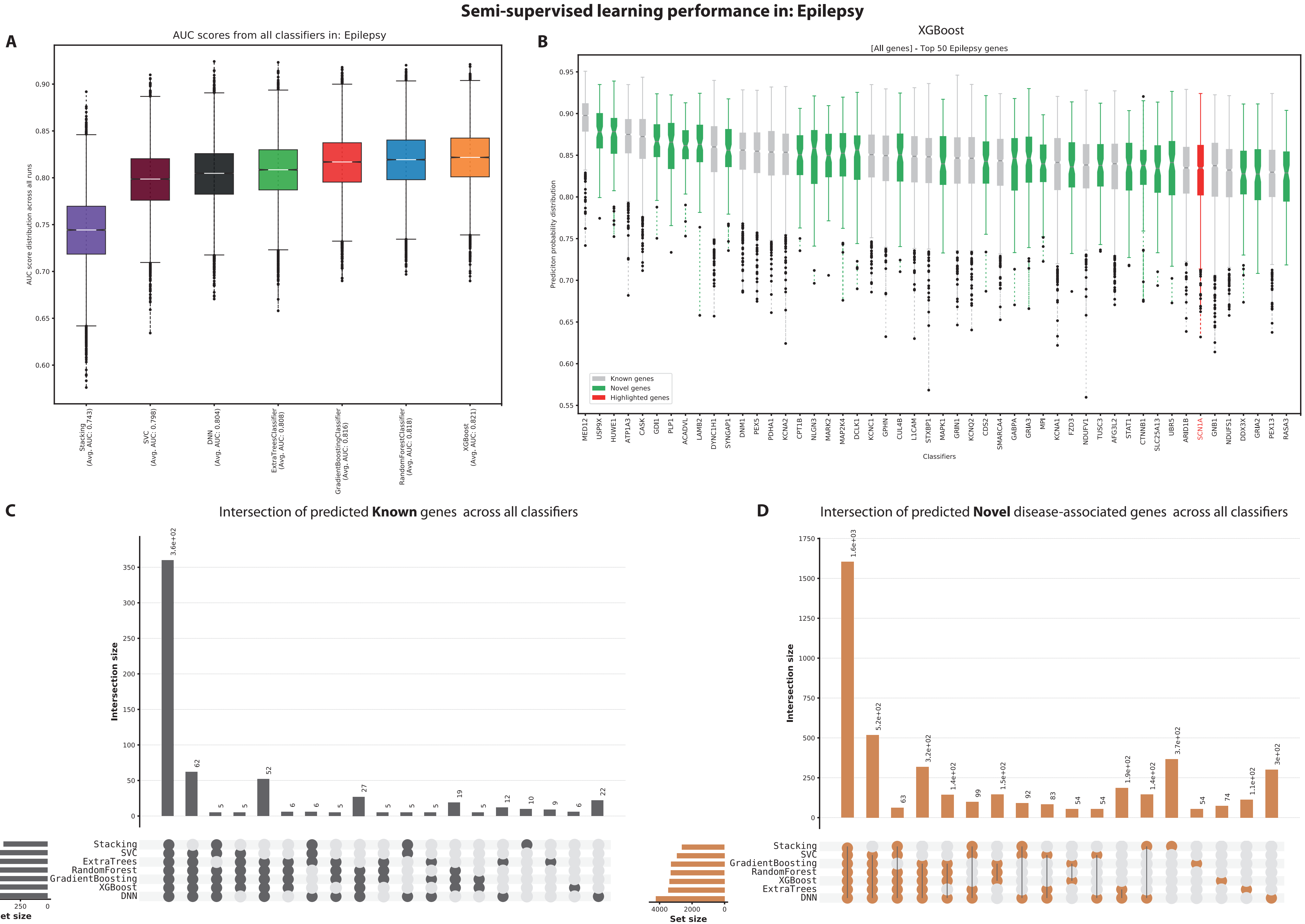

Figure S7. Mantis-ml performance on the Epilpesy disease case. A) AUC score distribution per standard classifier used during mantis-ml training. B) Prediction probabilities from the top 50 (known and novel) genes predicted with XGBoost as the standard classifier. C/D) Intersection sets of predicted known/novel genes across all classifiers.

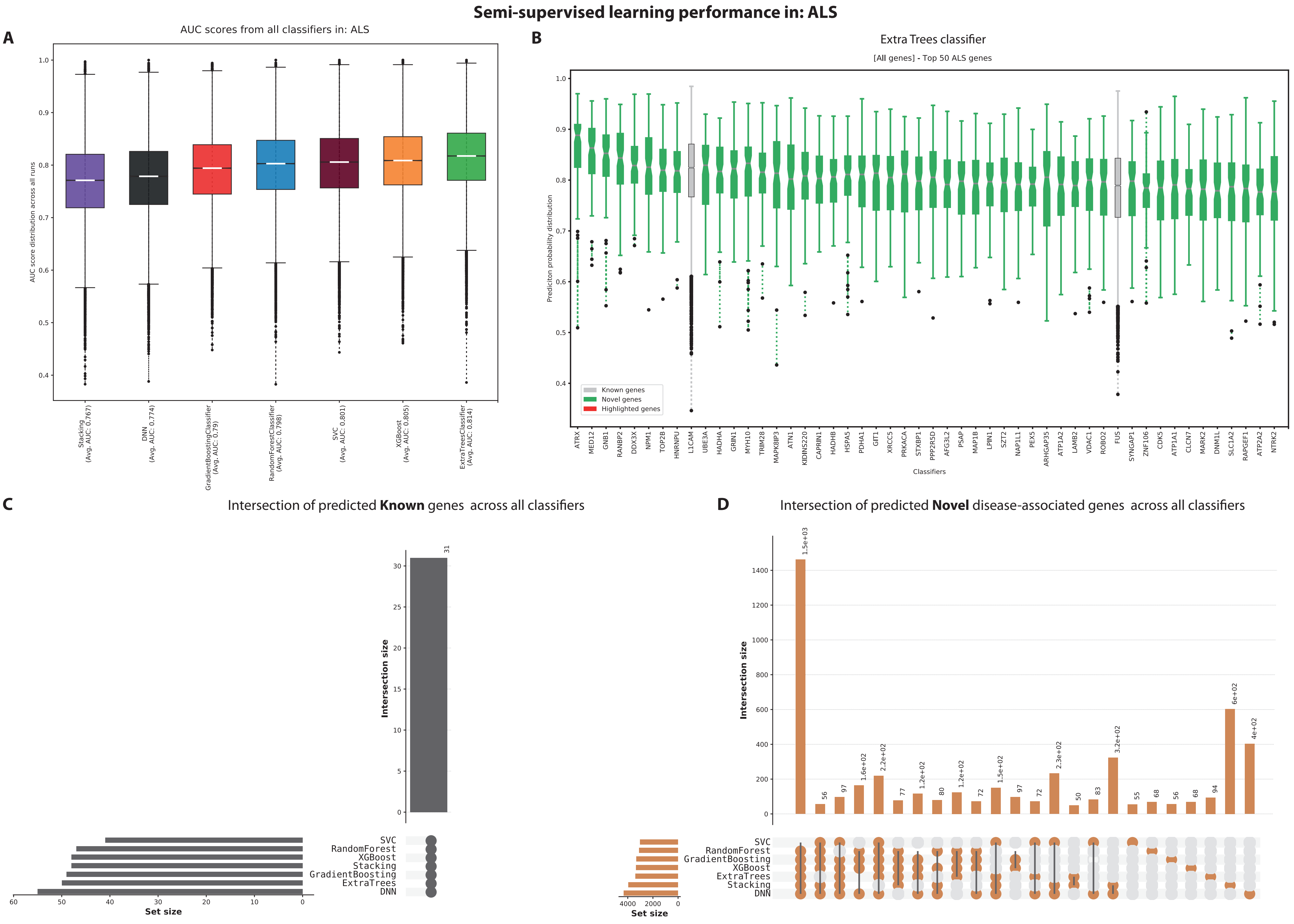

Figure S8. Mantis-ml performance on the ALS disease case. A) AUC score distribution per standard classifier used during mantis-ml training. B) Prediction probabilities from the top 50 (known and novel) genes predicted with Extra Trees as the standard classifier. C/D) Intersection sets of predicted known/novel genes across all classifiers.

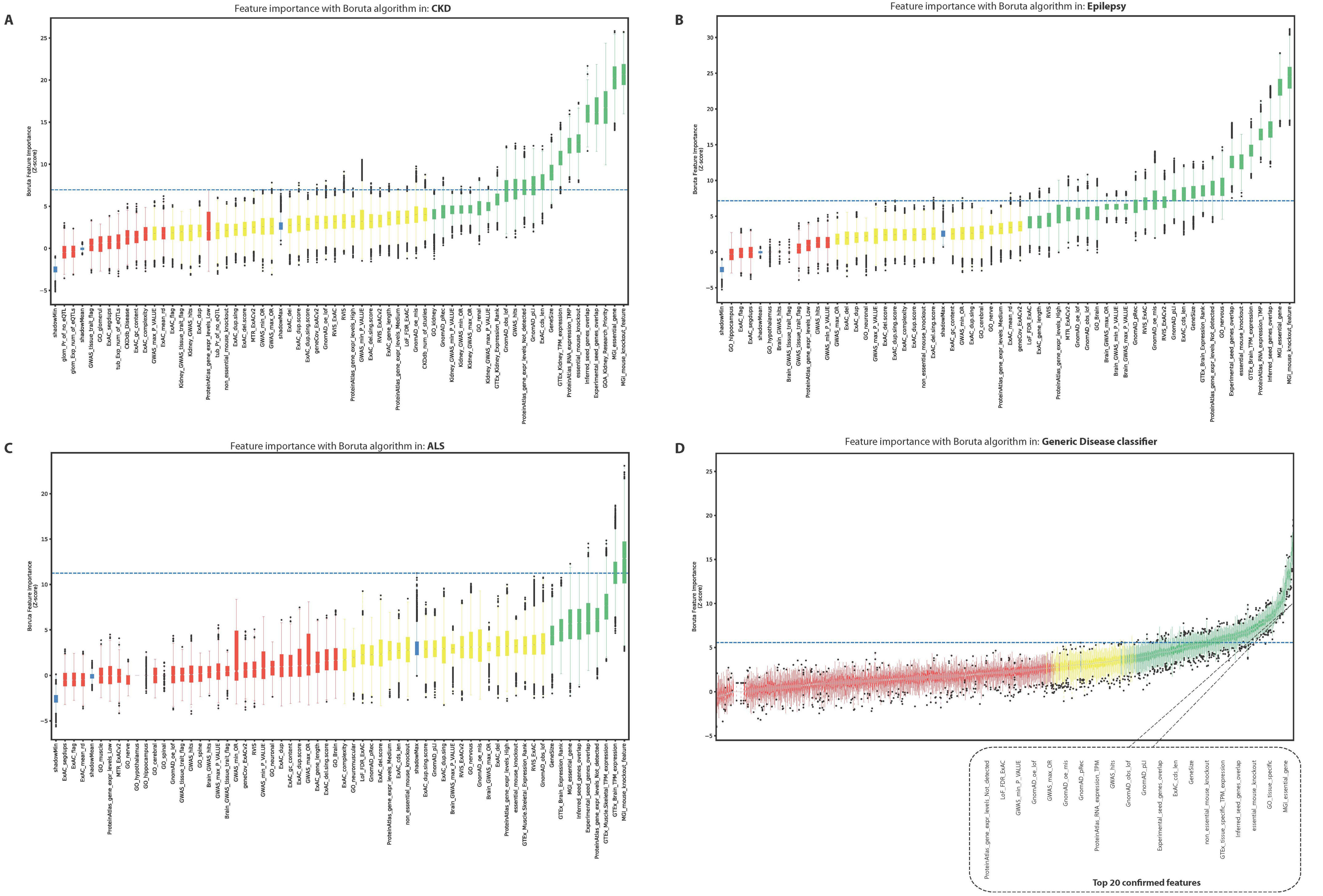

Figure S9. Distribution of feature importance scores extracted by a Random Forest classifier with the Boruta algorithm. Predictions are extracted across 100 balanced gene subsets with 10-fold cross-validation for the Chronic Kidney Disease (A), Epilepsy (B) and Amyotrophic Lateral Sclerosis (C) cases and for 1 balanced dataset with 10-fold cross validation for the Generic classifier (D). Confirmed features are shown in green, tentative in yellow and rejected ones with red. The random permuted features that are calculated as references by Boruta are shown in blue ('shadow' features). The top 20 confirmed features are shown for the Generic classifier.

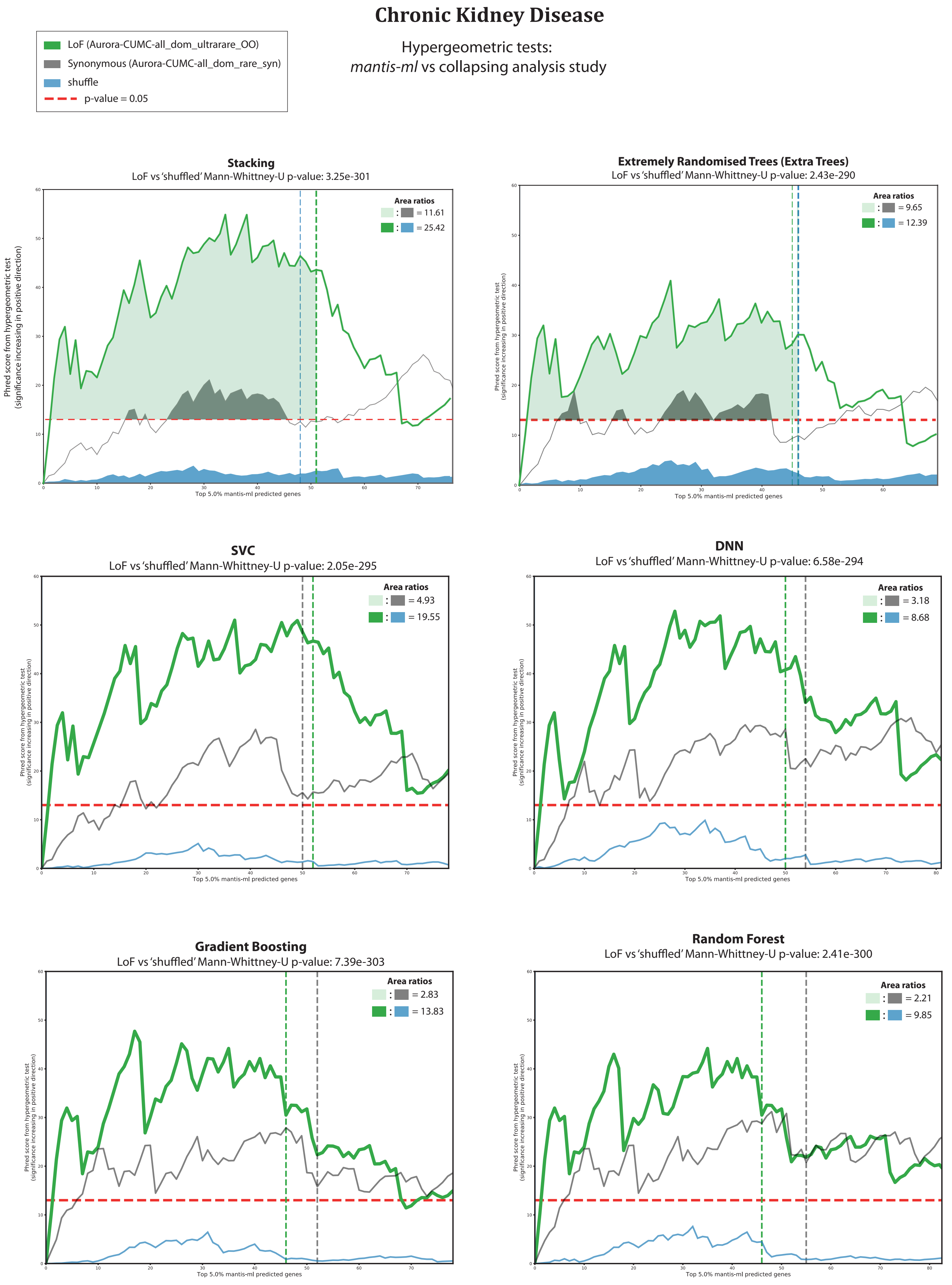

Figure S10. Cross-validation of mantis-ml predictions per classifier with rare-variant collapsing analysis results (applied for the Chronic Kidney Disease example)

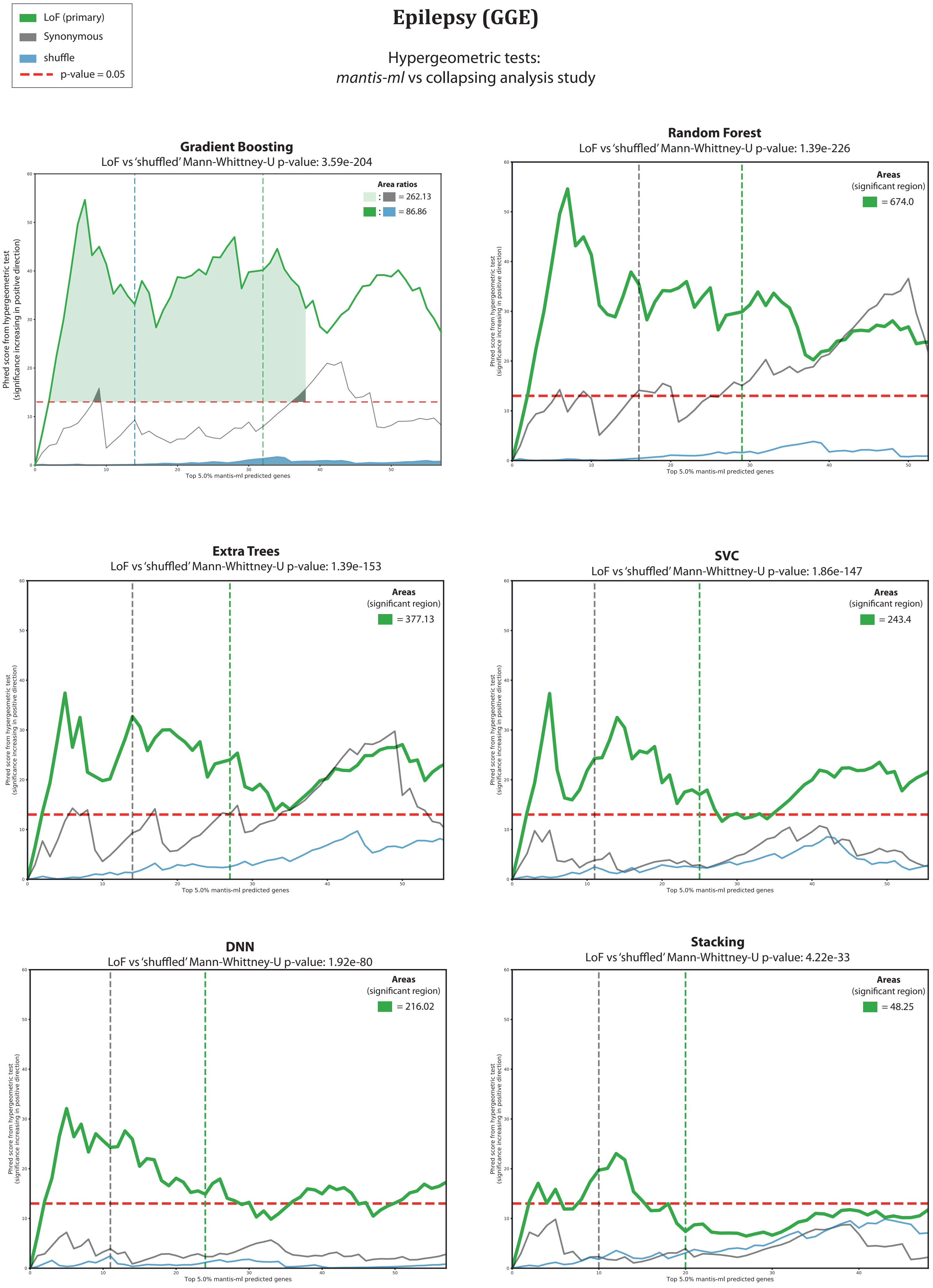

Figure S11. Cross-validation of mantis-ml predictions per classifier with rare-variant collapsing analysis results (applied for the Epilepsy disease example)

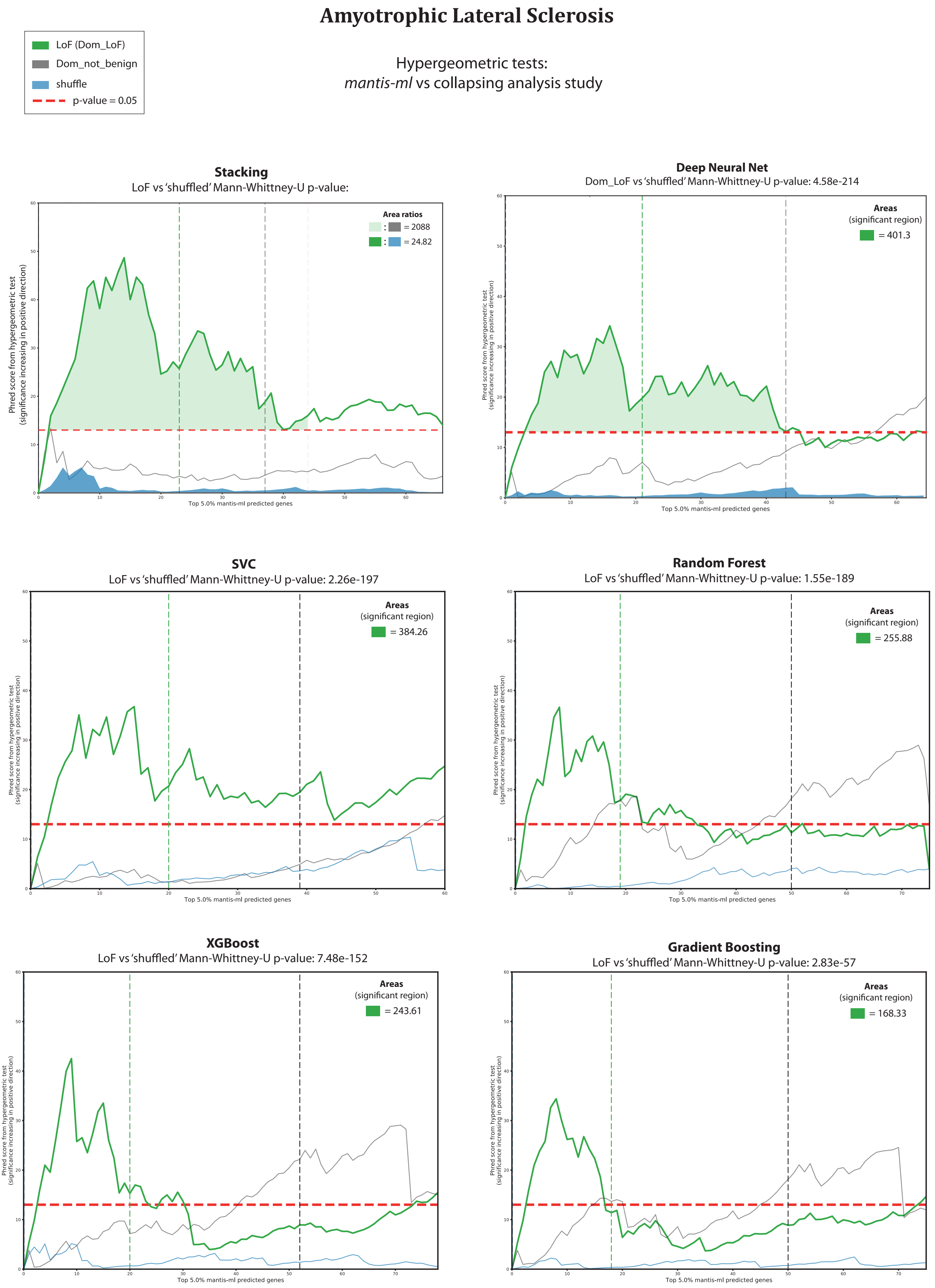

Figure S12. Cross-validation of mantis-ml predictions per classifier with rare-variant collapsing analysis results (applied for the ALS disease exampleh)

Consensus of mantis-ml 'known gene' predictions after overlap with collapsing results

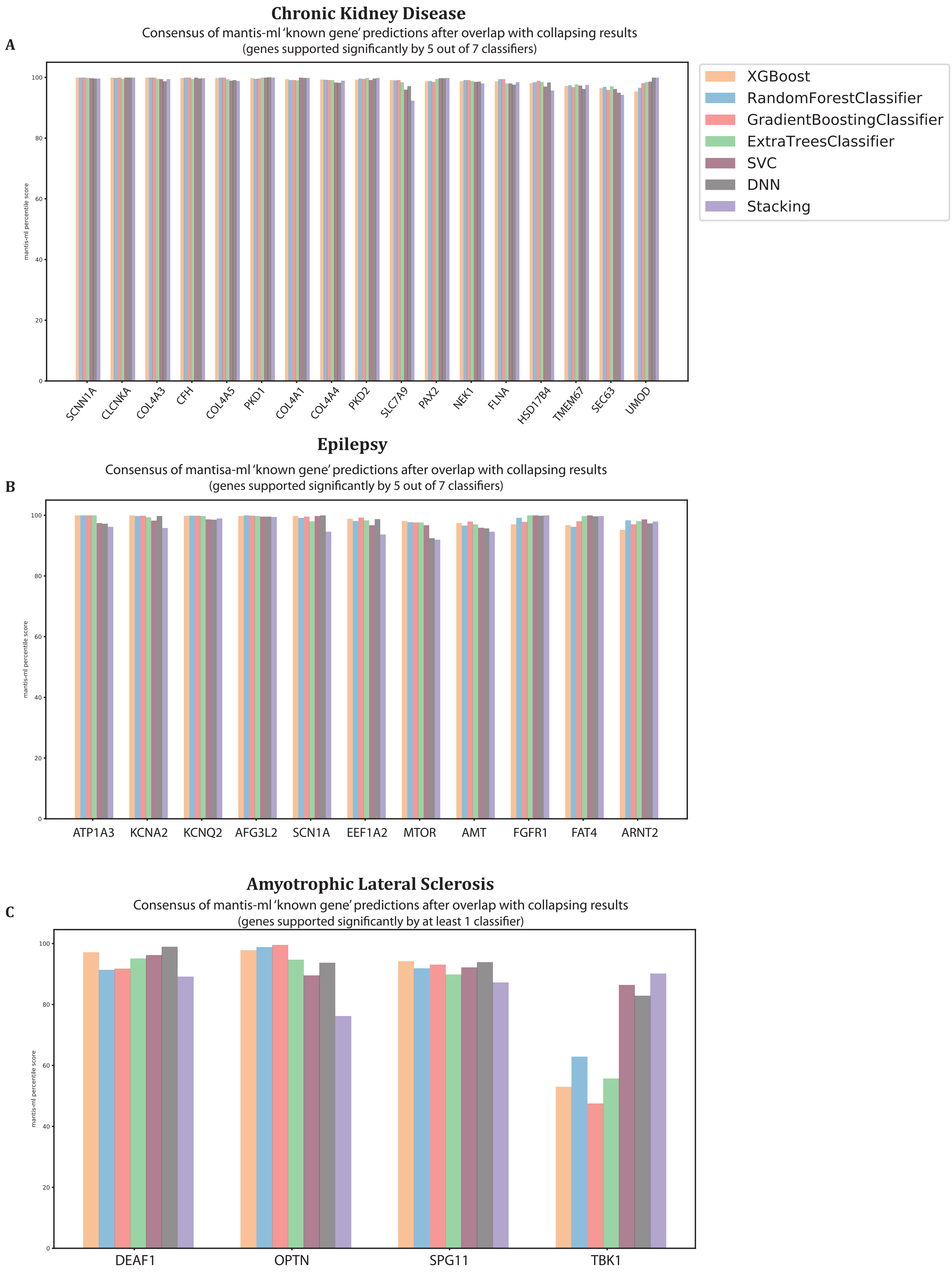

Figure S13. Consensus known genes across three disease cases: CKD (A), Epilepsy (B) and ALS (C), satisfying the significance threshold criteria in both the collapsing analysis results and the hypergeometric, supported by multiple numbers of classifiers used by mantis-ml.

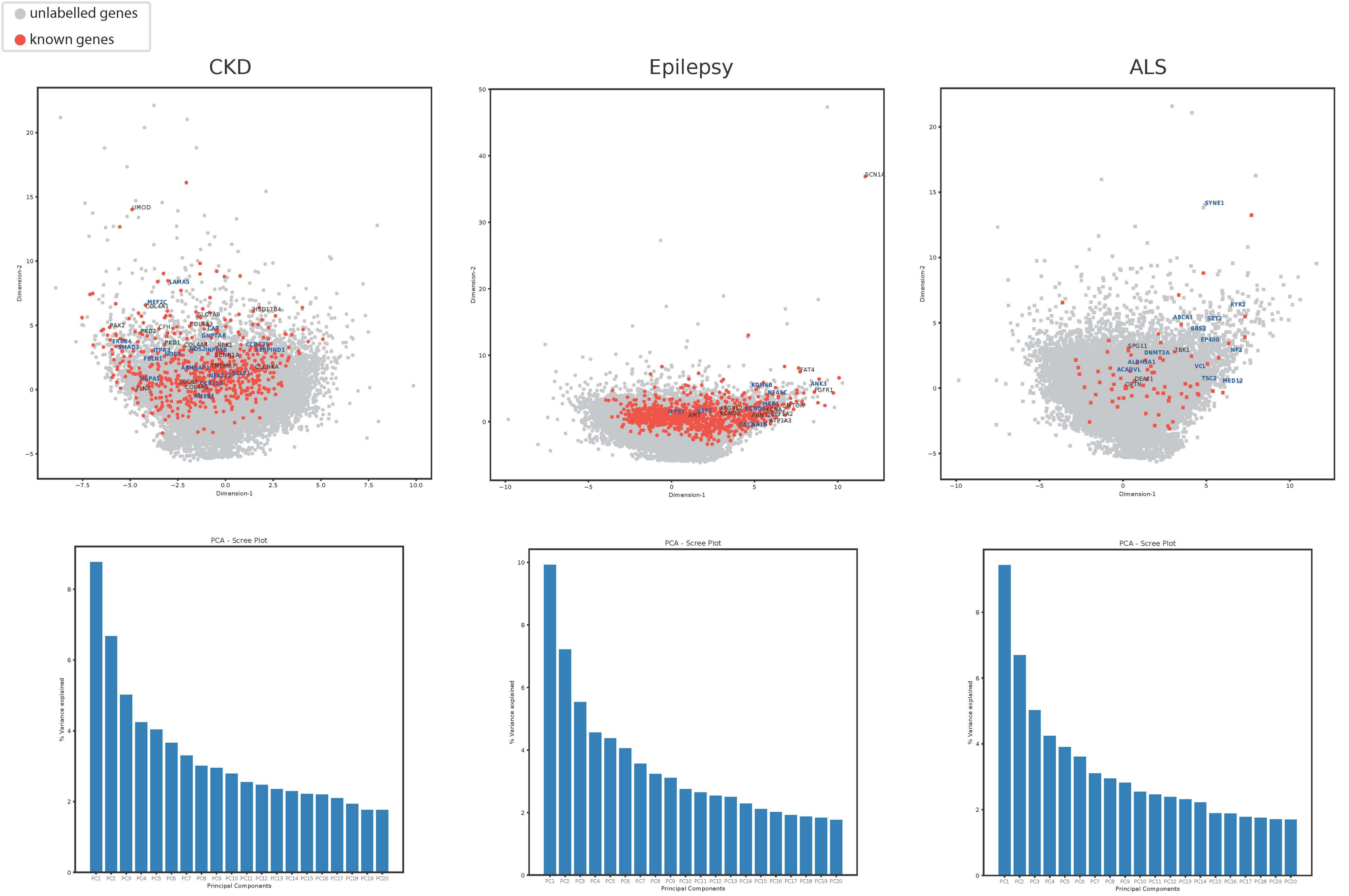

Figure S14. Principal Component Analysis (PCA) plots and Scree plots (variance explained by PCA components) for each disease example. Labelled genes are all the consensus novel (dark blue) and known genes predicted by overlapping mantis-ml predictions with rare-variant collapsing analysis results.

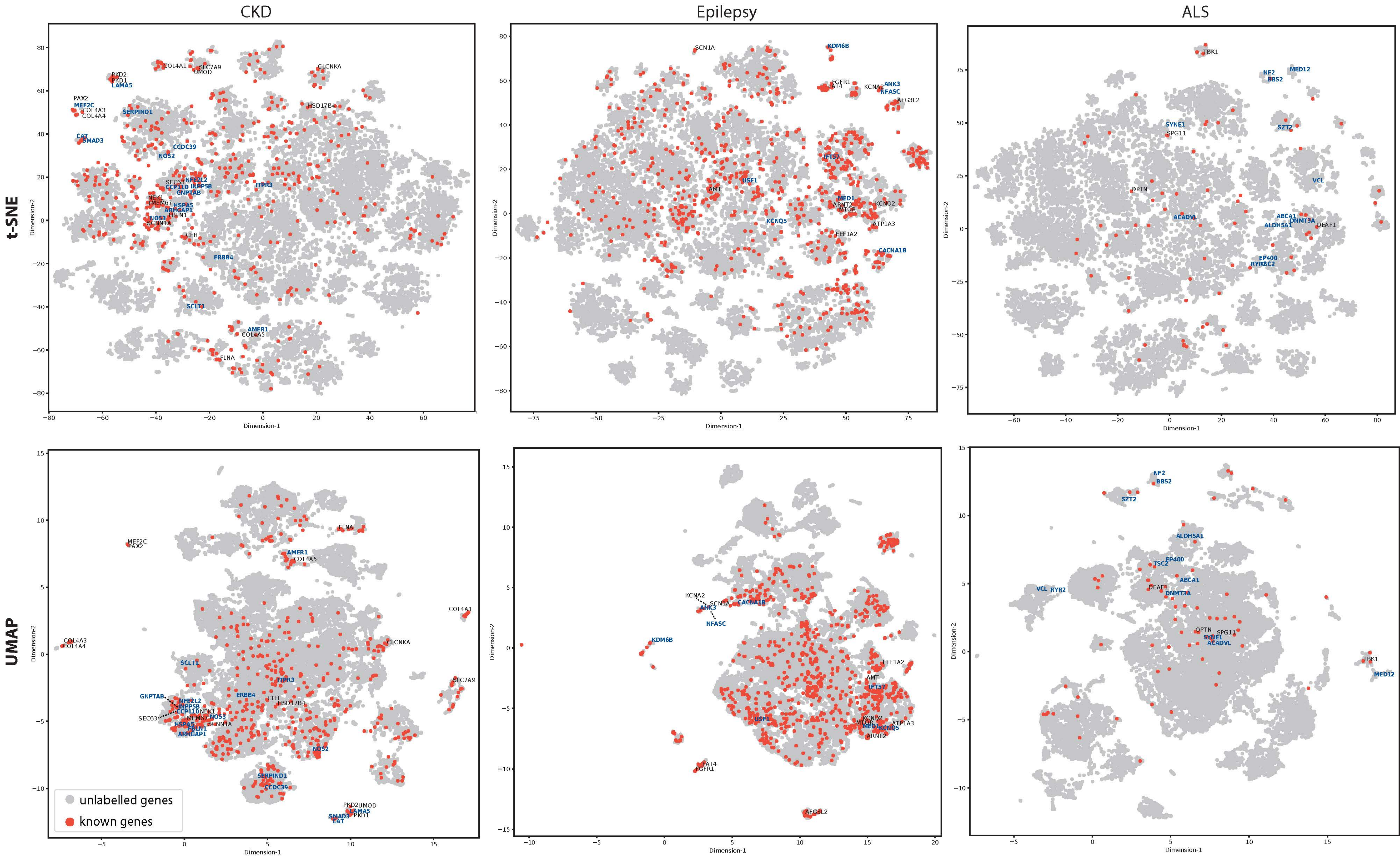

Figure S15. t-Distributed Stochastic Neighbor Embedding (t-SNE) and Uniform Manifold Approximation and Projection (UMAP) plots for 2D visualisation of all genes in each disease example. Labelled genes are all the consensus novel (dark blue) and known genes predicted by overlapping the mantis-ml predictions with rare-variant collapsing analysis results.

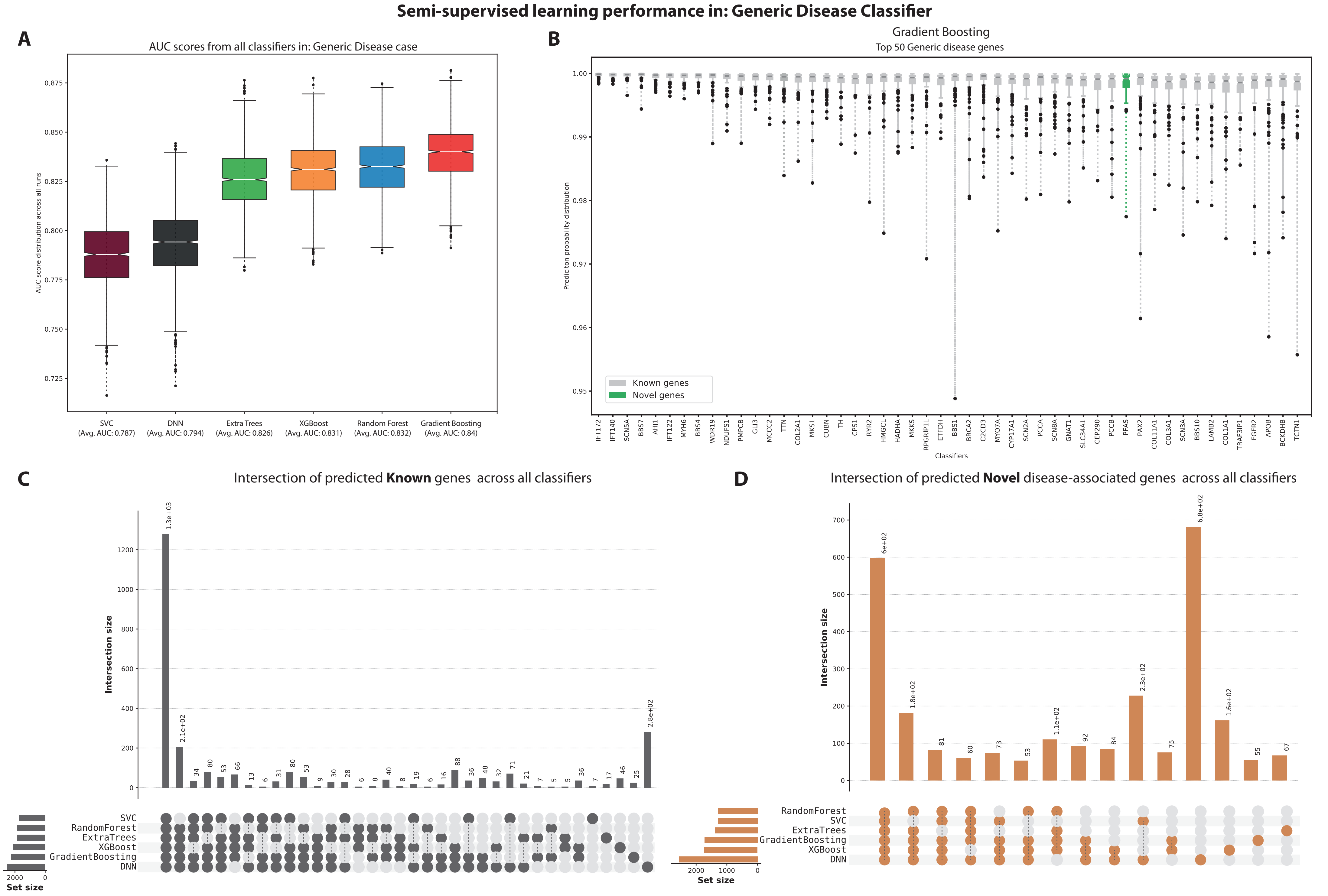

Figure S16. Mantis-ml performance on the Generic Disease case. A) AUC score distribution per standard classifier used during mantis-ml training. B) Prediction probabilities from the top 50 (known and novel) genes predicted with Gradient Boosting as the standard classifier. C/D) Intersection sets of predicted known/novel genes across all classifiers.

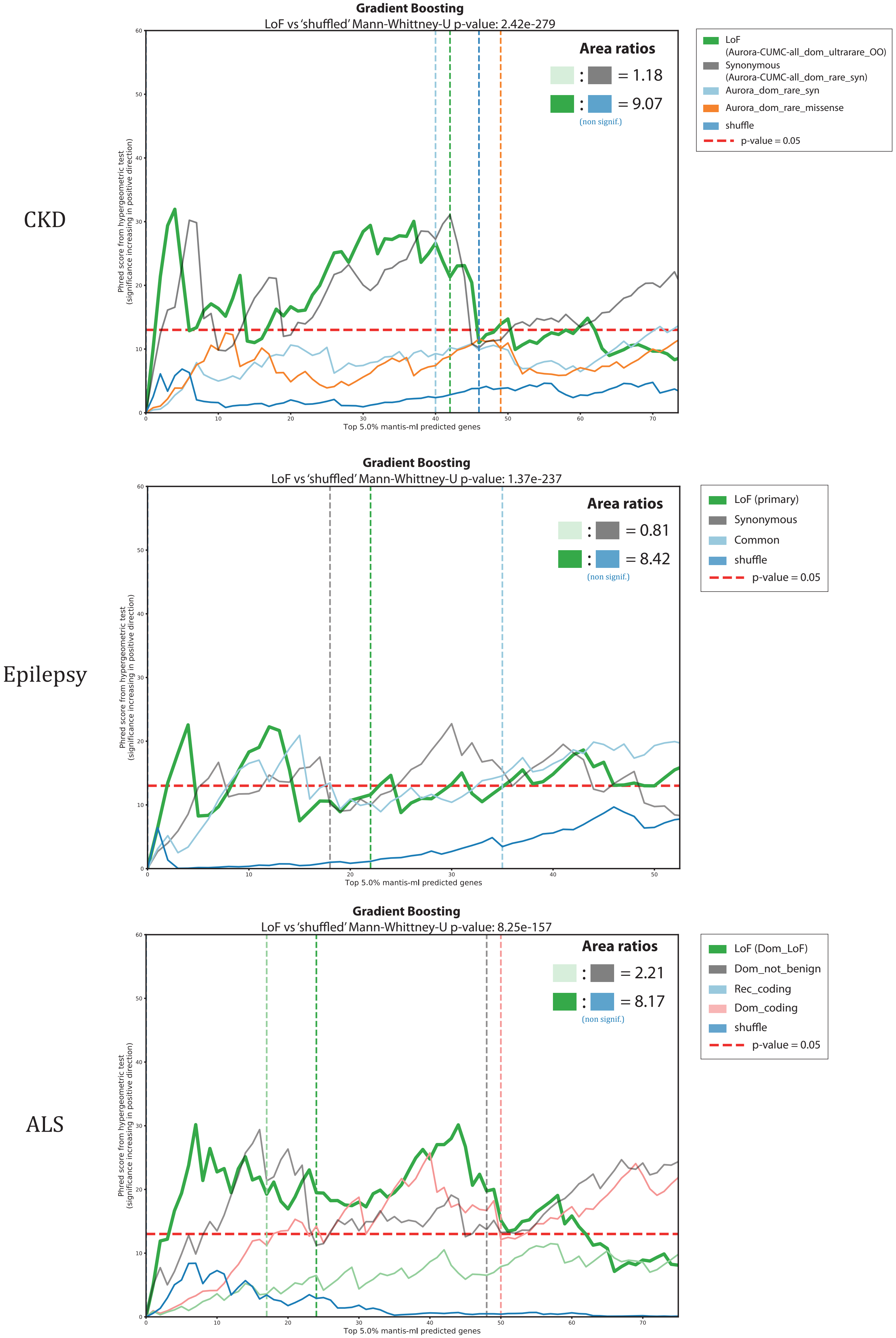

Figure S17. Enrichment (from hypergeometric test) of Generic mantis-ml predictions on disease specific collapsing analysis results from CKD, Epilepsy and ALS related studies.

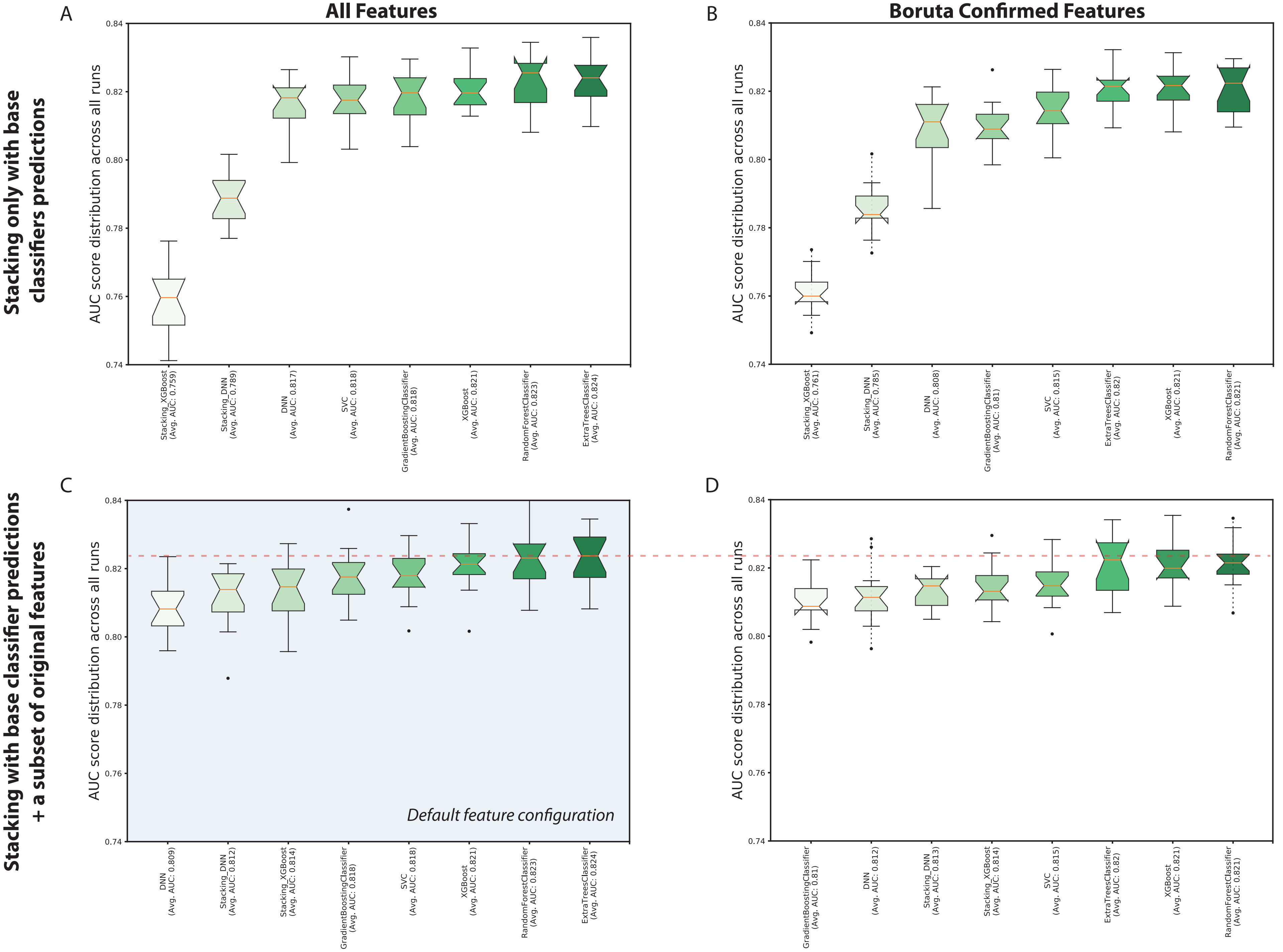

Figure S18. Benchmarking: AUC scores distribution from all classifiers in different feature selection configurations: a) using all features (after filtering) and training Stacking with base classifier predictions only, b) using only Boruta confirmed features and training Stacking with base classifier predictions + all Boruta confirmed features, c) using all features (after filtering) and training Stacking with base classifier predictions + all Boruta confirmed features and d) using only Boruta confirmed features and training Stacking with base classifier predictions + all Boruta confirmed features. Trainind data for benchmarking were compiled based on the Chronic Kidney Disease test case and included 15 random balanced datasets. Best average AUC performance was achieved by configuration (c) for all tested classifiers.

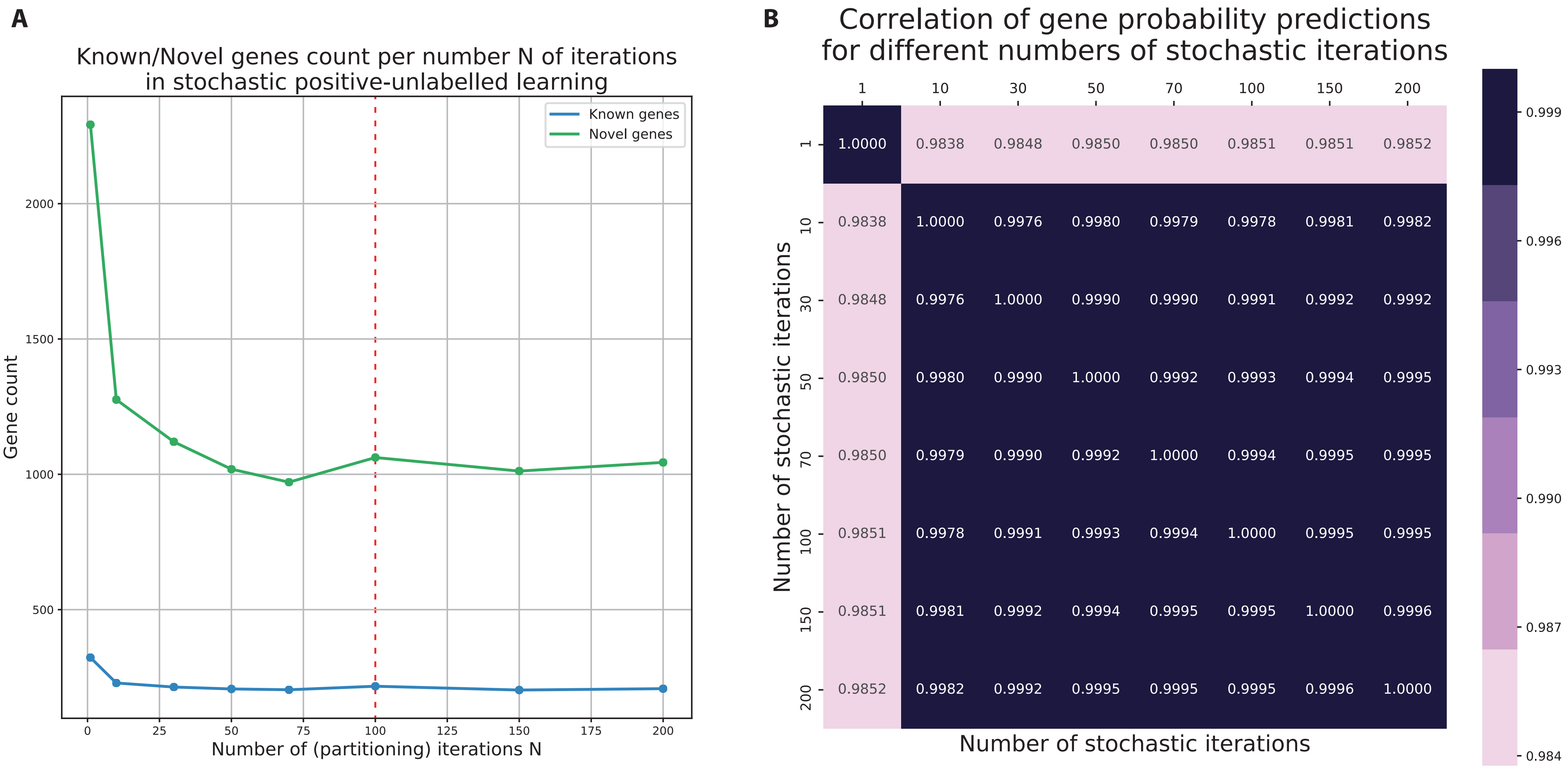

Figure S19. Benchmarking for: A) sensitivity check of the number of stochastic iterations to the predicted known and novel genes count and B) robustness of gene probability predictions for different numbers of iterations. (Tested numbers of iterations = [1, 10, 30, 50, 70, 100, 150, 200]).

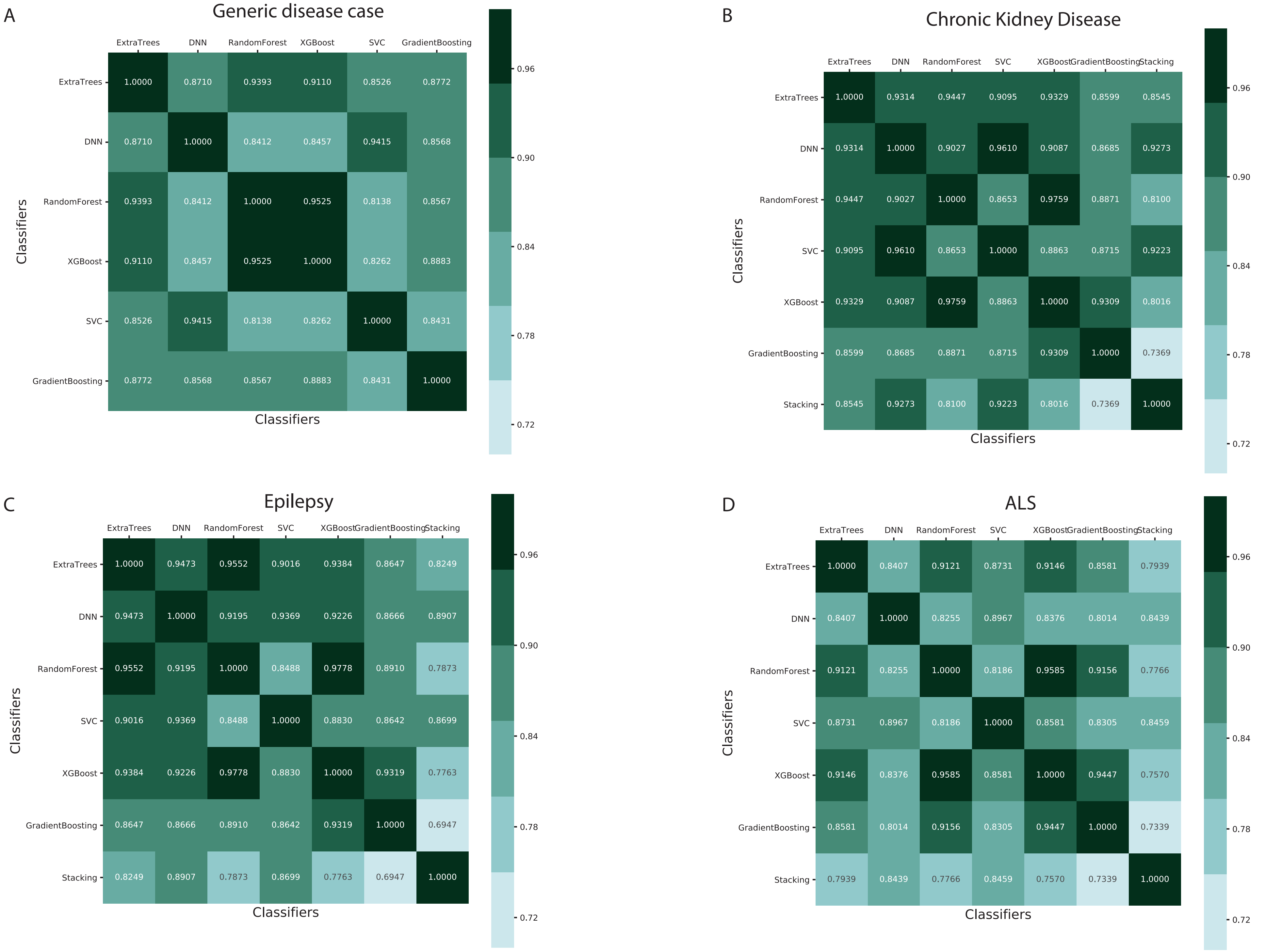

Figure S20. Correlation of gene probability predictions between different classifiers on the: A) Generic Disease, B) Chronic Kidney Disease, C) Epilepsy and D) ALS disease examples.

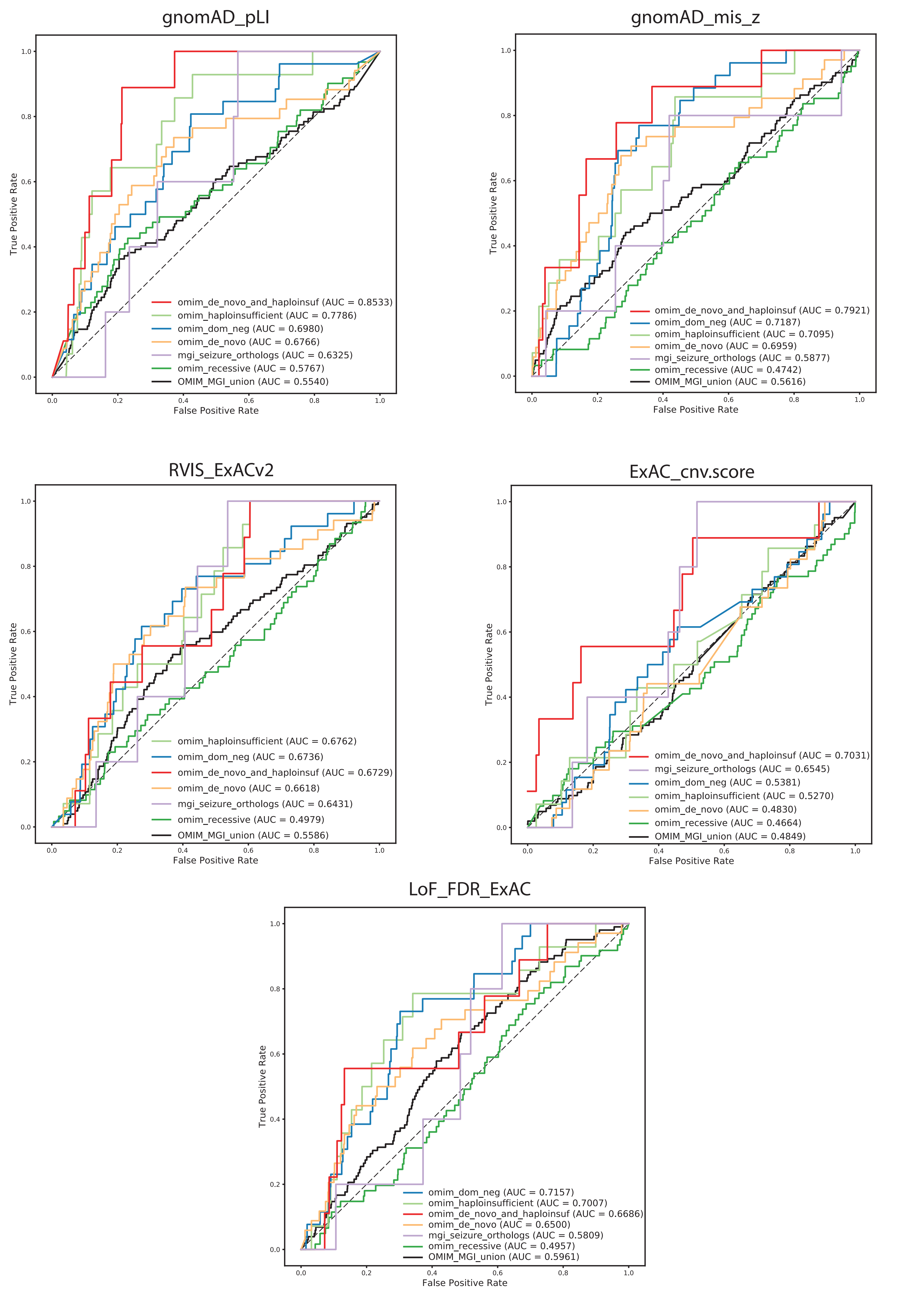

Figure S21. Predictive power of various intolerance scores for distinguishing different OMIM- and MGI-based genes from non-OMIM/MGI genes: gnomAD_pLI, gnomAD_mis_z, RVIS_ExACv2, ExAC_cnv.score and LoF_FDR_ExAC.

Cross-validation of mantis-ml predictions for "known" and "novel" genes against cohort-level rare-variant association studies

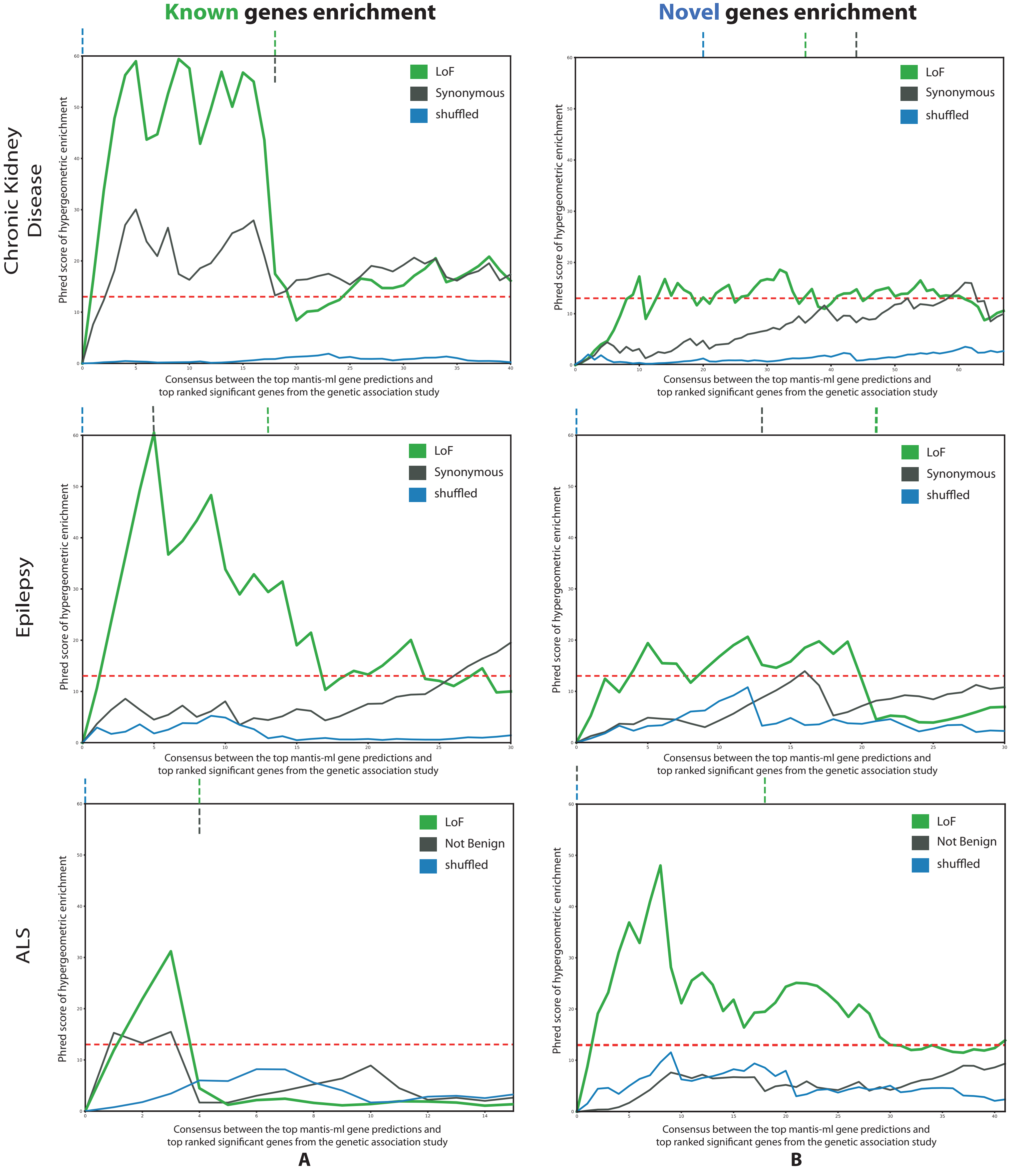

Figure S22. Hypergeometric test enrichment of disease-specific mantis-ml predictions for **A)** Known and **B)** Novel genes, against collapsing analysis results from CKD, Epilepsy and ALS cohorts and for different types of variants: Loss-of-Function (LoF), synonymous (if available) and shuffled. For the ALS disease example, a "Not Benign" class has been used (due to lack of a "synonymous variant"-based dataset), which represents a non-benign but less pathogenic than LoF set. The horizontal dashed red line corresponds to the significance threshold of p=0.05 for the hypergeometric tests. Where the plot(s) go above this line highlight significant enrichment of mantis-ml top gene predictions being enriched for among the population genomic collapsing analyses. The vertical dashed lines indicate the last index of top ranked genes from the collapsing analyses achieving a p-value < 0.05. For each disease example, the mantis-ml ranking used for performing the enrichment tests is the one extracted by the classifier with the highest average AUC performance (XGBoost for CKD and Epilepsy, ExtraTrees for ALS).

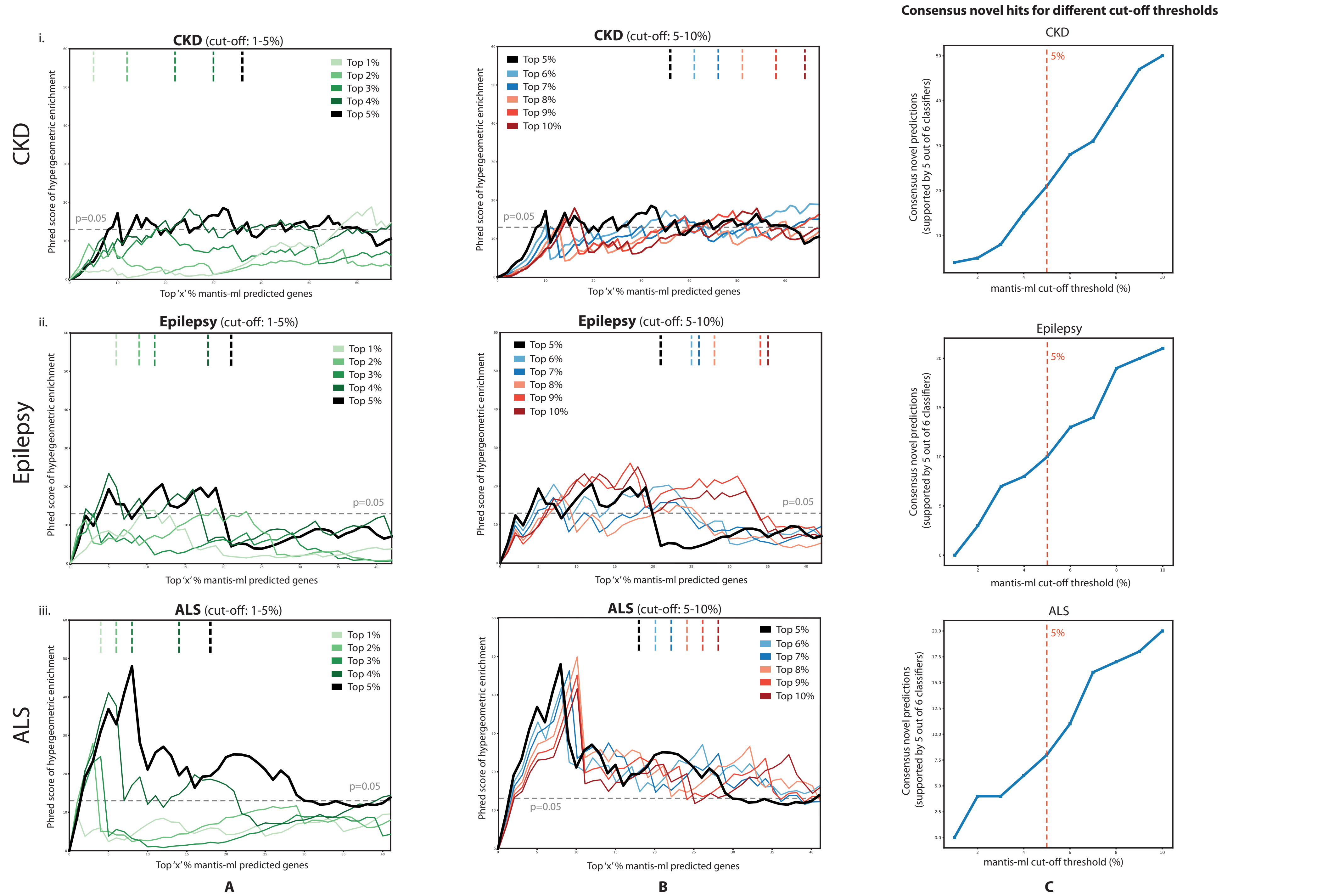

Figure S23. Sensitivity analysis for cut-off threshold of top mantis-ml "novel" gene predictions. **A & B)** Hypergeometric enrichment of collapsing analysis results (based on LoF variants) against different proportions of top mantis-ml predictions (1-10%) for three diseases: i) Chronic Kidney Disease, ii) Epilepsy and iii) Amyotrophic Lateral Sclerosis. **C)** Number of consensus novel gene predictions for different mantis-ml cut-off thresholds.

CKD

"Novel" genes (1-5% cutoff)
(Predicted by 5 out of 6 classifiers)

Top 1% : AMER1, LAMA5, NOS3, MEF2C (4 hits)

Top 2% : AMER1, LAMA5, NOS3, MEF2C, NOS2 (5 hits)

Top 3% : AMER1, LAMA5, NOS3, MEF2C, NOS2, GNPTAB, CAT, SMAD3 (8 hits)

Top 4% : AMER1, LAMA5, CCP110, NOS3, MEF2C, NOS2, GNPTAB, INPP5B, CAT, SMAD3, HSPA5, FBLN1, I TPR3, ARHGAP1, ERBB4 (15 hits)

Top 5% : AMER1, LAMA5, CCP110, NOS3, MEF2C, SCLT1,NOS2, IRS1, GNPTAB, INPP5B, CAT, CCDC39, SMAD3, HSPA5, FBLN1, ITPR3, MTTP, NFE2L2, ARHGAP1, ERBB4, M6PR (21 hits)

"Novel" genes (6-10% cutoff)
(Predicted by 5 out of 6 classifiers)

Top 6% : AMER2, LAMA5, CCP110, NOS3, MEF2C, SCLT1, NOS2, IRS1, SERPIND1, GNPTAB, HSPA4L, INPP5B, CAT, CCDC39, SMAD3, LRRFIP1, HSPA5, FBLN1, ITPR3, MTTP, CYP4F3, NFE2L2, ARHGAP1, ERBB4, GNE, ASH1L, M6PR, CTNNA1 (28 hits)

Top 7% : AMER1, LAMA5, CCP110, NOS3, MEF2C, SCLT1, NOS2, IRS1, SERPIND1, GNPTAB, HSPA4L, INPP5B, CAT, CCDC39, SMAD3, LRRFIP1, HSPA5, FBLN1, GCGR, ITPR3, MTTP, CYP4F3, NFE2L2, ARHGAP1, ERBB4, GNE, LIPA, ASH1L, M6PR, EFEMP1, CTNNA1 (31 hits)

Top 8% : AMER1, LAMA5, CCP110, NOS3, MEF2C, SCLT1, NOS2, IRS1, SERPIND1, GNPTAB, HSPA4L, INPP5B, CAT, CCDC39, SMAD3, LRRFIP1, HSPA5, FBLN1, GCGR, ITPR3, MTTP, CYP4F3, NFE2L2, ARHGAP1, ERBB4, GNE, LIPA, ASH1L, M6PR, PANK1, HOXD4, PAM, ITSN2, EFEMP1, PDE2A, CDH6, CDH2, SZT2, CTNNA1 (39 hits)

Top 9% : AMER1, LAMA5, CCP110, NOS3, MEF2C, SCLT1, NOS2, IRS1, DSG2, SERPIND1, GNPTAB, HSPA4L, INPP5B, CAT, CCDC39, ROBO3, SMAD3, LRRFIP1, HSPA5, FBLN1, GCGR, C5, ITPR3, MTTP, CYP4F3, SVIL, NFE2L2, ARHGAP1, ERBB4, GNE, LIPA, ASH1L, M6PR, PANK1, SLC12A7, HOXD4, PAM, ITSN2, EFEMP1, PLCB3, EIF2AK4, PDE2A, CDH6, CDH2, SZT2, TXNRD2, CTNNA1 (47 hits)

Top 10% : AMER1, LAMA5, CCP110, NOS3, MEF2C, SCLT1, NOS2, IRS1, DSG2, SERPIND1, GNPTAB, HSPA4L, INPP5B, CAT, CCDC39, ROBO3, SMAD3, LRRFIP1, HSPA5, FBLN1, GCGR, C5, ITPR3, MTTP, CYP4F3, SVIL, NFE2L2, ARHGAP1, ERBB4, GNE, LIPA, ASH1L, M6PR, PANK1, SLC12A7, HOXD4, PAM, ITSN2, EFEMP1, MXI1, PLCB3, EIF2AK4, RTCA, PDE2A, CDH6, CDH2, SZT2, TXNRD2, PKN1, CTNNA1 (50 hits)

Epilepsy

Top 1% : None (0 hits)

Top 2% : USF1, IFT57, ANK3 (3 hits)

Top 3% : USF1, IFT57, MED1, KDM6B, ANK3, NFASC, KCNQ5 (7 hits)

Top 4% : USF1, IFT57, MED1, KDM6B, CUX1, ANK3, NFASC, KCNQ5 (8 hits)

Top 5% : CACNA1B, USF1, IFT57, MED1, DMXL2, KDM6B, CUX1, ANK3, NFASC, KCNQ5 (10 hits)

Top 6% : CACNA1B, USF1, IFT57, MED1, DMXL2, KDM6B, CUX1, ANK3, NFASC, KCNQ5, RGS4, STK3, GAK (13 hits)

Top 7% : CACNA1B, USF1, IFT57, MED1, DMXL2, KDM6B, CUX1, ANK3, NFASC, KCNQ5, RGS4, STK3, ATXN1, GAK (14 hits)

Top 8% : CACNA1B, USF1, IFT57, VPS41, MED1, DMXL2, KDM6B, CUX1, ANK3, NFASC, KCNQ5, RGS4, ALKBH1, STK3, ATXN1, GAK, EFNB1, MYO10, TYRO3 (19 hits)

Top 9% : CACNA1B, USF1, IFT57, VPS41, MED1, DMXL2, KDM6B, CUX1, ANK3, NFASC, KCNQ5, RGS4, ALKBH1, STK3, ATXN1, GAK, EFNB1, PRKCG, MYO10, TYRO3 (20 hits)

Top 10% : CACNA1B, USF1, IFT57, VPS41, MED1, DMXL2, KDM6B, CUX1, ANK3, NFASC, KCNQ5, RGS4, ALKBH1, STK3, ATXN1, GAK, EFNB1, WDR83, PRKCG, MYO10, TYRO3 (21 hits)

ALS

Top 1% : None (0 hits)

Top 2% : MED12, SZT2, ACADVL, TSC2 (4 hits)

Top 3% : MED12, SZT2, ACADVL, TSC2 (4 hits)

Top 4% : MED12, SZT2, ACADVL, TSC2, OPA1, ABCA1 (6 hits)

Top 5% : MED12, SZT2, ACADVL, NF2, TSC2, OPA1, ABCA1, DNMT3A (8 hits)

Top 6% : MED12, SZT2, ACADVL, NF2, TSC2, VCL, OPA1, ALDH5A1, SYNE1, ABCA1, DNMT3A (11 hits)

Top 7% : MED12, SZT2, ACADVL, EP400, NF2, TSC2, VCL, OPA1, ADCYAP1R1, ALDH5A1, SYNE1, ABCA1, NIN, MYOF, DNMT3A, MDN1 (16 hits)

Top 8% : MED12, SZT2, ACADVL, EP400, NF2, TSC2, POLD1, VCL, OPA1, ADCYAP1R1, ALDH5A1, SYNE1, ABCA1, NIN, MYOF, DNMT3A, MDN1 (17 hits)

Top 9% : MED12, SZT2, ACADVL, EP400, NF2, TSC2, POLD1, VCL, OPA1, ADCYAP1R1, ALDH5A1, SYNE1, ABCA1, NIN, MYOF, DNMT3A, BBS2, MDN1 (18 hits)

Top 10% : MED12, SZT2, ACADVL, EP400, NF2, TSC2, APC, POLD1, VCL, OPA1, RYR2, ADCYAP1R1, ALDH5A1, SYNE1, ABCA1, NIN, MYOF, DNMT3A, BBS2, MDN1 (20 hits)

A (1-5%) B (6-10%)

Figure S24. Sensitivity analysis for different cut-off thresholds of top mantis-ml "novel" gene predictions. Consensus of "novel" gene predictions, supported by 5 out of 6 classifiers used by mantis-ml, across the three disease examples, for different % ratios of top mantis-ml predictions.: **A)** 1-5%. **B)** 6-10%.

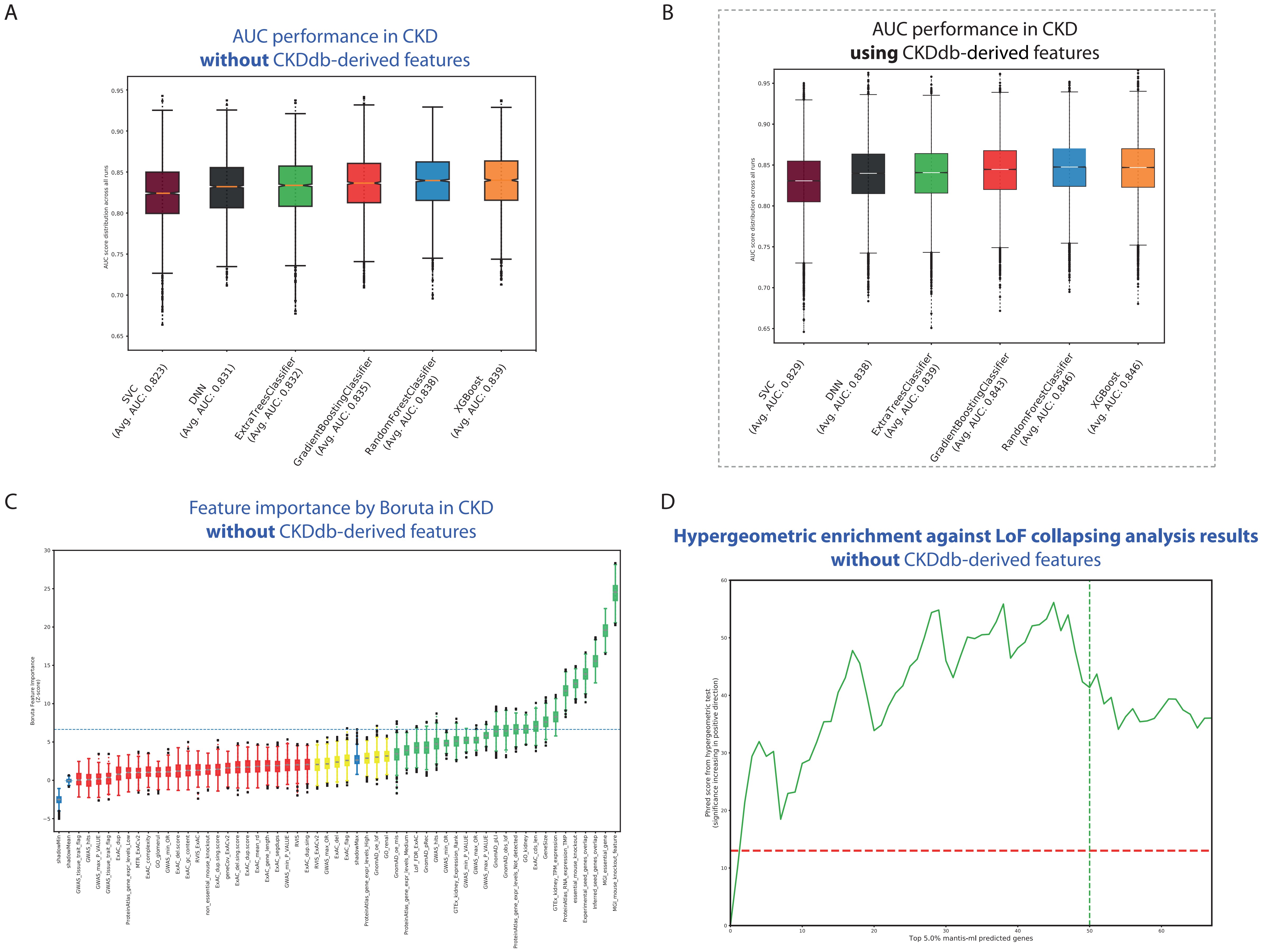

Figure S25. mantis-ml performance on CKD with/without CKDdb-derived features. **A.** AUC performance per classifier **without** using CKDdb-derived features. **B.** AUC performance per classifier **using** CKDdb-derived features. **C.** Boruta-based feature importance **without** using CKDdb-derived features. **D)** Hypergeometric enrichment of top 5% mantis-ml predictions against top collapsing analysis hits (based on LoF variants) **without** using CKDdb-derived features.

SUPPLEMENTAL METHODS

Annotation of disease/phenotype-associated genes

We are using the Human Phenotype Ontology (HPO) as our default resource to annotate disease/phenotype-associated genes. HPO contains over 13,000 terms and over 156,000 annotations to hereditary diseases. It is a comprehensive resource that leverages information from the collection of OMIM, DECIPHER, Orphanet and the medical literature. HPO originally started with a focus on Mendelian diseases but has gradually expanded onto other more common complex disease areas, such as cardiology and immunology, through regular workshops with clinicians. The use of HPO as our default annotation resource is consistent with other methods that we benchmarked to, such as Phenolyzer, which is also based on it.

Annotation of known disease-associated genes is performed by automatically selecting genes whose 'HPO-Term-Name' contains any of the 'Disease/Phenotype terms' and does not contain any of the 'Diseases/Phenotypes to exclude' from the config.yaml configuration file. Alternatively, the mantis-ml framework permits the use of user-specified positive label seed genes in place of the HPO mapping. This is accessible via the "*-k*" option with the *mantisml* command.

Distinct machine learning architecture of the mantis-ml framework

mantis-ml implements a novel design of a stochastic semi-supervised learning framework. To our knowledge, there is only one other instance of a "stochastic semi-supervised learning" approach[1]. That approach is fundamentally different to mantis-ml with respect to the target of the problem, solution architecture and end results.

Specifically, Xie et al.'s approach always begins with a single positive data point and augments either with a single negative data point (k-means clustering method) or a random subset of 20 unlabelled data points (logistic regression method). In each iteration, more unlabelled data points are added and each of them assigned to the positive or negative class (through clustering or supervised learning, respectively). Eventually, each data point is assigned a class based on majority voting across all iterations. The top/bottom 1% are then labelled as "positive" / "negative" and a new model is trained to be ready for prediction on new unseen data. This is a method applied to datasets with an extremely rare set of positive labels. However, results can be easily prone to extreme overfitting initiated by the starting seed data point and then gradually mitigating through the rest of data points, as they are labelled "positive" or "negative" to inform the next stages of learning.

The architecture of our method is fundamentally different. Firstly, it's applied into a finite set of data points (all known human genes). Given this fixed annotation of human genes, no new unlabelled data can be added to the dataset. Furthermore, mantis-ml starts with a set of M positive data points (genes annotated from HPO, and usually M>>1) and treats the rest (N) as unlabelled. The goal is to rank all M+N data points based on the information of all (M) positive labelled points. Instead of providing a label of "positive" or "negative" in the end, mantis-ml aims to provide a propensity score (prediction probability score) for each gene which indicates the likelihood of association of each gene with the respective disease. The only similarity here is that the final propensity score is the average from multiple iterations which contain a random process. In Xie et al.'s, the randomness comes only from the sampling of unlabelled data points, as they are added in batches of 1 or 20 points, while in mantis-

ml it is derived by the random partitioning of the entire gene space (for L stochastic iterations) and then random split and learning over k-folds in each balanced dataset.

One of the most substantial differences is that our method uses the entire set of "positive"-labelled genes for supervised learning, which enables a holistic assessment of the relationships between all features across a wider set of seed data points. At the same time, in each random balanced dataset (positive/unlabelled ratio = 2:3), we use 80% of our original "positive"-labelled points to reduce overfitting on a single ground truth set thus adding an extra parameter of regularisation. Finally, the end goal of mantis-ml is not to produce a model that can be applied to new unseen data, but rather self-rank an originally highly imbalanced dataset with a finite number of points.

Finally, we employ well established supervised methods as part of the mantis-ml framework (implemented in scikit-learn and tensorflow/keras) which are used during training on the balanced datasets. Mantis-ml does not represent a novel general-purpose supervised learning algorithm but is instead a comprehensive framework for semi-supervised learning on highly imbalanced datasets in a finite space of data points.

Concordance of results between different classifiers

We employed a nominal decision probability threshold of 0.5 for assessment of the concordance of results across classifiers, as shown in Suppl. Figures 6,7 and 8 (c-d). This threshold is not used for decision making. We instead retain raw prediction probabilities for each gene to then focus on top hits of the final ranking rather than adopt a threshold segregation approach.

We first assessed the concordance between classifiers on the Chronic Kidney Disease example. 280 known genes (47.7% of all known genes) have been identified by all classifiers and another 64 genes (11% of all known genes) by at least five classifiers. In terms of the novel disease-associated genes, again the largest group of predicted genes-of-interest (n=1,300) has been predicted by all classifiers. However, each classifier calculates their predictions with a ranking score (prediction probability) which immediately provides a prioritisation scheme for the extracted gene predictions. Having no further knowledge to validate mantis-ml predictions at this stage, we choose to consider the gene rankings of the classifier with the highest average and individual AUC scores (XGBoost) in that case as the default mantis-ml prioritisation scheme for CKD (Suppl. File 2; Table S1). We found that the mantis-ml gene prediction rankings were significantly correlated when comparing XGBoost and Random Forests (Pearson's r = 0.976; p < 2.2 x 10-308, Suppl. Fig. 20), further demonstrating the robustness of the predictions beyond the choice of classifier.

Moving on to the mantis-ml performance on the Epilepsy example, we observe again all tree-based classifiers (XGBoost, Random Forest, Gradient Boosting and Extra Trees) performing best, with respective average AUC scores in descending order: 0.821, 0.818, 0.816 and 0.808 (Suppl. Fig. 7). We observe the preponderance of 360 known genes (41.7% of all known genes) being predicted by all seven classifiers followed by another 67 genes (7.7% of all known genes) predicted by at least six classifiers (based on prediction probability threshold of 0.5). Additionally, around 1,600 novel genes have been suggested by all classifiers. We provide as the default gene ranking for Epilepsy the mantis-ml predictions acquired when using XGBoost as the standard classifier during positive-unlabelled learning, based on its best AUC performance among all classifiers (Suppl. File 2; Table S2).

With regards to mantis-ml predictions on ALS (Suppl. Fig. 8), Extra Trees followed by XGBoost, SVC and Random Forest showed the best performance (average AUC scores: 0.814, 0.805, 0.801 and 0.798). Moreover, 31 known (40.1% of all known genes) and 1,500 novel genes were predicted by all classifiers (based on prediction probability threshold of 0.5). We also observe in this disease example that beginning with a smaller number of seed genes (n=77) is accompanied by a drop in average AUC scores. This suggests that mantis-ml performance has greater confidence from an increased presence of known genes. We provide the Extra Trees ranking scores as the default mantis-ml predictions for ALS (Suppl. File 2; Table S3).

Relative performance in different disease contexts

We found that the relative performance of the different supervised-learning models depended on the disease context as demonstrated in the results per disease at the mantis-ml gene prioritisation atlas (https://dvitsios.github.io/mantis-ml-predictions). However, in general the classifiers themselves performed relatively comparably.

For instance, SVC has performed better than Random Forest and most of the other tree-based methods in several occasions such as ALS, Alzheimer's, Autism and Pulmonary disease. Additionally, DNN performs better than ExtraTrees or other tree-based methods in Cardiovascular Disease, Pulmonary Disease and Respiratory Disease. In other cases, tree-based models perform relatively compared to DNN and SVC.

In general, we believe that the relative performance of classifiers largely depends on the disease under study each time, as it is captured by the number of known seed genes, any sparsity of relevant features (in which case SVC would probably perform better) or other parameters such as the genetic context of the disease and how well or confidently it is captured by the disease-specific set of features available in the databases we are using. At the same time, the difference in performance between classifiers is in most cases relatively small as it falls within the range of a 0.05-0.1 change in AUC.

Description of the Boruta algorithm

Boruta is a wrapper algorithm that can be used with any model that can calculate feature importance scores. It starts with creating random permutations of the original features (shadow features) which are then used along with the original ones by the learning model. In an iterative manner, the original features' contribution is compared against those from shadow features (Z-score difference) and only original features that exceed the max. importance of the best performing shadow feature are labelled as 'Confirmed' and discarded from future iterations. The ones that are below the min. importance of the worst performing shadow feature are 'Rejected'. The rest continue being tested until being 'Confirmed' or 'Rejected' or until the max. number of iterations has been reached. With regards to extracting the attribute importance, we calculate Z-scores of mean decrease accuracy, however using Gini impurity is also an option available in the Boruta algorithm and its R package implementation. Specifically, this can be achieved by setting *getImp="getImpExtraGini"* in the *Boruta()* S3 method instead of using the default *getImp="getImpExtraZ"* option.

Refined prioritisation using Stacking classifier

DNN is often adopted for extracting features of increasing complexity as one goes deeper into the network and the extracted "engineered"-features could then be fed into another model, e.g. XGBoost to perform the prediction task. This has primarily been a successful approach in scenarios where the input feature set is "unstructured", e.g. with imaging, text or sound data. In our learning task, the original feature set is well curated and "engineered" based on domain-specific knowledge aggregated in life sciences. Thus, the stacking classifier (enabled via "-s" option in *mantisml*) in our case serves not as a higher-level feature extractor but rather as an attempt to smooth out predictions from multiple classifiers and provide more filtered and refined results that have been validated by multiple models.

Feature set reduction and considerations for noise elimination

mantis-ml integrates over 1,200 features in total. For instance, many features refer to Gene Ontology annotations across various terms or some other features may reflect gene expression across different tissues. Only disease/phenotype-relevant terms or tissues are eventually selected for a disease of interest in an automatic manner, based on the disease/phenotype terms provided by the user in free text form. This means that eventually only 50-80 features are used during training for a particular disease/phenotype, dramatically reducing the risk of inclusion of noisy features from the original set of 1,200 features. We have also tried eliminating the noise from irrelevant features even further, by training each model using only the Boruta confirmed features. Performance in that case drops slightly across all classifiers, compared to when using all features (Suppl. Fig. 18). Thus, the feature set used during training (without feature-selection by Boruta) appears to be a non-redundant representation of the original features.

Sensitivity analysis for balancing ratio between positive and unlabelled data points

We explored the impact of selecting different balancing ratios between positive (P) and unlabelled (U) data points when forming random balanced datasets. We ran the Chronic Kidney Disease example for i=2 stochastic iterations using three different P/U balancing ratios: 1, 1.5 and 2, and calculated the correlations of gene probability predictions from the same classifier between each pair of runs (**Table S1**). We observe that correlations between balancing ratios 1 and 1.5 are in the range of 0.934-0.985 while for balancing ratios 1 and 2 are in the range of 0.874-0.974 (runs with balancing ratios 1.5 and 2 are even more similar with Pearson's r = 0.959-0.989). Since we want to create (as much as possible) balanced datasets and predictions with balancing ratio 1.5 are highly correlated with those with ratio=1 and even more than when using ratio=2, we choose 1.5 as the default value for balancing ratio.

The positive-unlabelled ratio is also provided as an advanced parameter which could be adjusted by an expert user based on needs. This is accessible at: "mantis-ml-release/mantis_ml/conf/.conf" as "balancing_ratio" parameter in the "supervised_filters".

Table S1. Pearson's r correlations between gene probability predictions extracted by each classifier for different values of positive-unlabelled balancing ratios (p-value < 2.2×10^{-308} for all comparisons).

Classifier	Compared balancing ratios: 1 vs 1.5	Compared balancing ratios: 1.5 vs 2	Compared balancing ratios: 1 vs 2
XGBoost	0.985	0.989	0.974
Random Forest	0.983	0.989	0.972

Gradient Boosting	0.934	0.959	0.874
Extra Trees	0.987	0.991	0.978
DNN	0.96	0.972	0.943
SVC	0.971	0.977	0.925

Sensitivity analysis for top mantis-ml predictions cut-off threshold during validation with independent studies

When performing an enrichment test of the mantis-ml predictions against independent gene rankings from external studies (e.g. WES rare-variant association studies), we define a certain cut-off for the top mantis-ml predictions to use. We have explored 10 different thresholds, from 1% to 10% (in increments of 1). We observed that the enrichment signal is consistently significant across all three disease examples (CKD, Epilepsy and ALS) when selecting a cut-off threshold ≥ 5% (**Figure S23 A and B**). We also observed that for cut-off thresholds > 5%, the hypergeometric enrichment saturates, however allowing for the extraction of larger numbers of novel genes. In general, selecting a certain cut-off threshold allows for the extraction of different numbers of prioritised genes eventually (**Figure S23 C & Figure S24**). For instance, in CKD, the 1-10% cut-off thresholds result to 4, 5, 8, 15, 21, 28, 31, 39, 47 and 50 gene hits, respectively (**Figure S24**), that are supported both by mantis-ml and the collapsing analysis. It is possible for the user to define more stringent or lenient cut-off thresholds when aiming to extract a more compact or expanded list of suggested prioritised genes to facilitate any further follow-up validations (the default value is set to 5%).

Interactive visualisation of novel gene predictions

We provide as part of the mantis-ml results, interactive versions of the PCA, t-SNE and UMAP plots (enabled via the "bokeh" library) in the form of ".html" files under the "[output_dir]/Output-Figures/unsupervised-learning" directory. These plots highlight all seed genes in red and the top 40 novel predictions (extracted by the classifier with the highest average AUC performance) in black. The user can zoom in any sub-region of each of these plots and inspect the names of predicted novel genes as well as any neighbouring known genes.

External module requirements and versions

Python3 (tested with v3.6.7)

- numpy: 1.14.5
- numpydoc: 0.8.0
- pandas: 0.24.2
- scipy: 1.2.1
- scikit-learn: 0.20.3
- bokeh: 1.1.0
- h5py: 2.9.0
- tensorflow: 1.10.0
- Keras: 2.2.4
- matplotlib: 3.0.3

- palettable: 3.1.1
- plotly: 3.9.0
- PyYAML: 5.1
- seaborn: 0.9.0
- tables: 3.5.1
- twine: 3.0.0
- tqdm: 4.14
- umap-learn: 0.3.8
- xgboost: 0.80

R (tested with v3.5.1)

- Boruta package (v6.0.0)

Data availability & pre-processing

- Generic Resources

ExAC

Exome Aggregation Consortium (ExAC) data are available at: http://exac.broadinstitute.org/downloads (last accessed on 06/03/2019). We integrate all data from CNV Counts and Intolerance Scores (‘*exac-final-cnv.gene.scores071316*’) and the ‘*GeneSize*’ feature from the Functional Gene Constraint Scores (‘*fordist_cleaned_exac_r03_march16_z_pli_ rec_null_data.txt*’).

Essential mouse genes

We integrate data from Georgi et al. (2013) that contain annotation for human orthologs of mouse genes that have been found to be essential for basic developmental functions and/or survival in both species (available at: https://doi.org/10.1371/journal.pgen.1003484.s022, last accessed on 06/03/2019). Both genes that have been identified as essential or non-essential are recorded and used as features by *mantis-ml*.

Genic-intolerance scores

We integrate two types of genic-intolerance scores: Residual Variation Intolerance Score (RVIS) and Missense Tolerance Score (MTR). RVIS scores (applied to EVS, ExAC and ExAC v2) are publicly available at http://genic-intolerance.org while MTR scores are publicly available at http://mtr-viewer.mdhs.unimelb.edu.au (both last accessed on 06/03/2019).

GnomAD

Genome Aggregation Database (GnomAD) data are publicly available at https://gnomad.broadinstitute.org/downloads (release 2.1, last accessed on 06/03/2019). We integrate all gene constraint scores in the *mantis-ml* framework. We retain for each gene all associated constraint scores that correspond to the canonical transcript, choosing the longest one in case there are more than one canonical transcript annotated for a gene. Aforementioned ExAC is a subset of GnomAD; however, both versions of the associated constraint scores are adopted.

GWAS (used both in the generic and disease-specific models)

Genome Wide Association (GWAS) data are publicly available at: https://www.ebi.ac.uk/gwas/docs/file-downloads (last accessed on 06/03/2019). We integrate data from ‘All associations’ (v1.0.2). For the disease-specific model we select all entries that contain any

of the '*Disease/Phenotype terms*' and '*Additional associated terms*' and do not contain any of the '*Diseases/Phenotypes to exclude*' from *config.yaml*. For the generic model we include all entries. In both cases, however, we filter out any entry with a p-value over the genome-wide significance threshold (p-value threshold: $5x10^{-8}$). Then, both for the disease-specific and generic model, we assign a True boolean flag to every gene that has at least one GWAS hit for any of the query terms specified. We also record the total number of GWAS hits per gene as well as the min/max p-values and min/max Odds Ratios associated with each gene.

MGI (generic)
Mouse Genome Informatics (MGI) data are publicly available at: http://www.informatics.jax.org/downloads/reports/index.html (last accessed on 06/03/2019). We are integrating data from three files: Genotypes and Mammalian Phenotype Annotations for Marker Type Genes excluding conditional mutations ('*MGI_GenePheno.rpt*'), Mouse/Human Orthology with Phenotype Annotations ('*HMD_HumanPhenotype.rpt*') and Mammalian Phenotype Vocabulary in OBO v1.2, tab-delimited and OWL Formats ('*VOC_MammalianPhenotype.rpt*'). We combine all data from these files to link human with mouse orthologs and their associated high-level mammalian phenotype descriptions and IDs. Gene labelling for this feature is performed by string matching of the '*Disease/Phenotype terms*' and '*Additional associated terms*' from the given *config.yaml* file with the 'High-level Mammalian Phenotype ID' field in *hmd_human_pheno.processed.rpt*. Finally, we also annotate all genes that are associated with a 'Lethal' phenotype with a True boolean flag. The MP IDs associated with a 'Lethal' phenotype are: 0002058, 0002080, 0002081, 0002082, 0002083, 0006204, 0006205, 0006206, 0006207, 0006208, 0008527, 0008569, 0008762, 0009850, 0010768, 0010769, 0010770, 0010831, 0010832, 0011083, 0011084, 0011085, 0011086, 0011087, 0011088, 0011089, 0011090, 0011091, 0011092, 0011093, 0011094, 0011095, 0011096, 0011097, 0011098, 0011099, 0011100, 0011101, 0011102, 0011103, 0011104, 0011105, 0011106, 0011107, 0011108, 0011109, 0011110, 0011111, 0011112, 0011400, 0013292, 0013293, 0013294.

- Resources filtered by tissue/disease
GTEx
Genotype-Tissue Expression (GTEx) data are publicly available at: https://gtexportal.org/home/datasets (V7, last accessed on 06/03/2019). We integrate RNA-Seq data that contain the median TPM expression values by tissue ('*GTEx_Analysis_2016-01-15_v7_RNASeQCv1.1.8_gene_median_tpm.gct*', last accessed on 06/03/2019). For the tissue-specific model case, we subset the GTEx tissues that match any of the strings defined in the '*Disease/Phenotype terms*' and '*Additional associated terms*' fields in *config.yaml* and then aggregate all values by gene across all tissues. Additionally, we assign a rank for each gene based on the aggregate expression across all matching tissues (ranks = {1, 2, 3, …} in order of decreasing expression). Genes with overall expression less than the median among all genes are assigned the same rank, equal to the total number of genes, to increase signal-to-noise ratio for the most highly-expressed genes. As for the disease-generic model case, we keep expression values across all tissues and retain them for each gene as separate features, while no rank is computed in that case.

Human Phenotype Ontology
The Human Phenotype Ontology (HPO) data are publicly available at: http://www.human-phenotype-ontology.org. We are using Build #154 from HPO to annotate disease-associated genes (last accessed on 04/03/2019). We are using by default the '*ALL_SOURCES_FREQUENT_FEATURES_\ genes_to_phenotype.txt*' file (provided by the HPO consortium), as our reference annotation file to exclude phenotypic features that are observed occasionally (present in 5–29% of the cases), rarely

(present in 1–4% of the cases) or not at all (present in 0% of the cases). Annotation is performed by selecting genes whose '*HPO-Term-Name*' contains any of the '*Disease/Phenotype terms*' and does not contain any of the 'Diseases/Phenotypes to exclude' from the *config.yaml* configuration file.

Human Protein Atlas
Human Protein Atlas data are publicly available at: https://www.proteinatlas.org/about/download (version 18.1, last accessed on 06/03/2019). We integrate two types of data from Human Protein Atlas: Normal tissue data (*normal_tissue.tsv*), which contain levels of expression for each gene in different tissues and cell types (categorical variable: 'Not detected', 'Low', 'Medium', 'High') and RNA gene data (*rna_tissue.tsv*), which contain TPM expression values for each gene by Sample (where '*Sample*' in this case is similar with the '*Tissue*' field from Normal tissue data).
We initially filter out all entries that have an 'Uncertain' value in the 'Reliability' field. For the disease-specific model case, we select all genes which contain any of the strings from '*Disease/Phenotype terms*' and '*Additional associated terms*' and do not contain any of the '*Diseases/Phenotypes to exclude*' from *config.yaml* file in their "Tissue/Sample" fields for Normal tissue and RNA gene data, respectively. Normal tissue data contain in general multiple values (levels of expression) per gene for the different cell types under each tissue type. We collapse all values for each gene by selecting the highest level found in a cell type within each tissue ('Not detected' < 'Low' < 'Medium' < 'High'). With regards to RNA gene data, we aggregate all TPM values for each gene.
As for the generic disease model, expression levels from Normal tissue data are retrieved across all tissues, the highest level is retained for each gene and eventually we convert the four original levels into two: 'Not detected' and 'Low' are both considered as 'Low' and 'Medium' and 'High' are both considered as 'High'. This transformation is performed to increase signal-to-noise ratio on this feature when looking at expression across all tissues. Finally, RNA gene data are aggregated by gene for each Sample.

InWeb_IM
InWeb_IM data (human protein-protein interaction network data, Li et al. 2017) are publicly available at: https://www.intomics.com/inbio/map.html#downloads ('*inBio_Map_core_2016_09_12.zip*', last accessed on 06/03/2019). Protein-protein interactions are characterised as 'inferred' or 'experimental' based on the validation degree recorded for each interaction in the original analysis. We untangle all interacting genes for each gene by validation type ('inferred' or 'experimental') and during analysis we record the ratio of interacting genes that belong to the seed genes (positively labelled genes) in each disease-specific run.

MGI
Data compilation is performed as described at the 'MGI' section in 'Generic Resources'. For the disease-specific model, annotation is performed by selecting all genes whose linked phenotypes contain any of the strings from '*Disease/Phenotype terms*' and '*Additional associated terms*' and do not contain any of the '*Diseases/Phenotypes to exclude*' from *config.yaml*.

MSigDB
Molecular Signatures Database (MSigDB) data are publicly available at: http://software.broadinstitute.org/gsea/downloads.jsp (v6.2, last accessed on 06/03/2019). We integrate data from the c5 gene set (gene ontology sets). For the disease-specific model case, we select all gene ontology terms that contain any of the strings from '*Disease/Phenotype terms*' and '*Additional associated terms*' and do not contain any of the '*Diseases/Phenotypes to exclude*' from *config.yaml*. As for the disease-generic model, we retain all gene ontology terms. In both cases, gene ontology terms with less than 150 associated genes (0.08% of all genes) are filtered out to reduce

the number of features with near-zero variance. In the current dataset this leaves 1,009 of 5,917 gene ontology terms.

OMIM
Online Mendelian Inheritance in Man (OMIM) data are available under licensing at: https://www.omim.org (‘*genemap2.txt*’, last accessed on 06/03/2019). We have restricted OMIM data to the subset of entries where the field ‘*Phenotypes*’ contains ‘*(3)*’, which reflects entries where the ‘*molecular basis for the disorder is known; a mutation has been found in the gene*’. OMIM annotation data are used only for extracting a disease-generic gene ranking. By default, all genes which contain ‘*(3)*’ in their ‘*Phenotypes*’ field are annotated as disease-associated genes (value ‘*All*’ in ‘*generic_classifier*’ parameter in the mantis_ml/conf/*.config* file). Additional filtered layers of positive gene data are available by specifying different values for the ‘*generic_classifier*’ in mantis_ml/conf/*.config*: a) ‘*AD*’ for selecting only genes that include ‘*Autosomal dominant*’ annotation in their ‘*Phenotypes*’ field, b) ‘*AR*’ for selecting only genes that include ‘*Autosomal recessive*’ annotation in their ‘*Phenotypes*’ field, c) ‘*AD_only*’ for selecting only genes that include ‘*Autosomal dominant*’ annotation and at the same time do not contain ‘*Autosomal recessive’* annotation in their ‘Phenotypes’ field, d)) ‘*AR_only*’ for selecting only genes that include ‘*Autosomal recessive’* annotation and at the same time do not contain ‘*Autosomal dominant’* annotation in their ‘*Phenotypes*’ field.

All string-matching operations are case insensitive.

- Disease-specific Resources (currently supported)

i. **Chronic Kidney Disease (CKD):**

CKDdb
Data from the Chronic Kidney Disease database (CKDdb) are available at: http://www.padb.org/ckddb (last accessed on 08/03/2019). We annotate each gene that has been associated with a renal disease with a True boolean flag and also record the total number of studies in CKDdb that support this evidence.

nephQTL
eQTL data for the glomerular and tubulointerstitial tissues (NephQTL) are available at: http://nephqtl.org. This database contains *cis*-eQTLs of the glomerular and tubulointerstitial tissues of the kidney found in 187 participants in the NEPTUNE cohort. For each gene and tissue, we record the expected number of eQTLs, the probability of not having eQTLs and the False Discovery Rate (FDR).

ii. **Cardiovascular Disease:**

exSNP
Data from the database of expression associated SNPs (exSNP) are available at: http://www.exsnp.org/Download (last accessed on 08/03/2019). We are integrating disease associated high confidence ($r^2 > 0.8$) eQTLs for Coronary Artery Disease and Hypertension and record the total number of eQTLs associated for each gene in each condition.

Adipose eQTLs
Data for adipose eQTLs identified at GWAS loci for cardiometabolic diseases and traits were retrieved from Civelek et. al, 2017 (Table S8). We record for each gene the total number of GWAS loci and cis eQTLs that have been associated for cardiometabolic traits.

Platelet eQTLs

Data for platelet eQTLs were retrieved from Simon et. al, 2016 (Table S2). Platelets have been shown to contribute to ischemic cardiovascular events[2]. We record for each gene the total number of heterozygous coding sites with a marginal eQTL effect ($p < 10^{-4}$) and with 10 or more reads.

Reference gene set

We have specified as our reference gene set all genes annotated by the ExAC and gnomAD (v2) consortia (n=18,626) to include all genes with sufficient evidence and annotations, to avoid contaminating our dataset with imputed features for a large subset of genes.

Assessment of mantis-ml performance with/without CKDdb-derived features

CKDdb is a resource that captures information associated with CKD by mining existing literature followed-up by manual curation. That means that it integrates various types of putative disease annotations at the gene level, however, having highly variable degrees of confidence in terms of their true relevance with the disease. Thus, true biological signal in this resource is contaminated with noise from annotations that have little support (e.g. mentioned in a single publication without any experimental validation) and does not directly represent confirmed associations of a gene with CKD (this is extracted from HPO instead). Apart from that, when we look at the feature importance during mantis-ml training for CKD, we observe that the features extracted from CKDdb are not contributing significantly to the final predictions. Specifically, the feature that indicates the presence/absence of a gene in CKDdb (CKDdb_Disease) is classified as 'rejected' by the Boruta algorithm, with regards to its feature importance (**Figure S9 A**). Similarly, the number of studies associated per gene ("CKDdb_num_of_studies") is annotated as "tentative"/"inconclusive" (**Figure S9 A**). That indicates that none of the CKDdb-extracted features contributes significantly to the predictions, again eliminating the risk of contamination of the training set with information directly associated with the output label.

Furthermore, we also trained mantis-ml without using CKDdb-derived features. We observe that mantis-ml performance with/without CKDdb-derived performance remains practically unchanged, with a very minor improvement when using the CKDdb-derived features (**Figure 24 A & B**; AUC: 0.829-0.846 versus 0.823-0.839). Similarly, feature importance analysis between the two cases highlights the same sets of features as the most contributing ones (**Figure 24 C**). Finally, the hypergeometric enrichment against the collapsing analysis hits (without using CKDdb-based features) remains highly significant, again demonstrating that mantis-ml's performance on CKD is not primarily driven by the CKDdb annotation.

Supplemental References

1. Xie, J. Stochastic Semi-supervised Learning on Partially Labeled Imbalanced Data. *Area* (2011).

2. Simon, L. M. *et al.* Integrative Multi-omic Analysis of Human Platelet eQTLs Reveals Alternative Start Site in Mitofusin 2. *Am. J. Hum. Genet.* **98**, 883–897 (2016).
