# Supplementary material for: Mantis-ml: Disease-Agnostic Gene Prioritization from High-Throughput Genomic Screens by Stochastic Semi-supervised Learning
Source: Am J Hum Genet. 2020 May 7;106(5):659–78. doi: 10.1016/j.ajhg.2020.03.012 (PMC7212270; doi:10.1016/j.ajhg.2020.03.012)
Supplement: Document S2. Article plus Supplemental Information [file mmc3.pdf]

Mantis-ml: Disease-Agnostic Gene Prioritization from High-Throughput Genomic Screens by Stochastic Semi-supervised Learning

Dimitrios Vitsios[1,*] and Slavé Petrovski[1,*]

Access to large-scale genomics datasets has increased the utility of hypothesis-free genome-wide analyses. However, gene signals are often insufficiently powered to reach experiment-wide significance, triggering a process of laborious triaging of genomic-association-study results. We introduce mantis-ml, a multi-dimensional, multi-step machine-learning framework that allows objective assessment of the biological relevance of genes to disease studies. Mantis-ml is an automated machine-learning framework that follows a multi-model approach of stochastic semi-supervised learning to rank disease-associated genes through iterative learning sessions on random balanced datasets across the protein-coding exome. When applied to a range of human diseases, including chronic kidney disease (CKD), epilepsy, and amyotrophic lateral sclerosis (ALS), mantis-ml achieved an average area under curve (AUC) prediction performance of 0.81–0.89. Critically, to prove its value as a tool that can be used to interpret exome-wide association studies, we overlapped mantis-ml predictions with data from published cohort-level association studies. We found a statistically significant enrichment of high mantis-ml predictions among the highest-ranked genes from hypothesis-free cohort-level statistics, indicating a substantial improvement over the performance of current state-of-the-art methods and pointing to the capture of true prioritization signals for disease-associated genes. Finally, we introduce a generic mantis-ml score (GMS) trained with over 1,200 features as a generic-disease-likelihood estimator, outperforming published gene-level scores. In addition to our tool, we provide a gene prioritization atlas that includes mantis-ml's predictions across ten disease areas and empowers researchers to interactively navigate through the gene-triaging framework. Mantis-ml is an intuitive tool that supports the objective triaging of large-scale genomic discovery studies and enhances our understanding of complex genotype-phenotype associations.

Introduction

As a result of the vast interrogation of the protein-coding genome, the global research community has generated an extended amount of resources related to tissue-specific gene expression, intolerance to genetic variation, model organism function, and various other diverse annotation types. Additionally, it is evident that complex phenotypes, such as disease phenotypes, cannot be explained by the variability of a single data type (e.g., expression in tissue or animal models) but rather require the combination of a multitude of data types and resources that describe multiple aspects of the phenotype at different dimensions.[1–3]

The underlying biology of human disease is complex, and current knowledge provides a limited view of the full collection of disease-associated genes. We sought to explore this issue by leveraging the rich collection of well-curated gene-level annotations to identify patterns that are shared among genes associated with a disease and leverage those patterns to predict putatively novel genes of interest that have the most similar profiles and, thus, might also be associated with disease.

To achieve this goal, for each gene we harvested diverse types of information, including gene expression;[4] human disease literature;[5] mouse phenotypes;[6] proteomic;[7] interactome;[8] and genic metrics of human-lineage purifying selection[9–11] (see Supplemental Methods). Next, we developed mantis-ml, a machine-learning framework that can be applied to any disorder, given a starting set of genes that are associated with disease according to the Human Phenotype Ontology (HPO).[5] Mantis-ml's gene predictions without HPO-based annotations for the disease of interest are characterized as 'novel' throughout this work. The mantis-ml framework is based on a stochastic semi-supervised learning approach that solves inherent challenges presented by the problem of the high class imbalance in a finite space of data points (see Supplemental Methods).

Unlike other published gene-prioritization methods, we provide validation of our method's predictions against results from real cohort genetic studies. The cohort statistics from these analyses refer to gene-level statistics emerging from rare-variant genetic-association studies.[12] Specifically, we apply the predictions from our tool, mantis-ml, to the results of published exome-wide-association statistics and show a striking preferential enrichment of mantis-ml-predicted genes among the genes achieving the lowest p values in the respective case-control studies of those diseases. The three diverse disorders that we highlight as applications of mantis-ml are amyotrophic lateral sclerosis (ALS),[13] chronic kidney disease (CKD),[14] and epilepsy.[15]

Methods

Feature Pre-processing

Mantis-ml integrates gene-associated features from a diverse pool of gene-annotation sources, classified into three categories: generic

[1]Centre for Genomics Research, Discovery Sciences, BioPharmaceuticals R&D, AstraZeneca, 1 Francis Crick Avenue, CB2 0RE Cambridge, UK
*Correspondence: dimitrios.vitsios@astrazeneca.com (D.V.), slav.petrovski@astrazeneca.com (S.P.)
https://doi.org/10.1016/j.ajhg.2020.03.012.
© 2020 The Author(s). This is an open access article under the CC BY license (http://creativecommons.org/licenses/by/4.0/).

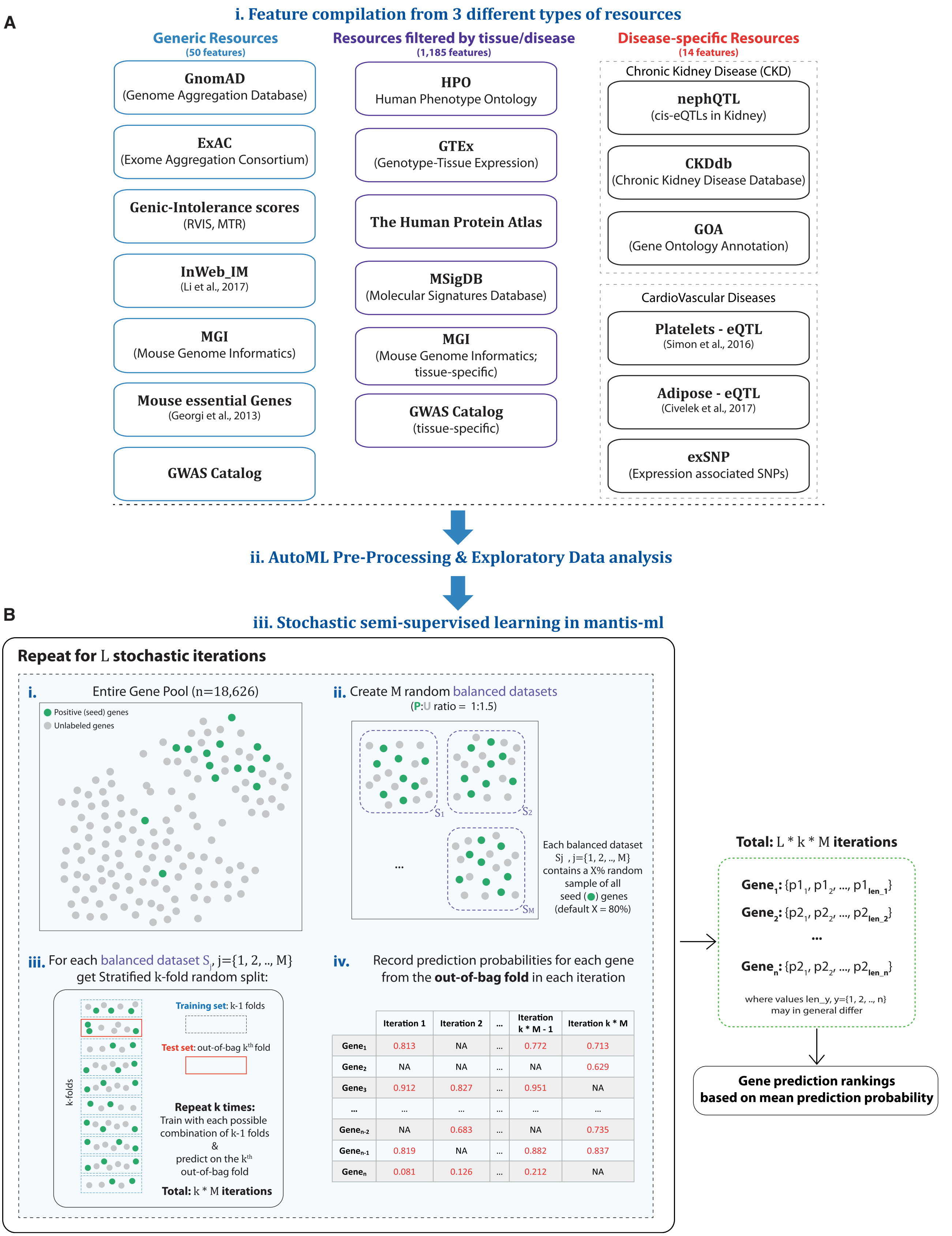

Figure 1. Generic Overview of Mantis-ml Workflow

(A) Data resources used by mantis-ml for feature extraction. Three data-type resources are integrated: generic (i.e., non-tissue- and non-disease-specific), filtered by disease or tissue, and filtered by disease-specific features (currently including disease-specific features for CKD

(legend continued on next page)

resources (disease and/or tissue agnostic), resources filtered by tissue, and disease-specific features (Figure 1A and Table S1). mantis-ml performs automatic feature pre-processing, which includes filtering of highly correlated features on the basis of a Pearson's r correlation threshold (parameter "eda_parameters -> high_corr_thres" in mantis_ml/conf/.config; default value: 0.8). Additionally, features with more than a certain amount of missing data are discarded (parameter "eda_parameters -> missing_data_thres" in mantis_ml/conf/.config; default value: 0.25). The remaining features with a missing-data ratio below the cut-off threshold are imputed with either a zero value or the median of the respective feature. Imputation with zero is performed either because most of the genes represent a binary flag ("non-existent" or 0 for missing data, e.g., "MGI_mouse_knockout_feature," "GOA_Kidney_Research_Priority," etc.) or because these features were extracted from computational or experimental studies that retrieve a biologically relevant signal only from a specific set of genes that are associated with the hypothesis under examination (e.g., "platelets_eQTL," "adipose_GWAS_locus" features, etc.). The features that are imputed with a median value are all genome-wide association study (GWAS) metrics, ExAC CNV-associated features, the residual variation intolerance score (RVIS), the missense tolerance ratio (MTR), and gene length. The selection of the median value for imputation in that case is based on the use of different global reference sets of genes for different studies or resources. This difference requires extrapolation of these features to genes with missing values because penalizing them with a zero value would most likely not be representative of the actual gene behavior with respect to these features. Finally, features are standardized to have mean zero value and unit variance.

Exploratory Data Analysis

Mantis-ml generates an extended set of visualizations, including heatmaps for pairwise feature correlations (prior and post feature filtering), missing-data ratios across features, and the distribution of numerical and/or categorical variables across the known and unlabeled genes from the entire gene pool (Figures S2 and S3). Additionally, mantis-ml automatically performs dimensionality reduction on the original feature set via principal component analysis (PCA), t-distributed stochastic neighboring embedding[16] (t-SNE), and uniform manifold approximation and projection[17] (UMAP) to allow for visualization of the original high-dimensional space in two dimensions (Figure S4).

Unlike PCA, t-SNE and UMAP are both non-linear projections of a high-dimensional space into a lower-dimensional space. They both compute probability distributions regarding the relationships between the points in the high-dimensional space but use different similarity kernels: Gaussian for t-SNE and non-Gaussian for UMAP. The computed similarities are then recreated in the lower-dimensional space (embedding space) with a Student's t distribution for t-SNE and a kernel proximal to t distribution for UMAP. The main practical difference between these algorithms is that t-SNE can capture local relationships well in the embedding space but does not preserve the global structure, whereas UMAP can preserve both the local and global structure in the lower dimensional space.

PCA is accompanied by a Scree plot summarizing the cumulative variance explained by the first 20 principal components. Because PCA can only capture linear relationships between the original vectors, the variance explained by the top principal components provides an insight into the prevalence of linear versus non-linear relationships between features in the original space. t-SNE is calculated with a default perplexity value of 30, whereas UMAP is run with the following default parameter values: "n_neighbors" = 5, "min_dist" = 0.3, "metric" = "correlation". Finally, all two-dimensional projected feature visualizations are provided both as static files (PDF format) and interactive visualizations (HTML format).

These visualizations aim to highlight any evident and/or trivial segregation of the known disease-associated genes from the unlabeled genes on the basis of either pairwise relationships between the features or any linear and/or non-linear relationships captured by the top projected vectors of the respective transformation spaces. However, the complexity underlying each of the studied diseases imposes the exploration of high-dimensional interactions between all features to elucidate the complex mechanisms that primarily drive pathogenicity in each case. We thus resort to machine-learning techniques to tackle this problem.

Stochastic Positive-Unlabeled Prediction with Standard Classifier and Benchmarking

Mantis-ml seeks to uncover any feature patterns among a collection of known positive-labeled disease-associated genes to then prioritize novel genes that share a highly similar feature profile with the known disease-associated genes. This problem falls into the broader machine-learning area of positive-unlabeled learning, a semi-supervised learning technique where the only labeled data points available are positive.

This is important in this context because we often have insufficient information about which genes among the remainder of the genome are definitively not associated with that disease (i.e., true negatives). There are several approaches aiming to solve positive-unlabeled problems, the most popular of which are (a) to treat unlabeled data as negative and perform learning with a standard classifier,[18] (b) to use bootstrap and bagging to iteratively train on random samples of positive and unlabeled data and make predictions on the basis of out-of-bag unlabeled data,[19] and (c) to use two-step approaches in which the first step tries to identify a confident set of negative points among the unlabeled set and then continues learning with a standard classifier.[20]

Here, we developed a gene-prioritization framework that is based on a variation of two of the positive-unlabeled approaches suggested above (a and b): a stochastic semi-supervised learning technique that is performed across multiple random balanced datasets from the entire gene set (L iterations over random partitionings of the entire gene space) and makes iterative predictions on out-of-bag data.

and cardiovascular disease). All features are compiled automatically on the basis of user-provided disease-associated query terms and pre-processed so they are ready to be provided as input to the supervised/un-supervised learning tasks. of mantis-ml.
(B) Illustration of the stochastic semi-supervised approach followed by mantis-ml over L iterations: (1) positive (seed) genes are annotated using the HPO (static for each stochastic iteration), (2) the entire gene pool is split into random balanced sets, each of which includes a random sample (default: 80%) of seed genes, (3) each balanced dataset is split into a stratified k number of folds, training is performed for each combination of $k - 1$ folds, and prediction is subsequently based on the kth out-of-bag fold each time, and (4) prediction probabilities are aggregated for each gene across all $L \times k \times M$ iterations.

The input data for mantis-ml are all coding genes, labeled on the basis of known or unknown annotation for a disease and accompanied by a large set of gene-level features extracted from public databases. We tested seven different classifiers to be used during positive-unlabeled learning for each balanced dataset of positive and unlabeled data points. These seven classifiers were random forest, extra trees (extremely randomized trees—a variation of random forest), gradient boosting, extreme gradient boosting (XGBoost), support vector classifier (SVC), deep neural networks (DNN), and a stacking (ensemble) classifier with four base classifiers (random forest, extra trees, gradient boosting, and SVC) followed by a DNN in the second layer.

We first fine-tuned each classifier separately by using two random balanced datasets from the CKD disease example with 10-fold cross-validation and performing grid search over a finite parameter space. We then benchmarked all classifiers by assessing their "area under curve" (AUC) performance on the same set of ten random balanced datasets with 10-fold cross-validation. All classifiers performed comparably (average AUC: 0.831–0.850), and random forest and extreme gradient boosting ranked as the top two classifiers, with mean AUCs equal to 0.850 ± 0.021 and 0.848 ± 0.021, respectively (Figures 2 and S5). Given the comparable performance across classifiers, we do not pick a single classifier that outperforms the rest in the problem of gene prioritization with positive-unlabeled learning. Thus, we apply all classifiers to each disease example examined in this work and then select the best performing classifier in each disease example on the basis of the average AUC scores achieved.

Eventually, we have scaled up the positive-unlabeled learning task to the entire gene space, which is covered by a random partitioning of the unlabeled genes in combination with a random subset of the positive (seed) genes each time. We extract the final ranking by averaging the prediction probabilities assigned to each gene from all the generated out-of-bag sets. This approach allows genes to compete with each other in a stochastic semi-supervised manner and self-sort because their respective features can capture enough of the variance of truly disease-informative characteristics.

Selection of Optimal Classifier Parameters via Grid-Search Cross-Validation

Fine-tuning for all "scikit-learn"-based classifiers (random forest, extra trees, gradient boosting, and SVC) and for XGBoost was performed over a pre-defined finite parameter grid space with GridSearchCV from "scikit-learn's" "model_selection" module and tested on two random balanced datasets from the CKD disease example (Table 1). The available kernel options that were tested with SVC were "linear," "poly," "rbf," and "sigmoid".

With regards to *keras*-based DNNs (comprised of feed-forward fully connected layers), we developed a module that performs grid search with cross-validation (*dnn_grid_search_cv.py*), including tuning of parameters such as size and number of hidden layers. This module currently supports simultaneous fine-tuning of up to two features but can otherwise fine-tune any DNN-related parameters in a single run with sequential steps of optimization that progressively select near-optimal features in a heuristic manner. "ReLU" has been used as the activation function across all hidden nodes, while "softmax" was used in the output layer. Additionally, seven different optimizers where tested as part of the grid search: "SGD," "RMSprop," "Adagrad," "Adadelta," "Adam," "Adamax," and "Nadam." The optimal parameters returned by grid search with cross-validation for each classifier are available in Table 1.

Selection of the Optimal Number of Stochastic Iterations in Positive-Unlabeled Learning

We examined the number of known and novel genes predicted for different numbers of stochastic iterations of positive-unlabeled learning by using an "extra trees" classifier on a disease-specific example (Figure S19). We observed that the number of predicted known genes is practically insensitive to the number of stochastic iterations. However, the number of novel genes requires a certain number of iterations until it reaches a stable state, which is around 1,000 genes. Specifically, the novel-gene count enters an oscillation zone around the stable state after $L = 10$ iterations, which is then further stabilized after $L = 100$ iterations.

In more rigorous testing, we assessed the correlation of mantis-ml average prediction probabilities when run for different number of stochastic iterations. Ideally, a robust algorithm should capture the same average profile for each gene irrespective of the number of iterations mantis-ml has been trained on. Specifically, we ran mantis-ml for the following numbers of stochastic iterations: 1, 10, 30, 50, 70, 100, 150, and 200. Pearson's r correlation of the average mantis-ml prediction probabilities extracted from just one iteration compared to all other numbers of iterations is always >0.984 ($p < 2.2 \times 10^{-308}$). For any other pair of stochastic iterations with $L > 1$, Pearson's correlations are in the range of 0.9976–0.999 ($p < 2.2 \times 10^{-308}$). These predictions demonstrate the robustness of mantis-ml predictions irrespective of the number of stochastic iterations used. Thus, we suggest using $L = 10$ iterations by default for a run on a disease-specific case. The user can further adjust this through the "*-i*" parameter when running the mantis-ml tool.

Application of Mantis-ml on Disease Examples

We applied all seven classifiers used during benchmarking across $L = 10$ stochastic iterations for each disease-specific positive-unlabeled learning task. No selection of positive-labeled genes, tissue, and disease-relevant features requires human curation beyond provision of the user-defined disease-associated inclusion and exclusion terms in the input config file. The total number of training-test tasks performed across an equivalent number of random balanced gene samples with cross-validation was 25,000, 17,000, and 79,500 for CKD, epilepsy, and ALS, respectively. These sizes are inversely proportional to the number of seed genes in each case, and this number directly affects the size of constructed balanced datasets across the entire gene pool.

All classifiers except for stacking showed comparable performance when applied to the entire gene set for each disease case. The stacking classifier consistently demonstrated slightly lower performance than the rest of the classifiers (on average 0.05 lower AUC score). This is somewhat expected because one of stacking's most notable properties is to smooth out predictions from its base classifiers. This means that predictions supported by most of its base classifiers are more likely to survive in the end, thus potentially lowering the total number of correctly identified genes, which might however be more robust than the predictions from each individual classifier as a result of its conservative nature.

Dictionary of Inclusion and Exclusion Query Terms for the Studied Disease Examples

Mantis-ml requires as input a YAML ain't markup language (YAML) config file containing information about the diseases/

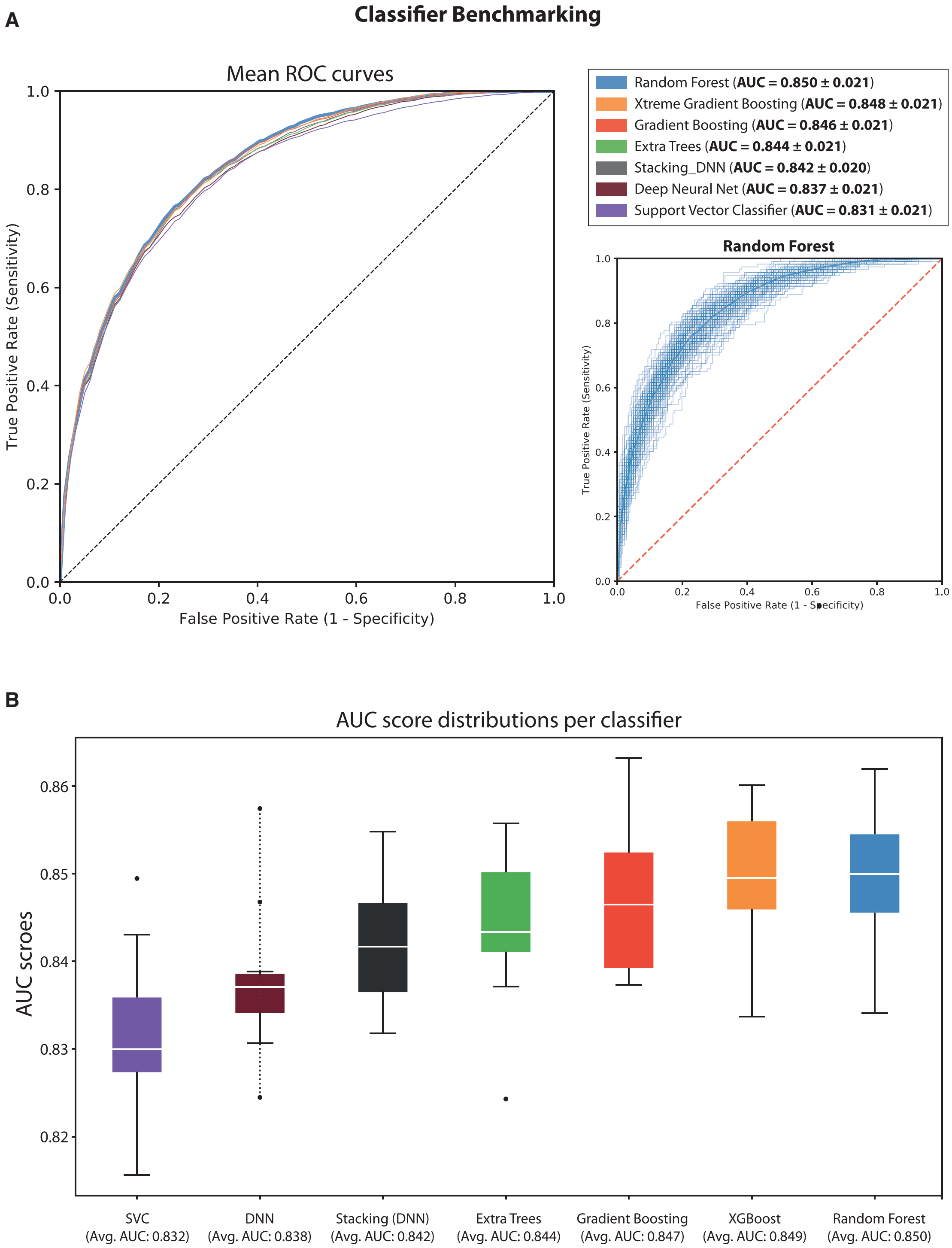

Figure 2. Mantis-ml Classification Performance Benchmarking Using Different Supervised Models
Benchmarking of Seven Different Classifiers during the Positive-Unlabeled Learning Step of mantis-ml: Random Forest, Extra Trees, Gradient Boosting, Extreme Gradient Boosting (XGBoost), Support Vector Classifier (SVC), Deep Neural Network (DNN), and a Stacking (Ensemble) Classifier with Four Base Classifiers (Random Forest, Extra Trees, Gradient Boosting, and SVC) followed by a DNN Mantis-ml was run on ten random balanced datasets with 10-fold cross-validation based on the CKD example.
(A) Mean receiver operating characteristic (ROC) curves from stochastic positive-unlabeled learning with one of the seven classifiers. ROC curves from all runs are also shown for the best performing classifier during benchmarking (random forest).
(B) Distribution of AUC scores across the seven classifiers tested. All classifiers showed comparable performance (AUC: 0.83–0.85), and tree-based methods ranked on the top.

Table 1. Optimal Parameters for Each Classifier Calculated with Grid Search and 10-fold Cross-Validation

Parameters Selected with Grid Search
**Deep neural network (DNN)**
hidden layers: 2
nodes per layer: [32, 32]
dropout ratio: 0.3
L2 regularization parameter: 0.01
optimizer: 'Adagrad'
epochs: 50
batch_size: 128
activation function: "ReLU"
**Extreme gradient boosting (XGBoost)**
learning_rate: 0.01
n_estimators: 300
max_depth: 5
min_child_weight: 3
gamma: 0
subsample: 0.8
colsample_bytree: 0.8
objective: "binary:logistic"
scale_pos_weight: 1
**Extra trees**
n_estimators: 100
max_features: "auto"
max_depth: 15
min_samples_leaf: 2
min_samples_split: 5
**Gradient boosting**
n_estimators: 500
max_features: "sqrt"
max_depth: 20
min_samples_leaf: 4
min_samples_split: 5
**Random forest**
n_estimators: 100
max_features: "auto"
max_depth: 15
min_samples_leaf: 2
min_samples_split: 4
warm_start: false
**Support vector classifier (SVC)**
C: 0.01
kernel: "linear"
gamma: "auto"
probability: true
shrinking: true

phenotypes of interest. One field in the config file is required ("disease/phenotype terms"), whereas another two fields are optional ("additional associated terms" and "diseases/phenotypes to exclude"). The descriptions for these fields are as follows:

- "disease/phenotype terms"- terms that characterize a phenotype or disease of interest and that are used for known disease-associated gene selection and filtering of relevant features (free text), required
- "additional associated terms"- terms used along with "disease/phenotype" terms to extract additional disease- or phenotype-associated features (free text), optional
- "diseases/phenotypes to exclude"- terms to exclude from disease or phenotype characterization and feature selection (free text), optional

The terms provided in these fields for each of the diseases under study (CKD, epilepsy, and ALS) are available in Table 2.

Estimating Feature Importance with the Boruta Algorithm

The Boruta algorithm was run on top of a "random forest" classifier trained on 100 random balanced datasets with 10-fold cross-validation and was run internally for 100 iterations. Boruta assesses the importance of each feature by comparing its contribution with the ones from random permuted features and eventually provides Z scores that quantify the distance from these comparisons. Upon each Boruta training cycle on a random balanced dataset, features are characterized as "confirmed," "tentative," or "rejected," and the full distribution of Z scores is provided for each of them. Because of the stochastic nature of the positive-unlabeled learning implemented by mantis-ml, features can be characterized by different labels in different runs of the Boruta algorithm. We have thus defined a decision threshold to classify each feature as "confirmed," "tentative," or "rejected" on the basis of its extracted labels across all Boruta runs. Specifically, for CKD and epilepsy, features are eventually classified as "confirmed" when they receive this label across at least 90% of all Boruta runs. The decision threshold for the "confirmed" feature classification has been set to 60% for ALS to compensate for the higher variance of extracted feature importance labels across all iterations; this higher variance is most likely due to the smaller set of seed genes in that case. In all three cases, features labeled as "rejected" are eventually classified as such in at least 90% of the cases, whereas the remaining features are characterized as "tentative" on the consensus labeling.

We also added an option for mantis-ml to be trained with only the set of "confirmed" features extracted by the Boruta algorithm (parameter "supervised_filters -> feature_selection: boruta" in mantis_ml/conf/*.config*). We then tested mantis-ml's performance with each of the standard classifiers when we used different configurations of features: all features that survived after the feature

Table 2. Query Terms per Disease Category Provided in the Configuration File for Mantis-ml (*config.yaml*)

	Disease or Phenotype terms	Additional associated terms	Diseases or Phenotypes to exclude
Chronic kidney disease (CKD)	renal, kidney, nephro, glomerul, distal tubule	-	adrenal
Epilepsy	epilep, seizure	brain, nerve, nervous, neuronal, cerebellum, cerebral, hippocampus, hypothalamus	-
Amyotrophic lateral sclerosis (ALS)	amyotrophic lateral sclerosis, degeneration of the lateral corticospinal tracts, dysfunction of lateral corticospinal tracts, atrophy of the spinal cord, progressive distal muscular atrophy, spinal muscular atrophy, first dorsal interossei muscle atrophy, cervical spinal cord atrophy, corticospinal tract atrophy, corticospinal tract hypoplasia, atrophy/degeneration involving the spinal cord	brain, muscle - skeletal, nerve, nervous, neuronal, spine, spinal, cerebellum, cerebral, hippocampus, hypothalamus, muscular dystrophy, muscular fitness, muscle function, muscle, neuromuscular	heart muscle, heart_muscle, cardiac_muscle, smooth_muscle, cardiac muscle, smooth muscle, striated_muscle, striated muscle

All query terms are case insensitive and follow regular rules of wild card pattern matching. Cells with a dash should be left empty in the config.yaml file.

pre-processing step or only Boruta-confirmed features (Figure S18). Training and prediction were performed with a set of 15 random balanced datasets from the CKD disease example. Mantis-ml performed slightly but non-significantly better when using the entire feature set (average AUC, 0.817 versus 0.815; two sample t test, $p = 0.515$). However, since Boruta does not need to be run by default as part of the mantis-ml workflow, the default configuration retains the entire processed feature space and allows the user to further explore by explicitly specifying “boruta” as the feature selection algorithm in the “supervised_filters” field in mantis_ml/conf/.*config*. We also tested the performance of the “stacking” classifier when fed with only the base classifier predictions versus by employing the original feature space on top of the extracted base classifier predictions. The stacking classifier’s performance was considerably better when retaining the original feature space at the second layer of the ensemble training and prediction, and this is the default configuration used by mantis-ml.

Enrichment of Top Mantis-ml Predictions among Different Types of Qualifying Variants

We performed a stepwise hypergeometric test to assess the enrichment of high mantis-ml predictions (top 5% per disease) among different types of qualifying variants from the collapsing analyses (focusing on the collapsing analyses gene subsets with p value <0.05).

We observed that the enrichment of high mantis-ml predictions among top-ranked putative loss-of-function (pLoF)-associated genes is always statistically significantly different both from a shuffled (randomized pLoF-associated gene list) enrichment signal (Mann-Whitney U test p value $= 2.91 \times 10^{-300}$, 5.53×10^{-147}, and 4.17×10^{-194} for CKD, epilepsy, and ALS, respectively) and the enrichment signal of genes associated with synonymous variants (Mann-Whitney U test p value $= 1.34 \times 10^{-125}$, 1.60×10^{-33}, and 2.36×10^{-84} for CKD, epilepsy, and ALS, respectively). For the enrichment analysis of synonymous variants, we have considered “Dom_coding” (dominant coding) as the comparator class in ALS because of the lack of a real synonymous-based collapsing-analysis gene list in the published analysis.

We sought to explore how each of the seven classifiers performed when overlapped with top-ranked whole-exome sequencing (WES)-based gene lists. We observed that with regard to AUC, the best-performing classifiers per disease category in mantis-ml also ranked among the top three classifiers in terms of area-under-curve ratios and/or total pLoF area (Figures S10–S12). Other classifiers also performed comparably or slightly better, which is in concordance with the similar AUC performance achieved from differing classifiers retrieved from the original mantis-ml training, again reinforcing the consistency and robustness of the framework irrespective of chosen classifier.

Visualization of Cross-Validated Mantis-ml Predictions and Downstream Analysis

After the cross-validation of mantis-ml predictions with rare-variant collapsing-analysis studies, we apply dimensionality reduction on the original feature space to highlight the novel predicted genes-of-highest interest in the entire exome space. PCA performed for each of the three disease examples used in this study achieves a slight segregation of positive and unlabeled genes; the consensus novel and known gene predictions tend to differentiate the most from the rest of genes (Figure S14). However, PCA fails to capture a high ratio of the total variance to be explained by its first two or three components (the variance explained by the first three components in each disease case is on average about 21%). Because PCA is representing the original features as linear combinations of the projected principal components, its inability to identify patterns of high variability in the entire gene set implies that the associations between the various collections of features driving gene predisposition to disease are probably non-linear.

Thus, we then apply two popular dimensionality-reduction techniques that can identify non-linear patterns in the original high-dimensional feature space for each disease example: t-SNE and UMAP. We observe that both methods map most of the consensus novel genes-of-highest-interest in the neighborhood of distinct clusters of known genes (Figure S15). Both techniques capture patterns in more localized regions of genes, although UMAP might also retain elements from the global structure more efficiently than t-SNE. By contrasting the two projections, we can identify clusters of genes that are more likely to be close (i.e., similar) to each other in absolute terms of distance (similarity). Finally, the mantis-ml tool provides interactive visualizations of all three projections (PCA, t-SNE, and UMAP) for further inspection of gene clusters and offers all extracted two-dimensional

representations of the original data space that allow for further extraction of clusters of genes (e.g., through the use of HDBSCAN for further downstream analysis).

Benchmarking of Gene-Prioritization Tools

Benchmarking of gene-prioritization tools is usually impeded by the high variability of input data, target-prediction goals, and output results provided by each tool. For this test, we selected the current state-of-the-art tools that allow for as much direct comparison against mantis-ml as possible.

Phenolyzer runs similarly to mantis-ml, in that the user only needs to provide disease-associated terms in free text. We ran Phenolyzer with the following terms by disease:

- CKD- "chronic kidney disease, nephropathy, glomerulopathy, kidney, renal" (in accordance with the terms employed by mantis-ml- "renal, kidney, nephro, glomerul, distal tubule")
- Epilepsy- epilepsy, seizure (in accordance with the terms employed by mantis-ml- "epilep, seizure")
- ALS: "amyotrophic lateral sclerosis, degeneration of the lateral corticospinal tracts, dysfunction of lateral corticospinal tracts, atrophy of the spinal cord, progressive distal muscular atrophy, spinal muscular atrophy, first dorsal interossei muscle atrophy, cervical spinal cord atrophy, corticospinal tract atrophy, corticospinal tract hypoplasia, atrophy/degeneration involving the spinal cord" (exactly the same as with mantis-ml).

For all Phenolyzer runs, we selected the "disease only" option in the "phenotype interpret" parameter. Selecting the "phenotype interpretation" option (i.e., looking for both diseases and phenotypes) for this parameter did not give any enrichment against collapsing analysis predictions (data not shown). On the other hand, we trained mantis-ml by selecting any phenotype or disease term associated with the provided string input (i.e., not restricting it exclusively to disease-associated terms), yet it managed to capture probable pathogenic gene signals substantially better than Phenolyzer. All other parameters for Phenolyzer were used with their default value in the webserver application:

- "Gene selection/Region selection/Weight Adjust/Word Cloud"- no
- "Addon Seed Gene"- "DisGenet Disease Gene Mapping," "Genetic Association Database"
- "Addon Gene Relations"- nothing selected
- "Addon Gene Scores": "Gene Haploinsufficiency Score," "Gene Intolerance Score"

When we tested Phenolyzer's predictions against the collapsing analyses, we ensured that we were testing only for genes that are represented both in Phenolyzer's output and in the collapsing results to avoid any unfair conclusions in the results.

With regard to ToppGene, for benchmarking on ALS we provided the same set of seed genes as with mantis-ml (77 genes) and provided the rest of the exome as the test set. Attempting to run ToppGene for CKD and epilepsy by providing the respective mantis-ml-generated sets of seed genes (587 and 864 genes, respectively) and providing the rest of the exome as the test set was not possible because the webserver was crashing while trying to read and pre-process the original training and test-set input. We thus employed as our test set a smaller set of genes that was amenable to processing by the ToppGene webserver. In order to compensate for the inability of ToppGene to look into the entire exome, we provided the top 7,000 genes predicted by the collapsing analyses on CKD and epilepsy as our test set, thus informing it with approximately the top one-thirdof the exome, which is more likely to be associated with the respective disease.

The training and test sets employed by ToppGene for each disease were also used for ToppNet. In addition to that, we selected the default graph-prioritization parameters for each disease example:

- Prioritization method: k-step Markov
- Step size: 6

All data and scripts used for benchmarking are available at the GitHub repository under "mantis-ml-release/misc/overlap-collapsing-analyses" and are separated into folders for each benchmarked tool.

Concordance of Classifier Predictions for the Generic Mantis-ml Score

The generic mantis-ml score (GMS) was trained on the basis of all OMIM disease-associated genes via six different classifiers: random forest, extra trees, gradient boosting, XGBoost, SVC, and DNN. Here, we have excluded the stacking classifier because it has a much longer training time than the other classifiers and, as a result, has a slightly lower AUC performance. Results retrieved by all classifiers were highly concordant. Specifically, of the 4,041 known genes, around 1,300 (32.2%) were consistently identified by all classifiers (with probability >0.5). Another 380 known genes (9.4%) were further identified by at least five of the six classifiers. With regard to novel disease-associated genes, again the largest group of predicted genes (n = 600) was predicted by all classifiers, and ~320 novels genes were predicted by at least five classifiers. In both cases, DNN had the highest number of known and novel predictions identified solely by a single classifier (280 and 680 predicted genes, respectively).

Mantis-ml Package Structure

We built our mantis-ml framework by using Python on top of the *sckit-learn* and *keras* libraries. We have also employed the "Boruta" R package for feature selection based on the Boruta algorithm. The main components of mantis-ml are the "pre_processing," "unsupervised_learn," "supervised_learn," "post_processing," and "validation" modules. The "pre_processing" module implements the functionality for compilation of the input feature table, which contains three classes of features: generic features (tissue and/or disease-agnostic), features filtered by tissue and disease-specific features. Compilation of tissue and/or disease-specific features is performed with a curated dictionary of relevant query terms. After data compilation, the "pre_processing" module implements the rest of its main functionality around feature pre-processing, exploratory data analysis, and visualization of features distribution. The "unsupervised_learn" module performs dimensionality reduction on the processed feature set for visualization purposes and extraction of two-dimensional representations of the data for downstream analysis, such as clustering and pathway-enrichment analysis. Processed feature tables are then passed on to the "supervised_learn" module for feature selection with Boruta and the stochastic positive-unlabeled learning task, which is the core

of the mantis-ml workflow. Prediction probabilities extracted from this step are fed to the "post_processing" module for aggregation of results and optional overlap with third-party studies (e.g., rare-variant cohort studies or any independently generated ranked gene list) through use of the "validation" module.

External Libraries Used for Implementations of Supervised-Learning Models

For random forest, extra trees, gradient boosting, and SVC, we used their implementations from scikit-learn (v0.20.3), whereas for DNNs we used Keras (v2.2.4) with Tensorflow (v1.10.0) in the backend. XGBoost's implementation was provided by the xgboost Python package (v0.80).

Computational Requirements and Time Complexity

All three benchmarked disease examples have been run on a simple linux utility for resource management (SLURM) cluster with 10 CPU cores (Intel(R) Xeon(R) CPU E5-2683 v. 4 at 2.10 GHz) for ten stochastic iterations. The total time required for each study was inversely proportional to the respective number of seed genes because this directly influences the total number of random balanced datasets that need to be trained with k-fold cross-validation. Specifically, total execution time across all classifiers was 1 h 55 min, 2 h 33 min, and 11 h 23 min for epilepsy, CKD, and ALS, respectively (for 864, 587, and 77 seed genes, respectively).

Results

Mantis-ml Overview

We developed mantis-ml as an automated machine-learning (AutoML) framework to enable learning from an arbitrary set of gene-associated features (Figure S1). We collated data from a diverse set of gene-annotation sources (Figure 1A and Table S1) classified into three categories: generic resources (disease and/or tissue agnostic), resources filtered by tissue, and finally, disease-specific features. Given a set of user-specified query terms relating to a tissue and/or disease of interest, the data compilation and cleaning is performed automatically (see Methods). Currently, over 1,200 gene-annotation features are integrated in our framework. Additionally, mantis-ml automatically generates a rich set of visualizations for exploratory analysis on the original feature space (see Methods).

Stochastic Semi-supervised Learning for Gene Prioritization

Positively labeled genes for the gene prioritization are retrieved by mantis-ml from the Human Phenotype Ontology[5] (HPO) on the basis of user-provided inclusion and exclusion query terms that are relevant to a particular disease (see Methods). The HPO component then identifies seed genes on the basis of the documentation of gene-disease association in OMIM and additional clinical terminology curated by clinicians participating in regular workshops hosted by the HPO team. An important peculiarity for this problem derives from the fact that in most diseases, the overall set of known disease-associated genes (positive labels) is typically in the range of approximately tens to hundreds of genes. This makes the entire protein-coding gene set (n = 18,626) highly imbalanced in terms of the overall population of positive and unlabeled data points. Additionally, the entire gene space is finite and practically already known, so we are not bound by the usual machine-learning requirement to train a model that can generalize well on unseen data. Our end goal is to rank the entire gene set with respect to a diverse pool of disease groups and ensure that our predictions are not biased by a single training dataset of a subset of genes assumed to be representative of the global distribution of all genes. As a result, and in combination with the lack of a well-defined negative set, training a sufficiently generalizable model to then make predictions on the basis of a test set would not be ideal in this context.

To address this issue, we constructed a gene-prioritization framework that is based on a positive-unlabeled approach: a stochastic semi-supervised learning technique (L iterations) across multiple random balanced datasets from the entire gene set (Figures 1B and S1B) with iterative predictions on out-of-bag data. In each stochastic iteration, we create a random partitioning of the unlabeled gene space to form M balanced datasets, with a positive-to-unlabeled points ratio equal to 1:1.5. Each balanced dataset contains a random X percentage sample of the positive (seed) genes (default $X = 80\%$) to reduce bias induced by use of the entire positive gene pool in each training task. The unlabeled data are treated as negative because training on positive and unlabeled data in general gives scores proportional to the ones retrieved by training on positive and negative data.[18] We then perform a stratified k-fold split on each balanced dataset (default $k = 10$) and train with a standard classifier for each possible combination of $k - 1$ folds (training set) followed by prediction each time on the out-of-bag k^{th} fold (test set). This process is performed k times over each balanced dataset, and upon each training cycle, prediction probabilities are retrieved only for the genes belonging to the respective test set (out-of-bag k^{th} fold). We tested seven different classifiers to be used during positive-unlabeled learning for each balanced dataset (see Methods). All classifiers performed comparably (average AUC, 0.831–0.850), implying that mantis-ml is not sensitive to the underlying machine-learning method but is rather enabled by the informativeness of the integrated data itself.

This process creates, from the entire gene space, multiple smaller gene poolsthat have comparable numbers of known and unlabeled genes and allow the classifier to capture strong patterns among the known genes to then rank all genes (both known and novel) with respect to a disease profile. Notably, this process can also identify mislabeled known genes and automatically readjust their rank given a sufficiently curated starting set of known disease-associated genes. The entire procedure is repeated for L iterations, each one leading to a random set of balanced sets to allow inclusion of each gene in out-of-bag sets multiple times and subsequently lead to less biased and more robust

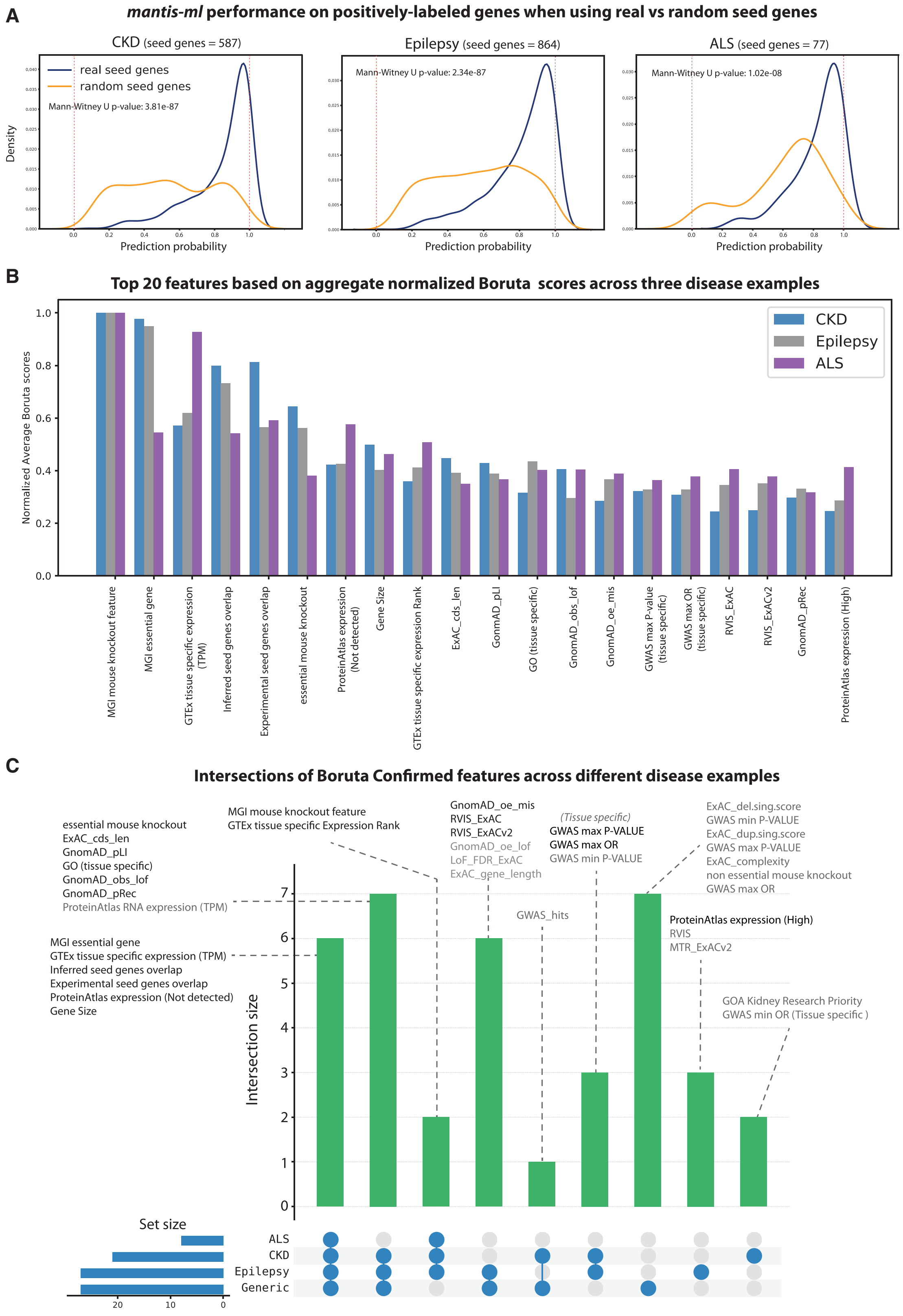

Figure 3. Mantis-ml Performance Sensitivity on Seed Genes and Consensus of Top Feature Contributors across Different Disease Examples as Determined with the Boruta Algorithm

(A) Prediction probability distributions from positively labeled (seed) genes across the three disease examples when selected from HPO versus randomly assigned. Random seed genes are predicted with an almost uniform probability distribution, whereas real seed genes successfully get ranked to the top of the spectrum (probability values close to 1).

(legend continued on next page)

results. mantis-ml does not define a static underlying model but prioritizes all genes on the basis of the probability prediction they have achieved over multiple iterations that have grouped the genes into random balanced groups. Eventually, we aggregate the prediction probabilities assigned to each gene member from out-of-bag sets (either positive or unlabeled) across all $L \times k \times M$ iterations. For each gene, this forms a probability distribution regarding the association of a gene with the disease under examination. The final gene predictions are ranked on the basis of the mean of their probability distributions (results equivalent with median, Pearson's $r > 0.9996$, $p < 2.2 \times 10^{-308}$).

Application on Three Disease Examples: ALS, CKD, and Epilepsy

We applied mantis-ml on three complex diseases: ALS, CKD and epilepsy (genetic generalized epilepsy). We selected these because studies involving hypothesis-free exome-wide association statistics have been previously published for these disease examples. The positively labeled gene set for each disease was selected on the basis of a user-defined curated dictionary of inclusion and exclusion terms used for automated querying and extraction from HPO (Table 2). Tissue-specific and disease-relevant features were automatically extracted on the basis of the same query terms applied to the mantis-ml integrated knowledgebase (see Methods). The total number of known (seed) positively labeled genes found for each disease on the basis of HPO was 587, 864, and 77 for CKD, epilepsy, and ALS, respectively.

We first ran a benchmarking test on ten random balanced datasets from the CKD example and obtained an average best AUC performance of 0.85 (Figure 2; see Methods); scores were comparable across all classifiers. We then applied all seven classifiers on each disease example Each yielded comparable performance (see Methods): average AUC scores were 0.846, 0.821, and 0.814 for CKD, epilepsy, and ALS, respectively. Specifically, XGBoost and random forest had the best performance in CKD (average AUC: 0.846); this was followed by gradient boosting and extra trees (average AUC: 0.843 and 0.839, respectively), and some of the most well-established CKD genes (*PKD1*, *PKD2*, *COL4A1*, *COL4A3*, *COL4A4,* and *COL4A5*) ranked in the top 0.2%–0.7% of all genes (Figure S6). An aggressive prediction-probability threshold of 0.5 was used for classifying genes as either predicted known or novel and assessing the concordance of results across classifiers. We observe high concordance between the predictions from all classifiers with regard to known and novel disease-associated genes (see Supplemental Methods). Having no further knowledge to validate mantis-ml predictions at this stage, we choose to consider the gene rankings of the classifier with the highest average and individual AUC scores as the default mantis-ml prioritization scheme for the respective disease.

We then sought to examine the importance of the seed-gene set for the prediction performance of mantis-ml. We thus tested the prediction probabilities across all seed (positively labeled) genes in the three disease examples (CKD, epilepsy, and ALS) when the seed-gene set is selected either randomly or on the basis of the HPO annotation (Figure 3A). The size of seed-gene sets varies in these disease examples (CKD: 587, epilepsy: 864, and ALS: 77), allowing exploration of the importance of seed-gene lists of varying length. We observed that in all three diseases, the prediction probabilities for seed genes when a real seed-gene set was used are skewed toward a probability of 1.0, whereas the respective distribution acquired when random seed genes of the same length were used is almost uniformly distributed across the entire probability spectrum (0.0–1.0). In all cases, the mantis-ml probability-score distributions obtained when real seed genes were used were significantly different from those obtained with random seed genes of matched gene-set size (Mann-Whitney U test p value $= 3.81 \times 10^{-87}$, 2.34×10^{-87}, and 1.02×10^{-08} for CKD, epilepsy, and ALS, respectively).

Mouse Model Phenotypes, Tissue Expression, Protein-Protein Interactions, and Intolerance Metrics Are Recurrently among the Top Features

Although mantis-ml focuses on identifying features highly predictive of known seed genes, we expect that some features are generally strong predictors of disease-associated genes. In total, mantis-ml integrates more than 1,200 features (Figure 1) that are automatically subset according to the disease under study. We sought to explore the contribution of each of the features during learning across all three examined disease examples. We adopted the Boruta[21] algorithm based on a random forest classifier across 100 random balanced gene subsets with 10-fold cross-validation (see Methods). The Boruta algorithm provides an unbiased assessment of feature contribution because it constructs artifactual features (shadow features) from random permutations of each of the actual features of a dataset and then iteratively confirms or rejects the original features on the basis of their Z score distances from the importance levels achieved by the random (shadow) features. We ran Boruta for CKD, epilepsy, and ALS and extracted the consensus profile of feature importance in each disease case across ten stochastic iterations (Figures S9A–S9C; see Methods). We then normalized the Z scores among the three disease cases (min-max normalization)

(B) Top feature contributors per disease example are based on the sum of normalized average Z scores returned by Boruta. Features are ranked merely on the basis of their Z scores and are considered confirmed features according to this rank but without reference to whether they reach significance level.
(C) Intersection of confirmed Boruta features across the three disease examples (CKD, epilepsy, and ALS) and the generic disease classifier. Features in black font correspond to the top 20 features as determined by the aggregate normalized Boruta scores.

to compare the relative importance of each feature in the different diseases and provide a disease-agnostic consensus of the feature-importance profile (Figures 3B and 3C).

The consensus of feature selection across CKD, epilepsy, and ALS reveals mouse-model phenotypes and tissue-specific expression (in kidney, brain, and either brain or skeletal muscle, respectively, on the basis of ProteinAtlas and GTEx) as consistently highly important contributors. Specifically, the "MGI mouse knockout feature" is the top contributor in all three cases. This feature captures human genes with mouse orthologs that (according to Mouse Genome Informatics [MGI]) are associated with a "high-level mammalian phenotype" relevant to the disease under study (see Methods). Moreover, human orthologs of mouse genes that have been found to be essential for basic developmental functions and/or survival in mice (MGI essential gene) are the next top contributors in CKD and epilepsy and are among the top contributors for ALS. Tissue-specific expression ["GTEx tissue specific expression (TPM)", "ProteinAtlas expression (not detected)," and "GTEx tissue specific expression rank"] follow in the order of consistent feature importance and makes a particularly high contribution to ALS compared to the other two disease cases. An interesting outcome of the Boruta algorithm is the emergence of protein-protein interactions-related features' ("inferred seed genes overlap" and "experimental seed genes overlap") being ranked in the top five recurrently important features. These represent bespoke constructed features to capture the ratio of known (seed) genes interacting directly with the index gene on the basis of either an "experimental" or "inferred" prediction from the "InWeb_IM" resource[8] (see Supplemental Methods). Finally, tissue-specific gene ontology (GO) terms, intolerance scores based on ExAC and GnomAD ("GnomAD_pLI", "GnomAD_obs_lof," "GnomAD_oe_mis," "GnomAD_pRec," and "RVIS"), tissue-specific GWAS metrics ("GWAS max p value" and "GWAS max OR"), and gene size complete the picture of the most contributing features for classification of disease-associated genes.

Application of Mantis-ml Predictions to Support Triaging of Exome-wide Cohort Association Studies

The rapid development of next-generation sequencing (NGS) technologies in recent years has led to the ubiquitous application of large-scale genomic studies by large genomic and/or healthcare institutions for research and diagnostic purposes. Of special interest are large association studies that assess the enrichment of rare predicted deleterious variants in a collection of disease-ascertained cases in comparison to an available control population. Depending on the contribution of individual genes to disease risk, these studies can provide experiment-wide significant results,[15,22] but more often they yield many highly ranked genes of interest that do not exceed the multiplicity-adjusted genome-wide statistical-significance threshold. Thus, biologically relevant genes are expected to be residing among stochastic signals representing the natural tail of the null distribution. Teasing apart the biological signals from stochastic signals among the top ranks is a key challenge for most high-throughput genomics screens. Often downstream bioinformatic analysis involves laborious post-hoc review through existing literature, and this process can be biased by differing decisions influenced by an individual researcher's prior experience or familiarity. Thus, the adoption of different resources by different researchers will ultimately result in triaging different genes.

Mantis-ml eliminates subjectivity and post-hoc design from gene prioritization by using a standardized set of community knowledge and collectively assessing all interactions (both linear and non-linear) between multiple features. To demonstrate the utility of leveraging the power of mantis-ml predictions to support triaging candidate genes found among results from WES-based association studies, we selected three disease studies whereby genes have been previously ranked on the basis of the significant case-enrichment of various types of qualifying variants, e.g., pLoF, missense, synonymous, etc. For CKD, we are cross-referencing a study examining the preponderance of rare pLoF and other types of rare variants across a population of 3,150 affected individuals and 9,563 controls.[14] Another study of 640 individuals with familial genetic generalized epilepsy and 3,877 controls has been employed for further triaging on the basis of the mantis-ml predictions for the epilepsy disease case.[15] Finally, for ALS, we are using the results from a study looking into the collapsing analysis results of nearly 3,000 individuals with ALS versus 6,405 controls.[13]

In each example, we ask the question of whether the lowest p values from the exome-wide association statistics (highest ranked from cohort studies) are significantly enriched for genes that achieved among the highest mantis-ml predictions for that corresponding disease. To this end, we apply a hypergeometric enrichment test asking whether the top 5% of mantis-ml predictions in each disease example are preferentially overlapping with the top signals (genes with $p < 0.05$) from the cohort-level association studies. To strengthen our experimental design with negative control components, we assess this enrichment across several differing classes of qualifying variants, including synonymous and permutation-based gene-ranks, which should represent the null, to contrast the more biologically interesting models, such as the pLoF and ultra-rare deleterious missense collapsing analyses.

We observed a strong enrichment in all three disease examples, and mantis-ml predictions overlapped significantly with the exome study ranking for pLoF variants (Figure 4A; see Methods). Notably, the significance of enrichment for pLoF signal within mantis-ml predictions is higher than for signals formed on the basis of other types of qualifying variant classes (synonymous, common, missense, and shuffled/random permutation; pLoF p value = 0.0004, 0.0005, and 0.009 for CKD, epilepsy, and ALS, respectively, on the basis of the hypergeometric

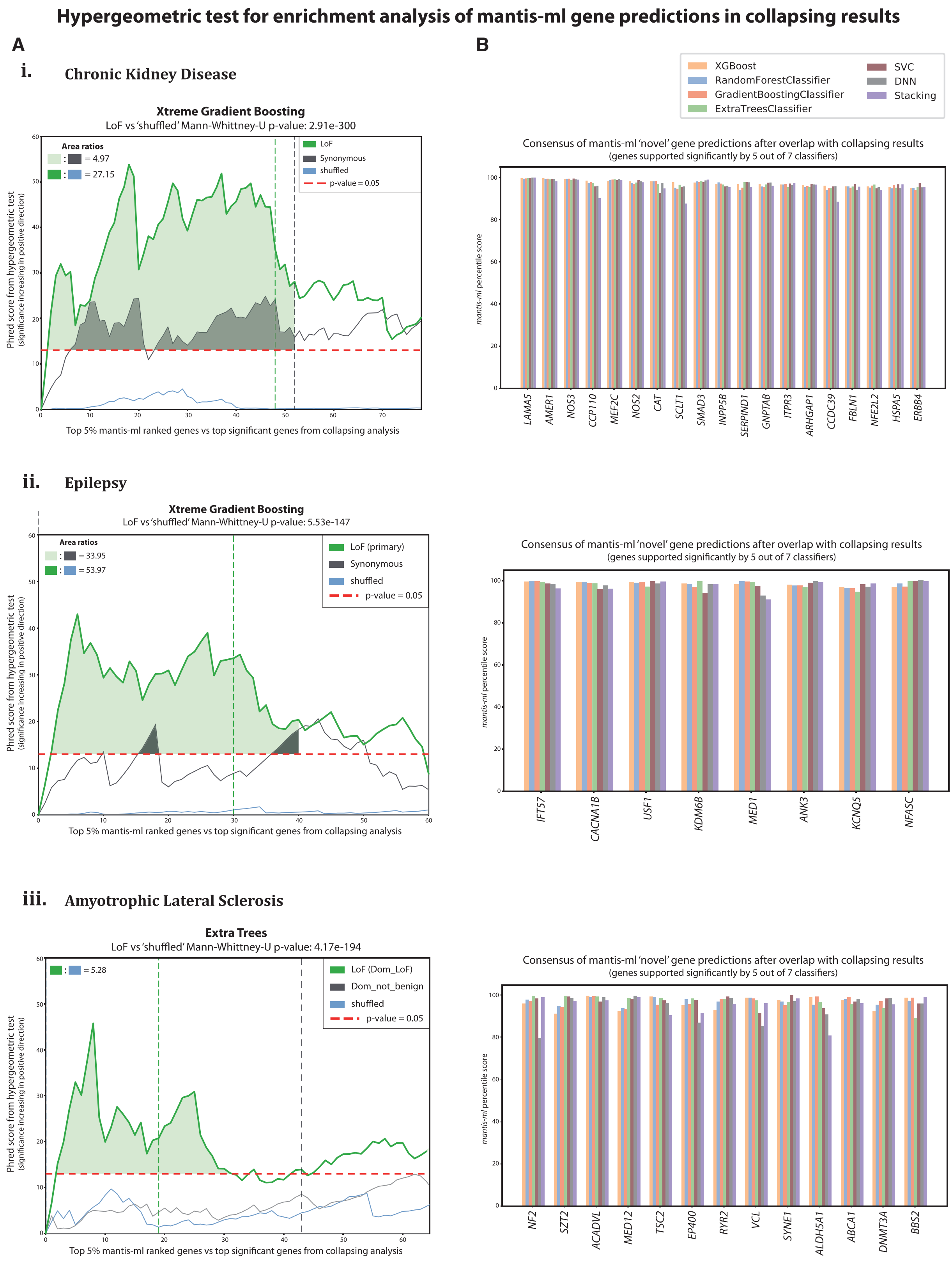

Figure 4. Cross-Validation of Mantis-ml Predictions (Wehn Both Known and Novel Genes Are Considered) with Cohort-Level Rare-Variant Association Studies

(A) Hypergeometric test enrichment of disease-specific mantis-ml predictions with collapsing-analysis results from CKD (1), epilepsy (2), and ALS (3) cohorts. The horizontal dashed red line corresponds to the significance threshold of p = 0.05 for the hypergeometric tests. The places where the plot(s) go above this line highlight significant enrichment of mantis-ml-top-gene predictions among the population-genomic collapsing analyses. The vertical dashed lines, colored on the basis of the different classes of qualifying variants, indicate

(legend continued on next page)

test at the step where collapsing analysis reaches a p value of 0.05). Additionally, the enrichment of high mantis-ml predictions among top-ranked pLoF-associated genes is always statistically significantly different both from a shuffled enrichment signal (Mann-Whitney U test p value = 2.91×10^{-300}, 5.53×10^{-147}, and 4.17×10^{-194} for CKD, epilepsy, and ALS, respectively) and from the enrichment signal of genes associated with synonymous variants (Mann-Whitney U test p value = 1.34×10^{-125}, 1.60×10^{-33}, and 2.36×10^{-84} for CKD, epilepsy, and ALS, respectively). This evidently suggests the biological relevance of mantis-ml predictions with pathogenicity likelihood.

We further quantified the enrichment signal by calculating the ratios of areas under the curve between the pLoF and synonymous collapsing-analysis signals for CKD and epilepsy; for ALS, we used the total areas covered by the pLoF enrichment signal for ALS (because of the lack of a synonymous-associated signal in the respective published study; Figure 4A). The synonymous enrichment signal serves as a negative control (technical baseline) because we expect genes prioritized during collapsing analysis based on synonymous variants not to be associated with pathogenicity, in general.

We also performed the hypergeometric test to assess the enrichment of mantis-ml-predicted "known" or "novel" genes, separately, against the results of the collapsing analysis. We observe that mantis-ml predictions for "known" genes are significantly enriched for the top results of the collapsing analysis for all three disease examples (Figure S22A), indicating the biological relevance of the HPO-extracted seed genes (average p values in the significantly enriched region for ALS, 0.0085; CKD, 0.0034; epilepsy, 0.019). Similarly, mantis-ml predictions of "novel" genes are also significantly enriched for the top collapsing-analysis hits (Figure S22B) consistently across all three disease examples, proving that mantis-ml captures novel signals that have direct genetic evidence and, thus, are more likely to be biologically relevant for the disease of interest (average p values in the significantly enriched region for ALS, 0.013; CKD, 0.044 ; epilepsy, 0.031).

By applying the hypergeometric test for mantis-ml prediction enrichment in published case-control association studies, we can eventually extract a consensus list of predicted novel genes of highest interest that satisfy both the hypergeometric test ($p < 0.05$) and the collapsing-analysis statistical significance threshold ($p < 0.05$). Through the application of mantis-ml predictions onto published collapsing-analysis results, we are able to highlight 19 (CKD), eight (epilepsy), and 13 (ALS) novel (unlabeled) genes of highest interest. To validate this approach, we also assessed the results retrieved with regard to known (seed) genes for each disease. For example, in CKD we observe that some of the most well-established CKD-associated genes (*PKD1*, *PKD2*, *COL4A1*, *COL4A3*, *COL4A4*, and *COL4A5*) rank in the top nine genes among the 17 known CKD genes that achieved both a collapsing analysis $p < 0.05$ and hypergeometric test $p < 0.05$ (Figure S13).

Downstream Review of Cross-Validated Mantis-ml and Collapsing-Analysis Predictions

The unlabeled genes of highest interest (novel mantis-ml predictions) represent a collection of genes that were not among the HPO-derived set of seed genes in the initial process of mantis-ml. We looked in the literature for references for the top-suggested novel genes per disease by mantis-ml and found supporting evidence for several genes. For instance, it has been reported in the last two years that LAMA5 variants are co-inherited with COL4A5 variants in familial hematuria,[23] and such co-inheritance might affect pediatric nephrotic syndrome.[24,25] Moreover, *NOS3* and *NOS2*—although not associated with CKD in OMIM—have been implicated in CKD in multiple studies,[26–29] whereas *MEF2C*, a gene typically associated with neurodevelopmental disorders, has also been associated with estimated glomerular filtration rate (eGFR) or proteinuria.[30] *SCLT1* deficiency has been linked with cystic kidney disease,[31] and *SMAD* genes (including SMAD3) have been reported to affect CKD progression when they are dysregulated.[32] *INPP5B* impairment has been associated with severe renal phenotypes, such as proximal-tubule endocytosis,[33] and targeting *NFE2L2* (NRF2) has been tested as a method of preventing kidney disease progression.[34]

With regard to the top novel epilepsy-associated predictions, *CACNA1B* is associated with the voltage-gated calcium channel that has been only recently implicated in epileptic phenotypes.[35–37] *USF1* deficiency in mice (in combination with *USF2* knockout) has been shown to cause epileptic seizures,[38] suggesting the important role this gene plays in normal brain function. Furthermore, *KDM6B* is associated with neuronal survival,[39] and when haploinsufficient, it has been reported to cause severe seizures.[40] *ANK3* has also been reported to be involved in epilepsy.[41,42] Loss-of-function (LoF) and gain-of-function variants in *KCNQ5* have been shown to cause epileptic encephalopathy.[43]

As for the ALS consensus novel predictions, missense variants in *SYNE1* have been reported to be associated with a multisystemic neurological-phenotypic spectrum

the last index of top-ranked genes from the collapsing analyses achieving a p value <0.05. The highlighted areas (light green and gray) represent the magnitude of enrichment signal for LoF and synonymous variants identified both from the collapsing analyses ($p < 0.05$) and the hypergeometric enrichment test against mantis-ml predictions ($p < 0.05$).
(B) Consensus of genes of highest interest (novel) that satisfy the significance-threshold criteria in both the collapsing-analysis and the hypergeometric results and that are supported by five out of seven classifiers used by mantis-ml in the CKD (1), epilepsy (2), and ALS (3) disease examples.

that includes ALS (see Web Resources).[44,45] *ALDH5A1* is significantly downregulated in the spinal cord of an ALS murine model,[46] whereas *ABCA1* is among the altered genes in the frontal cortex of ALS samples.[47] Finally, motor neurons in human ALS show significant abnormalities in *DNMT3A*, which is also overexpressed in synapses of mice with motor-neuron degeneration.[48]

We also report sets of novel genes that have not been previously associated with the respective disease. Specifically, we have predicted the following sets of most likely novel disease-associated genes: *AMER1*, *CCP110*, *CAT*, *SERPIND1*, *GNPTAB*, *ITPR3*, *ARHGAP1*, *CCDC39*, *FBLN1*, *HSPA5*, and *ERBB4* for CKD; *IFT57*, *MED1*, and *NFASC* for epilepsy; and *NF2*, *SZT2*, *ACADVL*, *MED12*, *TSC2*, *EP400*, *RYR2*, *VCL*, and *BBS2* for ALS.

Finally, we employ three-dimensionality reduction methods (PCA, t-SNE, and UMAP) and provide static and interactive visualizations of the predicted known and novel genes over the transformed two-dimensional spaces retrieved with each technique for visual exploration and downstream analysis (see Methods).

Benchmarking against Other Gene-Prioritization Methods

We then wanted to assess how mantis-ml predictions compare with previously published methods. Mantis-ml performs an exome-wide prioritization of genes without explicitly requiring a user-defined test set. Thus, we sought to employ methods that provide gene rankings across the whole exome or at least a large proportion of it so that they can be directly compared with mantis-ml. Eventually, we selected three methods for this benchmarking test: Phenolyzer,[49] which is the current state-of-the-art method, and another two popular tools, ToppGene[50] and ToppNet.[51] With regards to selecting a ground truth set for our benchmarking, we wanted to use a completely independent dataset so that none of the benchmarked tools, including mantis-ml, had features informed by our truth set, thus inducing a circular feedback loop of prediction and, consequently, biasing the results. We therefore employ the results from rare-variant genetic association studies (collapsing analyses[12]) to assess the enrichment of highly ranked genes from each tool against the top ranked genes from each cohort study, which are highly enriched for loss-of-function variants in cases compared to controls.

Earlier, we demonstrated that mantis-ml's predictions are highly enriched for the top predictions from collapsing analyses across three diseases for which data are publicly available (CKD, ALS, and epilepsy). We thus wanted to explore the degree of enrichment for the predictions from each external benchmarked tool when trained for the same diseases and phenotypes. We trained each tool by providing either the same disease-relevant terms or the same training set of genes as in mantis-ml, where applicable (see Methods). We ran the enrichment test against all gene predictions from mantis-ml and Phenolyzer because they provide prioritization scores for both "known" and "novel" genes (Figure 5A). ToppGene and ToppNet do not include prioritization results for the provided "known" (seed) genes; thus we also ran enrichment tests against only the novel "gene" predictions across all benchmarked tools (Figure 5B). We observe that mantis-ml enrichment among predictions from genetic association studies is substantially higher than enrichment for all other methods across all three disease examples (Figure 5). Phenolyzer achieves a significant enrichment for CKD and epilepsy when both known and novel genes are considered but does not reach the significance threshold for predictions of novel genes. ToppGene's novel predictions are relatively enriched in ALS, and ToppNet's novel predictions are borderline enriched in CKD and ALS. However, in all cases, enrichment derived from each of these tools is considerably lower than that derived from mantis-ml, and each of these methods overlaps with a much smaller set of genes that are significantly enriched in the respective collapsing analysis. Thus, mantis-ml provides the best-in-class prioritization for data extracted from whole-exome -sequencing studies and potentially from other high-throughput genomic studies as well (e.g., whole-genome sequencing and CRISPR screens).

Generic Mantis-ml Score for Gene Disease Likelihood

One of the key requirements for the above three disease-specific applications of mantis-ml is a sufficient collection of known OMIM disease-associated genes based on HPO term linkage. A rich collection of genes will not always be available. Therefore, we wanted to further explore the generation of generic mantis-ml predictions. To achieve this, we trained mantis-ml by using all current OMIM disease-associated genes (4,041 in total; see Methods) as seed genes to create a GMS that can be used as a general estimate of gene-disease likelihood. Although it does not take full advantage of tissue- and disease-specific features, the GMS could be an opportunity to prioritize genes among disorders about which we currently have insufficient knowledge about disease-associated genes.

We calculated the OMIM-based GMS by using six different classifiers (see Methods). Gradient boosting was the top-performing classifier (average AUC = 0.84), followed by random forest, XGBoost, and extra trees with comparable AUC scores (Figure S16). Similar to the disease-specific cases, in the generic disease mantis-ml results, we observe a high concordance between the predictions from all classifiers with regard to known disease-associated genes among the out-of-bag test sets (see Methods). We provide the average probability scores returned by gradient boosting as the default ranking for GMS along with the respective percentile score for each gene (Table S5).

Furthermore, we re-ran the Boruta algorithm for a single ($L = 1$) stochastic iteration to identify the most important features that drive gene classification on the basis of the entire OMIM disease annotation and compared it against the respective features (where applicable) extracted from

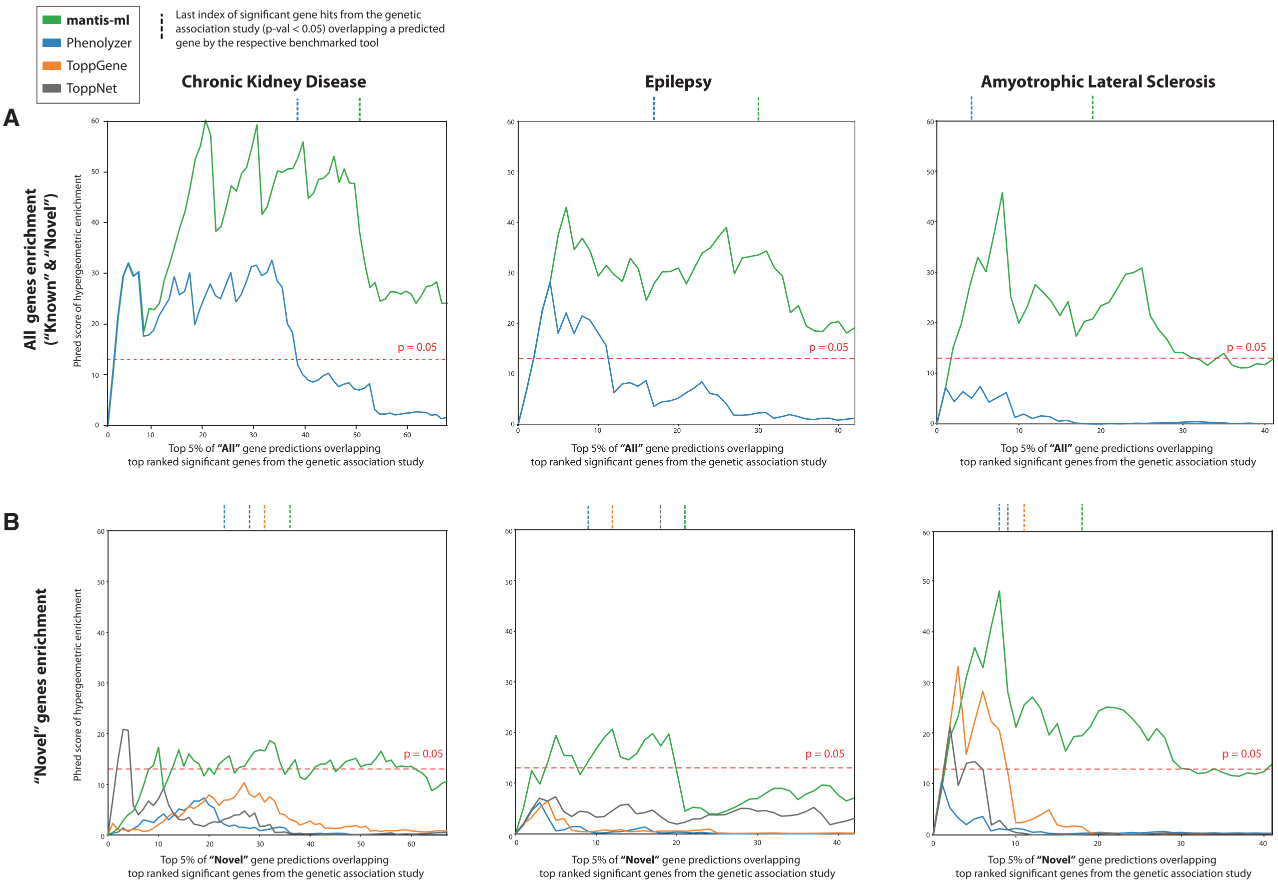

Figure 5. Benchmarking of Gene-Prioritization Tools Is Based on Enrichment of Their Top Predictions against Highly Ranked Genes from Rare-Variant Genetic-Association Studies across Three Disease Examples: CKD, Epilepsy, and ALS

(A and B) The enrichment signal for each tool is derived from a hypergeometric test between the significant hits ($p < 0.05$) from the genetic-association studies (looking into LoF variant enrichment) and the top 5% of "known" and "novel" gene predictions (A) or "novel" gene predictions only (B), from each of the benchmarked tools. The horizontal dashed red line corresponds to the significance threshold of $p = 0.05$ for the hypergeometric tests. The vertical dashed lines above each plot, which were colored on the basis of the respective benchmarked tool, indicate the last index of top-ranked genes that achieved a p value <0.05 in the collapsing analysis and overlapped the top 5% of gene predictions from that tool.

the disease-specific cases. We observe that mouse-model phenotypes ("MGI essential gene" and "essential mouse knockout"), protein expression (based on GTEx and Protein Atlas), protein-protein interaction features ("inferred seed genes overlap" and "experimental seed genes overlap") are still the top feature contributors as in CKD, epilepsy, and ALS (Figure 6A). Additionally, gene length, gene ontology features, and intolerance scores (RVIS and the scores based on GnomAD) rank highly in the normalized average Boruta score scale.

To validate the generic disease classifier results, we explored the ability of mantis-ml to correctly identify a set of known genes that has not been provided as part of the original seed gene set. Thus, we masked a random selection of 40% of the 4,041 seed genes (considered unlabeled) and then trained the generic mantis-ml algorithm by using gradient boosting as the standard classifier for $L = 5$ stochastic iterations. Notably, there was no significant difference between predictions of masked OMIM disease-associated genes and unmasked seed genes (Mann-Whitney U test p value = 0.119; Figure 6B). We explored the predictive power of GMS in this case in terms of distinguishing seed genes from unlabeled ones and retrieved AUC scores of 0.853 and 0.83 for unmasked and masked (hidden) seed genes, respectively (Figure 6C).

To avoid any over-prediction bias from the unmasked seed genes, we assessed the ability of GMS to efficiently stratify different OMIM- and MGI-based gene classes on the basis of prediction probabilities assigned to hidden seed genes only. The gene classes that we used have been defined in a previous work,[9] and the intersection with the hidden genes used in this case are as follows: OMIM dominant-negative genes (130), OMIM *de novo* and haploinsufficient genes (46), OMIM *de novo* genes (168), OMIM recessive genes (304), OMIM haploinsufficient genes (70), and MGI seizure orthologs (25). We also compiled the union of these gene classes as the OMIM_MGI_union set (510). We observed a high predictive power for GMS in this classification task; AUC scores ranged from 0.82–0.86 across the different OMIM or MGI gene sets and

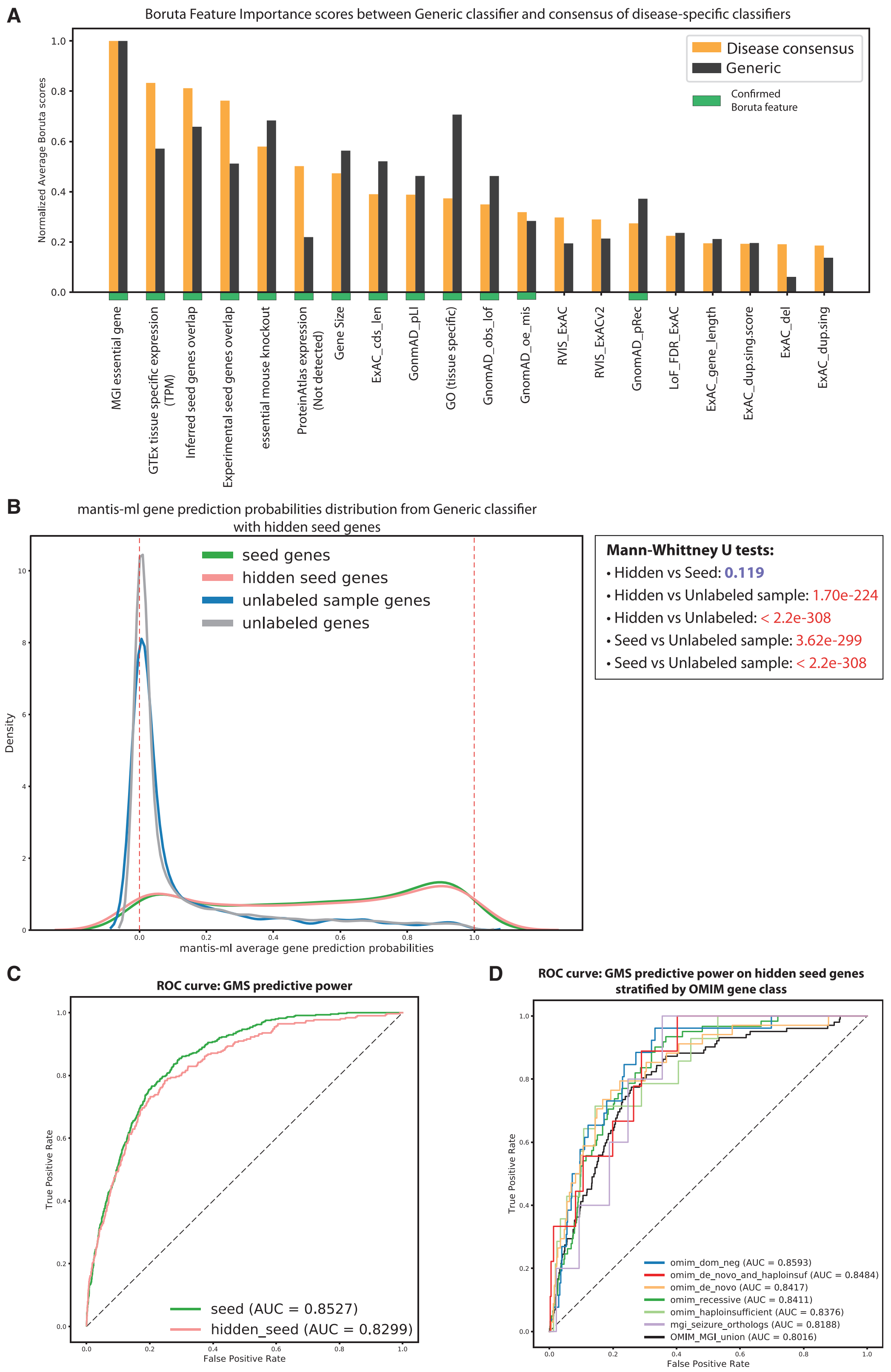

Figure 6. Generic Disease Mantis-ml Classifier for Estimation of Gene Disease Likelihood
(A) Comparison of consensus top feature contributors from CKD, epilepsy, and ALS with GMS feature-importance scores. The consensus of the disease-specific case is calculated as the mean of the normalized average Z scores returned by Boruta for each disease case.
(B) Generic mantis-ml prediction probabilities across different gene classes. The ranking was performed with 60% of the original seed genes set, and the other 40% of seed genes were treated as unlabeled. The unlabeled sample class represents a random sample from

(legend continued on next page)

was 0.80 of the union of these genes (Figure 6D). These scores are considerably higher than those from similar assessments using other metrics of genic intolerance: gnomAD_pLI, gnomAD_mis_z, RVIS_ExACv2, ExAC_cnv.score, and LoF_FDR_ExAC. The AUC performance of these scores in the task of OMIM/MGI versus non-OMIM/MGI classification (Figure S21) were in the range of 0.58–0.85 (gnomAD_pLI), 0.47–0.79 (gnomAD_mis_z), 0.47–0.70 (ExAC_cnv.score), 0.50–0.68 (RVIS_ExACv2), and 0.50–0.72 (LoF_FDR_ExAC). The respective AUC scores for the classification of the union of OMIM/MGI gene sets versus non-OMIM/MGI genes were in the range of 0.48–0.60 across all these metrics. It is, however, important to note that these component metrics are based on more specific datatypes, whereas mantis-ml leverages the information from a wider and more diverse collection of features. The ability of mantis-ml to correctly classify hidden seed genes underlines its power to subsequently correctly identify other unlabeled genes and provide a biologically meaningful ranking.

Finally, we explored how GMS would perform when overlapped with disease-specific rare-variant collapsing-analyses results. This exploration was similar to our above assessment where we used the disease-specific mantis-ml predictions (Figure S17). We noticed that there is still significant enrichment of the LoF signal in all three disease examples, but it is at a lower level than in the disease-specific cases (GMS ratios of LoF to synonymous area, 1.18, 0.81, 2.21; respective scores with disease-specific classifiers, 4.97, 33.95, not defined, as a result of the lack of a synonymous class in the ALS disease example). This highlights the added value of leveraging the disease-specific features to efficiently identify genes associated with a disease when such information (seed genes) is available.

Discussion

Presently, the genomics community is generating and analyzing large volumes of genomic data to better understand the genetic architecture of rare and common complex disorders. Here, we introduce a multi-dimensional machine-learning framework, mantis-ml, to support the triaging of the large-scale genome-wide readouts to further aid the prioritization of novel disease-associated genes. Mantis-ml takes its name from the Greek word *μάντης* which means “fortune teller.” Here, we have shown demonstrable predictive utility when combining mantis-ml with gene lists generated by large-scale association studies to enable a standardized and objective prioritization of genes for further functional validation in *in vitro* and *in vivo* models.

As with most machine-learning frameworks, one limitation of mantis-ml is its dependency on existing patterns. As such, mantis-ml is most powerful in identifying new disease-associated genes that might cause disease through an existing understood mechanism. Disease-associated genes representing an entirely unexpected disease mechanism might not be as highly prioritized. However, there might be opportunities to explore unsupervised approaches by clustering the results from t-SNE and UMAP (which are part of the mantis-ml processing workflow) to detect gene clusters that might not be well recognized. Moreover, the current mantis-ml package supports bespoke disease-specific features for CKD and cardiovascular disease (e.g., CKDdb, GOA, exSNP, etc.). These sets of features could be expanded for additional diseases via relevant data resources that might enable even more refined stratification of genes in other disease categories.

It will be interesting to explore how mantis-ml results validate against additional independent datasets that reflect gene rankings (e.g., CRISPR screens, GWAS etc.). For micro-array variant-level GWASs, one way to explore this would be to extract gene rankings on the basis of the lowest p value found for each gene. However, the mapping process is a challenge in this context because many of the variant signals from GWASs do not map to a single gene, especially in gene-dense regions of the genome, and thus, the confidence that a variant is mapped to the underlying biologically relevant gene can be low when the closest protein-coding gene is selected. Although this potential application on GWAS data is currently out of scope, we do provide the “mantisml-overlap” command line tool as part of the mantis-ml framework to enable users to validate the mantis-ml predictions against any other gene rankings (extracted e.g., from GWAS, CRISPR, or other high-throughput genetic screens).

Opportunities for future technical expansion of this approach include exploring integrating autoencoders for fully feature-agnostic dimensionality reduction as well as applying graph convolutional networks into a multiple kernel learning approach to better leverage information from multiple protein-protein interaction networks at the same time. Our framework could also extend to variant prioritization; however, this would require a revised design and a different collection of variant-level features.

We propose use of mantis-ml as an objective, standardized, fully quantitative and automated gene-prioritization tool for disease-specific or disease-agnostic studies. Additionally, we provide it as a complementary tool for the assessment of putative disease-associated genes extracted from completely orthogonal large-scale studies of human genetics thus reducing the required time for triaging top

the unlabeled genes of equal size to the set of hidden genes.. Mann-Whitney U tests were performed between the prediction-probability distributions of all pairs of gene classes (p values shown at the box on the right) to quantify their similarity degree.
(C) Predictive power of GMS to distinguish seed genes (unmasked and hidden) from unlabeled genes via a logistic regression classifier.
(D) Predictive power of GMS to distinguish different OMIM- and MGI-based hidden seed genes from unlabeled ones.

gene candidates from what is often a weeks to months-long process down to just a couple of hours.

Supplemental Data

Supplemental Data can be found online at https://doi.org/10.1016/j.ajhg.2020.03.012.

Acknowledgments

We thank Quanli Wang for useful discussions and his valuable feedback.

Declaration of Interests

The authors declare no competing interests. D.V. and S.P. report personal fees from AstraZeneca, during the conduct of the study. D.V.'s work was funded by the AstraZeneca post-doctorate program.

Received: January 28, 2020
Accepted: March 26, 2020
Published: May 7, 2020

Web Resources

The Mantis-ml Tool, https://github.com/astrazeneca-cgr-publications/mantis-ml-release

The Mantis-ml Gene Prioritization Atlas, https://dvitsios.github.io/mantis-ml-predictions

References

1. Zitnik, M., Nguyen, F., Wang, B., Leskovec, J., Goldenberg, A., and Hoffman, M.M. (2019). Machine learning for integrating data in biology and medicine: Principles, practice, and opportunities. Inf. Fusion *50*, 71–91.
2. Ritchie, M.D., Holzinger, E.R., Li, R., Pendergrass, S.A., and Kim, D. (2015). Methods of integrating data to uncover genotype-phenotype interactions. Nat. Rev. Genet. *16*, 85–97.
3. Karczewski, K.J., and Snyder, M.P. (2018). Integrative omics for health and disease. Nat. Rev. Genet. *19*, 299–310.
4. GTEx Consortium (2015). The Genotype-Tissue Expression (GTEx) pilot analysis: Multitissue gene regulation in humans. Science *80*, 648–660.
5. Köhler, S., Vasilevsky, N.A., Engelstad, M., Foster, E., McMurry, J., Aymé, S., Baynam, G., Bello, S.M., Boerkoel, C.F., Boycott, K.M., et al. (2017). The human phenotype ontology in 2017. Nucleic Acids Res. *45* (D1), D865–D876.
6. Smith, C.L., Blake, J.A., Kadin, J.A., Richardson, J.E., Bult, C.J.; and Mouse Genome Database Group (2018). Mouse Genome Database (MGD)-2018: knowledgebase for the laboratory mouse. Nucleic Acids Res. *46* (D1), D836–D842.
7. Uhlén, M., Fagerberg, L., Hallström, B.M., Lindskog, C., Oksvold, P., Mardinoglu, A., Sivertsson, Å., Kampf, C., Sjöstedt, E., Asplund, A., et al. (2015). Tissue-based map of the human proteome. Science *80*, 347.
8. Li, T., Wernersson, R., Hansen, R.B., Horn, H., Mercer, J., Slodkowicz, G., Workman, C.T., Rigina, O., Rapacki, K., Stærfeldt, H.H., et al. (2017). A scored human protein-protein interaction network to catalyze genomic interpretation. Nat. Methods *14*, 61–64.
9. Petrovski, S., Wang, Q., Heinzen, E.L., Allen, A.S., and Goldstein, D.B. (2013). Genic intolerance to functional variation and the interpretation of personal genomes. PLoS Genet. *9*, e1003709.
10. Traynelis, J., Silk, M., Wang, Q., Berkovic, S.F., Liu, L., Ascher, D.B., Balding, D.J., and Petrovski, S. (2017). Optimizing genomic medicine in epilepsy through a gene-customized approach to missense variant interpretation. Genome Res. *10*, 1715–1729.
11. Lek, M., Karczewski, K.J., Minikel, E.V., Samocha, K.E., Banks, E., Fennell, T., O'Donnell-Luria, A.H., Ware, J.S., Hill, A.J., Cummings, B.B., et al.; Exome Aggregation Consortium (2016). Analysis of protein-coding genetic variation in 60,706 humans. Nature *536*, 285–291.
12. Povysil, G., Petrovski, S., Hostyk, J., Aggarwal, V., Allen, A.S., and Goldstein, D.B. (2019). Rare-variant collapsing analyses for complex traits: guidelines and applications. Nat. Rev. Genet. *20*, 747–759.
13. Cirulli, E.T., Lasseigne, B.N., Petrovski, S., Sapp, P.C., Dion, P.A., Leblond, C.S., Couthouis, J., Lu, Y.F., Wang, Q., Krueger, B.J., et al. (2015). Exome sequencing in amyotrophic lateral sclerosis identifies risk genes and pathways. Science *80*, 1436–1441.
14. Cameron-Christie, S., Wolock, C.J., Groopman, E., Petrovski, S., Kamalakaran, S., Povysil, G., Vitsios, D., Zhang, M., Fleckner, J., March, R.E., et al. (2019). Exome-Based Rare-Variant Analyses in CKD. J. Am. Soc. Nephrol. *30*, 1109–1122.
15. Epi4K consortium; and Epilepsy Phenome/Genome Project (2017). Ultra-rare genetic variation in common epilepsies: a case-control sequencing study. Lancet Neurol. *16*, 135–143.
16. van der Maaten, L., and Hinton, G. (2008). Visualizing Data using t-SNE. Journal of Machine Learning Research,.
17. McInnes, L., Healy, J., Saul, N., and Großberger, L. (2018). UMAP: Uniform Manifold Approximation and Projection. The Journal of Open Source Software *29*, 861.
18. Elkan, C., and Noto, K. (2008). Learning classifiers from only positive and unlabeled data KDD '08: Proceedings of the 14th ACM SIGKDD International Conference on Knowledge Discovery and Data Mining (Association for Computing Machinery), pp. 213–220.
19. Mordelet, F., and Vert, J.P. (2014). A bagging SVM to learn from positive and unlabeled examples. Pattern Recognition Letters,.
20. Kaboutari, A., Bagherzadeh, J., and Kheradmand, F. (2014). An Evaluation of Two-Step Techniques for Positive-Unlabeled Learning in Text Classification. International Journal of Computer Applications Technology and Research *3*, 592–594.
21. Kursa, M.B., and Rudnicki, W.R. (2010). Feature selection with the boruta package. Journal of Statistical Software,.
22. Petrovski, S., Todd, J.L., Durheim, M.T., Wang, Q., Chien, J.W., Kelly, F.L., Frankel, C., Mebane, C.M., Ren, Z., Bridgers, J., et al. (2017). An exome sequencing study to assess the role of rare genetic variation in pulmonary fibrosis. Am. J. Respir. Crit. Care Med. *196*, 82–93.
23. Voskarides, K., Papagregoriou, G., Hadjipanagi, D., Petrou, I., Savva, I., Elia, A., Athanasiou, Y., Pastelli, A., Kkolou, M., Hadjigavriel, M., et al. (2018). COL4A5 and LAMA5 variants co-inherited in familial hematuria: digenic inheritance or genetic modifier effect? BMC Nephrol. *19*, 114.
24. Braun, D.A., Warejko, J.K., Ashraf, S., Tan, W., Daga, A., Schneider, R., et al. (2018). Genetic variants in the LAMA5

gene in pediatric nephrotic syndrome. Nephrology Dialysis Transplantation *34*, 485–493.
25. Prikhodina, L., Lebedenkova, M., Papizh, S., and Shatalov, P. (2017). MP837LAMA5 variants in childhood steroid-resistant nephrotic syndrome: candidate gene mutations or incidental findings? Nephrology Dialysis Transplantation,.
26. Baylis, C. (2007). Nitric oxide deficiency in chronic kidney disease. Am. J. Physiol.
27. Ramanathan, G., Periyasamy, S., and Lakkakula, B.V. (2014). NOS3 tagSNPs does not modify the chronic kidney disease progression in autosomal dominant polycystic kidney disease. Nephrology (Carlton) *19*, 537–541.
28. Chand, S., Chue, C.D., Edwards, N.C., Hodson, J., Simmonds, M.J., Hamilton, A., Gough, S.C., Harper, L., Steeds, R.P., Townend, J.N., et al. (2015). Endothelial nitric oxide synthase single nucleotide polymorphism and left ventricular function in early chronic kidney disease. PLoS ONE *10*, e0116160.
29. Nagase, S., Suzuki, H., Wang, Y., Kikuchi, S., Hirayama, A., Ueda, A., Takada, K., Oteki, T., Obara, M., Aoyagi, K., and Koyama, A. (2003). Association of ecNOS gene polymorphisms with end stage renal diseases. Mol. Cell. Biochem. *244*, 113–118.
30. Wuttke, M., Wong, C.S., Wühl, E., Epting, D., Luo, L., Hoppmann, A., Doyon, A., Li, Y., Sözeri, B., Thurn, D., et al.; CKDGen Consortium (2016). Genetic loci associated with renal function measures and chronic kidney disease in children: the Pediatric Investigation for Genetic Factors Linked with Renal Progression Consortium. Nephrol. Dial. Transplant. *31*, 262–269.
31. Li, J., Lu, D., Liu, H., Williams, B.O., Overbeek, P.A., Lee, B., Zheng, L., and Yang, T. (2017). Sclt1 deficiency causes cystic kidney by activating ERK and STAT3 signaling. Hum. Mol. Genet. *26*, 2949–2960.
32. Chen, L., Yang, T., Lu, D.W., Zhao, H., Feng, Y.L., Chen, H., Chen, D.Q., Vaziri, N.D., and Zhao, Y.Y. (2018). Central role of dysregulation of TGF-β/Smad in CKD progression and potential targets of its treatment. Biomed. Pharmacother. *101*, 670–681.
33. Inoue, K., Balkin, D.M., Liu, L., Nandez, R., Wu, Y., Tian, X., Wang, T., Nussbaum, R., De Camilli, P., and Ishibe, S. (2017). Kidney Tubular Ablation of *Ocrl*/*Inpp5b* Phenocopies Lowe Syndrome Tubulopathy. J. Am. Soc. Nephrol. *28*, 1399–1407.
34. Nezu, M., Suzuki, N., and Yamamoto, M. (2017). Targeting the KEAP1-NRF2 System to Prevent Kidney Disease Progression. Am. J. Nephrol. *45*, 473–483.
35. Noebels, J.L. (2013). The Voltage-Gated Calcium Channel and Absence Epilepsy. In Jasper's Basic Mechanisms of the Epilepsies, J.L. Noebels, M. Avoli, M.A. Rogawski, R.W. Olsen, and A.V. Delgado-Escueta, eds. (National Center for Biotechnology Information).
36. Heyes, S., Pratt, W.S., Rees, E., Dahimene, S., Ferron, L., Owen, M.J., and Dolphin, A.C. (2015). Genetic disruption of voltage-gated calcium channels in psychiatric and neurological disorders. Prog. Neurobiol. *134*, 36–54.
37. Wilson, S.M., et al. (2012). Inhibition of transmitter release and attenuation of anti-retroviral- associated and tibial nerve injury-related painful peripheral neuropathy by novel synthetic Ca2+ channel peptides. J. Biol. Chem. *287*, 35065–35077.
38. Sirito, M., Lin, Q., Deng, J.M., Behringer, R.R., and Sawadogo, M. (1998). Overlapping roles and asymmetrical cross-regulation of the USF proteins in mice. Proc. Natl. Acad. Sci. USA *95*, 3758–3763.
39. Wijayatunge, R., Chen, L.F., Cha, Y.M., Zannas, A.S., Frank, C.L., and West, A.E. (2014). The histone lysine demethylase Kdm6b is required for activity-dependent preconditioning of hippocampal neuronal survival. Mol. Cell. Neurosci. *61*, 187–200.
40. Lindgren, A.M., Hoyos, T., Talkowski, M.E., Hanscom, C., Blumenthal, I., Chiang, C., Ernst, C., Pereira, S., Ordulu, Z., Clericuzio, C., et al. (2013). Haploinsufficiency of KDM6A is associated with severe psychomotor retardation, global growth restriction, seizures and cleft palate. Hum. Genet. *132*, 537–552.
41. Lopez, A.Y., Wang, X., Xu, M., Maheshwari, A., Curry, D., Lam, S., Adesina, A.M., Noebels, J.L., Sun, Q.Q., and Cooper, E.C. (2017). Ankyrin-G isoform imbalance and interneuronopathy link epilepsy and bipolar disorder. Mol. Psychiatry *22*, 1464–1472.
42. Iqbal, Z., Vandeweyer, G., van der Voet, M., Waryah, A.M., Zahoor, M.Y., Besseling, J.A., Roca, L.T., Vulto-van Silfhout, A.T., Nijhof, B., Kramer, J.M., et al. (2013). Homozygous and heterozygous disruptions of ANK3: at the crossroads of neurodevelopmental and psychiatric disorders. Hum. Mol. Genet. *22*, 1960–1970.
43. Lehman, A., Thouta, S., Mancini, G.M.S., Naidu, S., van Slegtenhorst, M., McWalter, K., Person, R., Mwenifumbo, J., Salvarinova, R., Guella, I., et al.; CAUSES Study; and EPGEN Study (2017). Loss-of-Function and Gain-of-Function Mutations in KCNQ5 Cause Intellectual Disability or Epileptic Encephalopathy. Am. J. Hum. Genet. *101*, 65–74.
44. Mademan, I., Harmuth, F., Giordano, I., Timmann, D., Magri, S., Deconinck, T., Claaßen, J., Jokisch, D., Genc, G., Di Bella, D., et al. (2016). Multisystemic SYNE1 ataxia: confirming the high frequency and extending the mutational and phenotypic spectrum. Brain *139*, e46.
45. Yoshinaga, T., Nakamura, K., Ishikawa, M., Yamaguchi, T., Takano, K., Wakui, K., Kosho, T., Yoshida, K., Fukushima, Y., and Sekijima, Y. (2017). A novel frameshift mutation of *SYNE1* in a Japanese family with autosomal recessive cerebellar ataxia type 8. Hum. Genome Var. *4*, 17052.
46. Bergemalm, D., Forsberg, K., Jonsson, P.A., Graffmo, K.S., Brännström, T., Andersen, P.M., Antti, H., and Marklund, S.L. (2009). Changes in the spinal cord proteome of an amyotrophic lateral sclerosis murine model determined by differential in-gel electrophoresis. Mol. Cell. Proteomics *8*, 1306–1317.
47. Andrés-Benito, P., Moreno, J., Aso, E., Povedano, M., and Ferrer, I. (2017). Amyotrophic lateral sclerosis, gene deregulation in the anterior horn of the spinal cord and frontal cortex area 8: implications in frontotemporal lobar degeneration. Aging (Albany N.Y.) *9*, 823–851.
48. Martin, L.J., and Wong, M. (2013). Aberrant regulation of DNA methylation in amyotrophic lateral sclerosis: a new target of disease mechanisms. Neurotherapeutics *10*, 722–733.
49. Yang, H., Robinson, P.N., and Wang, K. (2015). Phenolyzer: phenotype-based prioritization of candidate genes for human diseases. Nat. Methods *12*, 841–843.
50. Chen, J., Bardes, E.E., Aronow, B.J., and Jegga, A.G. (2009). ToppGene Suite for gene list enrichment analysis and candidate gene prioritization. Nucleic Acids Res. *37*, W305-11.
51. Chen, J., Aronow, B.J., and Jegga, A.G. (2009). Disease candidate gene identification and prioritization using protein interaction networks. BMC Bioinformatics *10*, 73.

The American Journal of Human Genetics, Volume *106*

Supplemental Data

Mantis-ml: Disease-Agnostic Gene Prioritization from High-Throughput Genomic Screens by Stochastic Semi-supervised Learning

Dimitrios Vitsios and Slavé Petrovski

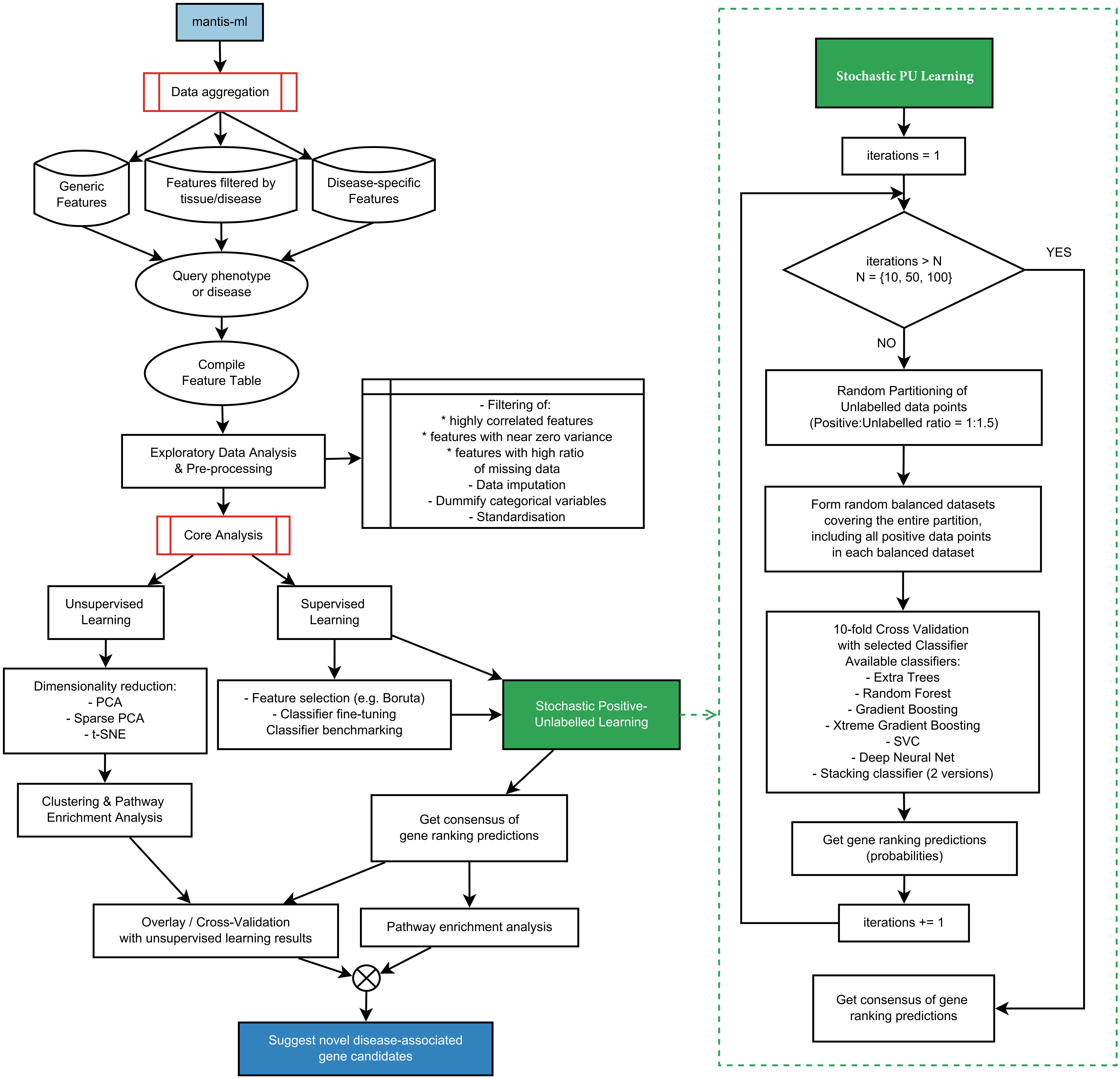

Figure S1. mantis-ml flowchart diagram visualizing the steps for data aggregation, pre-processing and the core analysis, including a detailed overview of the stochastic positive-unlabeled learning implementation.

mantis-ml pre-processing and exploratory analysis (CKD example)

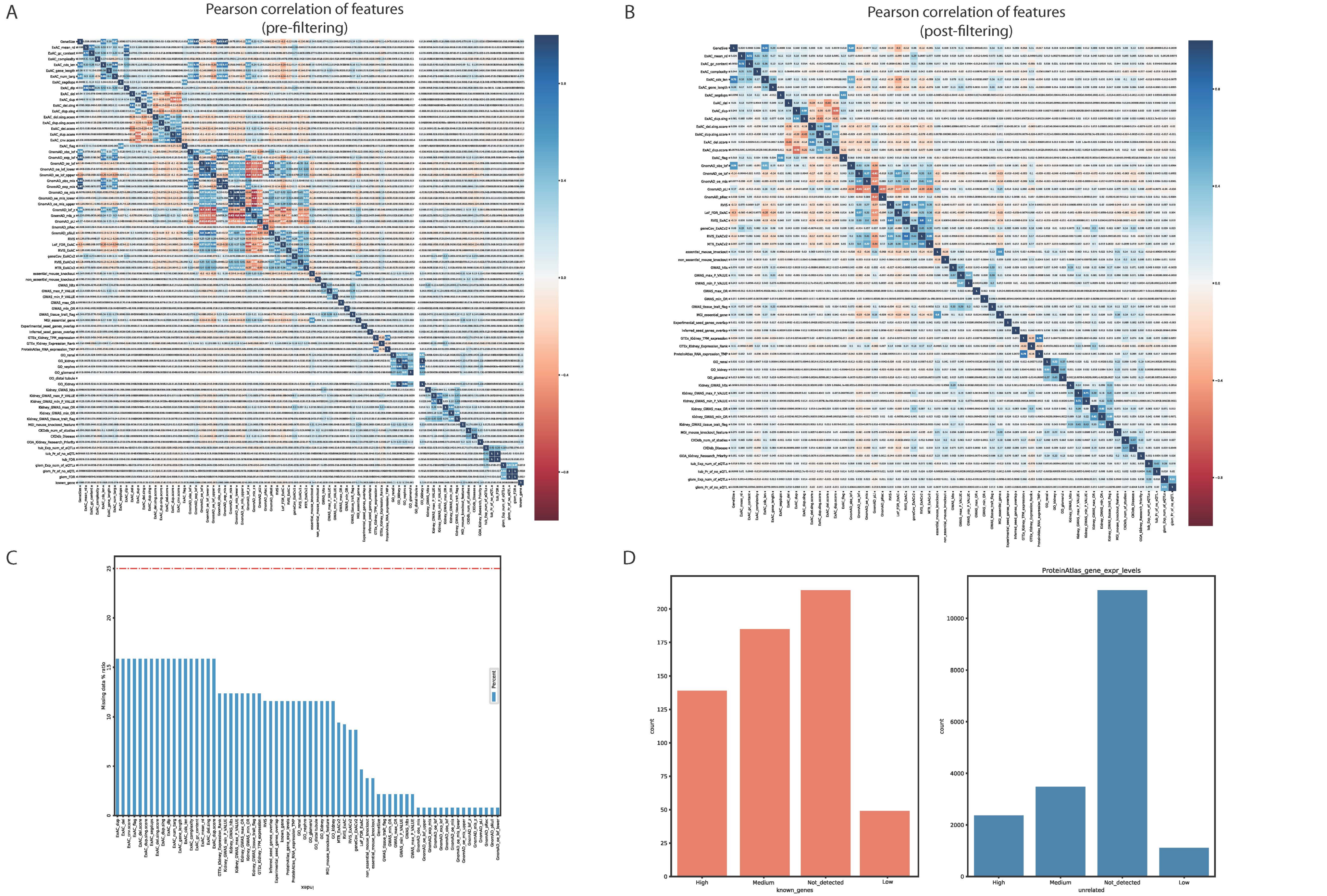

Figure S2. Data pre-processing and exploratory data analysis in Chronic Kidney Disease case: A) Pairwise feature correlation **before** filtering of highly-correlated features, B) Pairwise feature correlation **after** filtering of highly-correlated features, C) Missing data ratios and D) distribution of categorical feature profiles in positive and unlabelled genes.

Exploratory analysis - numerical features distribution

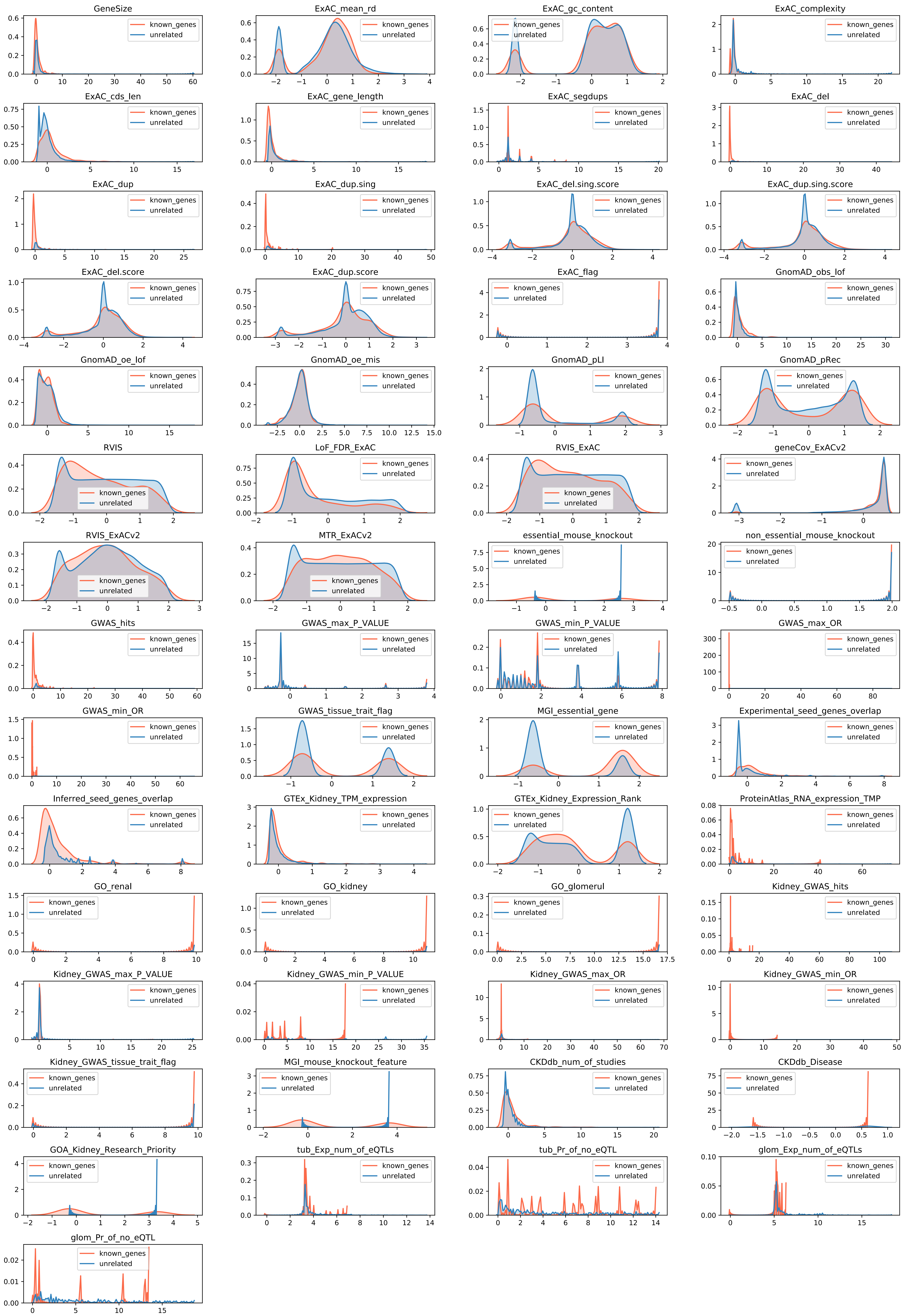

Figure S3. Exploratory data analysis in Chronic Kidney Disease case: distribution of numerical feature profiles in positive (known) and unlabelled genes.

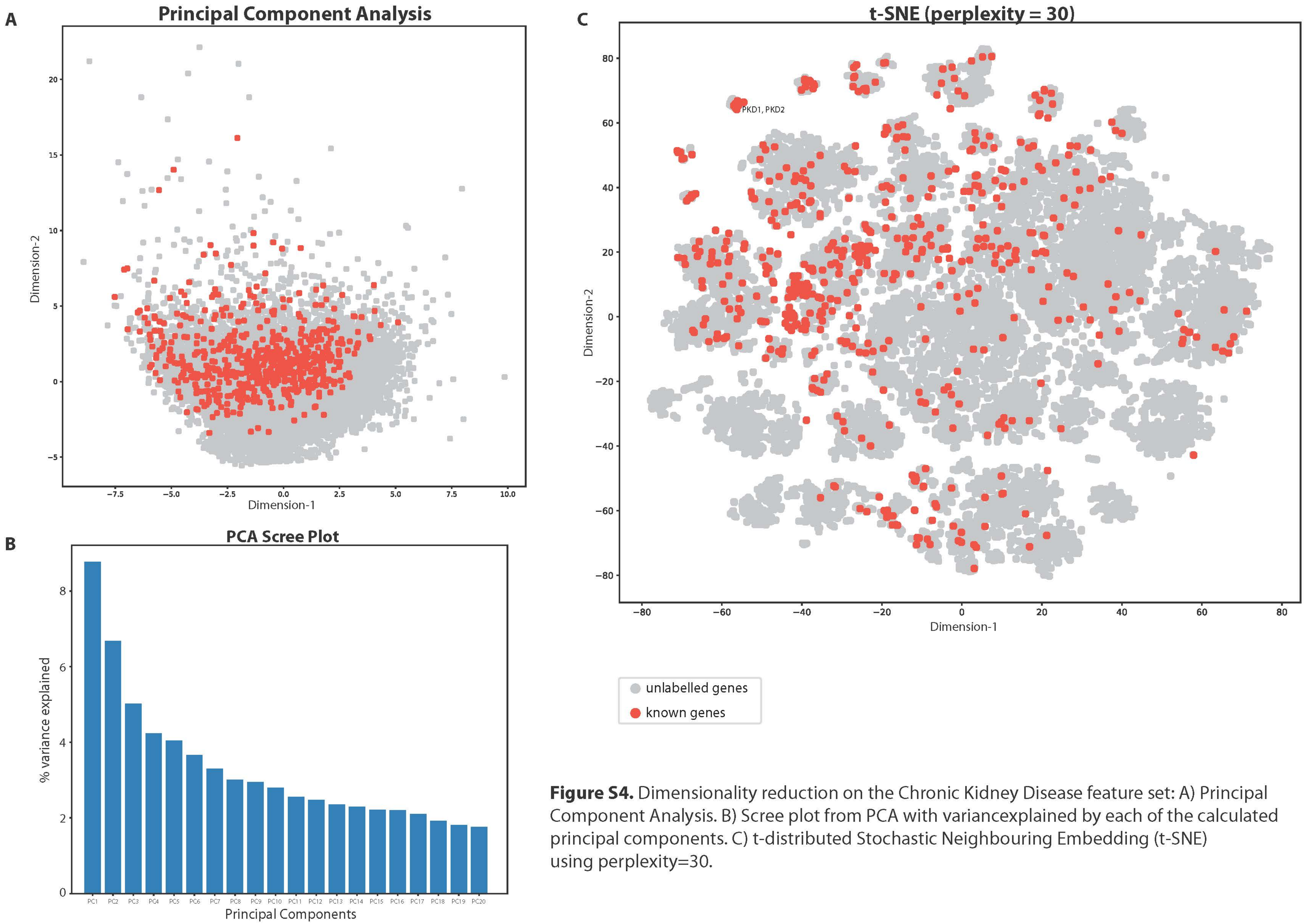

Figure S4. Dimensionality reduction on the Chronic Kidney Disease feature set: A) Principal Component Analysis. B) Scree plot from PCA with variancexplained by each of the calculated principal components. C) t-distributed Stochastic Neighbouring Embedding (t-SNE) using perplexity=30.

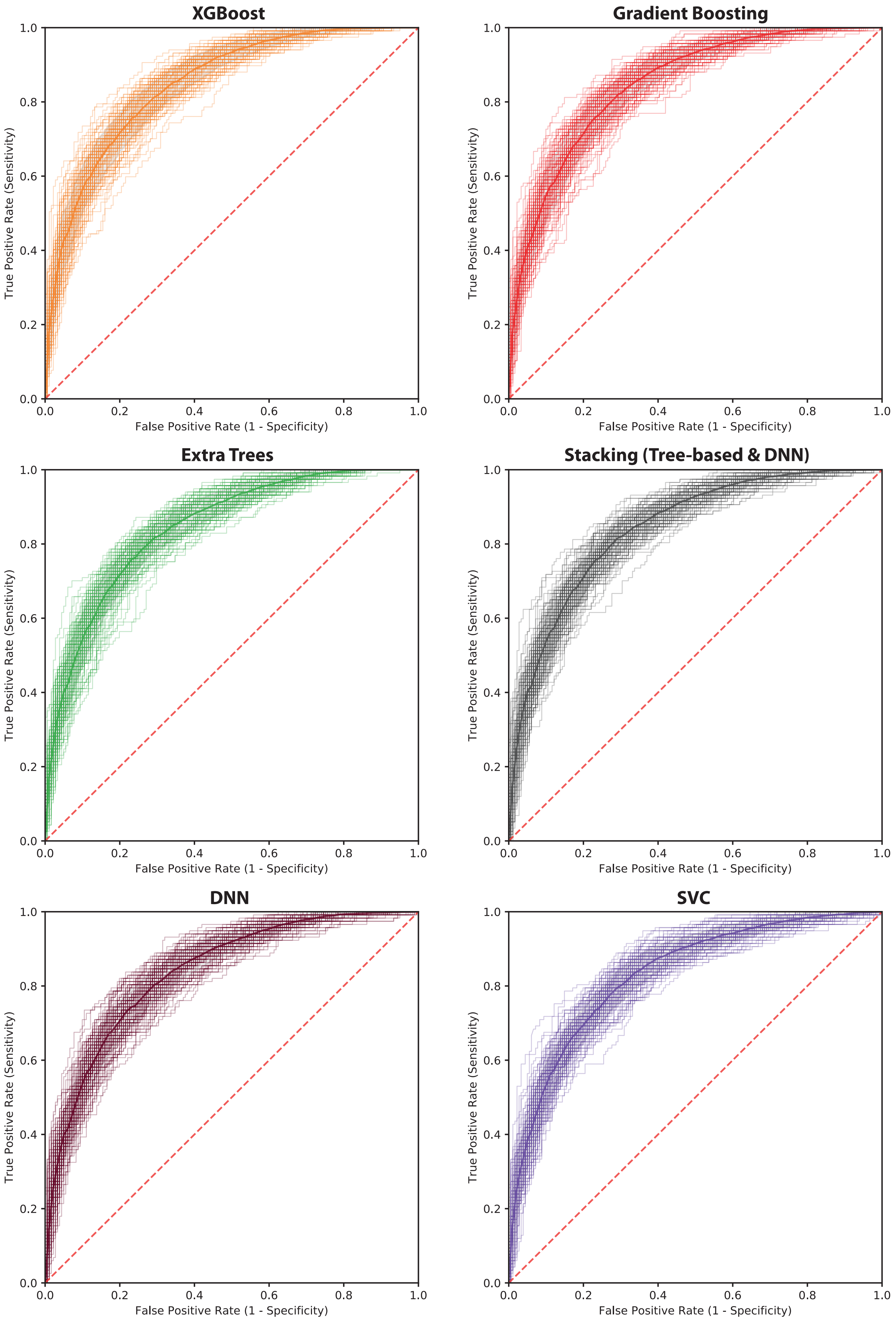

Figure S5. ROC curves from 10 batches of 10-fold Cross Validation with 6 different classiffers, in decreasing order of mean AUC: a) Random Forest, b) Xtreme Gradient Boosting, c) Gradient Boosting, d) Extra Trees, e) Stacking Classifier (1st layer: Extra Trees + Random Forest + Gradient Boosting + SVC; 2nd layer: DNN), f) Deep Neural Net (2-hidden layers) and g) Support Vector Classiffer.

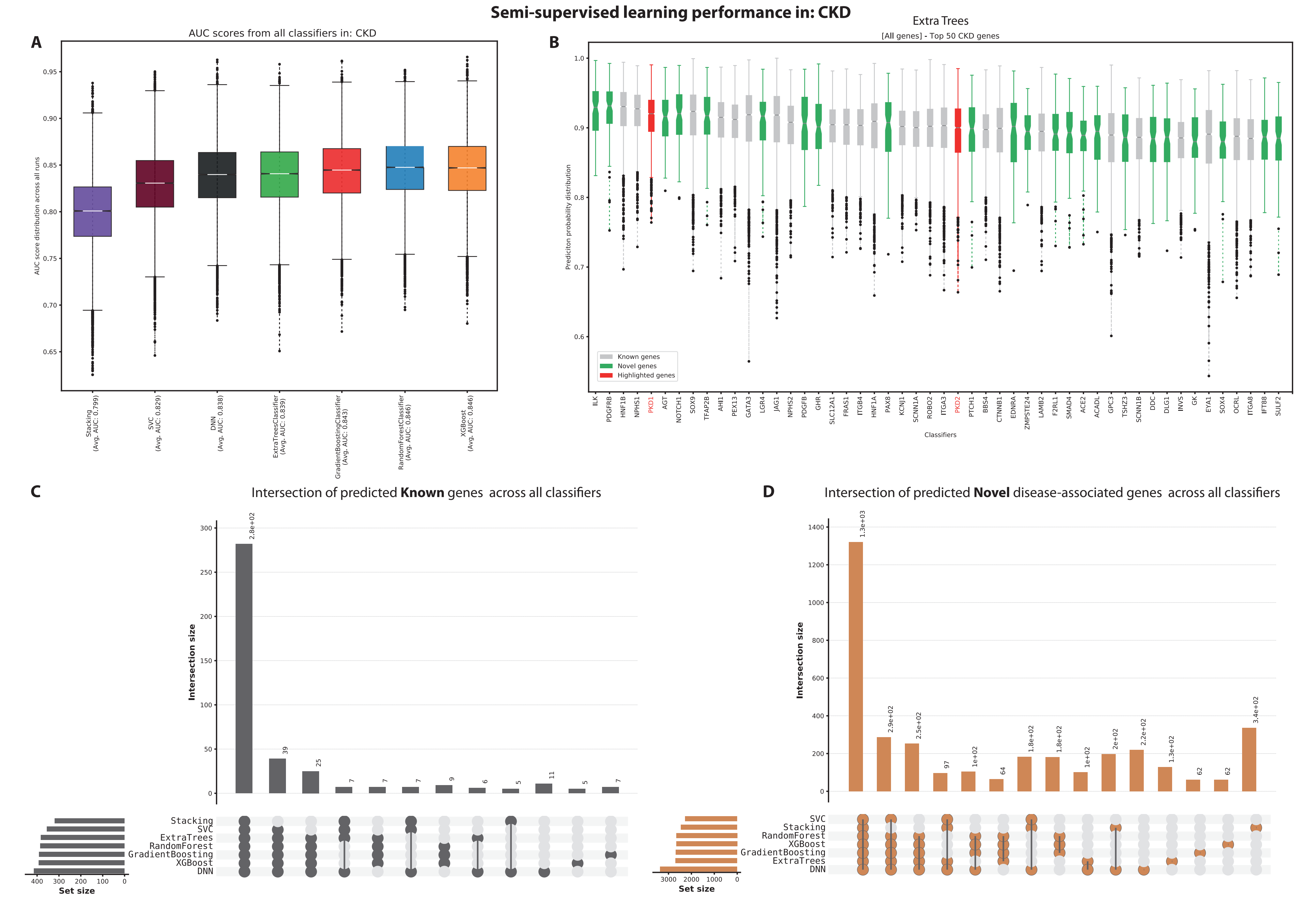

Figure S6. Mantis-ml performance on the Chronic Kidney Disease case. A) AUC score distribution per standard classifier used during mantis-ml training. B) Prediction probabilities from the top 50 (known and novel) genes predicted with Extra Trees as the standard classifier. C/D) Intersection sets of predicted known/novel genes across all classifiers.

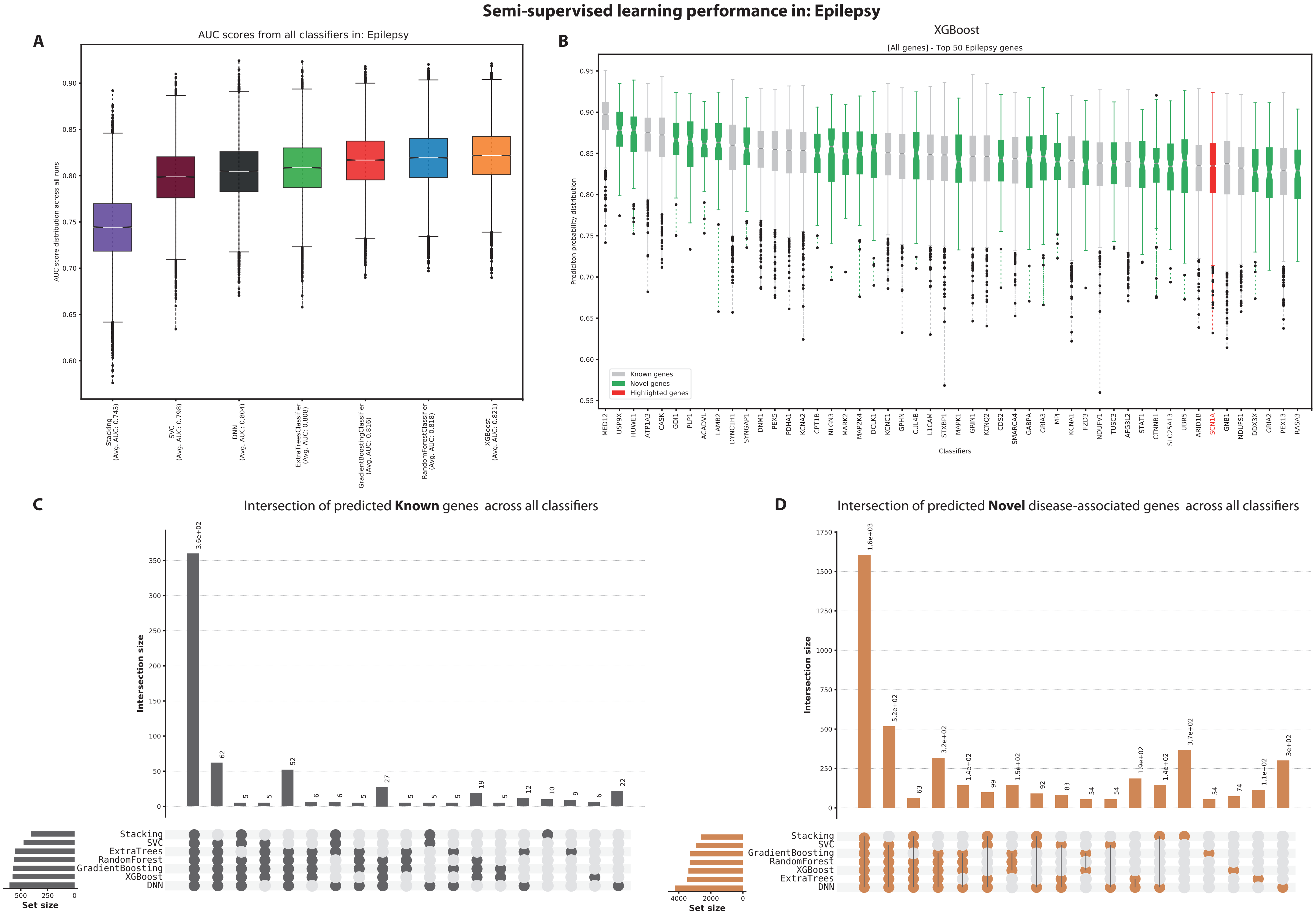

Figure S7. Mantis-ml performance on the Epilpesy disease case. A) AUC score distribution per standard classifier used during mantis-ml training. B) Prediction probabilities from the top 50 (known and novel) genes predicted with XGBoost as the standard classifier. C/D) Intersection sets of predicted known/novel genes across all classifiers.

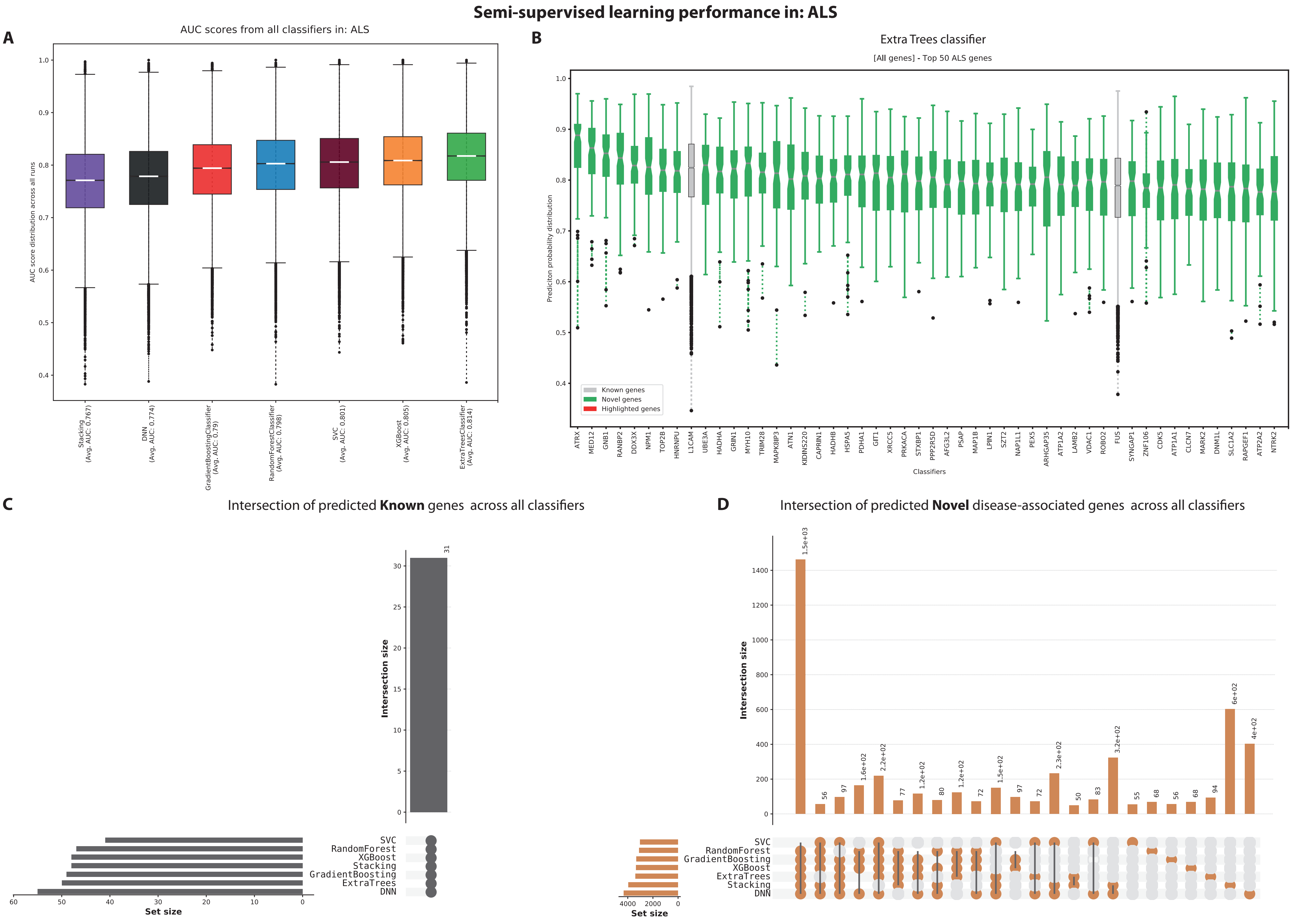

Figure S8. Mantis-ml performance on the ALS disease case. A) AUC score distribution per standard classifier used during mantis-ml training. B) Prediction probabilities from the top 50 (known and novel) genes predicted with Extra Trees as the standard classifier. C/D) Intersection sets of predicted known/novel genes across all classifiers.

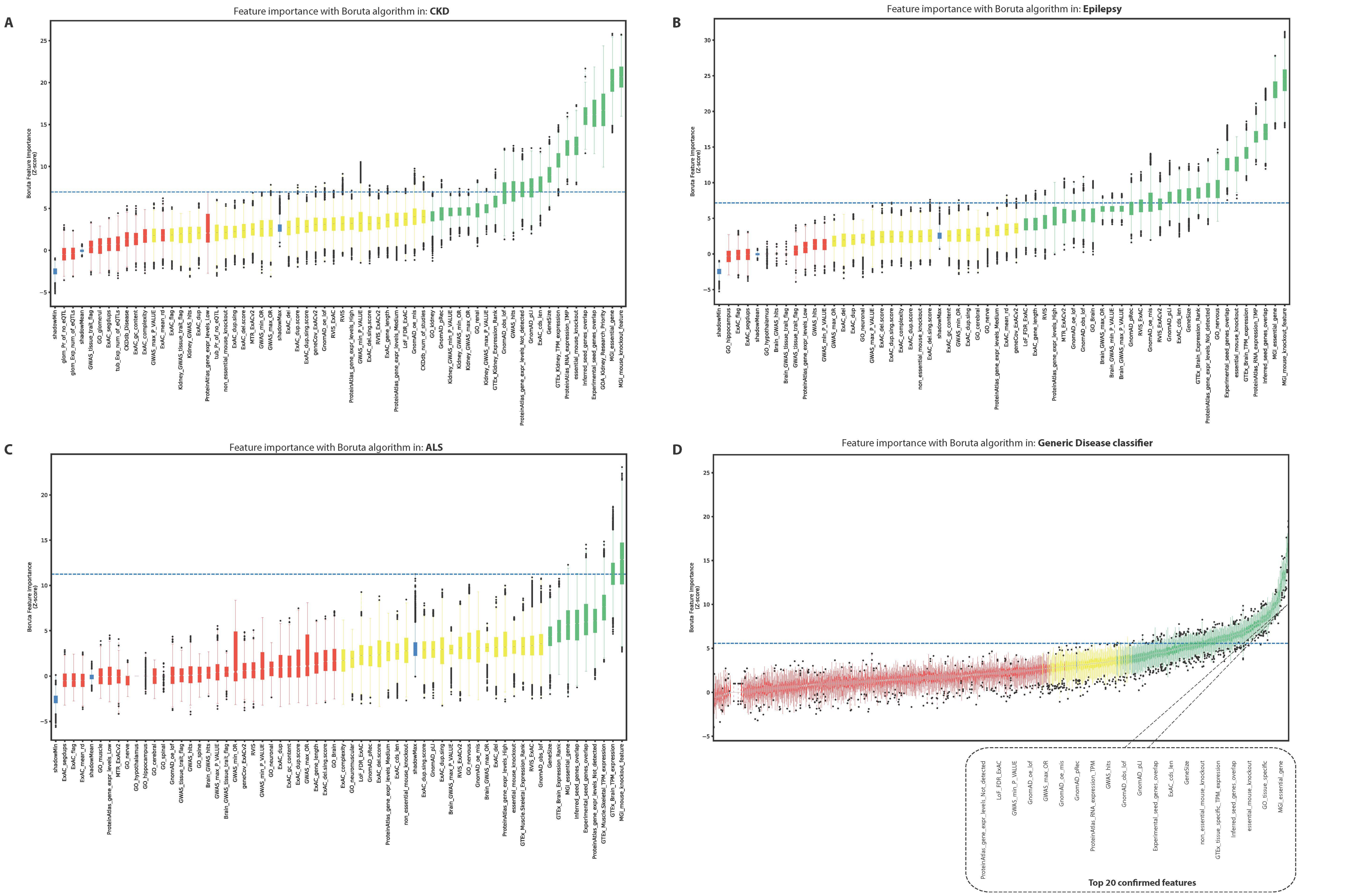

Figure S9. Distribution of feature importance scores extracted by a Random Forest classifier with the Boruta algorithm. Predictions are extracted across 100 balanced gene subsets with 10-fold cross-validation for the Chronic Kidney Disease (A), Epilepsy (B) and Amyotrophic Lateral Sclerosis (C) cases and for 1 balanced dataset with 10-fold cross validation for the Generic classifier (D). Confirmed features are shown in green, tentative in yellow and rejected ones with red. The random permuted features that are calculated as references by Boruta are shown in blue ('shadow' features). The top 20 confirmed features are shown for the Generic classifier.

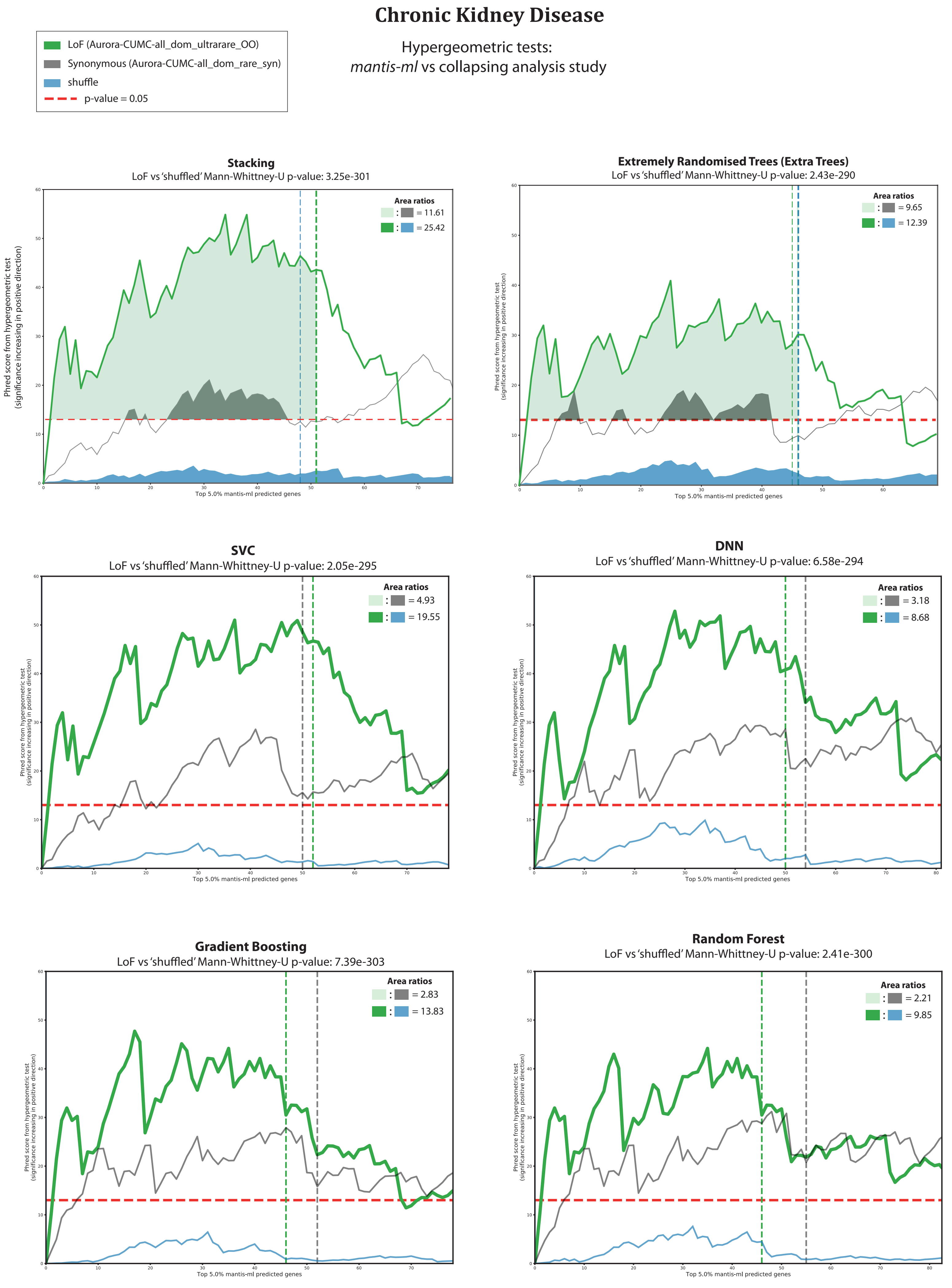

Figure S10. Cross-validation of mantis-ml predictions per classifier with rare-variant collapsing analysis results (applied for the Chronic Kidney Disease example)

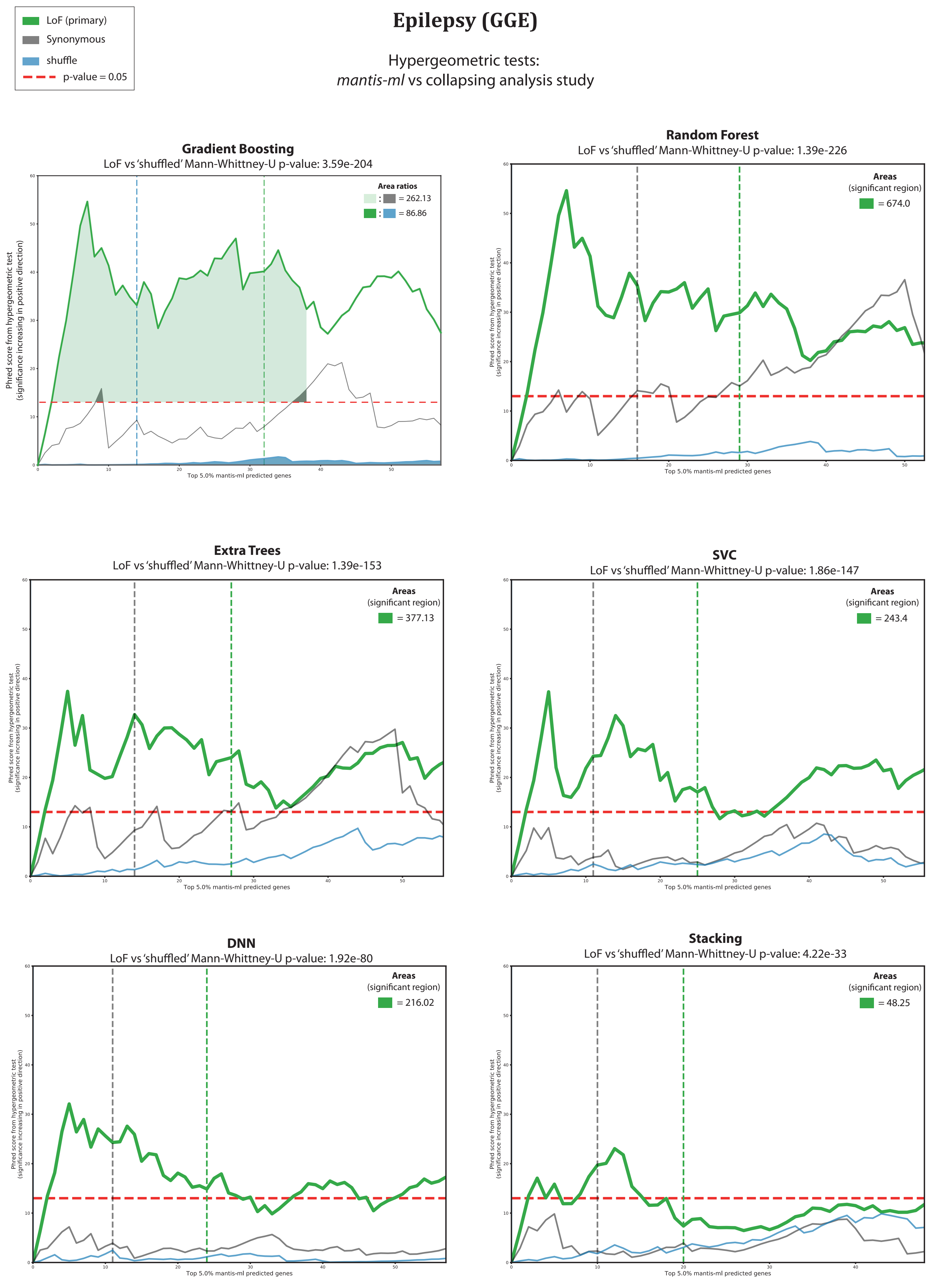

Figure S11. Cross-validation of mantis-ml predictions per classifier with rare-variant collapsing analysis results (applied for the Epilepsy disease example)

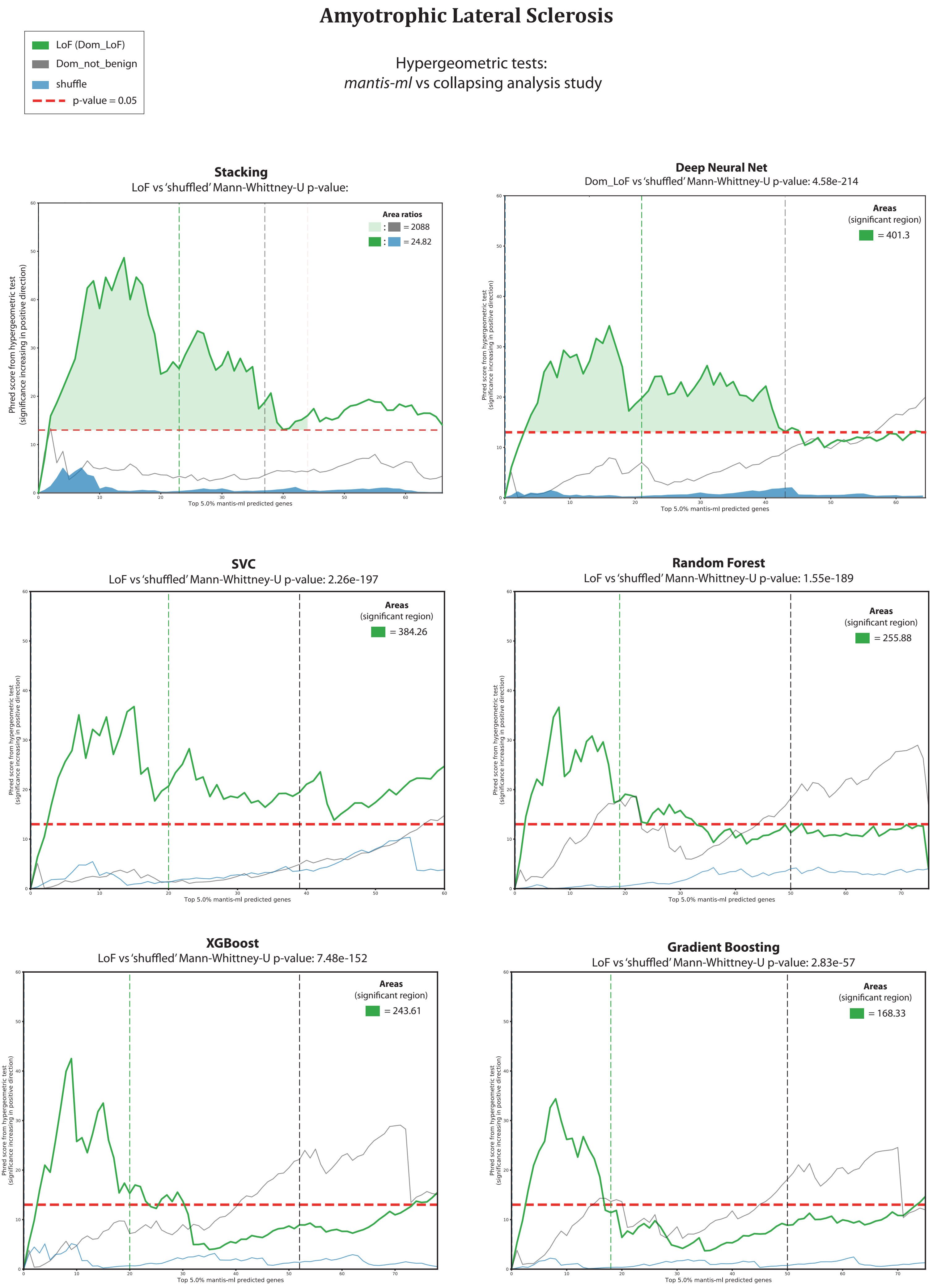

Figure S12. Cross-validation of mantis-ml predictions per classifier with rare-variant collapsing analysis results (applied for the ALS disease exampleh)

Consensus of mantis-ml 'known gene' predictions after overlap with collapsing results

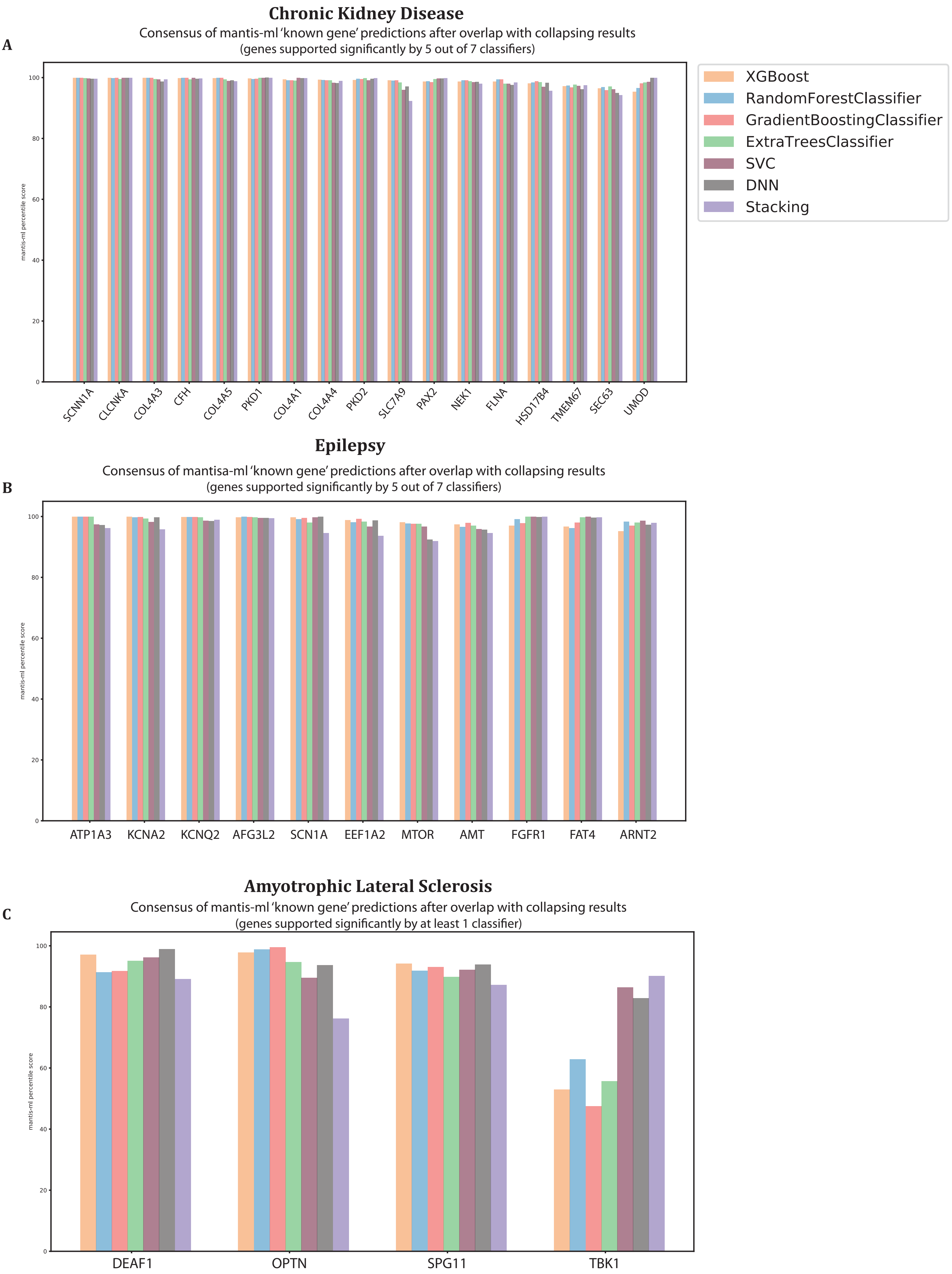

Figure S13. Consensus known genes across three disease cases: CKD (A), Epilepsy (B) and ALS (C), satisfying the significance threshold criteria in both the collapsing analysis results and the hypergeometric, supported by multiple numbers of classifiers used by mantis-ml.

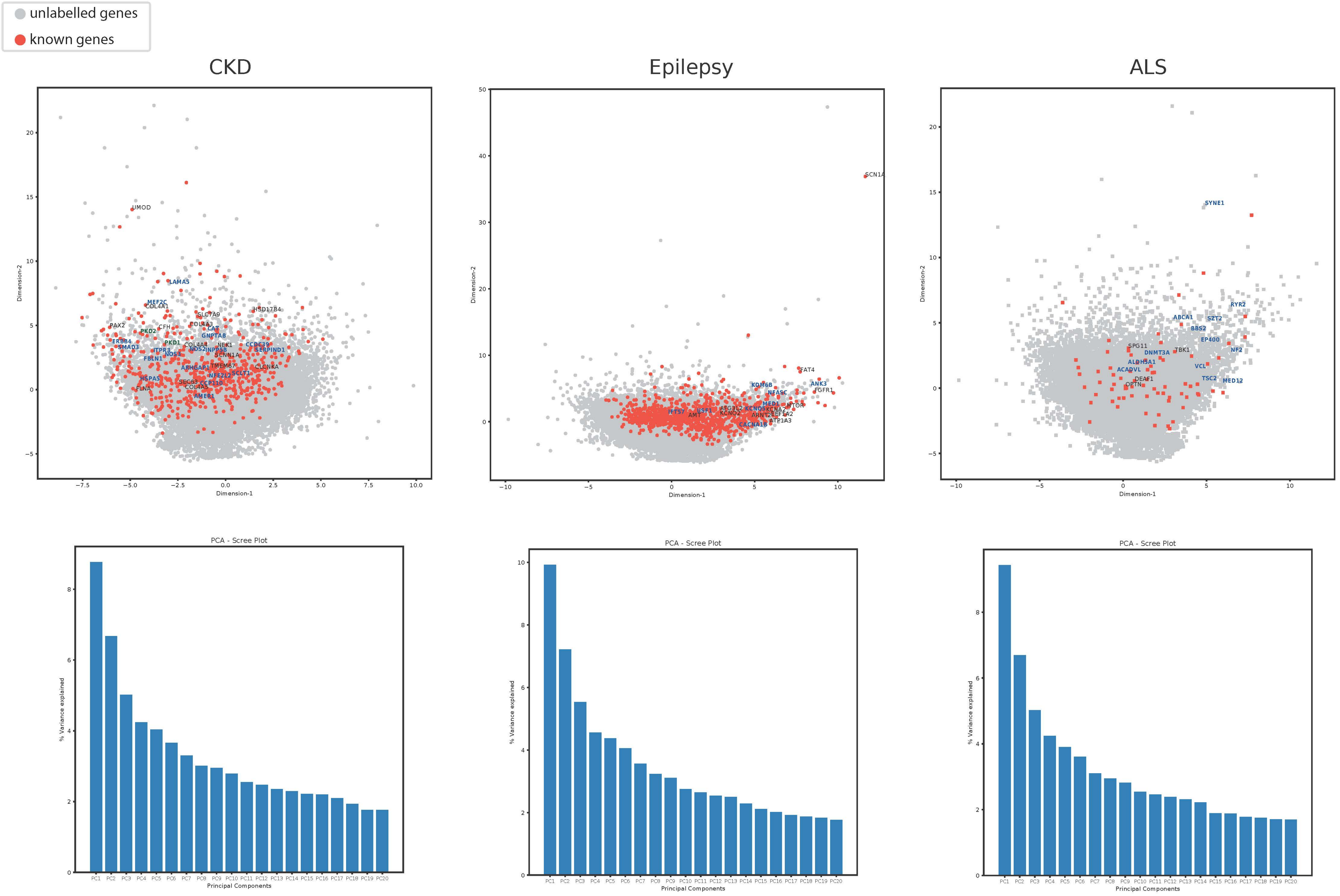

Figure S14. Principal Component Analysis (PCA) plots and Scree plots (variance explained by PCA components) for each disease example. Labelled genes are all the consensus novel (dark blue) and known genes predicted by overlapping mantis-ml predictions with rare-variant collapsing analysis results.

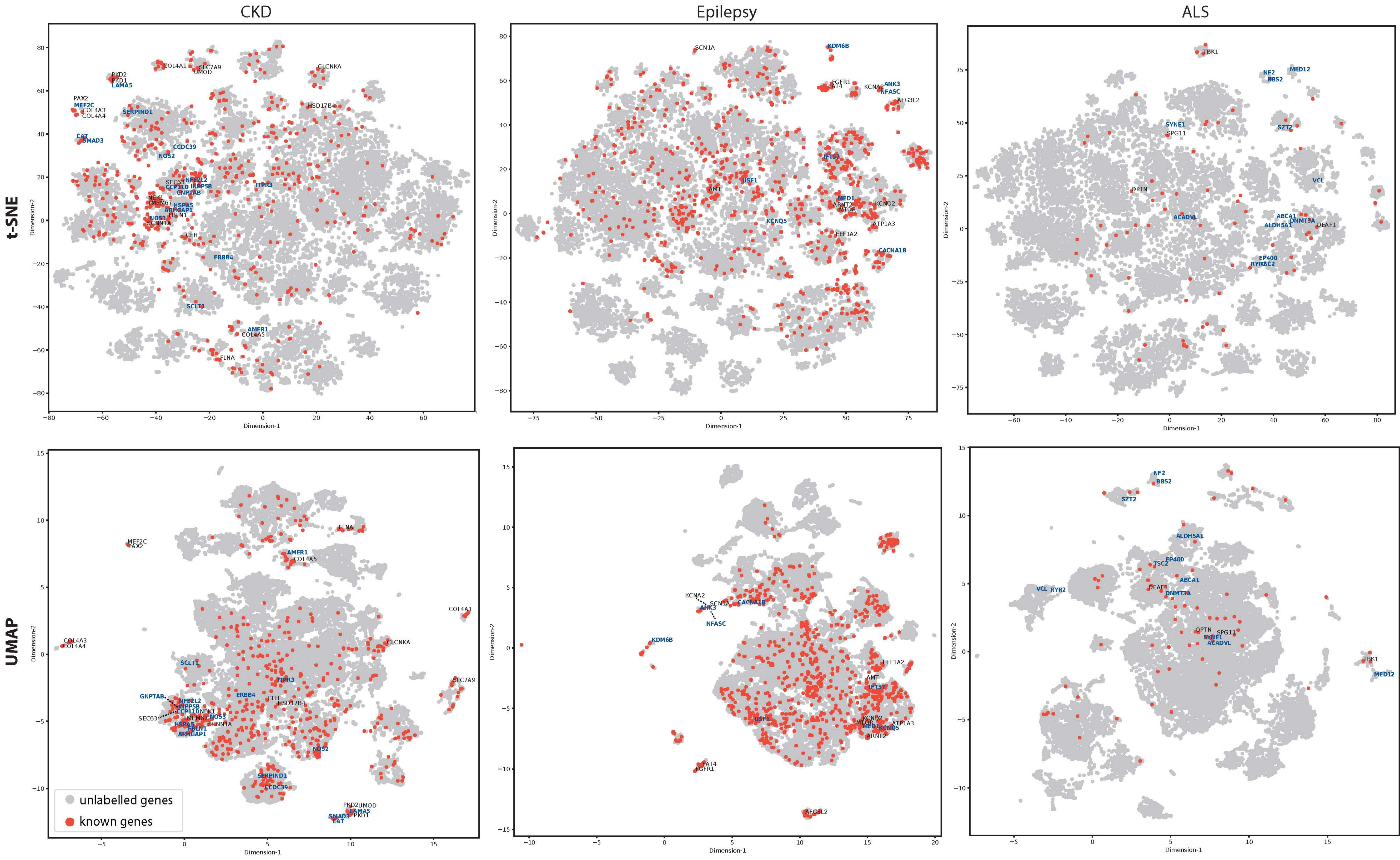

Figure S15. t-Distributed Stochastic Neighbor Embedding (t-SNE) and Uniform Manifold Approximation and Projection (UMAP) plots for 2D visualisation of all genes in each disease example. Labelled genes are all the consensus novel (dark blue) and known genes predicted by overlapping the mantis-ml predictions with rare-variant collapsing analysis results.

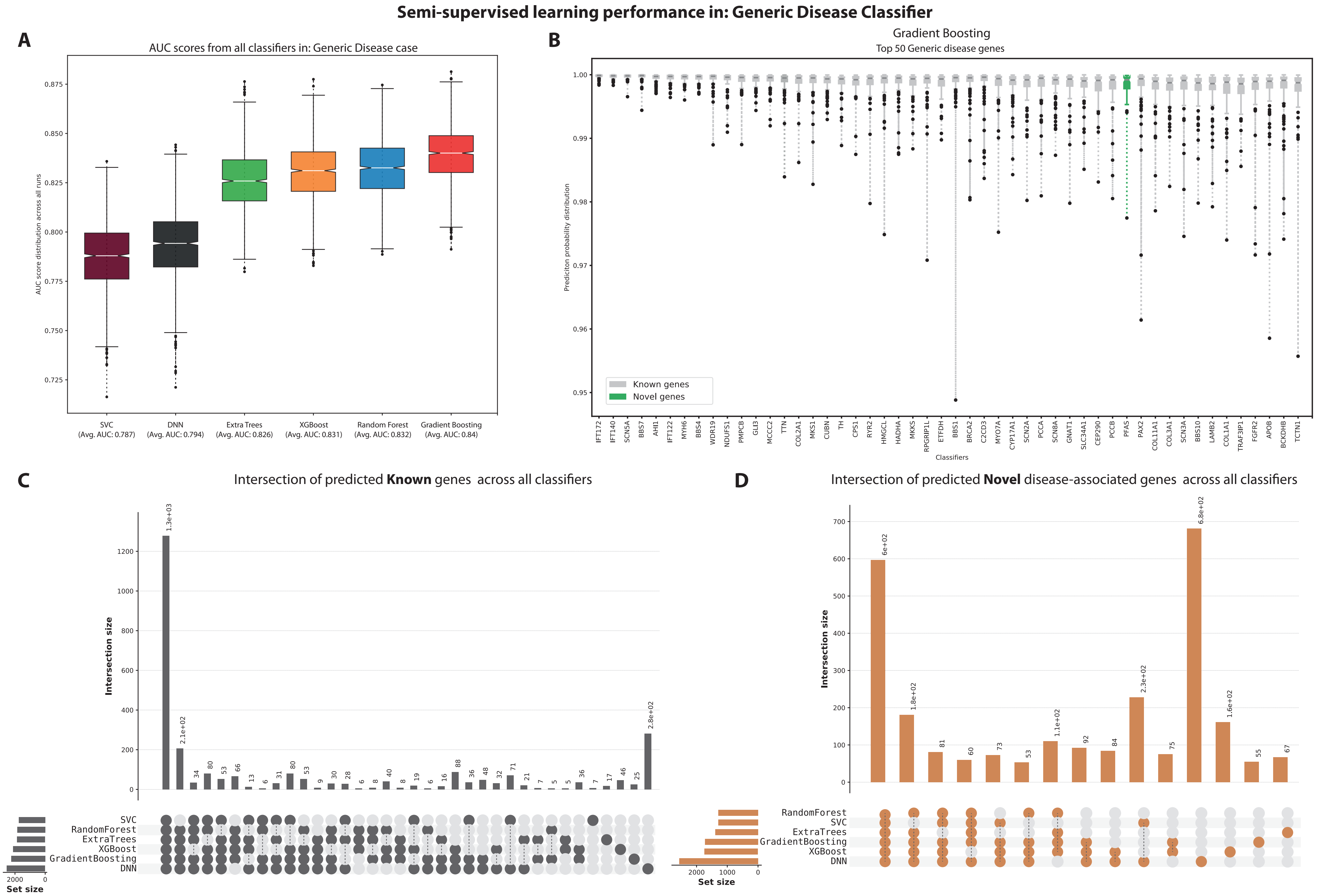

Figure S16. Mantis-ml performance on the Generic Disease case. A) AUC score distribution per standard classifier used during mantis-ml training. B) Prediction probabilities from the top 50 (known and novel) genes predicted with Gradient Boosting as the standard classifier. C/D) Intersection sets of predicted known/novel genes across all classifiers.

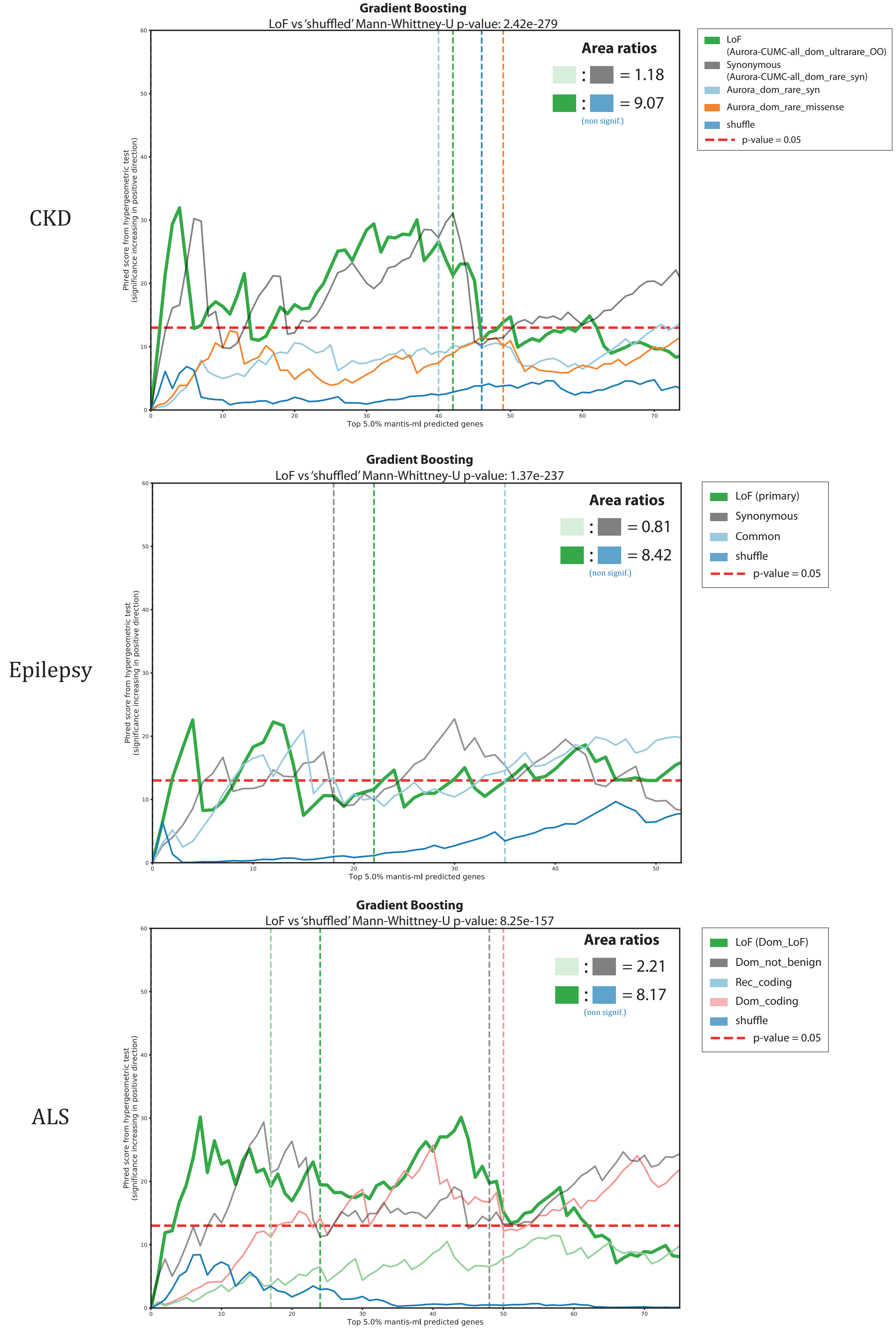

Figure S17. Enrichment (from hypergeometric test) of Generic mantis-ml predictions on disease specific collapsing analysis results from CKD, Epilepsy and ALS related studies.

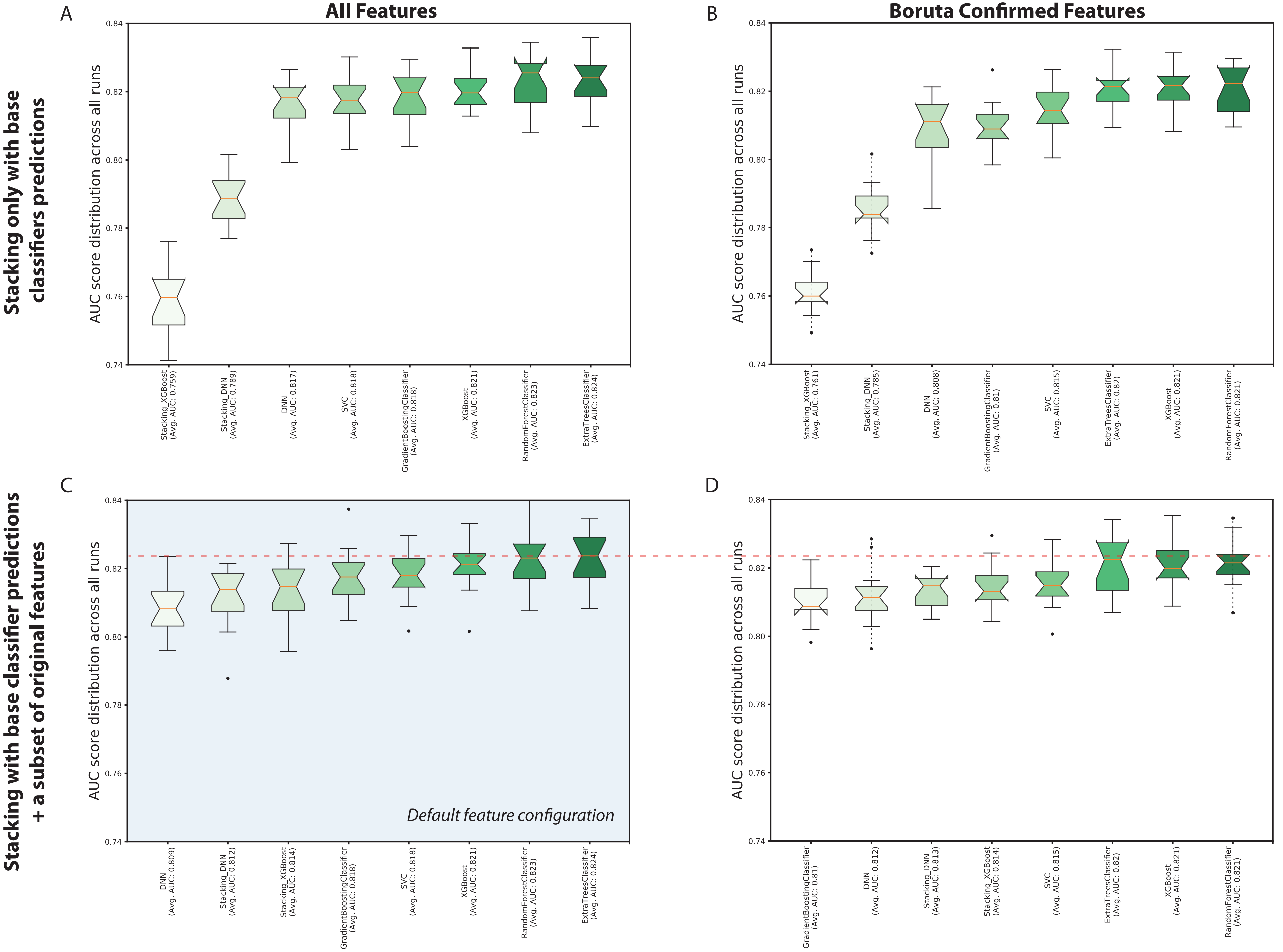

Figure S18. Benchmarking: AUC scores distribution from all classifiers in different feature selection configurations: a) using all features (after filtering) and training Stacking with base classifier predictions only, b) using only Boruta confirmed features and training Stacking with base classifier predictions + all Boruta confirmed features, c) using all features (after filtering) and training Stacking with base classifier predictions + all Boruta confirmed features and d) using only Boruta confirmed features and training Stacking with base classifier predictions + all Boruta confirmed features. Trainind data for benchmarking were compiled based on the Chronic Kidney Disease test case and included 15 random balanced datasets. Best average AUC performance was achieved by configuration (c) for all tested classifiers.

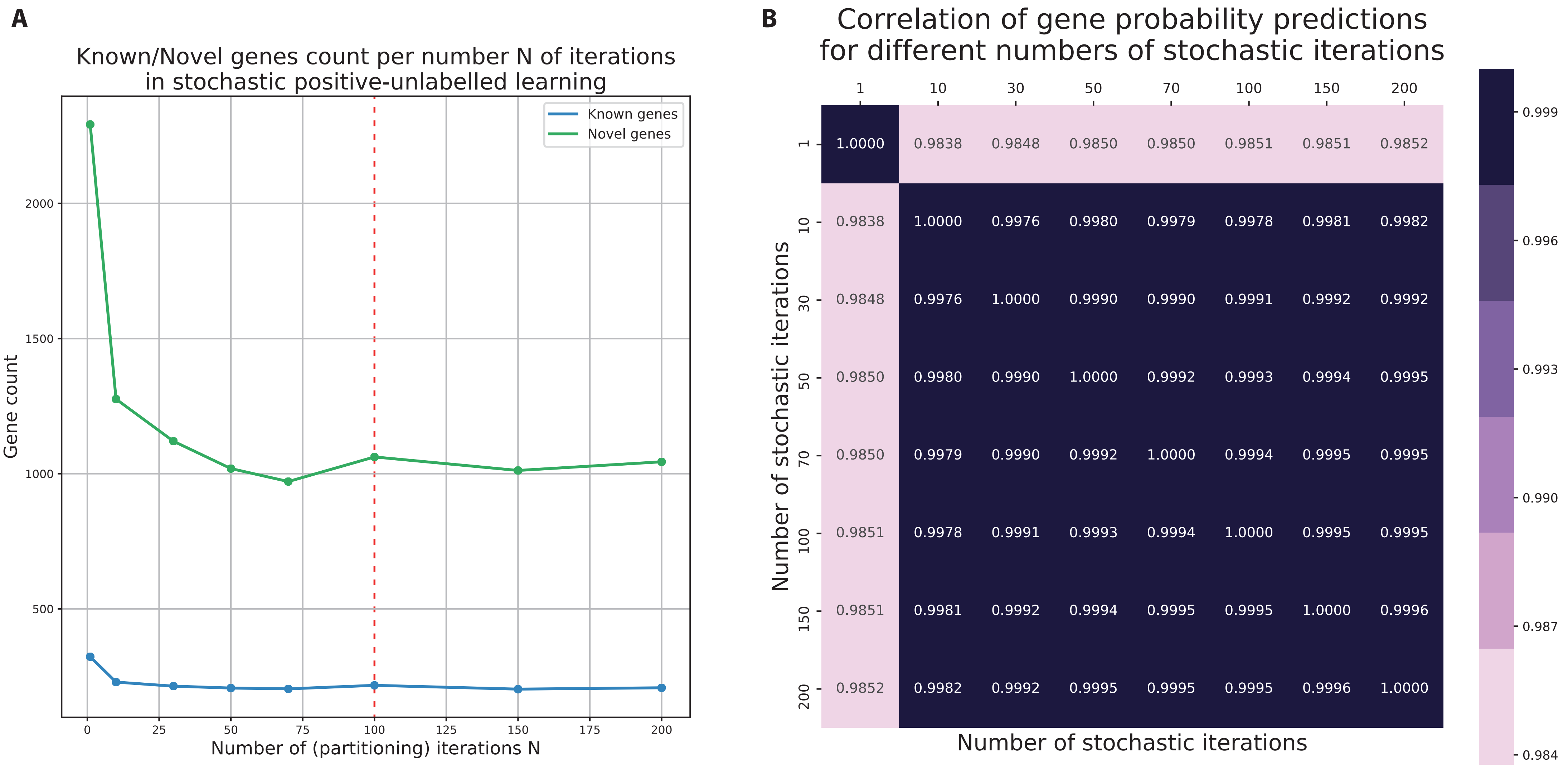

Figure S19. Benchmarking for: A) sensitivity check of the number of stochastic iterations to the predicted known and novel genes count and B) robustness of gene probability predictions for different numbers of iterations. (Tested numbers of iterations = [1, 10, 30, 50, 70, 100, 150, 200]).

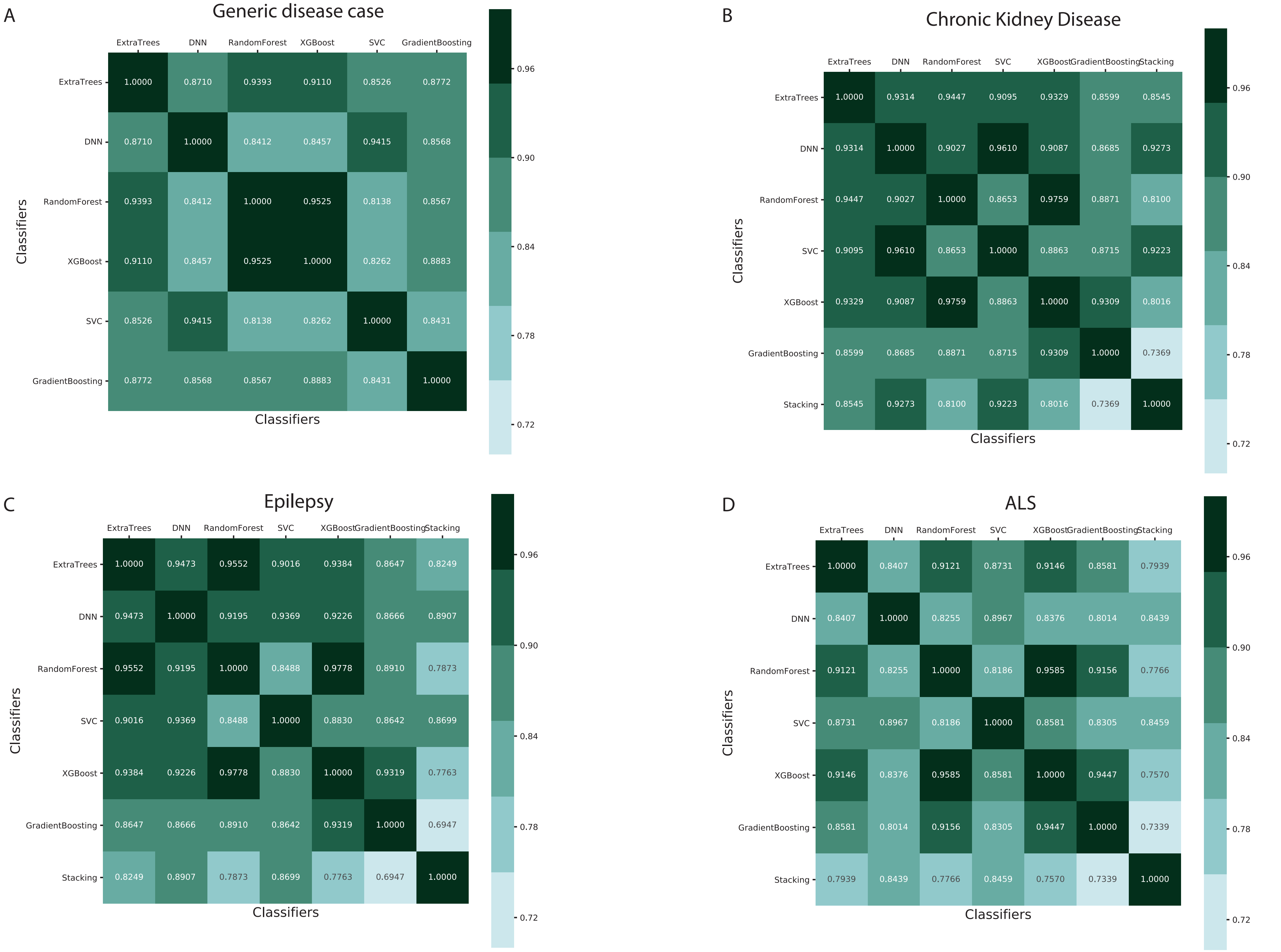

Figure S20. Correlation of gene probability predictions between different classifiers on the: A) Generic Disease, B) Chronic Kidney Disease, C) Epilepsy and D) ALS disease examples.

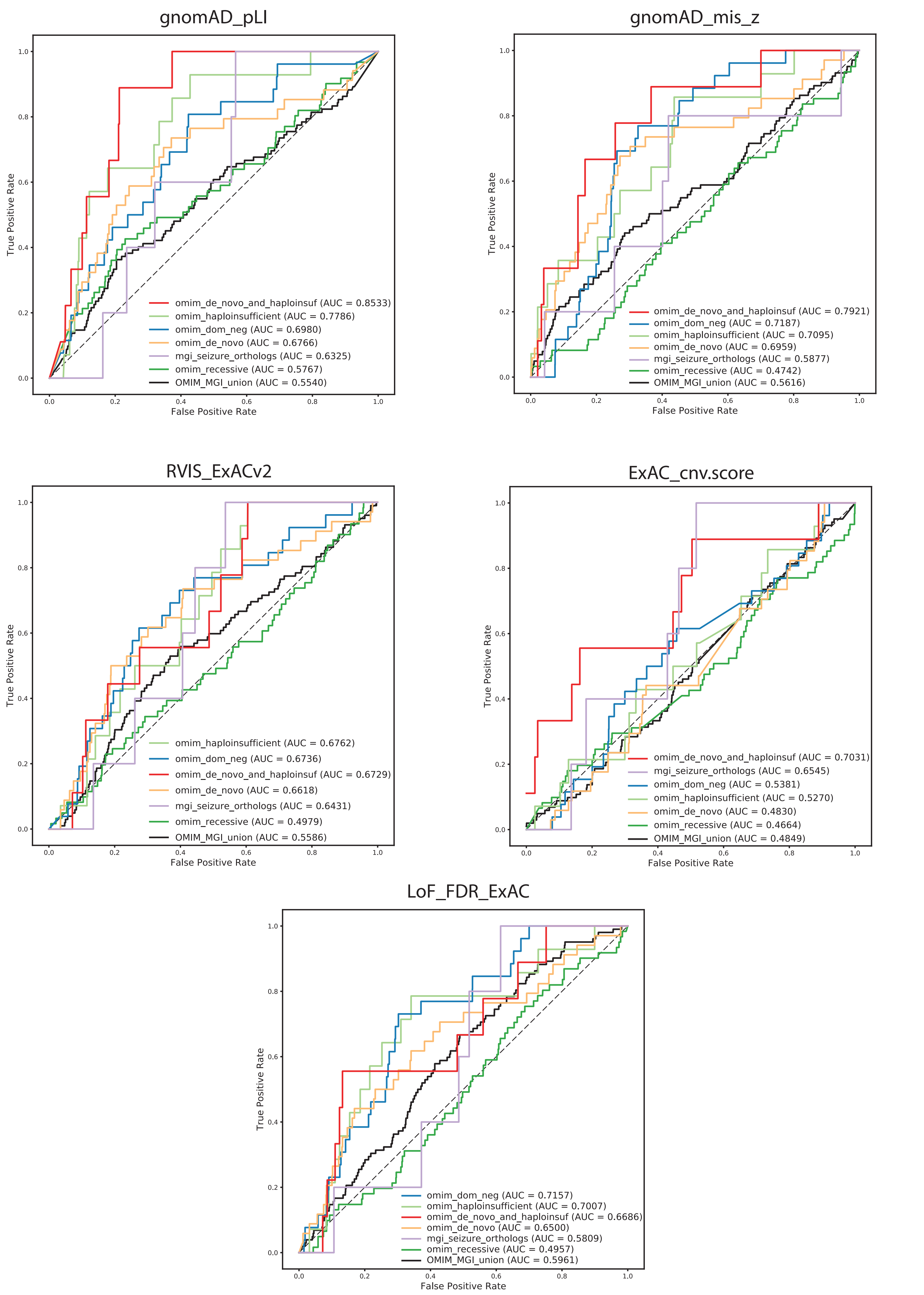

Figure S21. Predictive power of various intolerance scores for distinguishing different OMIM- and MGI-based genes from non-OMIM/MGI genes: gnomAD_pLI, gnomAD_mis_z, RVIS_ExACv2, ExAC_cnv.score and LoF_FDR_ExAC.

Cross-validation of mantis-ml predictions for "known" and "novel" genes against cohort-level rare-variant association studies

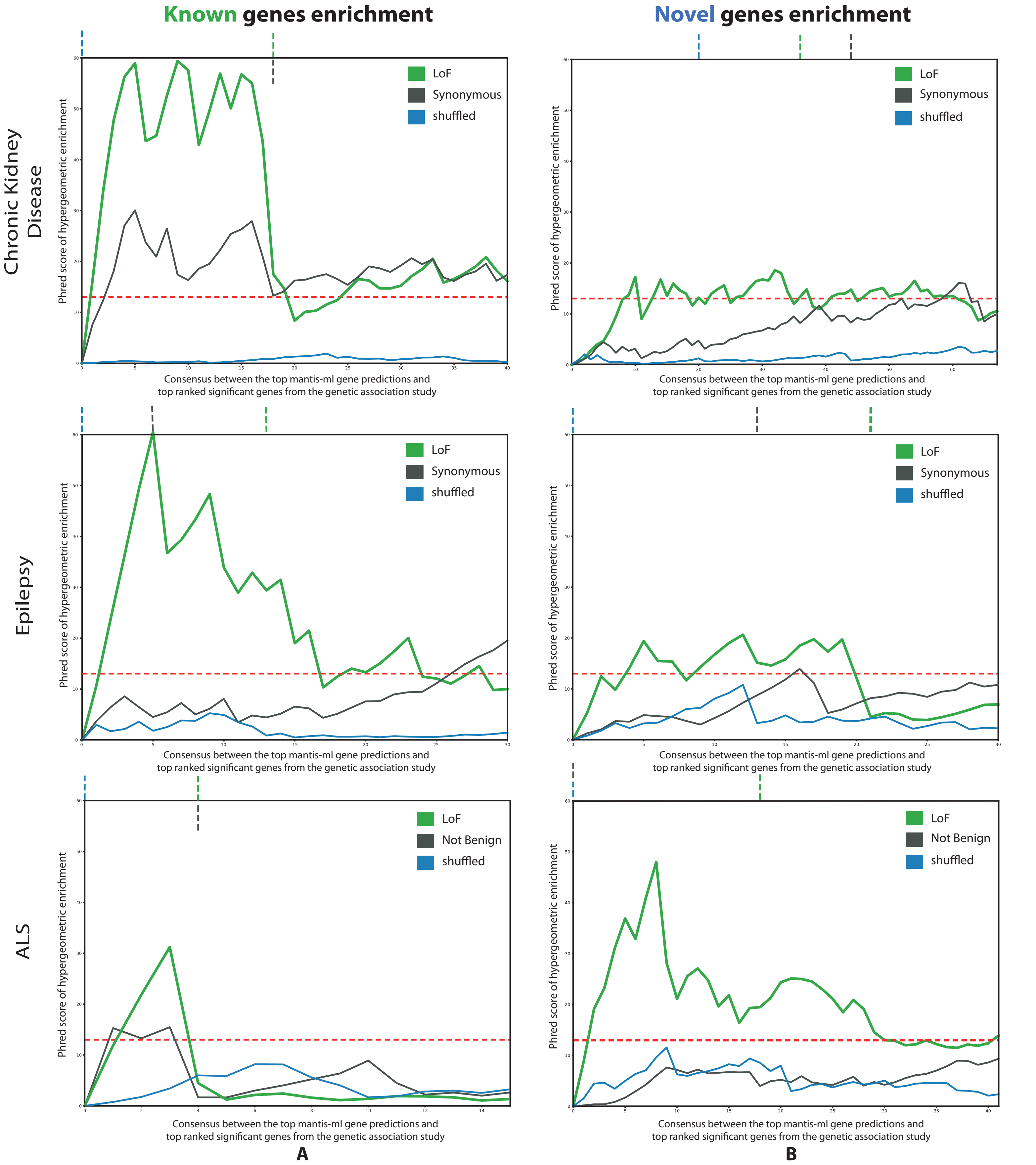

Figure S22. Hypergeometric test enrichment of disease-specific mantis-ml predictions for **A)** Known and **B)** Novel genes, against collapsing analysis results from CKD, Epilepsy and ALS cohorts and for different types of variants: Loss-of-Function (LoF), synonymous (if available) and shuffled. For the ALS disease example, a "Not Benign" class has been used (due to lack of a "synonymous variant"-based dataset), which represents a non-benign but less pathogenic than LoF set. The horizontal dashed red line corresponds to the significance threshold of p=0.05 for the hypergeometric tests. Where the plot(s) go above this line highlight significant enrichment of mantis-ml top gene predictions being enriched for among the population genomic collapsing analyses. The vertical dashed lines indicate the last index of top ranked genes from the collapsing analyses achieving a p-value < 0.05. For each disease example, the mantis-ml ranking used for performing the enrichment tests is the one extracted by the classifier with the highest average AUC performance (XGBoost for CKD and Epilepsy, ExtraTrees for ALS).

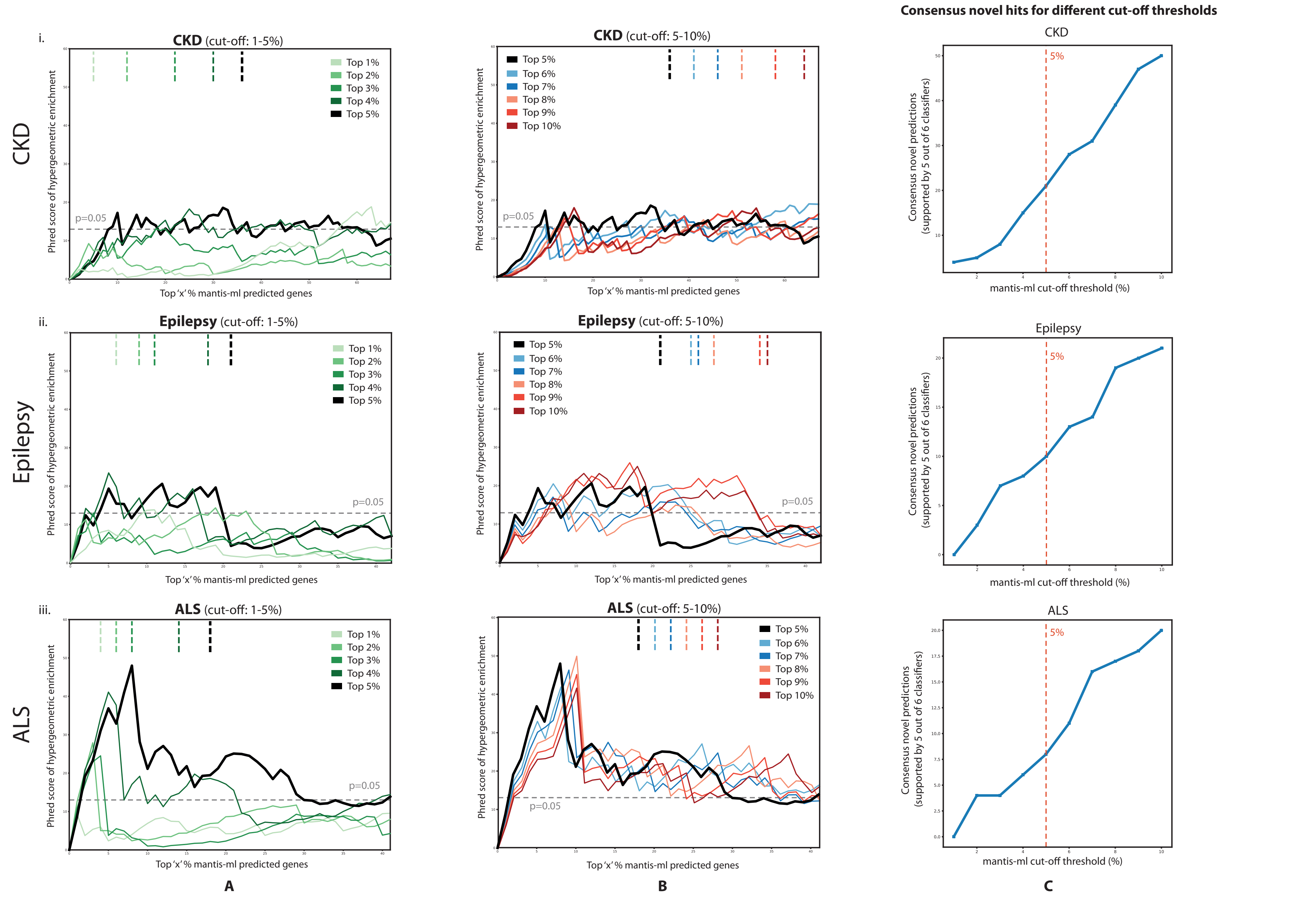

Figure S23. Sensitivity analysis for cut-off threshold of top mantis-ml "novel" gene predictions. **A & B)** Hypergeometric enrichment of collapsing analysis results (based on LoF variants) against different proportions of top mantis-ml predictions (1-10%) for three diseases: i) Chronic Kidney Disease, ii) Epilepsy and iii) Amyotrophic Lateral Sclerosis. **C)** Number of consensus novel gene predictions for different mantis-ml cut-off thresholds.

A

"Novel" genes (1-5% cutoff)
(Predicted by 5 out of 6 classifiers)

CKD

Top 1% : AMER1, LAMA5, NOS3, MEF2C (4 hits)

Top 2% : AMER1, LAMA5, NOS3, MEF2C, NOS2 (5 hits)

Top 3% : AMER1, LAMA5, NOS3, MEF2C, NOS2, GNPTAB, CAT, SMAD3 (8 hits)

Top 4% : AMER1, LAMA5, CCP110, NOS3, MEF2C, NOS2, GNPTAB, INPP5B, CAT, SMAD3, HSPA5, FBLN1, I TPR3, ARHGAP1, ERBB4 (15 hits)

Top 5% : AMER1, LAMA5, CCP110, NOS3, MEF2C, SCLT1,NOS2, IRS1, GNPTAB, INPP5B, CAT, CCDC39, SMAD3, HSPA5, FBLN1, ITPR3, MTTP, NFE2L2, ARHGAP1, ERBB4, M6PR (21 hits)

Epilepsy

Top 1% : None (0 hits)

Top 2% : USF1, IFT57, ANK3 (3 hits)

Top 3% : USF1, IFT57, MED1, KDM6B, ANK3, NFASC, KCNQ5 (7 hits)

Top 4% : USF1, IFT57, MED1, KDM6B, CUX1, ANK3, NFASC, KCNQ5 (8 hits)

Top 5% : CACNA1B, USF1, IFT57, MED1, DMXL2, KDM6B, CUX1, ANK3, NFASC, KCNQ5 (10 hits)

ALS

Top 1% : None (0 hits)

Top 2% : MED12, SZT2, ACADVL, TSC2 (4 hits)

Top 3% : MED12, SZT2, ACADVL, TSC2 (4 hits)

Top 4% : MED12, SZT2, ACADVL, TSC2, OPA1, ABCA1 (6 hits)

Top 5% : MED12, SZT2, ACADVL, NF2, TSC2, OPA1, ABCA1, DNMT3A (8 hits)

B

"Novel" genes (6-10% cutoff)
(Predicted by 5 out of 6 classifiers)

CKD

Top 6% : AMER2, LAMA5, CCP110, NOS3, MEF2C, SCLT1, NOS2, IRS1, SERPIND1, GNPTAB, HSPA4L, INPP5B, CAT, CCDC39, SMAD3, LRRFIP1, HSPA5, FBLN1, ITPR3, MTTP, CYP4F3, NFE2L2, ARHGAP1, ERBB4, GNE, ASH1L, M6PR, CTNNA1 (28 hits)

Top 7% : AMER1, LAMA5, CCP110, NOS3, MEF2C, SCLT1, NOS2, IRS1, SERPIND1, GNPTAB, HSPA4L, INPP5B, CAT, CCDC39, SMAD3, LRRFIP1, HSPA5, FBLN1, GCGR, ITPR3, MTTP, CYP4F3, NFE2L2, ARHGAP1, ERBB4, GNE, LIPA, ASH1L, M6PR, EFEMP1, CTNNA1 (31 hits)

Top 8% : AMER1, LAMA5, CCP110, NOS3, MEF2C, SCLT1, NOS2, IRS1, SERPIND1, GNPTAB, HSPA4L, INPP5B, CAT, CCDC39, SMAD3, LRRFIP1, HSPA5, FBLN1, GCGR, ITPR3, MTTP, CYP4F3, NFE2L2, ARHGAP1, ERBB4, GNE, LIPA, ASH1L, M6PR, PANK1, HOXD4, PAM, ITSN2, EFEMP1, PDE2A, CDH6, CDH2, SZT2, CTNNA1 (39 hits)

Top 9% : AMER1, LAMA5, CCP110, NOS3, MEF2C, SCLT1, NOS2, IRS1, DSG2, SERPIND1, GNPTAB, HSPA4L, INPP5B, CAT, CCDC39, ROBO3, SMAD3, LRRFIP1, HSPA5, FBLN1, GCGR, C5, ITPR3, MTTP, CYP4F3, SVIL, NFE2L2, ARHGAP1, ERBB4, GNE, LIPA, ASH1L, M6PR, PANK1, SLC12A7, HOXD4, PAM, ITSN2, EFEMP1, PLCB3, EIF2AK4, PDE2A, CDH6, CDH2, SZT2, TXNRD2, CTNNA1 (47 hits)

Top 10% : AMER1, LAMA5, CCP110, NOS3, MEF2C, SCLT1, NOS2, IRS1, DSG2, SERPIND1, GNPTAB, HSPA4L, INPP5B, CAT, CCDC39, ROBO3, SMAD3, LRRFIP1, HSPA5, FBLN1, GCGR, C5, ITPR3, MTTP, CYP4F3, SVIL, NFE2L2, ARHGAP1, ERBB4, GNE, LIPA, ASH1L, M6PR, PANK1, SLC12A7, HOXD4, PAM, ITSN2, EFEMP1, MXI1, PLCB3, EIF2AK4, RTCA, PDE2A, CDH6, CDH2, SZT2, TXNRD2, PKN1, CTNNA1 (50 hits)

Epilepsy

Top 6% : CACNA1B, USF1, IFT57, MED1, DMXL2, KDM6B, CUX1, ANK3, NFASC, KCNQ5, RGS4, STK3, GAK (13 hits)

Top 7% : CACNA1B, USF1, IFT57, MED1, DMXL2, KDM6B, CUX1, ANK3, NFASC, KCNQ5, RGS4, STK3, ATXN1, GAK (14 hits)

Top 8% : CACNA1B, USF1, IFT57, VPS41, MED1, DMXL2, KDM6B, CUX1, ANK3, NFASC, KCNQ5, RGS4, ALKBH1, STK3, ATXN1, GAK, EFNB1, MYO10, TYRO3 (19 hits)

Top 9% : CACNA1B, USF1, IFT57, VPS41, MED1, DMXL2, KDM6B, CUX1, ANK3, NFASC, KCNQ5, RGS4, ALKBH1, STK3, ATXN1, GAK, EFNB1, PRKCG, MYO10, TYRO3 (20 hits)

Top 10% : CACNA1B, USF1, IFT57, VPS41, MED1, DMXL2, KDM6B, CUX1, ANK3, NFASC, KCNQ5, RGS4, ALKBH1, STK3, ATXN1, GAK, EFNB1, WDR83, PRKCG, MYO10, TYRO3 (21 hits)

ALS

Top 6% : MED12, SZT2, ACADVL, NF2, TSC2, VCL, OPA1, ALDH5A1, SYNE1, ABCA1, DNMT3A (11 hits)

Top 7% : MED12, SZT2, ACADVL, EP400, NF2, TSC2, VCL, OPA1, ADCYAP1R1, ALDH5A1, SYNE1, ABCA1, NIN, MYOF, DNMT3A, MDN1 (16 hits)

Top 8% : MED12, SZT2, ACADVL, EP400, NF2, TSC2, POLD1, VCL, OPA1, ADCYAP1R1, ALDH5A1, SYNE1, ABCA1, NIN, MYOF, DNMT3A, MDN1 (17 hits)

Top 9% : MED12, SZT2, ACADVL, EP400, NF2, TSC2, POLD1, VCL, OPA1, ADCYAP1R1, ALDH5A1, SYNE1, ABCA1, NIN, MYOF, DNMT3A, BBS2, MDN1 (18 hits)

Top 10% : MED12, SZT2, ACADVL, EP400, NF2, TSC2, APC, POLD1, VCL, OPA1, RYR2, ADCYAP1R1, ALDH5A1, SYNE1, ABCA1, NIN, MYOF, DNMT3A, BBS2, MDN1 (20 hits)

Figure S24. Sensitivity analysis for different cut-off thresholds of top mantis-ml "novel" gene predictions. Consensus of "novel" gene predictions, supported by 5 out of 6 classifiers used by mantis-ml, across the three disease examples, for different % ratios of top mantis-ml predictions.: **A)** 1-5%. **B)** 6-10%.

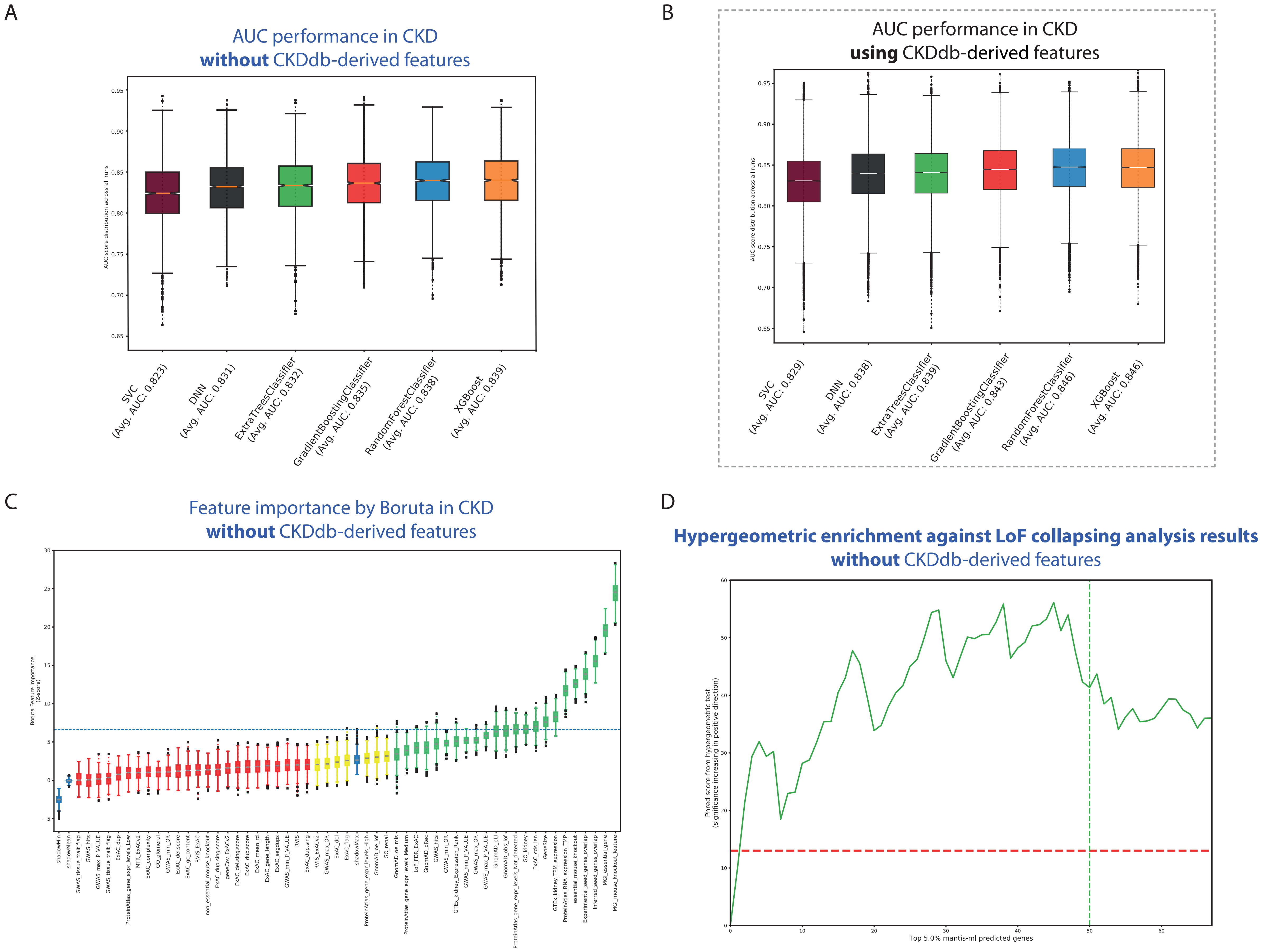

Figure S25. mantis-ml performance on CKD with/without CKDdb-derived features. **A.** AUC performance per classifier **without** using CKDdb-derived features. **B.** AUC performance per classifier **using** CKDdb-derived features. **C.** Boruta-based feature importance **without** using CKDdb-derived features. **D)** Hypergeometric enrichment of top 5% mantis-ml predictions against top collapsing analysis hits (based on LoF variants) **without** using CKDdb-derived features.

SUPPLEMENTAL METHODS

Annotation of disease/phenotype-associated genes

We are using the Human Phenotype Ontology (HPO) as our default resource to annotate disease/phenotype-associated genes. HPO contains over 13,000 terms and over 156,000 annotations to hereditary diseases. It is a comprehensive resource that leverages information from the collection of OMIM, DECIPHER, Orphanet and the medical literature. HPO originally started with a focus on Mendelian diseases but has gradually expanded onto other more common complex disease areas, such as cardiology and immunology, through regular workshops with clinicians. The use of HPO as our default annotation resource is consistent with other methods that we benchmarked to, such as Phenolyzer, which is also based on it.

Annotation of known disease-associated genes is performed by automatically selecting genes whose 'HPO-Term-Name' contains any of the 'Disease/Phenotype terms' and does not contain any of the 'Diseases/Phenotypes to exclude' from the config.yaml configuration file. Alternatively, the mantis-ml framework permits the use of user-specified positive label seed genes in place of the HPO mapping. This is accessible via the "*-k*" option with the *mantisml* command.

Distinct machine learning architecture of the mantis-ml framework

mantis-ml implements a novel design of a stochastic semi-supervised learning framework. To our knowledge, there is only one other instance of a "stochastic semi-supervised learning" approach[1]. That approach is fundamentally different to mantis-ml with respect to the target of the problem, solution architecture and end results.

Specifically, Xie et al.'s approach always begins with a single positive data point and augments either with a single negative data point (k-means clustering method) or a random subset of 20 unlabelled data points (logistic regression method). In each iteration, more unlabelled data points are added and each of them assigned to the positive or negative class (through clustering or supervised learning, respectively). Eventually, each data point is assigned a class based on majority voting across all iterations. The top/bottom 1% are then labelled as "positive" / "negative" and a new model is trained to be ready for prediction on new unseen data. This is a method applied to datasets with an extremely rare set of positive labels. However, results can be easily prone to extreme overfitting initiated by the starting seed data point and then gradually mitigating through the rest of data points, as they are labelled "positive" or "negative" to inform the next stages of learning.

The architecture of our method is fundamentally different. Firstly, it's applied into a finite set of data points (all known human genes). Given this fixed annotation of human genes, no new unlabelled data can be added to the dataset. Furthermore, mantis-ml starts with a set of M positive data points (genes annotated from HPO, and usually M>>1) and treats the rest (N) as unlabelled. The goal is to rank all M+N data points based on the information of all (M) positive labelled points. Instead of providing a label of "positive" or "negative" in the end, mantis-ml aims to provide a propensity score (prediction probability score) for each gene which indicates the likelihood of association of each gene with the respective disease. The only similarity here is that the final propensity score is the average from multiple iterations which contain a random process. In Xie et al.'s, the randomness comes only from the sampling of unlabelled data points, as they are added in batches of 1 or 20 points, while in mantis-

ml it is derived by the random partitioning of the entire gene space (for L stochastic iterations) and then random split and learning over k-folds in each balanced dataset.

One of the most substantial differences is that our method uses the entire set of “positive”-labelled genes for supervised learning, which enables a holistic assessment of the relationships between all features across a wider set of seed data points. At the same time, in each random balanced dataset (positive/unlabelled ratio = 2:3), we use 80% of our original “positive”-labelled points to reduce overfitting on a single ground truth set thus adding an extra parameter of regularisation. Finally, the end goal of mantis-ml is not to produce a model that can be applied to new unseen data, but rather self-rank an originally highly imbalanced dataset with a finite number of points.

Finally, we employ well established supervised methods as part of the mantis-ml framework (implemented in scikit-learn and tensorflow/keras) which are used during training on the balanced datasets. Mantis-ml does not represent a novel general-purpose supervised learning algorithm but is instead a comprehensive framework for semi-supervised learning on highly imbalanced datasets in a finite space of data points.

Concordance of results between different classifiers

We employed a nominal decision probability threshold of 0.5 for assessment of the concordance of results across classifiers, as shown in Suppl. Figures 6,7 and 8 (c-d). This threshold is not used for decision making. We instead retain raw prediction probabilities for each gene to then focus on top hits of the final ranking rather than adopt a threshold segregation approach.

We first assessed the concordance between classifiers on the Chronic Kidney Disease example. 280 known genes (47.7% of all known genes) have been identified by all classifiers and another 64 genes (11% of all known genes) by at least five classifiers. In terms of the novel disease-associated genes, again the largest group of predicted genes-of-interest (n=1,300) has been predicted by all classifiers. However, each classifier calculates their predictions with a ranking score (prediction probability) which immediately provides a prioritisation scheme for the extracted gene predictions. Having no further knowledge to validate mantis-ml predictions at this stage, we choose to consider the gene rankings of the classifier with the highest average and individual AUC scores (XGBoost) in that case as the default mantis-ml prioritisation scheme for CKD (Suppl. File 2; Table S1). We found that the mantis-ml gene prediction rankings were significantly correlated when comparing XGBoost and Random Forests (Pearson’s r = 0.976; p < 2.2 x 10-308, Suppl. Fig. 20), further demonstrating the robustness of the predictions beyond the choice of classifier.

Moving on to the mantis-ml performance on the Epilepsy example, we observe again all tree-based classifiers (XGBoost, Random Forest, Gradient Boosting and Extra Trees) performing best, with respective average AUC scores in descending order: 0.821, 0.818, 0.816 and 0.808 (Suppl. Fig. 7). We observe the preponderance of 360 known genes (41.7% of all known genes) being predicted by all seven classifiers followed by another 67 genes (7.7% of all known genes) predicted by at least six classifiers (based on prediction probability threshold of 0.5). Additionally, around 1,600 novel genes have been suggested by all classifiers. We provide as the default gene ranking for Epilepsy the mantis-ml predictions acquired when using XGBoost as the standard classifier during positive-unlabelled learning, based on its best AUC performance among all classifiers (Suppl. File 2; Table S2).

With regards to mantis-ml predictions on ALS (Suppl. Fig. 8), Extra Trees followed by XGBoost, SVC and Random Forest showed the best performance (average AUC scores: 0.814, 0.805, 0.801 and 0.798). Moreover, 31 known (40.1% of all known genes) and 1,500 novel genes were predicted by all classifiers (based on prediction probability threshold of 0.5). We also observe in this disease example that beginning with a smaller number of seed genes (n=77) is accompanied by a drop in average AUC scores. This suggests that mantis-ml performance has greater confidence from an increased presence of known genes. We provide the Extra Trees ranking scores as the default mantis-ml predictions for ALS (Suppl. File 2; Table S3).

Relative performance in different disease contexts

We found that the relative performance of the different supervised-learning models depended on the disease context as demonstrated in the results per disease at the mantis-ml gene prioritisation atlas (https://dvitsios.github.io/mantis-ml-predictions). However, in general the classifiers themselves performed relatively comparably.

For instance, SVC has performed better than Random Forest and most of the other tree-based methods in several occasions such as ALS, Alzheimer's, Autism and Pulmonary disease. Additionally, DNN performs better than ExtraTrees or other tree-based methods in Cardiovascular Disease, Pulmonary Disease and Respiratory Disease. In other cases, tree-based models perform relatively compared to DNN and SVC.

In general, we believe that the relative performance of classifiers largely depends on the disease under study each time, as it is captured by the number of known seed genes, any sparsity of relevant features (in which case SVC would probably perform better) or other parameters such as the genetic context of the disease and how well or confidently it is captured by the disease-specific set of features available in the databases we are using. At the same time, the difference in performance between classifiers is in most cases relatively small as it falls within the range of a 0.05-0.1 change in AUC.

Description of the Boruta algorithm

Boruta is a wrapper algorithm that can be used with any model that can calculate feature importance scores. It starts with creating random permutations of the original features (shadow features) which are then used along with the original ones by the learning model. In an iterative manner, the original features' contribution is compared against those from shadow features (Z-score difference) and only original features that exceed the max. importance of the best performing shadow feature are labelled as 'Confirmed' and discarded from future iterations. The ones that are below the min. importance of the worst performing shadow feature are 'Rejected'. The rest continue being tested until being 'Confirmed' or 'Rejected' or until the max. number of iterations has been reached. With regards to extracting the attribute importance, we calculate Z-scores of mean decrease accuracy, however using Gini impurity is also an option available in the Boruta algorithm and its R package implementation. Specifically, this can be achieved by setting *getImp="getImpExtraGini"* in the *Boruta()* S3 method instead of using the default *getImp="getImpExtraZ"* option.

Refined prioritisation using Stacking classifier

DNN is often adopted for extracting features of increasing complexity as one goes deeper into the network and the extracted "engineered"-features could then be fed into another model, e.g. XGBoost to perform the prediction task. This has primarily been a successful approach in scenarios where the input feature set is "unstructured", e.g. with imaging, text or sound data. In our learning task, the original feature set is well curated and "engineered" based on domain-specific knowledge aggregated in life sciences. Thus, the stacking classifier (enabled via "-s" option in *mantisml*) in our case serves not as a higher-level feature extractor but rather as an attempt to smooth out predictions from multiple classifiers and provide more filtered and refined results that have been validated by multiple models.

Feature set reduction and considerations for noise elimination

mantis-ml integrates over 1,200 features in total. For instance, many features refer to Gene Ontology annotations across various terms or some other features may reflect gene expression across different tissues. Only disease/phenotype-relevant terms or tissues are eventually selected for a disease of interest in an automatic manner, based on the disease/phenotype terms provided by the user in free text form. This means that eventually only 50-80 features are used during training for a particular disease/phenotype, dramatically reducing the risk of inclusion of noisy features from the original set of 1,200 features. We have also tried eliminating the noise from irrelevant features even further, by training each model using only the Boruta confirmed features. Performance in that case drops slightly across all classifiers, compared to when using all features (Suppl. Fig. 18). Thus, the feature set used during training (without feature-selection by Boruta) appears to be a non-redundant representation of the original features.

Sensitivity analysis for balancing ratio between positive and unlabelled data points

We explored the impact of selecting different balancing ratios between positive (P) and unlabelled (U) data points when forming random balanced datasets. We ran the Chronic Kidney Disease example for i=2 stochastic iterations using three different P/U balancing ratios: 1, 1.5 and 2, and calculated the correlations of gene probability predictions from the same classifier between each pair of runs (**Table S1**). We observe that correlations between balancing ratios 1 and 1.5 are in the range of 0.934-0.985 while for balancing ratios 1 and 2 are in the range of 0.874-0.974 (runs with balancing ratios 1.5 and 2 are even more similar with Pearson's r = 0.959-0.989). Since we want to create (as much as possible) balanced datasets and predictions with balancing ratio 1.5 are highly correlated with those with ratio=1 and even more than when using ratio=2, we choose 1.5 as the default value for balancing ratio.

The positive-unlabelled ratio is also provided as an advanced parameter which could be adjusted by an expert user based on needs. This is accessible at: "mantis-ml-release/mantis_ml/conf/.conf" as "balancing_ratio" parameter in the "supervised_filters".

Table S1. Pearson's r correlations between gene probability predictions extracted by each classifier for different values of positive-unlabelled balancing ratios (p-value < 2.2×10^{-308} for all comparisons).

Classifier	Compared balancing ratios: 1 vs 1.5	Compared balancing ratios: 1.5 vs 2	Compared balancing ratios: 1 vs 2
XGBoost	0.985	0.989	0.974
Random Forest	0.983	0.989	0.972

Gradient Boosting	0.934	0.959	0.874
Extra Trees	0.987	0.991	0.978
DNN	0.96	0.972	0.943
SVC	0.971	0.977	0.925

Sensitivity analysis for top mantis-ml predictions cut-off threshold during validation with independent studies

When performing an enrichment test of the mantis-ml predictions against independent gene rankings from external studies (e.g. WES rare-variant association studies), we define a certain cut-off for the top mantis-ml predictions to use. We have explored 10 different thresholds, from 1% to 10% (in increments of 1). We observed that the enrichment signal is consistently significant across all three disease examples (CKD, Epilepsy and ALS) when selecting a cut-off threshold ≥ 5% (**Figure S23 A and B**). We also observed that for cut-off thresholds > 5%, the hypergeometric enrichment saturates, however allowing for the extraction of larger numbers of novel genes. In general, selecting a certain cut-off threshold allows for the extraction of different numbers of prioritised genes eventually (**Figure S23 C & Figure S24**). For instance, in CKD, the 1-10% cut-off thresholds result to 4, 5, 8, 15, 21, 28, 31, 39, 47 and 50 gene hits, respectively (**Figure S24**), that are supported both by mantis-ml and the collapsing analysis. It is possible for the user to define more stringent or lenient cut-off thresholds when aiming to extract a more compact or expanded list of suggested prioritised genes to facilitate any further follow-up validations (the default value is set to 5%).

Interactive visualisation of novel gene predictions

We provide as part of the mantis-ml results, interactive versions of the PCA, t-SNE and UMAP plots (enabled via the "bokeh" library) in the form of ".html" files under the "[output_dir]/Output-Figures/unsupervised-learning" directory. These plots highlight all seed genes in red and the top 40 novel predictions (extracted by the classifier with the highest average AUC performance) in black. The user can zoom in any sub-region of each of these plots and inspect the names of predicted novel genes as well as any neighbouring known genes.

External module requirements and versions

Python3 (tested with v3.6.7)

- numpy: 1.14.5
- numpydoc: 0.8.0
- pandas: 0.24.2
- scipy: 1.2.1
- scikit-learn: 0.20.3
- bokeh: 1.1.0
- h5py: 2.9.0
- tensorflow: 1.10.0
- Keras: 2.2.4
- matplotlib: 3.0.3

- palettable: 3.1.1
- plotly: 3.9.0
- PyYAML: 5.1
- seaborn: 0.9.0
- tables: 3.5.1
- twine: 3.0.0
- tqdm: 4.14
- umap-learn: 0.3.8
- xgboost: 0.80

R (tested with v3.5.1)

- Boruta package (v6.0.0)

Data availability & pre-processing

- Generic Resources

ExAC

Exome Aggregation Consortium (ExAC) data are available at: http://exac.broadinstitute.org/downloads (last accessed on 06/03/2019). We integrate all data from CNV Counts and Intolerance Scores (‘*exac-final-cnv.gene.scores071316*’) and the ‘*GeneSize*’ feature from the Functional Gene Constraint Scores (‘*fordist_cleaned_exac_r03_march16_z_pli_ rec_null_data.txt*’).

Essential mouse genes

We integrate data from Georgi et al. (2013) that contain annotation for human orthologs of mouse genes that have been found to be essential for basic developmental functions and/or survival in both species (available at: https://doi.org/10.1371/journal.pgen.1003484.s022, last accessed on 06/03/2019). Both genes that have been identified as essential or non-essential are recorded and used as features by *mantis-ml*.

Genic-intolerance scores

We integrate two types of genic-intolerance scores: Residual Variation Intolerance Score (RVIS) and Missense Tolerance Score (MTR). RVIS scores (applied to EVS, ExAC and ExAC v2) are publicly available at http://genic-intolerance.org while MTR scores are publicly available at http://mtr-viewer.mdhs.unimelb.edu.au (both last accessed on 06/03/2019).

GnomAD

Genome Aggregation Database (GnomAD) data are publicly available at https://gnomad.broadinstitute.org/downloads (release 2.1, last accessed on 06/03/2019). We integrate all gene constraint scores in the *mantis-ml* framework. We retain for each gene all associated constraint scores that correspond to the canonical transcript, choosing the longest one in case there are more than one canonical transcript annotated for a gene. Aforementioned ExAC is a subset of GnomAD; however, both versions of the associated constraint scores are adopted.

GWAS (used both in the generic and disease-specific models)

Genome Wide Association (GWAS) data are publicly available at: https://www.ebi.ac.uk/gwas/docs/file-downloads (last accessed on 06/03/2019). We integrate data from ‘All associations’ (v1.0.2). For the disease-specific model we select all entries that contain any

of the '*Disease/Phenotype terms*' and '*Additional associated terms*' and do not contain any of the '*Diseases/Phenotypes to exclude*' from *config.yaml*. For the generic model we include all entries. In both cases, however, we filter out any entry with a p-value over the genome-wide significance threshold (p-value threshold: $5x10^{-8}$). Then, both for the disease-specific and generic model, we assign a True boolean flag to every gene that has at least one GWAS hit for any of the query terms specified. We also record the total number of GWAS hits per gene as well as the min/max p-values and min/max Odds Ratios associated with each gene.

MGI (generic)
Mouse Genome Informatics (MGI) data are publicly available at: http://www.informatics.jax.org/downloads/reports/index.html (last accessed on 06/03/2019). We are integrating data from three files: Genotypes and Mammalian Phenotype Annotations for Marker Type Genes excluding conditional mutations ('*MGI_GenePheno.rpt*'), Mouse/Human Orthology with Phenotype Annotations ('*HMD_HumanPhenotype.rpt*') and Mammalian Phenotype Vocabulary in OBO v1.2, tab-delimited and OWL Formats ('*VOC_MammalianPhenotype.rpt*'). We combine all data from these files to link human with mouse orthologs and their associated high-level mammalian phenotype descriptions and IDs. Gene labelling for this feature is performed by string matching of the '*Disease/Phenotype terms*' and '*Additional associated terms*' from the given *config.yaml* file with the 'High-level Mammalian Phenotype ID' field in *hmd_human_pheno.processed.rpt*. Finally, we also annotate all genes that are associated with a 'Lethal' phenotype with a True boolean flag. The MP IDs associated with a 'Lethal' phenotype are: 0002058, 0002080, 0002081, 0002082, 0002083, 0006204, 0006205, 0006206, 0006207, 0006208, 0008527, 0008569, 0008762, 0009850, 0010768, 0010769, 0010770, 0010831, 0010832, 0011083, 0011084, 0011085, 0011086, 0011087, 0011088, 0011089, 0011090, 0011091, 0011092, 0011093, 0011094, 0011095, 0011096, 0011097, 0011098, 0011099, 0011100, 0011101, 0011102, 0011103, 0011104, 0011105, 0011106, 0011107, 0011108, 0011109, 0011110, 0011111, 0011112, 0011400, 0013292, 0013293, 0013294.

- Resources filtered by tissue/disease

GTEx
Genotype-Tissue Expression (GTEx) data are publicly available at: https://gtexportal.org/home/datasets (V7, last accessed on 06/03/2019). We integrate RNA-Seq data that contain the median TPM expression values by tissue ('*GTEx_Analysis_2016-01-15_v7_RNASeQCv1.1.8_gene_median_tpm.gct*', last accessed on 06/03/2019). For the tissue-specific model case, we subset the GTEx tissues that match any of the strings defined in the '*Disease/Phenotype terms*' and '*Additional associated terms*' fields in *config.yaml* and then aggregate all values by gene across all tissues. Additionally, we assign a rank for each gene based on the aggregate expression across all matching tissues (ranks = {1, 2, 3, …} in order of decreasing expression). Genes with overall expression less than the median among all genes are assigned the same rank, equal to the total number of genes, to increase signal-to-noise ratio for the most highly-expressed genes. As for the disease-generic model case, we keep expression values across all tissues and retain them for each gene as separate features, while no rank is computed in that case.

Human Phenotype Ontology
The Human Phenotype Ontology (HPO) data are publicly available at: http://www.human-phenotype-ontology.org. We are using Build #154 from HPO to annotate disease-associated genes (last accessed on 04/03/2019). We are using by default the '*ALL_SOURCES_FREQUENT_FEATURES_\ genes_to_phenotype.txt*' file (provided by the HPO consortium), as our reference annotation file to exclude phenotypic features that are observed occasionally (present in 5–29% of the cases), rarely

(present in 1–4% of the cases) or not at all (present in 0% of the cases). Annotation is performed by selecting genes whose '*HPO-Term-Name*' contains any of the '*Disease/Phenotype terms*' and does not contain any of the 'Diseases/Phenotypes to exclude' from the *config.yaml* configuration file.

Human Protein Atlas

Human Protein Atlas data are publicly available at: https://www.proteinatlas.org/about/download (version 18.1, last accessed on 06/03/2019). We integrate two types of data from Human Protein Atlas: Normal tissue data (*normal_tissue.tsv*), which contain levels of expression for each gene in different tissues and cell types (categorical variable: 'Not detected', 'Low', 'Medium', 'High') and RNA gene data (*rna_tissue.tsv*), which contain TPM expression values for each gene by Sample (where '*Sample*' in this case is similar with the '*Tissue*' field from Normal tissue data).
We initially filter out all entries that have an 'Uncertain' value in the 'Reliability' field. For the disease-specific model case, we select all genes which contain any of the strings from '*Disease/Phenotype terms*' and '*Additional associated terms*' and do not contain any of the '*Diseases/Phenotypes to exclude*' from *config.yaml* file in their "Tissue/Sample" fields for Normal tissue and RNA gene data, respectively. Normal tissue data contain in general multiple values (levels of expression) per gene for the different cell types under each tissue type. We collapse all values for each gene by selecting the highest level found in a cell type within each tissue ('Not detected' < 'Low' < 'Medium' < 'High'). With regards to RNA gene data, we aggregate all TPM values for each gene.
As for the generic disease model, expression levels from Normal tissue data are retrieved across all tissues, the highest level is retained for each gene and eventually we convert the four original levels into two: 'Not detected' and 'Low' are both considered as 'Low' and 'Medium' and 'High' are both considered as 'High'. This transformation is performed to increase signal-to-noise ratio on this feature when looking at expression across all tissues. Finally, RNA gene data are aggregated by gene for each Sample.

InWeb_IM

InWeb_IM data (human protein-protein interaction network data, Li et al. 2017) are publicly available at: https://www.intomics.com/inbio/map.html#downloads ('*inBio_Map_core_2016_09_12.zip*', last accessed on 06/03/2019). Protein-protein interactions are characterised as 'inferred' or 'experimental' based on the validation degree recorded for each interaction in the original analysis. We untangle all interacting genes for each gene by validation type ('inferred' or 'experimental') and during analysis we record the ratio of interacting genes that belong to the seed genes (positively labelled genes) in each disease-specific run.

MGI

Data compilation is performed as described at the 'MGI' section in 'Generic Resources'. For the disease-specific model, annotation is performed by selecting all genes whose linked phenotypes contain any of the strings from '*Disease/Phenotype terms*' and '*Additional associated terms*' and do not contain any of the '*Diseases/Phenotypes to exclude*' from *config.yaml*.

MSigDB

Molecular Signatures Database (MSigDB) data are publicly available at: http://software.broadinstitute.org/gsea/downloads.jsp (v6.2, last accessed on 06/03/2019). We integrate data from the c5 gene set (gene ontology sets). For the disease-specific model case, we select all gene ontology terms that contain any of the strings from '*Disease/Phenotype terms*' and '*Additional associated terms*' and do not contain any of the '*Diseases/Phenotypes to exclude*' from *config.yaml*. As for the disease-generic model, we retain all gene ontology terms. In both cases, gene ontology terms with less than 150 associated genes (0.08% of all genes) are filtered out to reduce

the number of features with near-zero variance. In the current dataset this leaves 1,009 of 5,917 gene ontology terms.

OMIM
Online Mendelian Inheritance in Man (OMIM) data are available under licensing at: https://www.omim.org (‘*genemap2.txt*’, last accessed on 06/03/2019). We have restricted OMIM data to the subset of entries where the field ‘*Phenotypes*’ contains ‘*(3)*’, which reflects entries where the ‘*molecular basis for the disorder is known; a mutation has been found in the gene*’. OMIM annotation data are used only for extracting a disease-generic gene ranking. By default, all genes which contain ‘*(3)*’ in their ‘*Phenotypes*’ field are annotated as disease-associated genes (value ‘*All*’ in ‘*generic_classifier*’ parameter in the mantis_ml/conf/*.config* file). Additional filtered layers of positive gene data are available by specifying different values for the ‘*generic_classifier*’ in mantis_ml/conf/*.config*: a) ‘*AD*’ for selecting only genes that include ‘*Autosomal dominant*’ annotation in their ‘*Phenotypes*’ field, b) ‘*AR*’ for selecting only genes that include ‘*Autosomal recessive*’ annotation in their ‘*Phenotypes*’ field, c) ‘*AD_only*’ for selecting only genes that include ‘*Autosomal dominant*’ annotation and at the same time do not contain ‘*Autosomal recessive’* annotation in their ‘Phenotypes’ field, d)) ‘*AR_only*’ for selecting only genes that include ‘*Autosomal recessive’* annotation and at the same time do not contain ‘*Autosomal dominant’* annotation in their ‘*Phenotypes*’ field.

All string-matching operations are case insensitive.

- Disease-specific Resources (currently supported)

i. **Chronic Kidney Disease (CKD):**

CKDdb
Data from the Chronic Kidney Disease database (CKDdb) are available at: http://www.padb.org/ckddb (last accessed on 08/03/2019). We annotate each gene that has been associated with a renal disease with a True boolean flag and also record the total number of studies in CKDdb that support this evidence.

nephQTL
eQTL data for the glomerular and tubulointerstitial tissues (NephQTL) are available at: http://nephqtl.org. This database contains *cis*-eQTLs of the glomerular and tubulointerstitial tissues of the kidney found in 187 participants in the NEPTUNE cohort. For each gene and tissue, we record the expected number of eQTLs, the probability of not having eQTLs and the False Discovery Rate (FDR).

ii. **Cardiovascular Disease:**

exSNP
Data from the database of expression associated SNPs (exSNP) are available at: http://www.exsnp.org/Download (last accessed on 08/03/2019). We are integrating disease associated high confidence ($r^2 > 0.8$) eQTLs for Coronary Artery Disease and Hypertension and record the total number of eQTLs associated for each gene in each condition.

Adipose eQTLs
Data for adipose eQTLs identified at GWAS loci for cardiometabolic diseases and traits were retrieved from Civelek et. al, 2017 (Table S8). We record for each gene the total number of GWAS loci and cis eQTLs that have been associated for cardiometabolic traits.

Platelet eQTLs

Data for platelet eQTLs were retrieved from Simon et. al, 2016 (Table S2). Platelets have been shown to contribute to ischemic cardiovascular events[2]. We record for each gene the total number of heterozygous coding sites with a marginal eQTL effect ($p < 10^{-4}$) and with 10 or more reads.

Reference gene set

We have specified as our reference gene set all genes annotated by the ExAC and gnomAD (v2) consortia (n=18,626) to include all genes with sufficient evidence and annotations, to avoid contaminating our dataset with imputed features for a large subset of genes.

Assessment of mantis-ml performance with/without CKDdb-derived features

CKDdb is a resource that captures information associated with CKD by mining existing literature followed-up by manual curation. That means that it integrates various types of putative disease annotations at the gene level, however, having highly variable degrees of confidence in terms of their true relevance with the disease. Thus, true biological signal in this resource is contaminated with noise from annotations that have little support (e.g. mentioned in a single publication without any experimental validation) and does not directly represent confirmed associations of a gene with CKD (this is extracted from HPO instead). Apart from that, when we look at the feature importance during mantis-ml training for CKD, we observe that the features extracted from CKDdb are not contributing significantly to the final predictions. Specifically, the feature that indicates the presence/absence of a gene in CKDdb (CKDdb_Disease) is classified as 'rejected' by the Boruta algorithm, with regards to its feature importance (**Figure S9 A**). Similarly, the number of studies associated per gene ("CKDdb_num_of_studies") is annotated as "tentative"/"inconclusive" (**Figure S9 A**). That indicates that none of the CKDdb-extracted features contributes significantly to the predictions, again eliminating the risk of contamination of the training set with information directly associated with the output label.

Furthermore, we also trained mantis-ml without using CKDdb-derived features. We observe that mantis-ml performance with/without CKDdb-derived performance remains practically unchanged, with a very minor improvement when using the CKDdb-derived features (**Figure 24 A & B**; AUC: 0.829-0.846 versus 0.823-0.839). Similarly, feature importance analysis between the two cases highlights the same sets of features as the most contributing ones (**Figure 24 C**). Finally, the hypergeometric enrichment against the collapsing analysis hits (without using CKDdb-based features) remains highly significant, again demonstrating that mantis-ml's performance on CKD is not primarily driven by the CKDdb annotation.

Supplemental References

1. Xie, J. Stochastic Semi-supervised Learning on Partially Labeled Imbalanced Data. *Area* (2011).
2. Simon, L. M. *et al.* Integrative Multi-omic Analysis of Human Platelet eQTLs Reveals Alternative Start Site in Mitofusin 2. *Am. J. Hum. Genet.* **98**, 883–897 (2016).
